# Systematic Analysis of Expression Profiles and Prognostic Significance for *FAM83* Family in Non-small-Cell Lung Cancer

**DOI:** 10.3389/fmolb.2020.572406

**Published:** 2020-12-10

**Authors:** Junqing Gan, Qingwei Meng, Yanjing Li

**Affiliations:** ^1^Department of Medical Oncology, Harbin Medical University Cancer Hospital, Harbin, China; ^2^Department of Gastrointestinal Oncology, Harbin Medical University Cancer Hospital, Harbin, China

**Keywords:** non-small-cell lung cancer, *FAM83 family*, bioinformatics analysis, biomarker, prognostic value

## Abstract

**Background:**

Lung cancer remains a common malignancy and the leading cause of cancer-related deaths in the world. Although dramatic progress made in multimodal therapies, it still has a poor prognosis. The Family with sequence similarity 83 (*FAM83*) of poorly characterized proteins are defined by the presence of the conserved DUF1669 domain of unknown function at their N-termini, most of which significantly elevated levels of expression in multiple cancers. However, the expression and prognostic values of different *FAM83* family in lung cancer, especially in non-small-cell lung cancer (NSCLC), have not been clarified.

**Methods:**

ONCOMINE, UALCAN, GEPIA, Kaplan–Meier Plotter, cBioPortal, and STRING databases were utilized in this study.

**Results:**

The transcriptional levels of *FAM83*A/B/C/D/F/G/H were up-regulated in patients with NSCLC. A noticeable correlation was found between the over-expressions of *FAM83A/B/D/F/H* and clinical cancer stages in NSCLC patients. Besides, higher mRNA expressions of *FAM83A/B/C/D/F/H* were discovered to be expressively associated with overall survival (OS) in lung cancer patients, furthermore, *FAM83A*, *FAM83C*, and *FAM83H* in OS group achieved 0.9475/1, 0.971897/1, and 0.9454545/0.8974359 specificity/sensitivity in distinguishing short survivors from long survivors, respectively. Moreover, a high mutation rate of *FAM83* family (51%) was also observed in lung adenocarcinoma (LUAD) patients, and genetic alteration in the *FAM83* family was associated with shorter OS and disease-free survival (DFS) in LUAD patients.

**Conclusion:**

Our results indicated that *FAM83A/H* might play important roles in NSCLC tumorigenesis and might be risk factor for the survival of NSCLC patients.

## Introduction

In both sexes combined, lung cancer is the most commonly diagnosed cancer and the leading cause of cancer death (Bray et al., 2018). There are two main types of lung cancer: small cell lung cancer and non-small-cell lung cancer (NSCLC) (Alberg et al., 2005; Braicu et al., 2019), among which, NSCLC is mainly composed of lung adenocarcinoma (LUAD) and lung squamous cell carcinoma (LUSC), accounting for approximately 85% of new lung cancer cases (Wang Y. et al., 2019; Zheng et al., 2020). Efforts have been made in studying the mechanisms of the development, progression, and metastasis of NSCLC (Wang and Adjei, 2015; Popper, 2016; Inamura, 2018). However, the molecular characteristics of NSCLC, to date, remain unknown. Probing new highly specific and sensitive biomarkers and new molecular targets can not only help to elucidate the molecular mechanism of NSCLC patients, but also improve the prognosis of NSCLC patients.

The Family with sequence similarity 83 (*FAM83*) of proteins comprises of eight members of A–H, which have been categorized based on a highly conservative domain of the unknown function (DUF1669) at the N-terminus, while the C-terminal regions varied greatly among the different family members (Bartel et al., 2016; Snijders et al., 2017; Bozatzi and Sapkota, 2018). The first detailed analysis of the *FAM83* proteins in transformation resulted from the separate identification of *FAM83A* and *FAM83B* in distinct genetic screens performed by the Bissell (Lee et al., 2012) and Jackson Laboratories (Cipriano et al., 2012), respectively. Subsequently, diverse studies have confirmed that members of *FAM83* family with overexpression or dysregulation play important role in cell growth, proliferation, metastasis, and resistance to precision therapies (Bartel et al., 2016). Zhou et al. (2019) demonstrated that *FAM83A* accelerated NSCLC cell migration and invasion through the activation of epithelial-mesenchymal transition (EMT) via PI3K/ATK/Snail signaling. Moreover, a study verified that miR-143 restrained the proliferation, migration, and invasion of esophageal squamous cell carcinoma (ESCC) cells and also lead to G1/G0 phase arrest via down-regulation of *FAM83F* expression (Mao et al., 2016). A comprehensive study of *FAM83* family members in NSCLC will help to uncover the molecular mechanisms involved in the development of NSCLC and could unveil novel prognostic and therapeutic targets for the intractable disease.

In the past few years, studies have demonstrated abnormal expression in some members of the *FAM83* family and their prognostic value. For example, Fuziwara et al. (2019) reported that *FAM83F*, a novel oncogenic protein, was overexpressed in thyroid cancer and cross-regulated MAPK and TGF signaling pathways to promote the biological behavior and differentiation of thyroid follicular cells. *FAM83B* was recently identified as a novel oncogene involved in activating *CRAF/MAPK* signaling and driving epithelial cell transformation (Cipriano et al., 2012). Furthermore, evidence suggested that the upregulation of *FAM83* members was remarkably correlated with an elevated breast tumor grade and decreased overall survival (OS) (Cipriano et al., 2014). Nevertheless, the role of distinct *FAM83* family members remained unknown in the development and progression of NSCLC. In this present study, bioinformatics was performed initially to address this problem by analyzing the expression, prognosis, and mutations of different *FAM83* family members and their relations with individual cancer stages in NSCLC patients. Furthermore, we also analyzed the predicted functions and pathways of the mutations in the *FAM83* family as well as their 80 frequently altered genes.

## Materials and Methods

### Ethics Statement

The study has been admitted by the Institutional Review Board of The Harbin Medical University. Since all the data were retrieved from the online databases, it could be affirmed that all written informed consent had already been obtained.

### ONCOMINE Database

ONCOMINE database^1^ is a publicly accessible online cancer microarray database, which provides a genome-wide expression analysis (Rhodes et al., 2004). It was utilized to analyze the transcription levels of *FAM83* family between disparate cancer tissues and their corresponding adjacent normal control samples. In this study, the cell color is determined by the best gene rank percentile for the analysis within the cell, and Q–Q graph and histogram were used to detect whether the sample data obeyed normal distribution, then the Student’s *t*-test was applied to generate a *p*-value. A *p*-value of 0.05, a fold change of 2, and a gene rank in the top 10% were set as the significance thresholds.

### UALCAN

UALCAN^2^ is an omnibus, user-friendly, and interactive web resource based on The Cancer Genome Atlas (TCGA) level 3 RNA-seq and clinical data from 31 cancer types (Chandrashekar et al., 2017). In our study, UALCAN was used to illustrate the distinct expression levels of tumor and normal tissues. Student’s *t*-test was used to generate a *p*-value and the *p*-value cutoff was 0.05.

### GEPIA

GEPIA^3^ is a newly developed interactive web server for elaborating the RNA sequencing expression data of 9736 tumors and 8587 normal samples from the TCGA and Genotype-tissue Expression dataset, utilizing a standard processing pipeline. GEPIA offers customizable functions such as tumor/normal differential expression analysis, profiling according to cancer types or pathological stages, patient survival analysis, similar gene detection, correlation analysis, and dimensionality reduction analysis (Tang et al., 2017) For analysis, “Multiple Gene Comparison” was used to evaluate the multiple gene comparison analysis of *FAM83* family. “Expression DIY” was utilized to compare eight *FAM83* family members’ association with clinicopathologic parameters. The Student’s *t*-test was used to generate a *p*-value and the *p*-value cutoff was 0.05.

### Kaplan–Meier Plotter

The Kaplan Meier plotter^4^ is able to assess the effect of 54 k genes on survival in 21 cancer types. The largest datasets include breast, ovarian, lung, and gastric cancer (Nagy et al., 2018). In this study, it was used to evaluate the prognostic value of *FAM83* family mRNA expression in which cancer patients were split into high and low expression group based on median values of mRNA expression and validated by K-M survival curves, with the hazard ratio (HR) with 95% confidence intervals (CI) and log-rank *p*-value. The statically significant difference was considered when a *p*-value is <0.05.

### TCGA Data and cBioPortal

The Cancer Genome Atlas had both sequencing and pathological data on 30 different cancers (2012). The LUAD (TCGA, Firehose Legacy) dataset including data from 515 cases with pathology reports was selected for further analyses of *FAM83* family using the cBioPortal^5^), which is a comprehensive web resource for exploring, visualizing, and analyzing multidimensional cancer genomics data. In this study, we analyzed the genomic profiles of eight *FAM83* family members, which contained mutations, putative copy-number alterations from GISTIC, and mRNA Expression z-Scores (RNASeq V2 RSEM) with a *z*-score threshold of ±1.8 (Cerami et al., 2012; Gao et al., 2013). Genetic mutations in *FAM83* family and their association with OS and disease free survival (DFS) of lung cancer patients were displayed as Kaplan–Meier plots, and the log-rank test was performed to identify the significance of the diversity between the survival curves, and when a *p*-value is <0.05, the difference was considered statically significant. Co-expressed genes of *FAM83* family was performed with the “Co-expression” module of cBioportal. Pearson’s correlation coefficient was used to investigate the correlation between *FAM83* family and co-expressed genes and top ten co-expressed genes of each *FAM83* family with the largest Pearson’s correlation coefficient were listed.

### STRING

STRING^6^ is a database of known and predicted protein–protein interactions (PPI) (Szklarczyk et al., 2019). Herein, to detect the role of *FAM83* family co-expressed genes, the online database of STRING was applied to analyze associations among the PPI network of *FAM83* family co-expressed genes, and the species were set to Homo sapiens and a combined score of >0.7 was considered statistically significant. The nodes meant proteins; the edges meant the interaction of proteins and we hide disconnected nodes in the network.

### DAVID

Functions of *FAM83* family mutations and 80 genes significantly related to *FAM83* family mutations were analyzed by the Gene Ontology (GO) and Kyoto Encyclopedia of Genes and Genomes (KEGG) in the Database for Annotation, Visualization, and Integrated Discovery (DAVID)^7^ (Huang da et al., 2009a,b). Gene ontology analyses focus on three domains: biological processes (BP), cellular components (CC), and molecular functions (MF), and such analyses are commonly used to predict the functional roles of *FAM83* family mutations and 80 genes significantly associated with *FAM83* family mutations, while KEGG analysis can define the pathways related to the *FAM83* family mutations and 80 co-expressed genes associated with *FAM83* family mutations. Only terms with *p*-value of <0.05 were considered as significant.

## Results

### Aberrant Expression of *FAM83* Family in Patients With NSCLC

**FIGURE 1:**
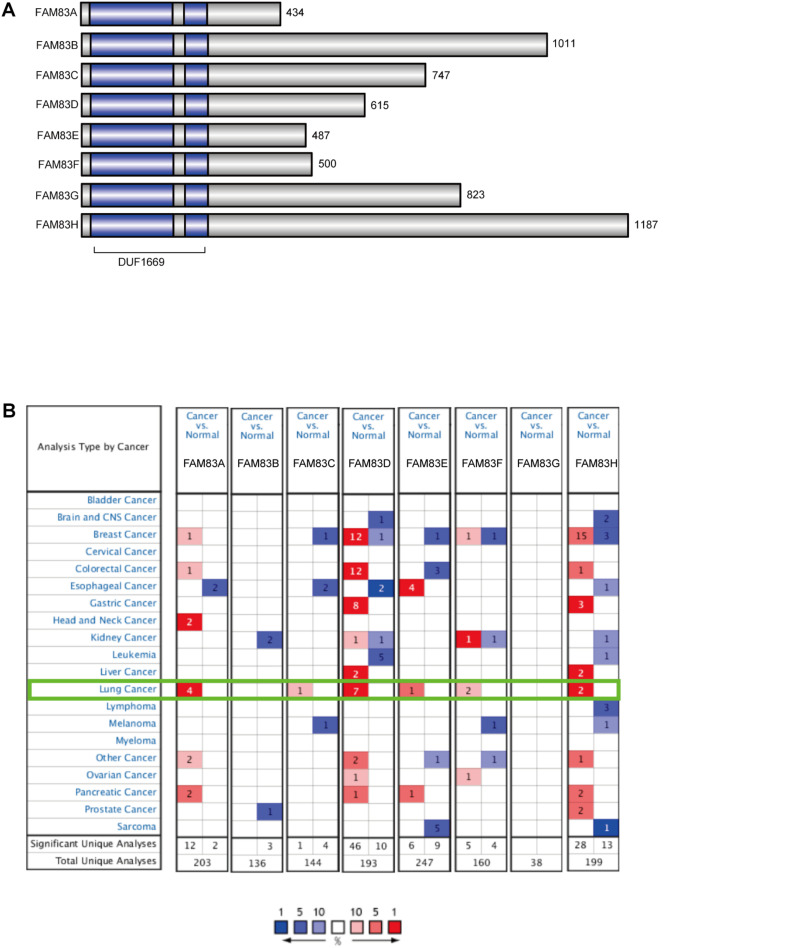
The expression of *FAM83* family in distinct types of cancer diseases (ONCOMINE database). **(A)** The pattern diagram of the human *FAM83* family of proteins and the conserved domain of unknown function, DUF1669 that characterizes them. **(B)** The figure shows the numbers of datasets with statistically significant mRNA up-regulation (red) or down-regulated expression (blue) of *FAM83* family. Student’s *t*-test was used to compared the different transcriptional. Cutoff of *p*-value and fold change were as following: *p*-value: 0.01, fold change: 2, gene rank: 10%, data type: mRNA.

**FIGURE 2:**
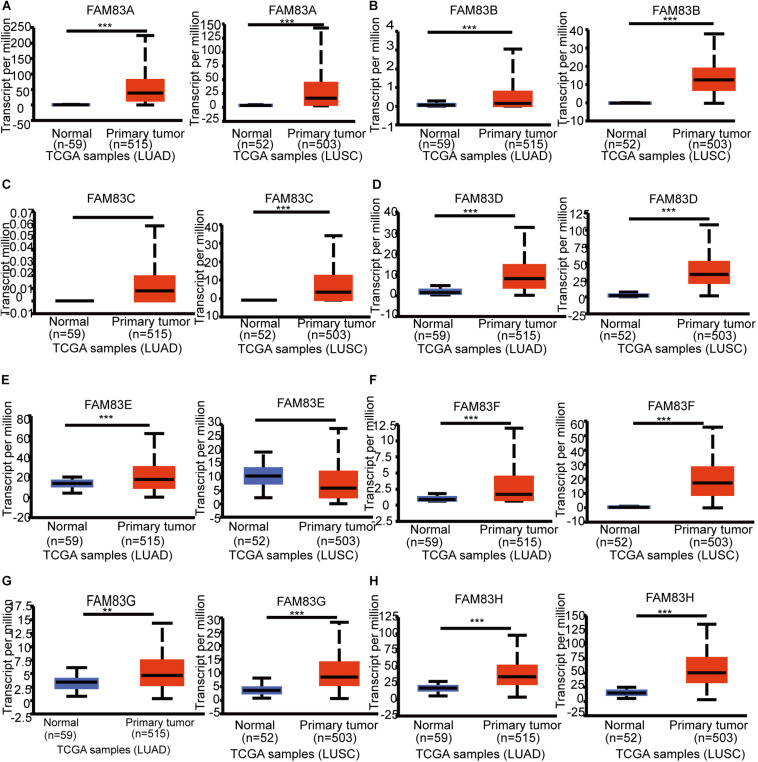
The mRNA expression of diverse *FAM83* family members in NSCLC tissues and adjacent lung tissues (UALCAN*).* mRNA expressions of *FAM83A/B/C/D/F/G/H* were found to be over-expressed in LUAD and LUSC tissues compared to normal samples **(A–D, F–H)**. *FAM83E* was higher in LUAD tissues than in lung tissues, whereas and the expression level of *FAM83E* was lower in the LUSC tissues than in lung tissues **(E)**. ***p < 0.001, **p < 0.01.

**FIGURE 3:**
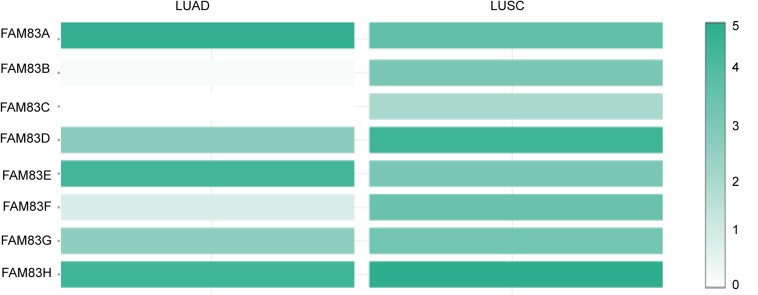
The relative level of *FAM83* family in NSCLC. *FAM83A* was the highest expression in LUAD and *FAM83H* was the highest in LUSC.

The *FAM83* family genes located at definite genomic sites (Table 1; Bartel et al., 2016) and these proteins are described by the presence of the conserved DUF1669 domain of unknown function at their N-terminal, whereas the rest of the proteins vary in length and are not conserved between members (Figure 1A; Bozatzi and Sapkota, 2018). ONCOMINE database (see text footnote 1) and UALCAN (see text footnote 2) were utilized to explore expression levels of *FAM83* family in cancers with those in normal samples. We used the Q–Q graph and histogram to detect whether the sample data obeyed normal distribution. As shown in Supplementary Figure 1, the sample data basically conforms to the normal distribution as we anticipated. Subsequently, we selected *t*-test as the main statistical method when analyzing the raw data in the ONCOMINE database. The results in Figure 1B showed that the mRNA expression levels of *FAM83A/C/D/E/F/H* were remarkably up-regulated in lung cancer tissues in multiple datasets. In the Okayama Lung dataset (Okayama et al., 2012), *FAM83A* overexpression was detected in LUAD (*N* = 226) compared with normal tissues (*N* = 20) with a fold change of 3.742 (*p* = 2.61E-22), while Hou found a 4.448-fold increase in *FAM83A* mRNA expression in 45 LUAD samples (*p* = 6.75E-13) (Hou et al., 2010), moreover, Selamat investigated a 3.928-fold increase in *FAM83A* mRNA expression in 58 LUAD tissues (*p* = 4.19E-17) (Selamat et al., 2012) and in the Garber dataset, the mRNA expression of *FAM83A* in LUAD (*N* = 40) was increased (by a fold change of 2.238, *p* = 1.00E-03) (Table 2; Garber et al., 2001). What is more, significant up-regulation of *FAM83C* was also found in LUAD tissues compared to normal tissues. The result from the Hou dataset unfolded that there was a 5.995-fold (*p* = 3.11E-09) increase in *FAM83C* mRNA expression in 27 LUAD tissues (Table 2; Hou et al., 2010). In the Garber dataset (Garber et al., 2001), *FAM83D* was overexpressed in all kinds of the lung cancer (*N* = 54) than in the normal tissues: by a fold change of 11.481, (*p* = 2.76-10) in LUSC, by a fold change of 6.635, (*p* = 4.07E-11) in LUAD, by a fold change of 7.297, (*p* = 2.00E-03) in large-cell lung carcinoma, by a fold change of 9.244 (*p* = 4.00E-03) in small cell lung carcinoma, respectively. Likewise, the transcription levels of *FAM83D* in 27 LUSC, 45 LUAD, and 19 large cell lung carcinoma (Hou et al., 2010) were higher than those in lung samples (*N* = 65), and their fold changes were 7.532 (*p* = 2.80E-20), 3.072 (*p* = 1.26E-11), 5.161 (*p* = 7.93E-06), respectively (Table 2). In the Selamat dataset (Selamat et al., 2012), the mRNA expression of *FAM83E i*n LUAD (*N* = 58) increased with a fold change of 2.043 (*p* = 2.23E-14). A similar trend of *FAM83F* was shown in the Hou database (Hou et al., 2010). *FAM83F* was notably up-regulated in 27 LUSC and 19 Large Cell Lung Carcinoma, with a fold change of 3.110 (*p* = 3.25E-08) and 2.328 (*p* = 3.49E-05), respectively (Table 2). The transcriptional levels of *FAM83H* in 58 LUAD (fold change = 2.715, *p* = 1.07E-23) and in 27 LUSC (fold change = 2.493, *p* = 6.81E-14) considerably differed from those in the normal samples in the Selamat (Selamat et al., 2012) and Hou datasets (Hou et al., 2010), respectively (Table 2). Furthermore, using the UALCAN (see text footnote 2), we compared the mRNA expression of *FAM83* family between 515 LUAD and 59 normal tissues, 503 LUSC and 52 normal tissues. The results in Figure 2 discovered that *FAM83A/B/C/D/F/G/H* were higher in LUAD and LUSC tissues than in lung tissues. Moreover, *FAM83E* was higher in LUAD than in lung tissues, nevertheless the expression level of *FAM83E* was lower in the LUSC tissues than in lung tissues. Besides, we also contrast the relative expression levels of *FAM83* family in LUAD and LUSC tissues and determined that among all the factors we evaluated, *FAM83A* was the highest expression in LUAD and *FAM83H* was the highest in LUSC (Figure 3). Taken together, our results showed that transcriptional expressions of *FAM83A/B/C/D/F/G/H* were over-expressed in patients with NSCLC.

**TABLE 1:** The distinct chromosomal locations of FAM83A–H members.

**FAM83 proteins**	**FAM83A**	**FAM83B**	**FAM83C**	**FAM83D**	**FAM83E**	**FAM83F**	**FAM83G**	**FAM83H**
Chromosomal location	8q24.13	6p12.1	20q11.22	20q11.23	19q13.33	22q13.1	17p11.2	8q24.3

**TABLE 2:** Remarkable changes of *FAM83* family expression in transcription level between lung cancer and normal lung tissues (ONCOMINE).

	**Types of lung cancer vs. lung**	**Fold change**	***p*-Value**	***t*-test**	**References**
*FAM83A*	Lung adenocarcinoma vs. normal	3.742	2.61E-22	14.243	Okayama et al., 2012
	Lung adenocarcinoma vs. normal	4.448	6.75E-13	9.589	Hou et al., 2010
	Lung adenocarcinoma vs. normal	3.928	4.19E-17	11.668	Selamat et al., 2012
	Lung adenocarcinoma vs. normal	2.238	1.00E-03	4.083	Garber et al., 2001
*FAM83C*	Squamous cell lung carcinoma vs. normal	5.995	3.11E-09	8.423	Hou et al., 2010
*FAM83D*	Squamous cell lung carcinoma vs. normal	11.481	2.76-10	12.486	Garber et al., 2001
	Lung adenocarcinoma vs. normal	6.635	4.07E-11	12.14	Garber et al., 2001
	Large cell lung carcinoma vs. normal	7.297	2.00E-03	6.314	Garber et al., 2001
	Small cell lung carcinoma vs. normal	9.244	4.00E-03	7.642	Garber et al., 2001
	Squamous cell lung carcinoma vs. normal	7.532	2.80E-20	15.532	Hou et al., 2010
	Lung adenocarcinoma vs. normal	3.072	1.26E-11	7.93	Hou et al., 2010
	Large cell lung carcinoma vs. normal	5.161	7.93E-06	5.639	Hou et al., 2010
*FAM83E*	Lung adenocarcinoma vs. normal	2.043	2.23E-14	9.288	Selamat et al., 2012
*FAM83F*	Squamous cell lung carcinoma vs. normal	3.110	3.25E-08	7.36	Hou et al., 2010
	Large cell lung carcinoma vs. normal	2.328	3.49E-05	5.088	Hou et al., 2010
*FAM83H*	Lung adenocarcinoma vs. normal	2.715	1.07E-23	13.761	Selamat et al., 2012
	Squamous cell lung carcinoma vs. normal	2.493	6.81E-14	11.353	Hou et al., 2010

### Correlation Between mRNA Expression of Different *FAM83* Family and Tumor Stages of NSCLC Patients

**FIGURE 4:**
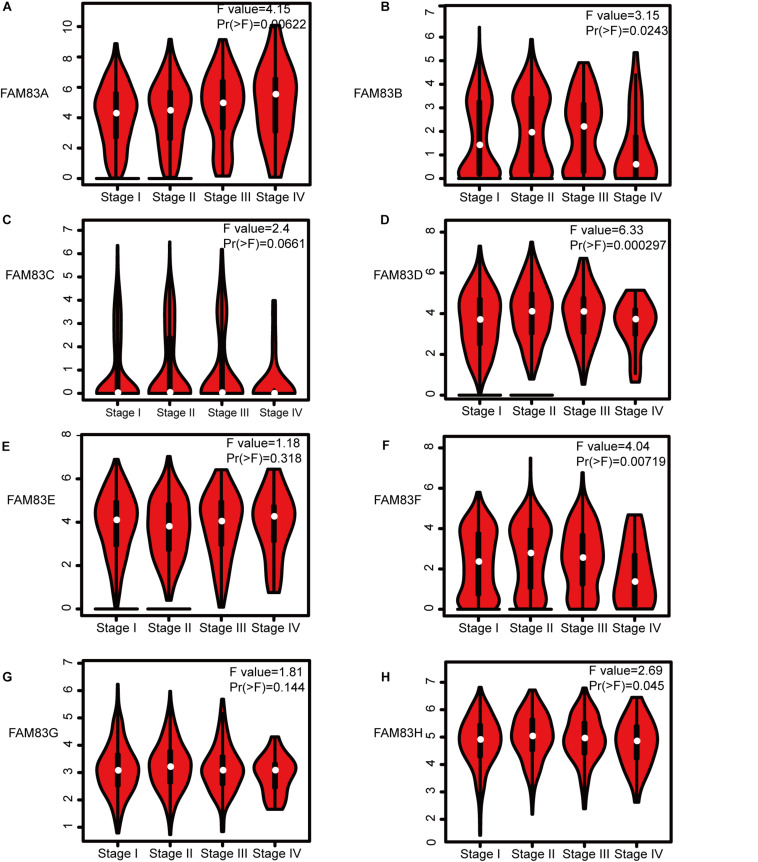
Correlation between *FAM83* family expression and tumor stage in NSCLC patients (GEPIA). The mRNA expressions of *FAM83A/B/D/F/H* were significantly related to patients’ individual cancer stages **(A,B,D,F,H)**, while mRNA expressions of *FAM83C/E/G* were not associated with patients’ individual cancer stages **(C,E,G)**.

Next, we analyzed the relationship between the mRNA expression of different *FAM83* family members with patients’ individual cancer stages of LUAD and LUSC patients by GEPIA (see text footnote 3). *FAM83A, FAM83B, FAM83D, FAM83F*, and *FAM83H* groups appreciably varied, whereas *FAM83C*, *FAM83E*, and *FAM83G* groups did not markedly differ (Figure 4). The reason why the mRNA expressions of *FAM83C/E/G* in NSCLC seemed to not significantly diverge may be as a result of small sample size. In short, the results above suggested that mRNA expressions of *FAM83A/B/D/F/H* were obviously related to patients’ individual cancer stages, and patients who were in more advanced cancer stages were inclined to express higher mRNA expression of *FAM83A/B/D/F/H.*

### Prognostic Features of *FAM83* Family in Patients With Lung Cancer

**FIGURE 5:**
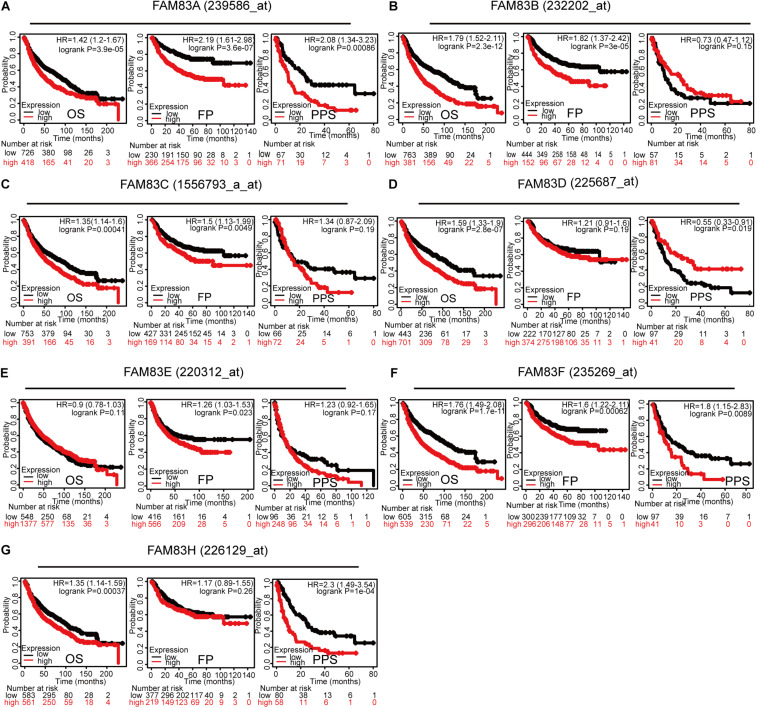
Prognostic feature of mRNA expression of distinct *FAM83* family members in lung cancer patients (Kaplan–Meier plotter). The OS, FP, and PPS survival curves comparing patients with high (red) and low (black) *FAM83* family member expression in lung cancer were plotted using the Kaplan–Meier plotter database at the threshold of *p*-value of <0.05. The association between Prognostic value and **(A)** FAM83A, **(B)** FAM83B, **(C)** FAM83C, **(D)** FAM83D, **(E)** FAM83E, **(F)** FAM83F, **(G)** FAM83H protein expression.

By means of data mining in the Kaplan–Meier plotter (2015 version; see text footnote 4), prognostic value of *FAM83* family mRNA for lung cancer patients including OS, progression-free survival (FP), and post-progression survival (PPS) were investigated, respectively. It could be seen that, in each cohort, patients were divided into low and high risk group based on cutoff value (Figure 5 and Table 3). Reversed relationship was shown between OS and the mRNA levels of *FAM83A/B/C/D/F/H*; however, there was no significant association in lung cancer between OS and *FAM83E*. There was no significant correlation in lung cancer between FP and either *FAM83D* or *FAM83H*, whereas high *FAM83A/B/C/E/F* mRNA expression led to a reduced FP. In addition, increased *FAM83A/D/F/H* mRNA expression levels were associated with PPS. Unfortunately, by querying this public database, we did not find the relationship between the mRNA expression of *FAM83G* and prognosis. We also used scatter diagram to determine appropriate cutoff values of *FAM83* family in OS group, FP group, and PPS group and to clarify their clinical significance (data from Supplementary Table 2). The scatter diagram in Supplementary Figure 2 revealed the statically significant difference (*p*-value < 0.05) when 362 and 1030 was adopted as the cutoff of *FAM83A* among all patients in OS group and FP group. And when *p*-value is <0.05, cutoff value of *FAM83B* (in FP group) is 13, *FAM83C* (in OS group and FP group) is 17 and 30, respectively, *FAM83D* (in PPS group) is 993, *FAM83E* (in FP group) is 16, *FAM83F* (in FP group) is 66, and *FAM83H* (in OS group) is 804. As shown in Supplementary Table 3, we described *FAM83* family prognostic potential: *FAM83A* in OS group and PPS group achieved a 0.9475/1 and 0.952381/0.7586207 specificity/sensitivity in distinguishing short survivors from long survivors, accordingly. *FAM83B* in FP group achieved a 0.8235294/0.9910714 specificity/sensitivity in distinguishing short survivors from long survivors. *FAM83C* in OS group and FP group achieved a 0.971897/1 and 1/0.9810427 specificity/sensitivity in distinguishing short survivors from long survivors, respectively. *FAM83D* in PPS group achieved a 0.8333333/0.9661017 specificity/sensitivity. *FAM83E* in FP group achieved a 0.3424658/0.9504717 specificity/sensitivity in discriminating short survivors from long survivors. *FAM83F* in FP group achieved a 0.9148936/0.6 specificity/sensitivity in discriminating short survivors from long survivors. FAM83H in OS group achieved a 0.9454545/0.8974359 specificity/sensitivity in distinguishing poor survival.

**TABLE 3:** The prognostic values of *FAM83* family members in lung cancer patients (Kaplan–Meier plotter).

***+FAM83* family**	**OS**				**FP**				**PPS**			
	
	**Cases**	**HR**	**95% CI**	***p*-value**	**Cases**	**HR**	**95% CI**	***p*-value**	**Cases**	**HR**	**95% CI**	***p*-value**
FAM83A (239586_at)	**1927**	**1.42**	**1.2–1.67**	**3.9e-05**	**982**	**2.19**	**1.61–2.98**	**3.6e-07**	**344**	**2.08**	**1.34–3.23**	**0.00086**
FAM83B (232202_at)	**1927**	**1.79**	**1.52–2.11**	**2.3e-12**	**982**	**1.82**	**1.37–2.42**	**3e-05**	344	0.73	0.47–1.12	0.15
FAM83C (1556793_a_at)	**1927**	**1.35**	**1.14–1.6**	**0.00041**	**982**	**1.5**	**1.13–1.99**	**0.0049**	344	1.34	0.87–2.09	0.19
FAM83D (225687_at)	**1927**	**1.59**	**1.33–1.9**	**2.8e-07**	982	1.21	0.91–1.6	0.19	**344**	**0.55**	**0.33–0.91**	**0.019**
FAM83E (220312_at)	1927	0.9	0.78–1.03	0.11	**982**	**1.26**	**1.03–1.53**	**0.023**	344	1.23	0.92–1.65	0.17
FAM83F (235269_at)	**1927**	**1.76**	**1.49–2.08**	**1.7e-11**	**982**	**1.6**	**1.22–2.11**	**0.00062**	**344**	**1.8**	**1.15–2.83**	**0.0089**
FAM83H (226129_at)	**1927**	**1.35**	**1.14–1.59**	**0.00037**	982	1.17	0.89–1.55	0.26	**344**	**2.3**	**1.49–3.54**	**1e-04**

*Bold values mean *p* < 0.05.*

### Genetic Mutations in *FAM83* Family and Their Associations With OS and Disease-Free Survival of Lung Cancer Patients

**FIGURE 6:**
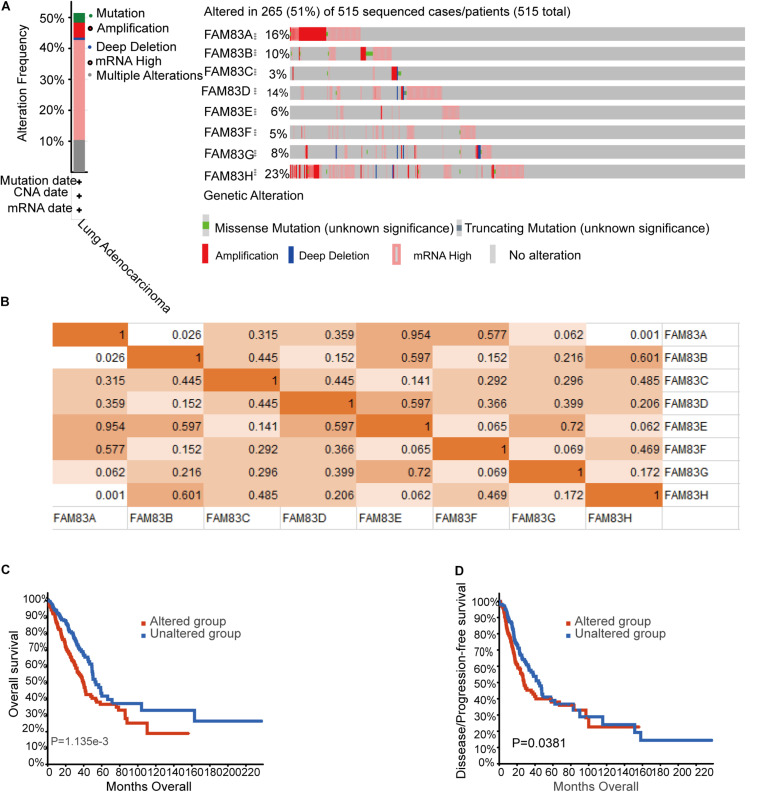
Genetic mutations in *FAM83* family and their association with OS and DFS of NSCLC patients (cBioPortal). **(A)** Summary of alterations in different expressed *FAM83* family in LUAD. **(B)** Correlations of different *FAM83* family members with each other in LUAD. **(C)** Genetic alterations in *FAM83* family were related to shorter OS and **(D)** DFS of LUAD patients.

Epigenetic alteration plays a vital role in early malignancies (Cancer Genome and Atlas Network, 2012). Then, we analyzed the *FAM83* family alterations and correlations by using the cBioPortal online tool (see text footnote 5) for LUAD (TCGA, Firehose Legacy), in which *FAM83* family was varied in 265 samples out of 515 patients with LUAD (51%) (Figure 6A). *FAM83H*, *FAM83A*, *FAM83D*, and *FAM83B* were the top four genes with genetic alterations, and their mutation rates were 23, 16, 14, and 10%, respectively. Besides, we also figured the correlations between *FAM83* family by analyzing their mRNA expression (RNA Seq V2 RSEM) via the cBioPortal online tool for LUAD (TCGA, Firehose Legacy), which contained Pearson’s correction. The consequences exposed noteworthy and positive relationship in the following *FAM83A* with *FAM83B* and *FAM83H* (Figure 6B). Furthermore, we analyzed the relationship of genetic alteration in *FAM83* family with OS and DFS of lung cancer patients. Results from the Kaplan–Meier plot and log-rank test uncovered that genetic alteration in *FAM83* family was related to shorter OS (Figure 6C, *p* = 1.135E-3) and DFS (Figure 6D, *p* = 0.0381) of lung cancer patients. These discovery observed that the change of *FAM83* family gene may also crucially affect the prognosis of lung cancer patients.

### GO and KEGG Enrichment Analysis of *FAM83* Family and Their 80 Co-expression Genes in NSCLC Patients

**FIGURE 7:**
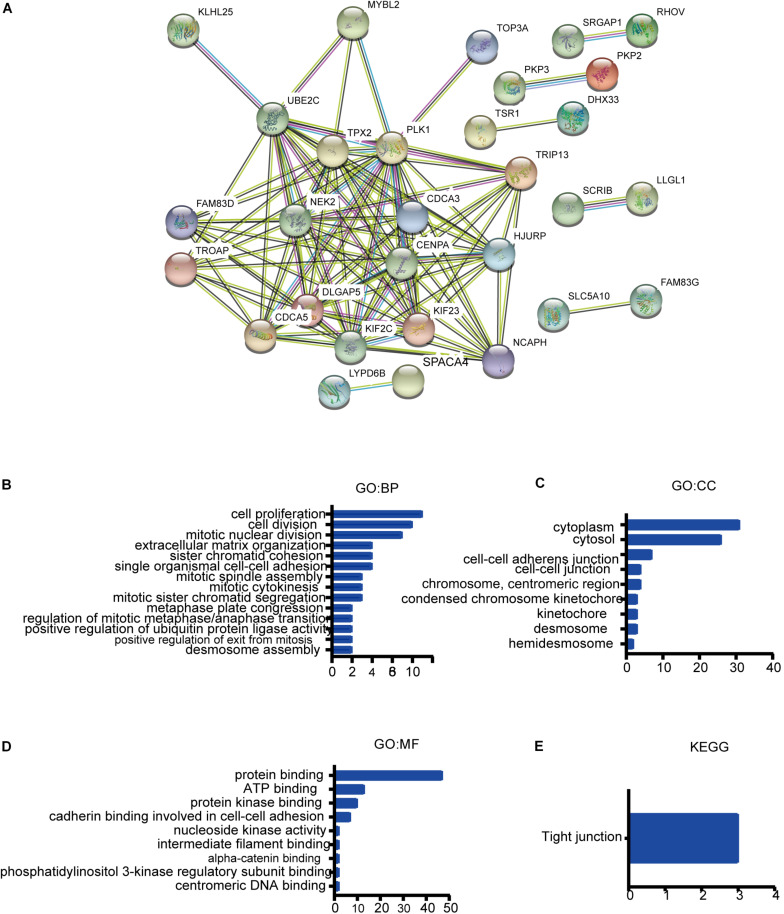
GO and KEGG enrichment analysis of *FAM83* family and their 80 co-expression genes in NSCLC patients. (STRING and DAVID). **(A)** PPI network. The nodes meant proteins; the edges meant the interaction of proteins **(B)** BP. **(C)** CC. **(D)** MF. **(E)** KEGG.

After analyzing the genetic alterations in *FAM83* family and their prognostic value in NSCLC patients, we then analyzed 80 co-expression genes that were significantly associated with *FAM83* family mutations with the “Co-expression” module of cBiopotal and listed them in Supplementary Table 1.Subsequently, we constructed an integrated network by STRING (see text footnote 6). The results in Figure 7A exposed that the cell cycle-related genes, including CDCA5 and CDCA3, and chromosome segregation participant genes, such as KIF23 and KIF2C, were closely connected with *FAM83* family alterations. In addition, GO and KEGG in DAVID (see text footnote 7) were employed to analyze the potential role of *FAM83* family and their 80 co-expression genes. As shown in Figures 7B–E, we found that BP such as GO:0007062 (sister chromatid cohesion), GO:0030198 (extracellular matrix organization), GO:0007067 (mitotic nuclear division), GO:0051301 (cell division), and GO:0008283 (cell proliferation) were remarkably regulated by the *FAM83* family mutations in NSCLC (Figure 7B). Moreover, CC, including GO:0005737 (cytoplasm), GO:0005829 (cytosol), GO:0005913 (cell–cell adherens junction), and GO:0005911 (cell–cell junction) were significantly associated with the *FAM83* family alterations (Figure 7C). Furthermore, *FAM83* family mutations also prominently affected the MF, such as GO:0005515 (protein binding), GO:0005524 (ATP binding), GO:0019901 (protein kinase binding), and GO:0098641 (cadherin binding involved in cell–cell adhesion). In the KEGG analysis, only hsa04530 (Tight junction) was greatly related to the functions of *FAM83* family in NSCLC (Figure 7E).

## Discussion

While all eight *FAM83* members share the highly conserved DUF1669, they also possess unique C-terminus of variable length and otherwise lack any significant homology beyond the DUF1669 (Fulcher et al., 2018), explaining the reason of poor enrichment function. Thus, we consider that the DUF1669 is the carcinogenetic part of the *FAM83* proteins (Cipriano et al., 2014), and therefore the most attractive therapeutic target. Based on the divergent sizes of the *FAM83* proteins (ranging from 434–1179 amino acids), each protein likely has additional DUF1669-independent cellular functions (Bartel et al., 2016). Therefore, the present study is the first time to analyze the mRNA expression, mutation, and prognostic roles of different *FAM83* factors in NSCLC.

*FAM83A* has been reported to play a role in promoting cancer and reducing drug sensitivity in a variety of cancer (Liu et al., 2020). Parameswaran et al. (2019) indicated that *FAM83A* was overexpressed in human and murine pancreatic cancers and protected from cell death in pancreatic cancer cells by activating essential MEK/ERK survival signaling. Similarly, a study on breast cancer found that overexpression of *FAM83A* also enhanced cell proliferation and invasiveness, while increasing resistance to tyrosine kinase inhibitors (TKIs). Conversely, down-regulation of *FAM83A* restrained the malignant phenotype, reduced tumor growth in immunocompromised mice, and increased TKI responsiveness (Grant, 2012). In the past few years, the expression of *FAM83A* was found to be noticeably over-expressed in lung cancer, moreover, *FAM83A* was related to an advanced TNM stage and poor prognosis, and regulated the Wnt and Hippo signaling pathways and EMT process to accelerate lung cancer cells’ proliferation and invasion (Zheng et al., 2020). In our present study, we found that the up-expression of *FAM83A i*n NSCLC tissues and that the mRNA expression of *FAM83A* was dramatically related to patients’ individual cancer stages. What was more, higher mRNA expression of *FAM83A* led to a reduced OS, FP, and PPS of lung cancer patients, which were similar to the findings of Yu et al.’s (2020) studies. Further analysis determined that the patients were categorized by cutoffs of 362 for *FAM83A* low expression and high expression and achieved a 0.8333333/0.9661017 specificity/sensitivity in distinguishing poor survival from OS. These discoveries raise the possibility that this gene may play a remarkable role in the tumorigenesis of NSCLC and might become a new therapeutic target in the future.

Studies from Okabe et al. (2015) showed that *FAM83B* was a novel biomarker for the diagnosis and prognosis of LUSC. Furthermore, Shen et al. (2017) discovered that the expression of *FAM83B* was observably enhanced both in pancreatic ductal adenocarcinoma cell lines and tumor tissues. *FAM83B* expression was positively related with advanced clinical stage and poor vital status. Higher *FAM83B* expression led to a reduced OS in pancreatic ductal adenocarcinoma patients, regardless of the lymphatic metastasis status. What was more, down-regulation of *FAM83B* contributed to G0/G1 phase arrest and inhibition of cell proliferation (Shen et al., 2017). Similarly, Cipriano et al. (2012) found that *FAM83B* expression was dramatically elevated in cancer, increased tumor grade, and decreased OS. In our study, *FAM83B* expression levels in NSCLC tumor tissues were markedly higher than that in non-tumor tissues. We also certified that *FAM83B* expression was dramatically related to tumor stage in patients with NSCLC. Reduced OS and FP with high FAM83B expression, indicating that *FAM83B* may be a prognosis signature and potential oncogene of NSCLC.

In recent years, significant overexpression of *FAM83D* has been found in a variety of cancers, including lung cancer, hepatocellular carcinoma, gastric cancer, invasive ovarian cancer, and colorectal cancer (Liao et al., 2015; Mu et al., 2017; Wang F. et al., 2019; Zhang et al., 2019; Yin et al., 2020). Studies from Mu et al. (2017) showed that the *FAM83D* mRNA expression level was markedly up-regulated in colorectal cancerous tissues and cell lines. Mechanistically, *FAM83D* suppression promoted colorectal cancer cell apoptosis, inhibited cell proliferation, cell migration, and invasion through inhibiting the FBXW7/Notch1 signal pathway (Mu et al., 2017). Moreover, the up-regulation of *FAM83D* mRNA and protein levels were detected in human gastric cancer tumor tissues and cell lines, comparing with the adjacent normal tissues and non-malignant gastric epithelial cell lines, respectively, and that reversed relationship shown between OS, DFS, and *FAM83D* in gastric cancer patients (Wang F. et al., 2019). In our present research, *FAM83D* expression was higher in NSCLC tissues than in normal ones and was obviously and negatively related to tumor stage in NSCLC patients. Furthermore, an elevated level of FAM83D was markedly associated with worse OS and PPS in NSCLC patients. These discoveries raise the possibility that *FAM83D* may be a prognosis signature and potential oncogene of NSCLC.

The overexpression of *FAM83F* protein and mRNA had been found in ESCC tissues in recent years. Moreover, miR-143 restrained the expression of FAM83F and thereby refrained the proliferation, migration, and invasion and induced G1/G0 phase arrest of ESCC cells (Mao et al., 2016). Besides, *FAM83F* was also found high expression in papillary thyroid cancer (Fuziwara et al., 2019). Furthermore, the functional study had shown that MiR-650 expression in glioma tissues was greatly decreased, while the expression of *FAM83F* was remarkably up-regulated and MiR-650 could promote cell proliferation through facilitating the expression of *FAM83F* (Xu et al., 2018). In our present study, we verified that the expression of *FAM83F* in NSCLC tissues was higher than that in normal tissues. We also confirmed that *FAM83F* expression was distinctly correlated with tumor stage in patients with NSCLC patients. A high *FAM83F* expression was prominent correlated with the poor OS, FP, and PPS in all of the patients with lung cancer. These discoveries raise the possibility that *FAM83F* may be a prognosis signature and potential oncogene of NSCLC.

A study has discovered that the expression of *FAM83H* was increased in osteosarcomas tissues and tended to reduce the survival of osteosarcoma patients by univariate analysis. This study also discovered that *FAM83H* regulated the progression of osteosarcomas via a mechanism involving the stabilization of β-catenin and the promotion of proliferation and invasiveness of osteosarcomas (Kim et al., 2019). Functional study had shown that *FAM83H* was up-regulated in liver cancer cells, and nuclear expression of *FAM83H* signed shorter survival of HCC patients. They also considered FAM83H as an oncogene in liver cancer, which is closely associated with the MYC (Kim et al., 2017). Likewise, in our investigation, markedly higher mRNA expression of *FAM83H* was also found in NSCLC tissues, mRNA expression of *FAM83H* was remarkably correlated with patients’ individual cancer stages. Accordingly, higher mRNA expression of *FAM83H* was also considerably related with shorter OS and PPS of lung cancer patients. Consistent with our results, Chen et al. (2019) demonstrated that *FAM83H* is increased in cervical cancer tissues and that high *FAM83H* expression tends to worsen the OS. In addition, suppression of *FAM83H* refrained proliferation, colony formation, migration, and invasion of CC cells through inactivating PI3K/AKT pathway (Chen et al., 2019). Further analysis demonstrated that the patients were categorized by cutoffs of 804 for *FAM83H* low expression and high expression and achieved a 0.9454545/0.8974359 specificity/sensitivity in distinguishing poor survival from OS. Together with the other findings discussed above, our results suggested that *FAM83H* might play an oncogenic role in NSCLC and those findings might provide theoretical basis and wide possibilities to develop the combination of FAM83H and NSCLC.

There are a few studies on the role of *FAM83* family members *C*, *E*, and *G*. The up-regulation of *FAM83C* and *FAM83E* was reported in bladder cancer specimens and ovarian cancer (Cipriano et al., 2014; Snijders et al., 2017). Our study disclosed that *FAM83C* was overexpressed in human NSCLC than normal tissues. What was more, higher mRNA expression of *FAM83C* was also significantly related with a shorter OS and FP of lung cancer patients, further analysis showed that the patients were categorized by cutoffs of 17 for *FAM83C* low expression and high expression and achieved a 0.971897/1 specificity/sensitivity in distinguishing poor survival from OS. Furthermore, the expression of *FAM83E* was increased in human LUAD than in normal tissues, while the expression of *FAM83E* was lower in LUSC tissues than in normal tissues. *FAM83C/E* expression were not related to tumor stage in patients with NSCLC and there was no obvious correlation in lung cancer between OS, PPS, and *FAM83E* expression. All in all, this finding was in contradiction with the role of *FAM83C* and *FAM83E* as an oncogene. Our study detected that the expression of *FAM83G* was up-regulated in human NSCLC, which was not related to the clinical characteristics of NSCLC patients and we could not find any literature as far as the role of *FAM83G* in cancer progression and data on prognostic factors in the Kaplan-Meier plotter.

There were some limitations in our study. On the one hand, all the data analyzed in our study were obtained from different online databases, which might cause background heterogeneity, thus further studies of larger sample sizes are required to confirm our findings. On the other hand, the study did not conduct experiments to verify the results obtained from bioinformatics analysis. Subsequently, further *in vitro* and *in vivo* studies should be performed to affirm our results, and might provide some desirable results to us.

## Conclusion

In this study, we formulated the expression and prognostic value of *FAM83* family in NSCLC. Besides, we also provided a thorough understanding of the heterogeneity and complexity of the molecular biology of NSCLC, which put forward a new direction for the diagnosis and treatment of NSCLC. Additionally, our results also revealed that overexpression of *FAM83A/B/D/F/H* was memorably related to clinical cancer stages in NSCLC patients. Besides, higher mRNA expressions of *FAM83A/B/C/D/F/H* were found to be notably associated with OS in lung cancer patients, furthermore, *FAM83A*, *FAM83C*, and *FAM83H* in OS group achieved a 0.9475/1, 0.971897/1, and 0.9454545/0.8974359 specificity/sensitivity in distinguishing short survivors from long survivors, respectively. Moreover, a high mutation rate of *FAM83* family (51%) was also discovered in LUAD patients, and genetic alteration in *FAM83* family was associated with a shorter OS and DFS in LUAD patients. Our results indicated that *FAM83A/H* might play an important role in NSCLC oncogenesis and might be a risk factor for survivals of NSCLC patients. To sum up, it is a charming hypothesis that targeting the *FAM83A/H* might be a correlatedly unexplored field in the future repertoire of tumor oncogenesis and provide a promising potential in tumor.

## Data Availability Statement

The datasets presented in this study can be found in online repositories. The names of the repository/repositories and accession number(s) can be found in the article/Supplementary Material.

## Author Contributions

JG and QM developed the idea, designed the research, and performed the data analysis work. JG drafted the manuscript. YL and QM reviewed the manuscript. All authors read and approved the final manuscript.

## Conflict of Interest

The authors declare that the research was conducted in the absence of any commercial or financial relationships that could be construed as a potential conflict of interest.

## Footnotes

1 http://www.oncomine.org

2 http://ualcan.path.uab.edu/

3 http://gepia.cancer-pku.cn/

4 http://kmplot.com/analysis/

5 http://www.cbioportal.org

6 http://www.string-db.org

7 https://david.ncifcrf.gov/summary.jsp

## Supplementary Material

Supplementary Figure 1 Q–Q graph and histogram were used to detect whether the sample data obeyed normal distribution. In Q–Q graph, *X*-axis is the normal distribution quantile and the *Y*-axis is the sample quantile (**A–K** left). In the histogram, the *X*-axis is the equidistance segmentation range of gene expression value, the *Y*-axis is the sample proportion located in this region, and the red curve is the density fitting curve, and the purple curve is the normal distribution fitting curve (**A–K** right).

Supplementary Figure 2 The scatter diagram is used to describe the relationship between *p*-value and cutoff. The *X*-axis represents the cutoff, the *Y*-axis represents the *p*-value, and the position shown in the arrow is the cutoff value corresponding to the *p* < 0.05.

## Abbreviations

NSCLC: non-small-cell lung cancer
LUAD: lung adenocarcinoma
LUSC: lung squamous cell carcinoma
TCGA: The Cancer Genome Atlas
GO: Gene Ontology
KEGG: Kyoto Encyclopedia of Genes and Genomes
BP: biological processes
CC: cellular components
MF: molecular functions
OS: over survival
DFS: disease free survival.
