# Corrigendum: Systematic Analysis of Expression Profiles and Prognostic Significance for FAM83 Family in Non-Small-Cell Lung Cancer

**DOI:** 10.3389/fmolb.2021.653454

**Published:** 2021-04-13

**Authors:** Junqing Gan, Qingwei Meng, Yanjing Li

**Affiliations:** ^^1^^ Department of Medical Oncology, Harbin Medical University Cancer Hospital, Harbin, China; ^^2^^ Department of Gastrointestinal Oncology, Harbin Medical University Cancer Hospital, Harbin, China

**Keywords:** non–small-cell lung cancer, FAM83 family, bioinformatics analysis, biomarker, prognostic value

In the published article, there were errors in affiliations 1 and 2 and the order of corresponding authors. Instead of “Junqing Gan^1^, Yanjing Li^1,^* and Qingwei Meng^2,^* ^1^Department of Gastrointestinal Oncology, Harbin Medical University Cancer Hospital, Harbin, China ^2^Department of Medical Oncology, Harbin Medical University Cancer Hospital, Harbin, China,” it should be “Junqing Gan^1^, Qingwei Meng^1,^* and Yanjing Li^2,^* ^1^Department of Medical Oncology, Harbin Medical University Cancer Hospital, Harbin, China ^2^Department of Gastrointestinal Oncology, Harbin Medical University Cancer Hospital, Harbin, China.”

The authors apologize for these errors and state that this does not change the scientific conclusions of the article in any way. The original article has been updated.

