# Supplementary figures and images for: Systematic Analysis of Expression Profiles and Prognostic Significance for FAM83 Family in Non-small-Cell Lung Cancer
Source: Front Mol Biosci. 2020 Dec 10;7:572406. doi: 10.3389/fmolb.2020.572406 (PMC7758490; doi:10.3389/fmolb.2020.572406)

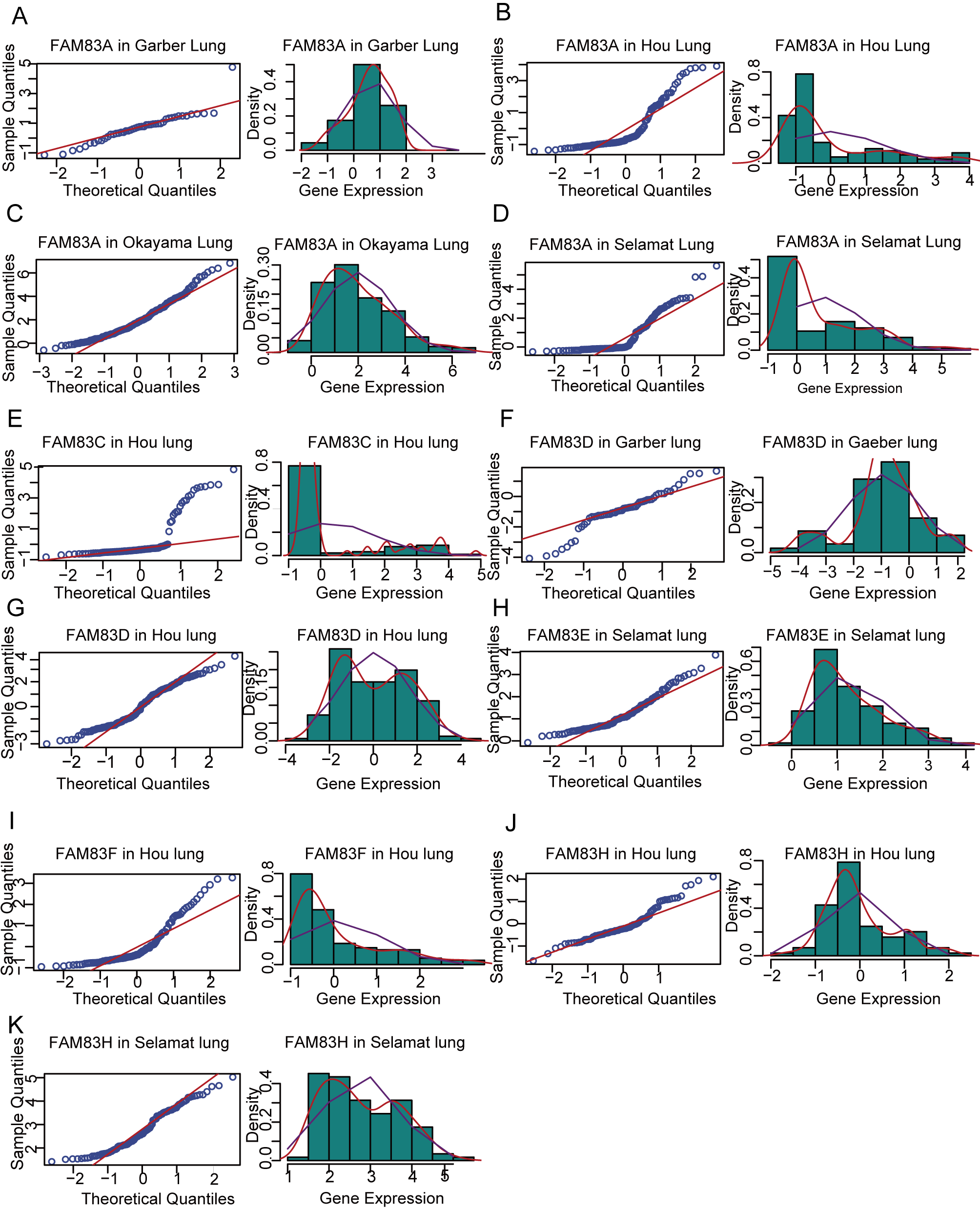

Supplement: Supplementary Figure 1 — Q–Q graph and histogram were used to detect whether the sample data obeyed normal distribution. In Q–Q graph, X-axis is the normal distribution quantile and the Y-axis is the sample quantile (A–K left). In the histogram, the X-axis is the equidistance segmentation range of gene expression value, the Y-axis is the sample proportion located in this region, and the red curve is the density fitting curve, and the purple curve is the normal distribution fitting curve (A–K right). [file Image_1.TIF]

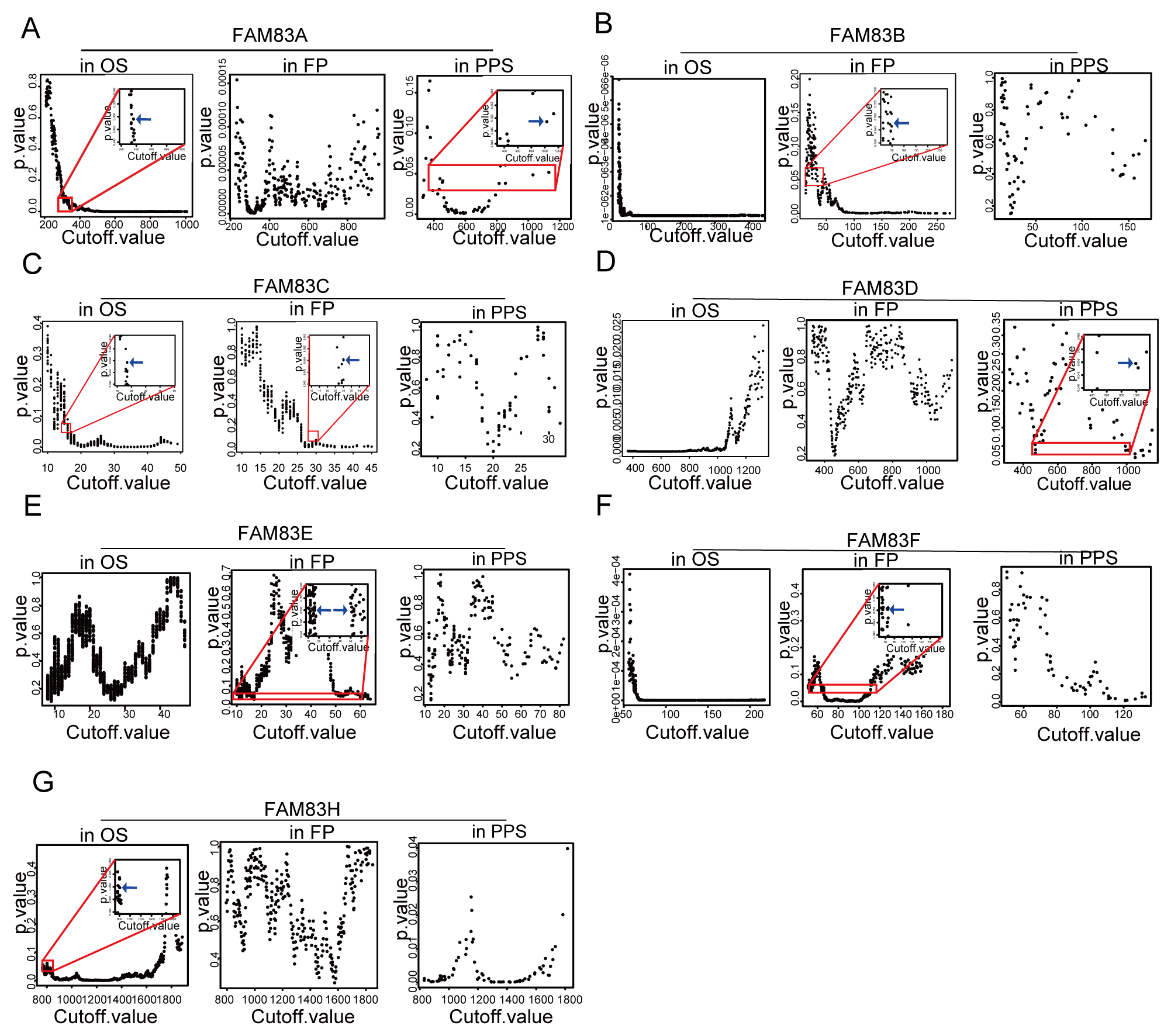

Supplement: Supplementary Figure 2 — The scatter diagram is used to describe the relationship between p-value and cutoff. The X-axis represents the cutoff, the Y-axis represents the p-value, and the position shown in the arrow is the cutoff value corresponding to the p < 0.05. [file Image_2.TIF]
