# Supplementary material for: Systematic Analysis of Expression Profiles and Prognostic Significance for FAM83 Family in Non-small-Cell Lung Cancer
Source: Front Mol Biosci. 2020 Dec 10;7:572406. doi: 10.3389/fmolb.2020.572406 (PMC7758490; doi:10.3389/fmolb.2020.572406)
Supplement: Supplementary file 3 [file Table_1.DOCX]

**Table 1. The expression level of YTHDF2 in public LIHC datasets**

| **Dataset** | **P-value** | **Type** | **Nums** | **Mean** | **SD** |
| --- | --- | --- | --- | --- | --- |
| TCGA+  GTEx | 4.671e−09 | tumor | 374 | 1766.39 | 448.72 |
|  |  | normal | 160 | 1539.58 | 412.6 |
| GSE14520-GPL3921 | 1.863e-14 | tumor | 225 | 7.52 | 0.55 |
|  |  | normal | 220 | 7.16 | 0.50 |
| GSE63898-GPL13667 | 3.366e-05 | tumor | 228 | 8.64 | 0.423 |
|  |  | normal | 168 | 8.48 | 0.356 |
| GSE64041-GPL6244 | 4.16-04 | tumor | 60 | 8.48 | 0.16 |
|  |  | normal | 60 | 8.35 | 0.16 |
| GSE14520-GPL571 | 0.005 | tumor | 22 | 7.58 | 0.61 |
|  |  | normal | 21 | 7.24 | 0.28 |

**SD：Standard Deviation**
