# Supplementary material for: Systematic Analysis of Expression Profiles and Prognostic Significance for FAM83 Family in Non-small-Cell Lung Cancer
Source: Front Mol Biosci. 2020 Dec 10;7:572406. doi: 10.3389/fmolb.2020.572406 (PMC7758490; doi:10.3389/fmolb.2020.572406)
Supplement: Supplementary file 4 [file Table_2.DOCX]

**Supplementary Table 2. The cutoff and p values of FAM83 family members in lung cancer patients (Kaplan-Meier plotter).**

| FAM83 family | OS | | FAM83 family | FP | | FAM83 family | PPS | |
| --- | --- | --- | --- | --- | --- | --- | --- | --- |
|  | cutoff | p value |  | cutoff | p value |  | cutoff | p value |
| FAM83A | 209 | 0.761294932220244 | FAM83A | 227 | 3.39981790522442e-05 | FAM83A | 331 | 0.0210179880353797 |
| FAM83A | 209 | 0.706183162622426 | FAM83A | 227 | 5.61076183834103e-05 | FAM83A | 335 | 0.0233664908100594 |
| FAM83A | 209 | 0.68241246019776 | FAM83A | 228 | 8.83526195497917e-05 | FAM83A | 337 | 0.0420049939477677 |
| FAM83A | 209 | 0.738591229037017 | FAM83A | 229 | 6.915285871158e-05 | FAM83A | 350 | 0.0689666434509628 |
| FAM83A | 210 | 0.674090718345498 | FAM83A | 231 | 0.000104463625969937 | FAM83A | 358 | 0.107784197980883 |
| FAM83A | 210 | 0.732663656097463 | FAM83A | 233 | 0.00015403929640362 | FAM83A | 359 | 0.106938268172816 |
| FAM83A | 211 | 0.695426516798708 | FAM83A | 236 | 0.000122410533977045 | FAM83A | 368 | 0.152946192405017 |
| FAM83A | 211 | 0.717446054377035 | FAM83A | 238 | 9.2684181434677e-05 | FAM83A | 375 | 0.164298912779058 |
| FAM83A | 212 | 0.760752756428808 | FAM83A | 240 | 6.93459180362448e-05 | FAM83A | 378 | 0.0946522141980555 |
| FAM83A | 213 | 0.715115292408769 | FAM83A | 241 | 5.53976658264542e-05 | FAM83A | 387 | 0.0668014174158655 |
| FAM83A | 213 | 0.78492252773905 | FAM83A | 241 | 4.05221821519647e-05 | FAM83A | 393 | 0.0294439930678118 |
| FAM83A | 213 | 0.834771488060147 | FAM83A | 242 | 3.08635928275194e-05 | FAM83A | 425 | 0.0250798998536905 |
| FAM83A | 215 | 0.781558418456816 | FAM83A | 242 | 2.93612270823519e-05 | FAM83A | 434 | 0.0306902628006548 |
| FAM83A | 216 | 0.835330648334491 | FAM83A | 242 | 2.20538301501701e-05 | FAM83A | 439 | 0.0438038368027536 |
| FAM83A | 217 | 0.842758178079738 | FAM83A | 245 | 1.84592007835475e-05 | FAM83A | 454 | 0.0394067728287771 |
| FAM83A | 217 | 0.798077696334634 | FAM83A | 247 | 1.38442424043419e-05 | FAM83A | 457 | 0.0411180583922465 |
| FAM83A | 218 | 0.74960445047409 | FAM83A | 247 | 2.66273363580503e-05 | FAM83A | 464 | 0.0204536494201324 |
| FAM83A | 219 | 0.723695405445559 | FAM83A | 248 | 4.83967655604859e-05 | FAM83A | 468 | 0.00988064953037215 |
| FAM83A | 220 | 0.683083716919495 | FAM83A | 249 | 8.70852880886848e-05 | FAM83A | 474 | 0.00928428846457272 |
| FAM83A | 221 | 0.739390798451668 | FAM83A | 250 | 6.41304609911056e-05 | FAM83A | 479 | 0.0141757675187083 |
| FAM83A | 224 | 0.758019381115453 | FAM83A | 250 | 4.99377337258113e-05 | FAM83A | 485 | 0.00592199975958146 |
| FAM83A | 225 | 0.799863422418316 | FAM83A | 250 | 3.79040390789004e-05 | FAM83A | 492 | 0.00341808899277408 |
| FAM83A | 225 | 0.766633603421877 | FAM83A | 251 | 6.37816199654027e-05 | FAM83A | 492 | 0.00559652771093877 |
| FAM83A | 229 | 0.727126043770342 | FAM83A | 252 | 0.000108057331647626 | FAM83A | 513 | 0.0063855577766379 |
| FAM83A | 229 | 0.769154286003468 | FAM83A | 254 | 8.28377445037839e-05 | FAM83A | 516 | 0.00505285208842228 |
| FAM83A | 229 | 0.826038737859811 | FAM83A | 260 | 6.305186653462e-05 | FAM83A | 530 | 0.00370366908303978 |
| FAM83A | 231 | 0.792249811309368 | FAM83A | 261 | 0.000105646592687465 | FAM83A | 533 | 0.00209549612419946 |
| FAM83A | 232 | 0.821849563913966 | FAM83A | 263 | 8.09686195741488e-05 | FAM83A | 534 | 0.00285557298762315 |
| FAM83A | 235 | 0.758260913246708 | FAM83A | 264 | 6.94982065551467e-05 | FAM83A | 536 | 0.00163310943561724 |
| FAM83A | 236 | 0.659108414219609 | FAM83A | 265 | 5.40451688025921e-05 | FAM83A | 540 | 0.003229585284554 |
| FAM83A | 237 | 0.616548180500404 | FAM83A | 268 | 4.10652108311814e-05 | FAM83A | 541 | 0.00528904667646878 |
| FAM83A | 238 | 0.580804837959576 | FAM83A | 272 | 7.62266497055478e-05 | FAM83A | 547 | 0.00233458137016867 |
| FAM83A | 240 | 0.535671609277303 | FAM83A | 274 | 5.8172095870273e-05 | FAM83A | 569 | 0.00152347580237795 |
| FAM83A | 240 | 0.582481217505323 | FAM83A | 275 | 4.70228779435911e-05 | FAM83A | 586 | 0.00214175429790013 |
| FAM83A | 241 | 0.552811923552956 | FAM83A | 278 | 4.33440094109868e-05 | FAM83A | 590 | 0.000862712137788113 |
| FAM83A | 241 | 0.566223814986187 | FAM83A | 279 | 3.2580436858136e-05 | FAM83A | 601 | 0.00182729440051516 |
| FAM83A | 241 | 0.516875268056854 | FAM83A | 279 | 2.52370847423806e-05 | FAM83A | 652 | 0.00374665199197605 |
| FAM83A | 242 | 0.48349808350109 | FAM83A | 279 | 2.02707551120124e-05 | FAM83A | 660 | 0.00590067706104091 |
| FAM83A | 242 | 0.477854446083743 | FAM83A | 280 | 1.50882060365051e-05 | FAM83A | 677 | 0.00509921844421616 |
| FAM83A | 242 | 0.444966932922187 | FAM83A | 284 | 1.11214730390476e-05 | FAM83A | 689 | 0.00833552540806242 |
| FAM83A | 244 | 0.427549891195998 | FAM83A | 284 | 1.98704391459259e-05 | FAM83A | 705 | 0.00306385162762722 |
| FAM83A | 245 | 0.473395527693452 | FAM83A | 285 | 1.36589426418369e-05 | FAM83A | 712 | 0.00536705792565624 |
| FAM83A | 245 | 0.441260871565797 | FAM83A | 285 | 1.22335598179333e-05 | FAM83A | 722 | 0.0075486125060184 |
| FAM83A | 246 | 0.367811452348772 | FAM83A | 286 | 9.34777163012946e-06 | FAM83A | 745 | 0.00964901530401643 |
| FAM83A | 247 | 0.400576946588635 | FAM83A | 286 | 6.66879727388391e-06 | FAM83A | 751 | 0.0165097557765852 |
| FAM83A | 249 | 0.450528593534107 | FAM83A | 287 | 5.08795530157478e-06 | FAM83A | 792 | 0.0238209455435838 |
| FAM83A | 249 | 0.484964779191747 | FAM83A | 287 | 4.03374779994774e-06 | FAM83A | 815 | 0.0381636530429774 |
| FAM83A | 249 | 0.442314629250535 | FAM83A | 290 | 6.37971776850911e-06 | FAM83A | 823 | 0.0595811625382995 |
| FAM83A | 250 | 0.485134989831313 | FAM83A | 292 | 4.81076432743062e-06 | FAM83A | 854 | 0.0608018940350743 |
| FAM83A | 250 | 0.529088487449162 | FAM83A | 293 | 3.49312057170744e-06 | FAM83A | 855 | 0.0382803013483413 |
| FAM83A | 250 | 0.582387381927073 | FAM83A | 293 | 2.52587542328262e-06 | FAM83A | 883 | 0.0639546953315567 |
| FAM83A | 250 | 0.546490715771832 | FAM83A | 293 | 4.44715059517939e-06 | FAM83A | 886 | 0.0872388322086488 |
| FAM83A | 251 | 0.516264756282186 | FAM83A | 293 | 8.37445311361504e-06 | FAM83A | 908 | 0.127338107627979 |
| FAM83A | 251 | 0.546596788454976 | FAM83A | 295 | 6.98690607503823e-06 | FAM83A | 921 | 0.126029683999012 |
| FAM83A | 251 | 0.495081386642315 | FAM83A | 297 | 5.48599243526799e-06 | FAM83A | 926 | 0.113012255263796 |
| FAM83A | 252 | 0.466914999472749 | FAM83A | 297 | 4.04782591140951e-06 | FAM83A | 939 | 0.109684759146393 |
| FAM83A | 252 | 0.52164146506386 | FAM83A | 297 | 2.97536094244596e-06 | FAM83A | 960 | 0.115409588975399 |
| FAM83A | 252 | 0.488052683472921 | FAM83A | 298 | 2.33129592903863e-06 | FAM83A | 970 | 0.104412532872578 |
| FAM83A | 253 | 0.532278857122053 | FAM83A | 299 | 1.84039502520687e-06 | FAM83A | 995 | 0.133437139719034 |
| FAM83A | 253 | 0.515264982397049 | FAM83A | 299 | 1.64618352739023e-06 | FAM83A | 1025 | 0.132504243959803 |
| FAM83A | 254 | 0.535788437877543 | FAM83A | 301 | 1.1970166689347e-06 | FAM83A | 1028 | 0.106675934873019 |
| FAM83A | 256 | 0.501245461399991 | FAM83A | 301 | 2.04808989733259e-06 | FAM83A | 1030 | 0.0486022340169211 |
| FAM83A | 257 | 0.467121824087745 | FAM83A | 301 | 1.46935246542721e-06 | FAM83A | 1040 | 0.0775062208286049 |
| FAM83A | 257 | 0.52337999618666 | FAM83A | 302 | 1.06635604574704e-06 | FAM83A | 1087 | 0.0987791287360322 |
| FAM83A | 260 | 0.484874245511929 | FAM83A | 304 | 7.98629327337745e-07 | FAM83A | 1088 | 0.128339843326191 |
| FAM83A | 261 | 0.452676696136266 | FAM83A | 306 | 1.46478066969642e-06 | FAM83A | 1129 | 0.0639189262963732 |
| FAM83A | 261 | 0.433242131864079 | FAM83A | 306 | 1.41035812875757e-06 | FAM83A | 1131 | 0.0516802346439181 |
| FAM83A | 263 | 0.403161554124859 | FAM83A | 306 | 2.10792325591292e-06 | FAM83A | 1182 | 0.0876628792895793 |
| FAM83A | 264 | 0.388407266317143 | FAM83A | 306 | 3.65967046763927e-06 | FAM83A | 1226 | 0.0817115980486304 |
| FAM83A | 265 | 0.362015949916858 | FAM83A | 307 | 2.64128295881906e-06 | FAM83A | 1231 | 0.0810039200752395 |
| FAM83A | 265 | 0.354823109982049 | FAM83A | 308 | 2.00975693964035e-06 | FAM83A | 1235 | 0.099145686120379 |
| FAM83A | 266 | 0.328172578690173 | FAM83A | 308 | 1.44847325264672e-06 | FAM83B | 12 | 0.610844602108638 |
| FAM83A | 268 | 0.350823235854073 | FAM83A | 309 | 1.04913683887518e-06 | FAM83B | 13 | 0.714085314628147 |
| FAM83A | 268 | 0.354035623240397 | FAM83A | 311 | 1.76485763288431e-06 | FAM83B | 13 | 0.876321958916408 |
| FAM83A | 270 | 0.389863162973463 | FAM83A | 314 | 1.27510474551106e-06 | FAM83B | 13 | 0.953970581383181 |
| FAM83A | 270 | 0.323415561292985 | FAM83A | 314 | 1.00683178641085e-06 | FAM83B | 14 | 0.994946604568042 |
| FAM83A | 270 | 0.279126258833819 | FAM83A | 318 | 7.5185876392782e-07 | FAM83B | 14 | 0.849408058588102 |
| FAM83A | 272 | 0.235433153653568 | FAM83A | 318 | 5.31102285278722e-07 | FAM83B | 14 | 0.968990619901257 |
| FAM83A | 272 | 0.215724058164695 | FAM83A | 319 | 4.09316327485561e-07 | FAM83B | 15 | 0.891967788990766 |
| FAM83A | 272 | 0.206051071424888 | FAM83A | 320 | 8.17500710523716e-07 | FAM83B | 16 | 0.813163568185763 |
| FAM83A | 272 | 0.239155820458912 | FAM83A | 320 | 5.97717643273343e-07 | FAM83B | 16 | 0.708024824576418 |
| FAM83A | 274 | 0.250897153846578 | FAM83A | 321 | 4.1649467455678e-07 | FAM83B | 16 | 0.588625475795474 |
| FAM83A | 275 | 0.235750704168357 | FAM83A | 323 | 3.55187155219445e-07 | FAM83B | 16 | 0.869724350418729 |
| FAM83A | 277 | 0.267805489368778 | FAM83A | 323 | 5.25120244721579e-07 | FAM83B | 17 | 0.921158144203183 |
| FAM83A | 278 | 0.285705584265373 | FAM83A | 324 | 1.04040036867955e-06 | FAM83B | 17 | 0.974238215780308 |
| FAM83A | 278 | 0.271678836957198 | FAM83A | 324 | 2.05467213962327e-06 | FAM83B | 19 | 0.810766833101938 |
| FAM83A | 279 | 0.248660974306763 | FAM83A | 325 | 1.3520786669454e-06 | FAM83B | 19 | 0.661163093184917 |
| FAM83A | 279 | 0.265352085780045 | FAM83A | 326 | 1.01842944661557e-06 | FAM83B | 19 | 0.525565121057573 |
| FAM83A | 279 | 0.298555450417791 | FAM83A | 328 | 1.67417627388584e-06 | FAM83B | 19 | 0.428351001866674 |
| FAM83A | 280 | 0.274439892986311 | FAM83A | 328 | 3.14567236800036e-06 | FAM83B | 20 | 0.636012875820008 |
| FAM83A | 281 | 0.249421795767221 | FAM83A | 330 | 5.72203528326423e-06 | FAM83B | 20 | 0.514170109259804 |
| FAM83A | 281 | 0.286755607940034 | FAM83A | 330 | 4.48998873685659e-06 | FAM83B | 21 | 0.465119364991525 |
| FAM83A | 281 | 0.310609881798915 | FAM83A | 331 | 3.42241229947168e-06 | FAM83B | 22 | 0.341365487399945 |
| FAM83A | 283 | 0.291325942882306 | FAM83A | 331 | 6.3377047176224e-06 | FAM83B | 22 | 0.256893467779983 |
| FAM83A | 284 | 0.277986641306626 | FAM83A | 333 | 4.63458935846407e-06 | FAM83B | 23 | 0.205608094128625 |
| FAM83A | 285 | 0.245113351701294 | FAM83A | 334 | 8.12398625547963e-06 | FAM83B | 23 | 0.152198872767503 |
| FAM83A | 285 | 0.238371480781287 | FAM83A | 335 | 5.73931892739431e-06 | FAM83B | 24 | 0.156248188185817 |
| FAM83A | 286 | 0.261575372401978 | FAM83A | 335 | 9.26561576427349e-06 | FAM83B | 25 | 0.270797413746888 |
| FAM83A | 286 | 0.231946996801228 | FAM83A | 337 | 7.11201888881806e-06 | FAM83B | 25 | 0.269656960145913 |
| FAM83A | 286 | 0.254081928440308 | FAM83A | 337 | 9.84400600349489e-06 | FAM83B | 25 | 0.188442431693445 |
| FAM83A | 287 | 0.23572159487878 | FAM83A | 337 | 1.80808066790783e-05 | FAM83B | 27 | 0.222146846063311 |
| FAM83A | 287 | 0.2601088840645 | FAM83A | 337 | 1.34872499218501e-05 | FAM83B | 27 | 0.351674585371845 |
| FAM83A | 287 | 0.237328617546698 | FAM83A | 341 | 9.83686107883451e-06 | FAM83B | 27 | 0.509414094306707 |
| FAM83A | 289 | 0.257164691959589 | FAM83A | 342 | 7.41315558494476e-06 | FAM83B | 28 | 0.484274361901819 |
| FAM83A | 290 | 0.232923653455297 | FAM83A | 349 | 5.7443818152812e-06 | FAM83B | 29 | 0.493381594340847 |
| FAM83A | 291 | 0.214432997923983 | FAM83A | 350 | 4.49516342031796e-06 | FAM83B | 31 | 0.378799052515672 |
| FAM83A | 292 | 0.23167942743376 | FAM83A | 350 | 7.45953232533394e-06 | FAM83B | 31 | 0.305888462542284 |
| FAM83A | 292 | 0.209720713746668 | FAM83A | 353 | 5.24531911248728e-06 | FAM83B | 35 | 0.469213899532838 |
| FAM83A | 293 | 0.205073831522317 | FAM83A | 354 | 5.05346292819096e-06 | FAM83B | 37 | 0.351295183591358 |
| FAM83A | 293 | 0.185050315825314 | FAM83A | 355 | 3.52957075463612e-06 | FAM83B | 39 | 0.556069377253104 |
| FAM83A | 293 | 0.206903043176279 | FAM83A | 358 | 5.39720103842062e-06 | FAM83B | 39 | 0.558199407439784 |
| FAM83A | 295 | 0.197141810833478 | FAM83A | 359 | 9.1914269507302e-06 | FAM83B | 40 | 0.621165312716479 |
| FAM83A | 295 | 0.166021831322969 | FAM83A | 363 | 1.53771391660433e-05 | FAM83B | 41 | 0.819496624321122 |
| FAM83A | 297 | 0.154114102042107 | FAM83A | 364 | 1.13738325381384e-05 | FAM83B | 42 | 0.774216082875133 |
| FAM83A | 297 | 0.139947256374008 | FAM83A | 367 | 8.40397625362133e-06 | FAM83B | 45 | 0.93523192861859 |
| FAM83A | 297 | 0.126817713845992 | FAM83A | 367 | 1.39508902075209e-05 | FAM83B | 45 | 0.955988911330201 |
| FAM83A | 298 | 0.117571955141638 | FAM83A | 368 | 9.37990219057904e-06 | FAM83B | 53 | 0.837859212414582 |
| FAM83A | 298 | 0.13698718541074 | FAM83A | 370 | 1.51917416056191e-05 | FAM83B | 55 | 0.844246665743779 |
| FAM83A | 299 | 0.127947099424746 | FAM83A | 375 | 1.11209375544434e-05 | FAM83B | 55 | 0.850644166484147 |
| FAM83A | 299 | 0.113589400312579 | FAM83A | 378 | 1.65797335223763e-05 | FAM83B | 56 | 0.688839608362593 |
| FAM83A | 300 | 0.10261892221541 | FAM83A | 378 | 2.38877460345077e-05 | FAM83B | 61 | 0.905279392548809 |
| FAM83A | 300 | 0.116092719590532 | FAM83A | 379 | 3.72890202128932e-05 | FAM83B | 62 | 0.907822187715882 |
| FAM83A | 300 | 0.133331585781892 | FAM83A | 380 | 2.72230075923748e-05 | FAM83B | 78 | 0.774229482796926 |
| FAM83A | 301 | 0.155778222868207 | FAM83A | 385 | 4.85890468352036e-05 | FAM83B | 79 | 0.750109376760964 |
| FAM83A | 301 | 0.136710776009522 | FAM83A | 386 | 3.50039140735302e-05 | FAM83B | 84 | 0.753790189474016 |
| FAM83A | 301 | 0.151820120808126 | FAM83A | 387 | 2.54990311433778e-05 | FAM83B | 85 | 0.649243627149532 |
| FAM83A | 301 | 0.136491698349149 | FAM83A | 392 | 3.58917045565489e-05 | FAM83B | 86 | 0.878953481057958 |
| FAM83A | 302 | 0.123767443786733 | FAM83A | 393 | 2.69312515589804e-05 | FAM83B | 89 | 0.915838205907814 |
| FAM83A | 303 | 0.113385954129995 | FAM83A | 396 | 2.05874732841707e-05 | FAM83B | 96 | 0.98330218207085 |
| FAM83A | 303 | 0.124926260110755 | FAM83A | 397 | 3.60350801454721e-05 | FAM83B | 104 | 0.704019546237743 |
| FAM83A | 305 | 0.110214639375668 | FAM83A | 399 | 5.60532211990803e-05 | FAM83B | 114 | 0.758067555909745 |
| FAM83A | 306 | 0.119171518496465 | FAM83A | 400 | 8.49054291061583e-05 | FAM83B | 133 | 0.528054886565419 |
| FAM83A | 306 | 0.117900643953725 | FAM83A | 401 | 6.15886149683225e-05 | FAM83B | 133 | 0.676396940991215 |
| FAM83A | 306 | 0.102989135477794 | FAM83A | 402 | 4.60256491857328e-05 | FAM83B | 133 | 0.424043390288561 |
| FAM83A | 306 | 0.114290822397937 | FAM83A | 402 | 7.23876448366126e-05 | FAM83B | 141 | 0.387839097801412 |
| FAM83A | 308 | 0.10232664701524 | FAM83A | 405 | 0.000113027404947615 | FAM83B | 142 | 0.39460896121701 |
| FAM83A | 308 | 0.0919669491518369 | FAM83A | 409 | 9.87239723738715e-05 | FAM83B | 144 | 0.397325770801133 |
| FAM83A | 308 | 0.0741609443715069 | FAM83A | 411 | 7.62997768410595e-05 | FAM83B | 146 | 0.452105174349273 |
| FAM83A | 309 | 0.0663835290309994 | FAM83A | 412 | 0.000111886750203121 | FAM83B | 155 | 0.517108203540736 |
| FAM83A | 309 | 0.0660521043340465 | FAM83A | 415 | 8.3448213233711e-05 | FAM83B | 156 | 0.37339959019211 |
| FAM83A | 309 | 0.0585900950586254 | FAM83A | 418 | 5.7465878269415e-05 | FAM83B | 161 | 0.524484339072148 |
| FAM83A | 310 | 0.0615753713580344 | FAM83A | 420 | 4.1233970191404e-05 | FAM83B | 168 | 0.607625576670947 |
| FAM83A | 311 | 0.0697138760633313 | FAM83A | 425 | 2.78702608590448e-05 | FAM83C | 8 | 0.656088285344147 |
| FAM83A | 311 | 0.0783818944235488 | FAM83A | 425 | 2.00776209045147e-05 | FAM83C | 9 | 0.409581721084374 |
| FAM83A | 313 | 0.0691807773028411 | FAM83A | 425 | 3.21395760797157e-05 | FAM83C | 10 | 0.533625640583579 |
| FAM83A | 313 | 0.0773241647336327 | FAM83A | 426 | 5.08224029917361e-05 | FAM83C | 10 | 0.544383679276421 |
| FAM83A | 314 | 0.069438302697535 | FAM83A | 428 | 4.04126863008162e-05 | FAM83C | 10 | 0.70796477463923 |
| FAM83A | 314 | 0.0778578283330705 | FAM83A | 428 | 2.92516362471909e-05 | FAM83C | 10 | 0.882254864915941 |
| FAM83A | 316 | 0.0650928248929883 | FAM83A | 429 | 2.1042955762992e-05 | FAM83C | 10 | 0.93945353682341 |
| FAM83A | 316 | 0.078517193329475 | FAM83A | 430 | 1.78416090366386e-05 | FAM83C | 10 | 0.942272068269578 |
| FAM83A | 316 | 0.0901684284804704 | FAM83A | 432 | 1.35338582621964e-05 | FAM83C | 10 | 0.878158783662732 |
| FAM83A | 318 | 0.0973508432663241 | FAM83A | 433 | 1.01428463630767e-05 | FAM83C | 11 | 0.675323616263952 |
| FAM83A | 319 | 0.0861369370843007 | FAM83A | 434 | 1.54924363488097e-05 | FAM83C | 11 | 0.72086386044812 |
| FAM83A | 319 | 0.078684045425143 | FAM83A | 439 | 2.73106766065995e-05 | FAM83C | 12 | 0.816189066923724 |
| FAM83A | 320 | 0.0929672002071588 | FAM83A | 446 | 3.86252456812097e-05 | FAM83C | 12 | 0.881964460179184 |
| FAM83A | 320 | 0.0965598103891972 | FAM83A | 448 | 2.96974125922663e-05 | FAM83C | 13 | 0.903522196004753 |
| FAM83A | 320 | 0.0873460584518061 | FAM83A | 449 | 2.36960537126675e-05 | FAM83C | 14 | 0.944612848180637 |
| FAM83A | 321 | 0.0765679276492151 | FAM83A | 451 | 1.77585829628366e-05 | FAM83C | 14 | 0.768531149688532 |
| FAM83A | 323 | 0.0881598643768832 | FAM83A | 452 | 1.27390770951674e-05 | FAM83C | 14 | 0.672273315654644 |
| FAM83A | 324 | 0.078928305102803 | FAM83A | 454 | 1.01888687896115e-05 | FAM83C | 14 | 0.912265673263486 |
| FAM83A | 324 | 0.0831153572502776 | FAM83A | 457 | 1.61922704473763e-05 | FAM83C | 15 | 0.884369942827159 |
| FAM83A | 326 | 0.0699326606195329 | FAM83A | 459 | 2.57262414279713e-05 | FAM83C | 16 | 0.810788821452192 |
| FAM83A | 329 | 0.0768177712593284 | FAM83A | 460 | 4.21639040847307e-05 | FAM83C | 16 | 0.967385957345285 |
| FAM83A | 330 | 0.058635414252729 | FAM83A | 461 | 3.03394664458772e-05 | FAM83C | 17 | 0.76931006330081 |
| FAM83A | 330 | 0.0652350859786972 | FAM83A | 464 | 2.45021282230775e-05 | FAM83C | 17 | 0.759002808992443 |
| FAM83A | 330 | 0.0599406294023976 | FAM83A | 465 | 3.77667229240876e-05 | FAM83C | 17 | 0.635913881971829 |
| FAM83A | 330 | 0.0545018977146635 | FAM83A | 468 | 2.99574726992108e-05 | FAM83C | 17 | 0.784806280025128 |
| FAM83A | 331 | 0.0471712388519147 | FAM83A | 468 | 5.13566094574593e-05 | FAM83C | 17 | 0.601262255320345 |
| FAM83A | 331 | 0.0535796073703527 | FAM83A | 472 | 4.23740760760839e-05 | FAM83C | 18 | 0.556457973831849 |
| FAM83A | 332 | 0.0476814819204787 | FAM83A | 474 | 3.23917682196029e-05 | FAM83C | 18 | 0.384217952291825 |
| FAM83A | 334 | 0.0543382304207112 | FAM83A | 476 | 5.41637335006792e-05 | FAM83C | 18 | 0.472139728679616 |
| FAM83A | 335 | 0.060053257765786 | FAM83A | 476 | 3.90278890241895e-05 | FAM83C | 18 | 0.399976627717442 |
| FAM83A | 337 | 0.0687921039342615 | FAM83A | 477 | 2.95394826520342e-05 | FAM83C | 19 | 0.234219794890459 |
| FAM83A | 337 | 0.0734248778704239 | FAM83A | 479 | 2.14768512198038e-05 | FAM83C | 19 | 0.30963040362841 |
| FAM83A | 337 | 0.0509173813998216 | FAM83A | 480 | 3.56811643600427e-05 | FAM83C | 20 | 0.251018373103944 |
| FAM83A | 337 | 0.0454076453869063 | FAM83A | 485 | 2.71394611159847e-05 | FAM83C | 20 | 0.185818043210185 |
| FAM83A | 339 | 0.0398464531429227 | FAM83A | 485 | 2.12855956391859e-05 | FAM83C | 21 | 0.230495022392914 |
| FAM83A | 339 | 0.0342195449499345 | FAM83A | 486 | 3.91062630507288e-05 | FAM83C | 21 | 0.289881151849112 |
| FAM83A | 340 | 0.0303325999427225 | FAM83A | 487 | 2.73965408254565e-05 | FAM83C | 21 | 0.355739088906459 |
| FAM83A | 340 | 0.0254491204611515 | FAM83A | 492 | 1.97417151436933e-05 | FAM83C | 21 | 0.34409890444292 |
| FAM83A | 341 | 0.0229639394891003 | FAM83A | 492 | 3.44093984778792e-05 | FAM83C | 21 | 0.434717021575958 |
| FAM83A | 342 | 0.0203417336499704 | FAM83A | 493 | 6.0274759059203e-05 | FAM83C | 21 | 0.324629149630491 |
| FAM83A | 347 | 0.0231054605406295 | FAM83A | 504 | 4.37007177133036e-05 | FAM83C | 22 | 0.425580143956872 |
| FAM83A | 347 | 0.0199089813873203 | FAM83A | 505 | 4.3563709091181e-05 | FAM83C | 22 | 0.410339064596622 |
| FAM83A | 349 | 0.0211831013968679 | FAM83A | 507 | 3.38803580506065e-05 | FAM83C | 22 | 0.451598724845878 |
| FAM83A | 350 | 0.0251123187880337 | FAM83A | 512 | 2.44436240372635e-05 | FAM83C | 22 | 0.349244605083511 |
| FAM83A | 350 | 0.0255392666685547 | FAM83A | 513 | 1.83623599236496e-05 | FAM83C | 22 | 0.444803847258719 |
| FAM83A | 350 | 0.0298482213548766 | FAM83A | 514 | 2.80088987961431e-05 | FAM83C | 22 | 0.413784684977634 |
| FAM83A | 350 | 0.0313099706218307 | FAM83A | 515 | 2.02890289638466e-05 | FAM83C | 23 | 0.502382547150196 |
| FAM83A | 353 | 0.032190724420486 | FAM83A | 516 | 1.43676258954015e-05 | FAM83C | 23 | 0.460090198802248 |
| FAM83A | 354 | 0.0318121672731642 | FAM83A | 517 | 1.93436250135999e-05 | FAM83C | 23 | 0.484240240710712 |
| FAM83A | 355 | 0.0332064666927699 | FAM83A | 527 | 1.43959299501211e-05 | FAM83C | 23 | 0.457012583036909 |
| FAM83A | 358 | 0.0379829749187359 | FAM83A | 530 | 1.00005459716899e-05 | FAM83C | 23 | 0.332334810272443 |
| FAM83A | 359 | 0.0422914112478375 | FAM83A | 533 | 1.54787507463631e-05 | FAM83C | 24 | 0.420535432619382 |
| FAM83A | 362 | 0.049098356877995 | FAM83A | 533 | 1.10482572920577e-05 | FAM83C | 24 | 0.541795660200394 |
| FAM83A | 363 | 0.0489890430269463 | FAM83A | 533 | 1.87055774458485e-05 | FAM83C | 24 | 0.692875964117721 |
| FAM83A | 364 | 0.0435670958592188 | FAM83A | 534 | 1.32499059444908e-05 | FAM83C | 25 | 0.79529873341489 |
| FAM83A | 367 | 0.0387538826730001 | FAM83A | 535 | 1.99714927697785e-05 | FAM83C | 25 | 0.79136727044244 |
| FAM83A | 367 | 0.0448616172063147 | FAM83A | 536 | 1.28308525695956e-05 | FAM83C | 26 | 0.805769474068151 |
| FAM83A | 368 | 0.036941208894887 | FAM83A | 540 | 2.22850696020142e-05 | FAM83C | 28 | 0.979431100062518 |
| FAM83A | 368 | 0.0367422051555655 | FAM83A | 541 | 4.00398663383891e-05 | FAM83C | 28 | 0.940870532538147 |
| FAM83A | 370 | 0.040863337292538 | FAM83A | 542 | 5.40152113440571e-05 | FAM83C | 28 | 0.916363032179319 |
| FAM83A | 373 | 0.0360885366038159 | FAM83A | 542 | 4.01452804438625e-05 | FAM83C | 28 | 0.999172236107578 |
| FAM83A | 373 | 0.0357915546163279 | FAM83A | 543 | 5.92159798434094e-05 | FAM83C | 29 | 0.960115939795964 |
| FAM83A | 374 | 0.0434819885621154 | FAM83A | 543 | 4.29958622055586e-05 | FAM83C | 29 | 0.933532271653925 |
| FAM83A | 374 | 0.0381621804735508 | FAM83A | 547 | 3.12390184288105e-05 | FAM83C | 29 | 0.920887307273361 |
| FAM83A | 375 | 0.0408170776402103 | FAM83A | 547 | 2.53381446455361e-05 | FAM83C | 29 | 0.81425652489808 |
| FAM83A | 378 | 0.0352861197082826 | FAM83A | 550 | 3.32472438898901e-05 | FAM83C | 30 | 0.663359482868868 |
| FAM83A | 379 | 0.0311135281470927 | FAM83A | 554 | 2.3168814603246e-05 | FAM83C | 30 | 0.451204320024572 |
| FAM83A | 379 | 0.0330080624886123 | FAM83A | 569 | 1.63873040839354e-05 | FAM83C | 30 | 0.445299248406596 |
| FAM83A | 380 | 0.0284811116137048 | FAM83A | 573 | 2.44070573301213e-05 | FAM83C | 30 | 0.475078670999825 |
| FAM83A | 380 | 0.0243113463296598 | FAM83A | 576 | 1.71596666553665e-05 | FAM83C | 31 | 0.603240681017788 |
| FAM83A | 381 | 0.0199204075141437 | FAM83A | 577 | 1.70193523389972e-05 | FAM83C | 32 | 0.369799542776899 |
| FAM83A | 382 | 0.0169468382690022 | FAM83A | 581 | 1.28490302354268e-05 | FAM83D | 321 | 0.257894702351365 |
| FAM83A | 382 | 0.0133133062212072 | FAM83A | 586 | 9.10906470687606e-06 | FAM83D | 323 | 0.221759705246152 |
| FAM83A | 385 | 0.0152511890971261 | FAM83A | 590 | 1.50918659326524e-05 | FAM83D | 336 | 0.245620690971253 |
| FAM83A | 385 | 0.0126494679103376 | FAM83A | 594 | 2.78241202282671e-05 | FAM83D | 350 | 0.372353338602836 |
| FAM83A | 386 | 0.0157904802582919 | FAM83A | 601 | 2.02433944448515e-05 | FAM83D | 364 | 0.289742791832562 |
| FAM83A | 387 | 0.0133425049269223 | FAM83A | 609 | 2.72197027753146e-05 | FAM83D | 364 | 0.291594403255725 |
| FAM83A | 388 | 0.0103405271058259 | FAM83A | 626 | 1.94088514407495e-05 | FAM83D | 368 | 0.21781477595054 |
| FAM83A | 389 | 0.0117608422306863 | FAM83A | 628 | 3.44096456469582e-05 | FAM83D | 380 | 0.139004350732334 |
| FAM83A | 392 | 0.0141648408374126 | FAM83A | 629 | 2.61265333561358e-05 | FAM83D | 393 | 0.164911386558207 |
| FAM83A | 392 | 0.0158208404805546 | FAM83A | 631 | 1.84295945971027e-05 | FAM83D | 403 | 0.312554152388631 |
| FAM83A | 393 | 0.0138493260998591 | FAM83A | 632 | 3.30542496067693e-05 | FAM83D | 416 | 0.247263921665211 |
| FAM83A | 394 | 0.0121401427681354 | FAM83A | 636 | 2.52128287388149e-05 | FAM83D | 422 | 0.225948001362553 |
| FAM83A | 394 | 0.0145081324066322 | FAM83A | 643 | 2.34393676448176e-05 | FAM83D | 441 | 0.146457815282448 |
| FAM83A | 395 | 0.0123126463510276 | FAM83A | 650 | 1.56642548770928e-05 | FAM83D | 443 | 0.162459015441547 |
| FAM83A | 396 | 0.0154359622700513 | FAM83A | 651 | 1.1271923907585e-05 | FAM83D | 450 | 0.125197737671351 |
| FAM83A | 397 | 0.0193171334004014 | FAM83A | 652 | 9.04473354442414e-06 | FAM83D | 455 | 0.147389166711648 |
| FAM83A | 399 | 0.0206744624931306 | FAM83A | 655 | 1.64189001491067e-05 | FAM83D | 459 | 0.101987037554233 |
| FAM83A | 400 | 0.0229599760055195 | FAM83A | 660 | 2.61696096281291e-05 | FAM83D | 464 | 0.0750094988961794 |
| FAM83A | 400 | 0.0274757438157344 | FAM83A | 660 | 2.14774468020097e-05 | FAM83D | 464 | 0.0786691328889344 |
| FAM83A | 400 | 0.0321458094409448 | FAM83A | 662 | 1.59274153961562e-05 | FAM83D | 468 | 0.0538037200869891 |
| FAM83A | 401 | 0.026845747065352 | FAM83A | 672 | 1.08419686957873e-05 | FAM83D | 470 | 0.0303222109239625 |
| FAM83A | 401 | 0.0302649092908169 | FAM83A | 672 | 7.83004558585936e-06 | FAM83D | 471 | 0.0396708581465056 |
| FAM83A | 401 | 0.0344483523812954 | FAM83A | 673 | 5.24088398973175e-06 | FAM83D | 477 | 0.0779631389466484 |
| FAM83A | 403 | 0.0304232085032233 | FAM83A | 677 | 7.40206393007695e-06 | FAM83D | 484 | 0.0913474170720978 |
| FAM83A | 405 | 0.024242901442042 | FAM83A | 682 | 1.37084546303663e-05 | FAM83D | 495 | 0.0603789079077214 |
| FAM83A | 410 | 0.0229733497190474 | FAM83A | 684 | 1.08585664022014e-05 | FAM83D | 505 | 0.120159675198431 |
| FAM83A | 412 | 0.0276208785343887 | FAM83A | 686 | 8.36155218959169e-06 | FAM83D | 506 | 0.0808307666489225 |
| FAM83A | 412 | 0.0201566965348877 | FAM83A | 689 | 1.38418457438512e-05 | FAM83D | 514 | 0.0645942300600748 |
| FAM83A | 413 | 0.0173473534053432 | FAM83A | 690 | 2.04307522926064e-05 | FAM83D | 521 | 0.0911626505032702 |
| FAM83A | 414 | 0.0152631462917455 | FAM83A | 690 | 3.52695798810799e-05 | FAM83D | 527 | 0.188077128171274 |
| FAM83A | 418 | 0.0132431933937388 | FAM83A | 704 | 2.39095012019856e-05 | FAM83D | 542 | 0.193057871452002 |
| FAM83A | 418 | 0.0159212626621112 | FAM83A | 705 | 4.4428926872215e-05 | FAM83D | 553 | 0.20031503727764 |
| FAM83A | 419 | 0.0130429856095023 | FAM83A | 707 | 7.76572458234416e-05 | FAM83D | 559 | 0.225937349180665 |
| FAM83A | 420 | 0.0164363204409393 | FAM83A | 708 | 5.47216668032105e-05 | FAM83D | 566 | 0.235918119313632 |
| FAM83A | 421 | 0.0133232337643258 | FAM83A | 712 | 3.94223436763938e-05 | FAM83D | 573 | 0.378249263660785 |
| FAM83A | 421 | 0.0162523491465575 | FAM83A | 713 | 6.55730558249196e-05 | FAM83D | 578 | 0.274556100482789 |
| FAM83A | 422 | 0.0146366235604828 | FAM83A | 714 | 4.6485053125943e-05 | FAM83D | 586 | 0.27234487850012 |
| FAM83A | 423 | 0.0168019298280032 | FAM83A | 716 | 3.18366237805884e-05 | FAM83D | 591 | 0.204430689991366 |
| FAM83A | 425 | 0.0142929783717474 | FAM83A | 721 | 2.38831538041356e-05 | FAM83D | 607 | 0.164563357478013 |
| FAM83A | 425 | 0.0121198441962507 | FAM83A | 722 | 1.72168509186606e-05 | FAM83D | 618 | 0.205589563147488 |
| FAM83A | 425 | 0.0104731111346907 | FAM83A | 737 | 3.11378415795173e-05 | FAM83D | 627 | 0.324898055081718 |
| FAM83A | 427 | 0.0121115829268278 | FAM83A | 745 | 2.20362134734772e-05 | FAM83D | 631 | 0.286578281746886 |
| FAM83A | 428 | 0.00992363710785491 | FAM83A | 748 | 3.9635214222632e-05 | FAM83D | 647 | 0.27090155245498 |
| FAM83A | 428 | 0.0074895202279312 | FAM83A | 751 | 2.83227065905864e-05 | FAM83D | 651 | 0.355947907165603 |
| FAM83A | 428 | 0.00619142213462068 | FAM83A | 761 | 5.21378291865858e-05 | FAM83D | 704 | 0.330236201196107 |
| FAM83A | 429 | 0.00516747173236482 | FAM83A | 773 | 3.92432117809097e-05 | FAM83D | 728 | 0.241706083821198 |
| FAM83A | 429 | 0.00623626907428926 | FAM83A | 781 | 2.62322487853028e-05 | FAM83D | 728 | 0.220011861609434 |
| FAM83A | 430 | 0.00729976456492224 | FAM83A | 784 | 2.08730060227774e-05 | FAM83D | 752 | 0.179017652020377 |
| FAM83A | 432 | 0.00933999388548333 | FAM83A | 785 | 2.51060086154616e-05 | FAM83D | 763 | 0.193222511802195 |
| FAM83A | 432 | 0.0115894990687578 | FAM83A | 788 | 1.71189407430275e-05 | FAM83D | 768 | 0.17180086761423 |
| FAM83A | 434 | 0.0102154518913991 | FAM83A | 792 | 1.18473441528226e-05 | FAM83D | 783 | 0.124509835448148 |
| FAM83A | 436 | 0.0123594742838197 | FAM83A | 808 | 1.7180678362364e-05 | FAM83D | 791 | 0.106393074927234 |
| FAM83A | 436 | 0.0144381839389392 | FAM83A | 815 | 2.95024927712931e-05 | FAM83D | 791 | 0.0988224032268664 |
| FAM83A | 437 | 0.0122031121716916 | FAM83A | 817 | 4.18107775347905e-05 | FAM83D | 852 | 0.190224854776256 |
| FAM83A | 437 | 0.0150081419201403 | FAM83A | 818 | 6.27433512359318e-05 | FAM83D | 860 | 0.148221727743929 |
| FAM83A | 439 | 0.0131616699717766 | FAM83A | 819 | 4.0918188064478e-05 | FAM83D | 926 | 0.114770352058349 |
| FAM83A | 440 | 0.0116308776845917 | FAM83A | 823 | 6.85501935161575e-05 | FAM83D | 928 | 0.0719076730219824 |
| FAM83A | 440 | 0.0100321416360702 | FAM83A | 834 | 0.000113176484259618 | FAM83D | 944 | 0.119790559895887 |
| FAM83A | 443 | 0.0116318837609076 | FAM83A | 837 | 7.46657535681728e-05 | FAM83D | 973 | 0.0803596632973199 |
| FAM83A | 448 | 0.0145896917798818 | FAM83A | 842 | 5.89521422316583e-05 | FAM83D | 993 | 0.0495280174107864 |
| FAM83A | 448 | 0.0178943231959226 | FAM83A | 842 | 4.84332147254544e-05 | FAM83D | 995 | 0.0380235524242672 |
| FAM83A | 449 | 0.0163383996341265 | FAM83A | 854 | 3.24796258592149e-05 | FAM83D | 1009 | 0.0387490015007545 |
| FAM83A | 449 | 0.0128741668249974 | FAM83A | 855 | 5.88880373701407e-05 | FAM83D | 1025 | 0.0478321619158998 |
| FAM83A | 451 | 0.0118402179535158 | FAM83A | 855 | 0.000102551273202838 | FAM83D | 1049 | 0.0287324513327699 |
| FAM83A | 451 | 0.00950809109284527 | FAM83A | 856 | 8.26745134876859e-05 | FAM83D | 1050 | 0.0187144475009959 |
| FAM83A | 451 | 0.0080761895753586 | FAM83A | 867 | 5.62583450973576e-05 | FAM83D | 1084 | 0.0378470344864659 |
| FAM83A | 452 | 0.010251664995932 | FAM83A | 870 | 3.76303127190004e-05 | FAM83D | 1093 | 0.0385662041138331 |
| FAM83A | 454 | 0.009318506116909 | FAM83A | 881 | 3.18144097799893e-05 | FAM83D | 1097 | 0.024042669026504 |
| FAM83A | 457 | 0.0104918866100567 | FAM83A | 883 | 6.15309470324306e-05 | FAM83D | 1133 | 0.0360254498132882 |
| FAM83A | 458 | 0.00903717422842911 | FAM83A | 883 | 0.000105503241146712 | FAM83D | 1138 | 0.0540039998786093 |
| FAM83A | 459 | 0.00672038201891154 | FAM83A | 884 | 7.474360836626e-05 | FAM83D | 1148 | 0.0929779950982681 |
| FAM83A | 459 | 0.00630167255785714 | FAM83A | 886 | 5.06265618716551e-05 | FAM83E | 12 | 0.332511791789337 |
| FAM83A | 460 | 0.0072761583518795 | FAM83A | 889 | 7.7072095756912e-05 | FAM83E | 12 | 0.390081449432407 |
| FAM83A | 461 | 0.00604370509015424 | FAM83A | 889 | 6.4976420758928e-05 | FAM83E | 12 | 0.423372505372145 |
| FAM83A | 461 | 0.00476098962314775 | FAM83A | 898 | 4.51267557949342e-05 | FAM83E | 12 | 0.507461395353351 |
| FAM83A | 462 | 0.00392235470797686 | FAM83A | 901 | 2.93978628828827e-05 | FAM83E | 12 | 0.602700262242416 |
| FAM83A | 464 | 0.00455364516387706 | FAM83A | 908 | 2.20025766549968e-05 | FAM83E | 12 | 0.57844890001161 |
| FAM83A | 465 | 0.00386584085688947 | FAM83A | 909 | 4.03332558125328e-05 | FAM83E | 12 | 0.676922748905469 |
| FAM83A | 468 | 0.00346054078009405 | FAM83A | 921 | 2.72195857648568e-05 | FAM83E | 12 | 0.49443034718393 |
| FAM83A | 468 | 0.00403323877800765 | FAM83A | 926 | 4.02361147936177e-05 | FAM83E | 12 | 0.493904405748422 |
| FAM83A | 472 | 0.0036683565065272 | FAM83A | 939 | 6.8563230047641e-05 | FAM83E | 12 | 0.273502833616093 |
| FAM83A | 473 | 0.00320189604411368 | FAM83A | 952 | 0.00011918587172728 | FAM83E | 13 | 0.296549254325949 |
| FAM83A | 474 | 0.00411874503278638 | FAM83A | 954 | 9.77208294630706e-05 | FAM83E | 13 | 0.167869637544312 |
| FAM83A | 474 | 0.0030762317148964 | FAM83A | 955 | 8.76835544530544e-05 | FAM83E | 13 | 0.176971055862625 |
| FAM83A | 476 | 0.00379128416087028 | FAM83A | 960 | 5.67170528952238e-05 | FAM83E | 13 | 0.208593710605891 |
| FAM83A | 477 | 0.00313104493858925 | FAM83A | 961 | 9.14785612246617e-05 | FAM83E | 13 | 0.257258186793952 |
| FAM83A | 478 | 0.00265544006958014 | FAM83B | 13 | 0.0299652364298813 | FAM83E | 13 | 0.312770492474218 |
| FAM83A | 479 | 0.00273771551632423 | FAM83B | 13 | 0.0418111243213931 | FAM83E | 13 | 0.333488420340402 |
| FAM83A | 482 | 0.00230661677482415 | FAM83B | 13 | 0.0568785541177991 | FAM83E | 14 | 0.382699701125266 |
| FAM83A | 485 | 0.00164286220112669 | FAM83B | 13 | 0.0477543614810443 | FAM83E | 14 | 0.446326252915017 |
| FAM83A | 485 | 0.00193588793019104 | FAM83B | 13 | 0.0409433677332041 | FAM83E | 14 | 0.469531392369379 |
| FAM83A | 487 | 0.00177710517264555 | FAM83B | 13 | 0.034880133412541 | FAM83E | 14 | 0.322671477979311 |
| FAM83A | 489 | 0.00148737061726368 | FAM83B | 14 | 0.0455460962812714 | FAM83E | 15 | 0.388345200921611 |
| FAM83A | 490 | 0.00190302725956922 | FAM83B | 14 | 0.0567570854256704 | FAM83E | 15 | 0.465145047243232 |
| FAM83A | 492 | 0.00250277734644497 | FAM83B | 14 | 0.0777145041966156 | FAM83E | 15 | 0.499386501767342 |
| FAM83A | 492 | 0.00242951096315089 | FAM83B | 14 | 0.0954349569604633 | FAM83E | 15 | 0.489116817915757 |
| FAM83A | 492 | 0.00303760991805794 | FAM83B | 15 | 0.0829949116986403 | FAM83E | 15 | 0.569257356897523 |
| FAM83A | 493 | 0.00363903173208448 | FAM83B | 15 | 0.0815037593510002 | FAM83E | 16 | 0.591919671284982 |
| FAM83A | 498 | 0.00394748406641934 | FAM83B | 15 | 0.069755754466643 | FAM83E | 16 | 0.690288132376033 |
| FAM83A | 500 | 0.0030311294498459 | FAM83B | 15 | 0.0594726321401704 | FAM83E | 17 | 0.73500460436053 |
| FAM83A | 504 | 0.0025773537840511 | FAM83B | 15 | 0.0787651971808506 | FAM83E | 17 | 0.758635442696735 |
| FAM83A | 504 | 0.00256915660242059 | FAM83B | 16 | 0.066876032041292 | FAM83E | 17 | 0.841486400291787 |
| FAM83A | 505 | 0.00217035053574078 | FAM83B | 16 | 0.0565418959165244 | FAM83E | 17 | 0.933121499551582 |
| FAM83A | 507 | 0.00188573063993283 | FAM83B | 16 | 0.0716141768148085 | FAM83E | 18 | 0.992279612446771 |
| FAM83A | 507 | 0.00157835269059492 | FAM83B | 16 | 0.0907168623802314 | FAM83E | 18 | 0.940223486751857 |
| FAM83A | 509 | 0.00203409988678959 | FAM83B | 16 | 0.119809694716084 | FAM83E | 18 | 0.919083160314673 |
| FAM83A | 512 | 0.00228848828610402 | FAM83B | 16 | 0.142169665046762 | FAM83E | 18 | 0.827565356009787 |
| FAM83A | 513 | 0.00197717765927232 | FAM83B | 16 | 0.138189321753662 | FAM83E | 19 | 0.819568003277207 |
| FAM83A | 514 | 0.00175732854420805 | FAM83B | 16 | 0.121876178504105 | FAM83E | 19 | 0.739172291719535 |
| FAM83A | 514 | 0.00141225986855288 | FAM83B | 17 | 0.153872883349997 | FAM83E | 19 | 0.79267389659523 |
| FAM83A | 515 | 0.00118299348479608 | FAM83B | 17 | 0.134593572286118 | FAM83E | 20 | 0.642562917570572 |
| FAM83A | 515 | 0.000959192657340392 | FAM83B | 17 | 0.123165825611234 | FAM83E | 20 | 0.712042592994856 |
| FAM83A | 515 | 0.000948708126853441 | FAM83B | 17 | 0.109875304041074 | FAM83E | 20 | 0.696192789312628 |
| FAM83A | 516 | 0.00127357628902689 | FAM83B | 17 | 0.130814228840361 | FAM83E | 20 | 0.694459252621007 |
| FAM83A | 517 | 0.00104066356751057 | FAM83B | 17 | 0.161998625573199 | FAM83E | 20 | 0.523267201094185 |
| FAM83A | 522 | 0.00119858656355269 | FAM83B | 17 | 0.142276612426537 | FAM83E | 20 | 0.607048974759218 |
| FAM83A | 523 | 0.00155073152919176 | FAM83B | 18 | 0.125717072560962 | FAM83E | 21 | 0.658775312186289 |
| FAM83A | 525 | 0.00136069277097977 | FAM83B | 18 | 0.111261123230053 | FAM83E | 21 | 0.702762980720315 |
| FAM83A | 526 | 0.00110100560333903 | FAM83B | 18 | 0.100827144745243 | FAM83E | 21 | 0.654920405951535 |
| FAM83A | 527 | 0.00110598563913498 | FAM83B | 18 | 0.0864631302356005 | FAM83E | 22 | 0.46319961188708 |
| FAM83A | 527 | 0.000885556940885613 | FAM83B | 18 | 0.0746897618182423 | FAM83E | 22 | 0.496227752492593 |
| FAM83A | 530 | 0.000705254179107506 | FAM83B | 18 | 0.0630001764574679 | FAM83E | 22 | 0.474292554133327 |
| FAM83A | 531 | 0.00059860502003379 | FAM83B | 19 | 0.0558655437260421 | FAM83E | 23 | 0.399323837189068 |
| FAM83A | 532 | 0.000473455768669141 | FAM83B | 19 | 0.0733177690209438 | FAM83E | 23 | 0.348080635669895 |
| FAM83A | 533 | 0.000592692122716785 | FAM83B | 19 | 0.0968252032500814 | FAM83E | 23 | 0.407214973860359 |
| FAM83A | 533 | 0.000490580157102612 | FAM83B | 19 | 0.117582724041462 | FAM83E | 23 | 0.423925356612226 |
| FAM83A | 533 | 0.000607523905768477 | FAM83B | 19 | 0.149016583169389 | FAM83E | 23 | 0.485475827577664 |
| FAM83A | 534 | 0.00049917435911794 | FAM83B | 20 | 0.130755864233139 | FAM83E | 23 | 0.414015469720532 |
| FAM83A | 534 | 0.000676348300696015 | FAM83B | 20 | 0.123256008586 | FAM83E | 23 | 0.409780998152493 |
| FAM83A | 536 | 0.000568264417961283 | FAM83B | 20 | 0.146245682307891 | FAM83E | 24 | 0.467261841827388 |
| FAM83A | 538 | 0.000739375932621021 | FAM83B | 20 | 0.129671904296361 | FAM83E | 24 | 0.417660266558635 |
| FAM83A | 540 | 0.000506701916785933 | FAM83B | 20 | 0.154503812681805 | FAM83E | 24 | 0.400209799875281 |
| FAM83A | 541 | 0.000662453626199688 | FAM83B | 20 | 0.199328469104439 | FAM83E | 24 | 0.4399255457401 |
| FAM83A | 541 | 0.000550769227560587 | FAM83B | 20 | 0.175920432116181 | FAM83E | 24 | 0.493712371344823 |
| FAM83A | 542 | 0.000430149286467131 | FAM83B | 20 | 0.161207016480567 | FAM83E | 24 | 0.467217949954433 |
| FAM83A | 544 | 0.000265675251895909 | FAM83B | 20 | 0.141889337051883 | FAM83E | 24 | 0.523309763883235 |
| FAM83A | 547 | 0.00035289138164974 | FAM83B | 20 | 0.1231643299754 | FAM83E | 24 | 0.561643006867114 |
| FAM83A | 547 | 0.000470205316865861 | FAM83B | 21 | 0.105657247989896 | FAM83E | 25 | 0.625329707654841 |
| FAM83A | 547 | 0.000492500428776941 | FAM83B | 21 | 0.130430824339639 | FAM83E | 25 | 0.603003464283618 |
| FAM83A | 550 | 0.000382157169442701 | FAM83B | 21 | 0.114328738063998 | FAM83E | 25 | 0.651871232383016 |
| FAM83A | 550 | 0.000273092469573474 | FAM83B | 22 | 0.0990468737070248 | FAM83E | 25 | 0.587691224998313 |
| FAM83A | 558 | 0.000214591796989632 | FAM83B | 22 | 0.0851072674984872 | FAM83E | 25 | 0.599003094392762 |
| FAM83A | 558 | 0.000155219345293224 | FAM83B | 22 | 0.105257531168663 | FAM83E | 25 | 0.577607403293506 |
| FAM83A | 565 | 0.000191793324169456 | FAM83B | 22 | 0.0932874695143566 | FAM83E | 25 | 0.632585095170717 |
| FAM83A | 567 | 0.000152768788516975 | FAM83B | 22 | 0.0806196574702365 | FAM83E | 26 | 0.545846142847986 |
| FAM83A | 569 | 0.000125178174751056 | FAM83B | 22 | 0.0694053688961141 | FAM83E | 27 | 0.495505960368648 |
| FAM83A | 570 | 0.000147955084404301 | FAM83B | 22 | 0.084585284837243 | FAM83E | 27 | 0.559068051600641 |
| FAM83A | 571 | 0.00019145117713046 | FAM83B | 22 | 0.104785782362001 | FAM83E | 28 | 0.590962302756423 |
| FAM83A | 573 | 0.000165752951410117 | FAM83B | 23 | 0.0908743938360747 | FAM83E | 28 | 0.581349692287264 |
| FAM83A | 573 | 0.000130522060669164 | FAM83B | 23 | 0.0860630771775316 | FAM83E | 28 | 0.554304623672816 |
| FAM83A | 576 | 0.000104705531350331 | FAM83B | 23 | 0.0731160810048344 | FAM83E | 28 | 0.406586261007693 |
| FAM83A | 576 | 0.000126293905790241 | FAM83B | 23 | 0.0962116877302983 | FAM83E | 28 | 0.460073005435054 |
| FAM83A | 577 | 0.00012530592369886 | FAM83B | 24 | 0.120960978161552 | FAM83E | 29 | 0.442597476442709 |
| FAM83A | 580 | 0.000150255331896753 | FAM83B | 24 | 0.105066759469256 | FAM83E | 29 | 0.479423760903169 |
| FAM83A | 581 | 0.000200179624755741 | FAM83B | 24 | 0.0908981382874356 | FAM83E | 29 | 0.52697266554026 |
| FAM83A | 581 | 0.000131586366121472 | FAM83B | 24 | 0.0789988230119748 | FAM83E | 30 | 0.583935208975567 |
| FAM83A | 584 | 0.000106822375893766 | FAM83B | 24 | 0.0669662632341072 | FAM83E | 30 | 0.511129044732701 |
| FAM83A | 586 | 6.90008139448635e-05 | FAM83B | 24 | 0.0651873737498236 | FAM83E | 31 | 0.392406846439488 |
| FAM83A | 586 | 8.87167547069851e-05 | FAM83B | 24 | 0.0808405246865794 | FAM83E | 31 | 0.441105744695271 |
| FAM83A | 586 | 0.000107613918397284 | FAM83B | 24 | 0.0699425814875961 | FAM83E | 31 | 0.339804845855362 |
| FAM83A | 587 | 8.86507256427495e-05 | FAM83B | 24 | 0.0848195446383198 | FAM83E | 31 | 0.372754866549335 |
| FAM83A | 588 | 0.000116784917100553 | FAM83B | 24 | 0.0759076211743766 | FAM83E | 31 | 0.400132934769846 |
| FAM83A | 588 | 9.26709620051169e-05 | FAM83B | 24 | 0.0674623386795287 | FAM83E | 31 | 0.427287217918575 |
| FAM83A | 590 | 0.000112621190295527 | FAM83B | 24 | 0.0586592774377597 | FAM83E | 31 | 0.347736469046298 |
| FAM83A | 590 | 0.00013942397529274 | FAM83B | 25 | 0.0520201911302213 | FAM83E | 32 | 0.413025092878141 |
| FAM83A | 592 | 0.000192007621117429 | FAM83B | 25 | 0.0659412358566238 | FAM83E | 32 | 0.427080861904759 |
| FAM83A | 592 | 0.000258058419094924 | FAM83B | 25 | 0.0859723542039344 | FAM83E | 32 | 0.479326225618798 |
| FAM83A | 593 | 0.000356990803215218 | FAM83B | 25 | 0.0962992388308418 | FAM83E | 32 | 0.544144299267411 |
| FAM83A | 594 | 0.000311889426353532 | FAM83B | 25 | 0.0899248970913258 | FAM83E | 32 | 0.496089136864844 |
| FAM83A | 596 | 0.000257313827777097 | FAM83B | 25 | 0.112214498337585 | FAM83E | 33 | 0.578563096321481 |
| FAM83A | 597 | 0.000188137422478762 | FAM83B | 25 | 0.0976086056665342 | FAM83E | 33 | 0.633138204239503 |
| FAM83A | 601 | 0.000249248972095924 | FAM83B | 26 | 0.0863751209042795 | FAM83E | 34 | 0.723123989488546 |
| FAM83A | 605 | 0.000282578144990859 | FAM83B | 26 | 0.0800051847316775 | FAM83E | 34 | 0.797559892788999 |
| FAM83A | 609 | 0.000246234061966021 | FAM83B | 26 | 0.0798873333786261 | FAM83E | 35 | 0.84565008689707 |
| FAM83A | 613 | 0.000201042021689564 | FAM83B | 27 | 0.0661900941607555 | FAM83E | 35 | 0.937654621858342 |
| FAM83A | 617 | 0.000243703118221605 | FAM83B | 27 | 0.0833234794955221 | FAM83E | 35 | 0.803831783687935 |
| FAM83A | 617 | 0.000272238846511339 | FAM83B | 27 | 0.0720457316097889 | FAM83E | 36 | 0.725254569184324 |
| FAM83A | 622 | 0.000352752849517139 | FAM83B | 27 | 0.0894702434978586 | FAM83E | 36 | 0.780778176597877 |
| FAM83A | 625 | 0.000479423057399827 | FAM83B | 27 | 0.116201804284678 | FAM83E | 36 | 0.826514876728693 |
| FAM83A | 628 | 0.000409602689912164 | FAM83B | 27 | 0.149575704796123 | FAM83E | 36 | 0.835591955364114 |
| FAM83A | 629 | 0.000350872107241693 | FAM83B | 27 | 0.131700795833483 | FAM83E | 36 | 0.909896461623182 |
| FAM83A | 630 | 0.000285957769847231 | FAM83B | 28 | 0.114351208982698 | FAM83E | 36 | 0.818790031468177 |
| FAM83A | 632 | 0.000334014005422034 | FAM83B | 28 | 0.0995405751569405 | FAM83E | 37 | 0.918559236527594 |
| FAM83A | 632 | 0.000395226991877449 | FAM83B | 28 | 0.0857047541427569 | FAM83E | 37 | 0.95198223743974 |
| FAM83A | 634 | 0.000339562543671631 | FAM83B | 28 | 0.110041072652139 | FAM83E | 38 | 0.919941479893897 |
| FAM83A | 636 | 0.000259769161352541 | FAM83B | 29 | 0.0970101387994382 | FAM83E | 38 | 0.939476898217448 |
| FAM83A | 636 | 0.000206314205984162 | FAM83B | 29 | 0.111707887439972 | FAM83E | 38 | 0.821058951568302 |
| FAM83A | 639 | 0.000280198393089903 | FAM83B | 30 | 0.142600607406099 | FAM83E | 39 | 0.913339967034061 |
| FAM83A | 648 | 0.000228700550989707 | FAM83B | 30 | 0.125393794862804 | FAM83E | 40 | 0.978548226546327 |
| FAM83A | 650 | 0.000227245903209557 | FAM83B | 30 | 0.111179433200907 | FAM83E | 40 | 0.88695126573623 |
| FAM83A | 651 | 0.000188360415613313 | FAM83B | 30 | 0.0980383234122054 | FAM83E | 40 | 0.755229179870972 |
| FAM83A | 652 | 0.000168244411126886 | FAM83B | 30 | 0.0853041223925085 | FAM83E | 41 | 0.701929886832422 |
| FAM83A | 652 | 0.000118074756524395 | FAM83B | 30 | 0.0763082855227589 | FAM83E | 41 | 0.771441399415687 |
| FAM83A | 652 | 0.000162969177296787 | FAM83B | 31 | 0.0671454914527523 | FAM83E | 41 | 0.823030704731813 |
| FAM83A | 654 | 0.00021435234720815 | FAM83B | 31 | 0.0586981041294135 | FAM83E | 42 | 0.839021614957939 |
| FAM83A | 655 | 0.000145844397231501 | FAM83B | 31 | 0.0510879712532675 | FAM83E | 43 | 0.706394573904569 |
| FAM83A | 656 | 0.000191702254934824 | FAM83B | 31 | 0.0669574557378016 | FAM83E | 43 | 0.774893158375949 |
| FAM83A | 658 | 0.000172279628219729 | FAM83B | 31 | 0.0863710939289579 | FAM83E | 43 | 0.815534800323975 |
| FAM83A | 660 | 0.00018563132240729 | FAM83B | 32 | 0.0740514848087955 | FAM83E | 44 | 0.710543714700857 |
| FAM83A | 660 | 0.000236793364049883 | FAM83B | 33 | 0.0647967913864577 | FAM83E | 44 | 0.774974942936387 |
| FAM83A | 660 | 0.00029035609694335 | FAM83B | 34 | 0.0553488709127206 | FAM83E | 45 | 0.805317893553822 |
| FAM83A | 661 | 0.000241295302325972 | FAM83B | 34 | 0.0475965726224978 | FAM83E | 45 | 0.847783626986886 |
| FAM83A | 662 | 0.000156991943044149 | FAM83B | 35 | 0.0407770783121584 | FAM83E | 45 | 0.813442470002072 |
| FAM83A | 664 | 0.00012345138685021 | FAM83B | 35 | 0.0346774487249513 | FAM83E | 45 | 0.754303924096738 |
| FAM83A | 666 | 0.000116316026125093 | FAM83B | 35 | 0.029136539450431 | FAM83E | 46 | 0.575008684997684 |
| FAM83A | 666 | 0.000146032366046684 | FAM83B | 35 | 0.0260721931084924 | FAM83E | 48 | 0.542186147090045 |
| FAM83A | 666 | 0.000183342318877915 | FAM83B | 35 | 0.0327041426392245 | FAM83E | 50 | 0.632093700384542 |
| FAM83A | 671 | 0.000252017340279421 | FAM83B | 36 | 0.0274330005782079 | FAM83E | 50 | 0.527204773784188 |
| FAM83A | 672 | 0.000209633631604408 | FAM83B | 36 | 0.0227913053164783 | FAM83E | 51 | 0.418987890211714 |
| FAM83A | 673 | 0.000164103639554646 | FAM83B | 37 | 0.0204660042496046 | FAM83E | 51 | 0.489820590338117 |
| FAM83A | 677 | 0.000190887669704732 | FAM83B | 37 | 0.0169680209762563 | FAM83E | 52 | 0.399719218660126 |
| FAM83A | 680 | 0.000252226122191091 | FAM83B | 37 | 0.0147458377435629 | FAM83E | 52 | 0.466351787766504 |
| FAM83A | 682 | 0.000338245426981137 | FAM83B | 39 | 0.0137992165215028 | FAM83E | 53 | 0.463377352323133 |
| FAM83A | 682 | 0.000273959069709968 | FAM83B | 39 | 0.0125442300959311 | FAM83E | 53 | 0.526314645244581 |
| FAM83A | 682 | 0.000185161669886825 | FAM83B | 39 | 0.0177854757939473 | FAM83E | 54 | 0.531766504267957 |
| FAM83A | 682 | 0.00014926766380169 | FAM83B | 39 | 0.0228717322703866 | FAM83E | 54 | 0.576517605973465 |
| FAM83A | 684 | 0.000131992514418159 | FAM83B | 40 | 0.0276714784559168 | FAM83E | 55 | 0.391155855948533 |
| FAM83A | 689 | 0.000114478237520496 | FAM83B | 40 | 0.0231799889573872 | FAM83E | 55 | 0.309682280633777 |
| FAM83A | 690 | 8.29410420584424e-05 | FAM83B | 41 | 0.0311819811336513 | FAM83E | 58 | 0.331596088420894 |
| FAM83A | 696 | 6.1968412827135e-05 | FAM83B | 42 | 0.0379931924625639 | FAM83E | 61 | 0.404089209686482 |
| FAM83A | 705 | 5.83625167635089e-05 | FAM83B | 42 | 0.0471463741688263 | FAM83E | 62 | 0.405406913627041 |
| FAM83A | 705 | 7.93803319841365e-05 | FAM83B | 43 | 0.0398789977883224 | FAM83E | 62 | 0.478637444212965 |
| FAM83A | 707 | 6.3075839939753e-05 | FAM83B | 43 | 0.0334149889956723 | FAM83E | 63 | 0.450349519604402 |
| FAM83A | 708 | 4.97597079501057e-05 | FAM83B | 43 | 0.0303287156338251 | FAM83E | 64 | 0.450106015597829 |
| FAM83A | 712 | 3.99158811242217e-05 | FAM83B | 44 | 0.03797190514256 | FAM83E | 65 | 0.52361897842223 |
| FAM83A | 713 | 5.16087361292428e-05 | FAM83B | 44 | 0.0518251714986916 | FAM83E | 65 | 0.598791041083464 |
| FAM83A | 714 | 5.98417288626668e-05 | FAM83B | 45 | 0.0444248138121223 | FAM83E | 65 | 0.558757912746195 |
| FAM83A | 716 | 4.62582588361087e-05 | FAM83B | 45 | 0.0376214845425268 | FAM83E | 67 | 0.635727521180104 |
| FAM83A | 721 | 3.88804498396699e-05 | FAM83B | 45 | 0.0446676747348733 | FAM83E | 68 | 0.628200425085186 |
| FAM83A | 721 | 4.66263972850762e-05 | FAM83B | 46 | 0.0577363448542986 | FAM83E | 69 | 0.590975994504747 |
| FAM83A | 722 | 5.55338867529033e-05 | FAM83B | 48 | 0.0512531209336769 | FAM83E | 70 | 0.58689905328701 |
| FAM83A | 722 | 7.26976326445981e-05 | FAM83B | 49 | 0.0463707849914986 | FAM83E | 71 | 0.412509246357063 |
| FAM83A | 724 | 0.000103814867671497 | FAM83B | 50 | 0.0386266051193044 | FAM83E | 72 | 0.425160769317729 |
| FAM83A | 724 | 0.000146210486810447 | FAM83B | 51 | 0.0360985711406946 | FAM83E | 72 | 0.510578689835496 |
| FAM83A | 729 | 0.000175275438910966 | FAM83B | 51 | 0.0298466184706004 | FAM83E | 73 | 0.415104042354805 |
| FAM83A | 729 | 0.000100918384103167 | FAM83B | 53 | 0.0247796587176919 | FAM83E | 74 | 0.49705554572934 |
| FAM83A | 732 | 7.57571549425817e-05 | FAM83B | 53 | 0.0208003277516068 | FAM83E | 75 | 0.449744039910832 |
| FAM83A | 734 | 9.75616179431285e-05 | FAM83B | 53 | 0.0169278176582522 | FAM83E | 76 | 0.434507818715874 |
| FAM83A | 737 | 0.000131182921554557 | FAM83B | 55 | 0.0219447805741139 | FAM83E | 78 | 0.374652767230843 |
| FAM83A | 737 | 0.000105951558787246 | FAM83B | 55 | 0.0310575076678131 | FAM83E | 78 | 0.379059060203949 |
| FAM83A | 739 | 0.000123797729483346 | FAM83B | 55 | 0.0414775315817778 | FAM83E | 79 | 0.41723527678227 |
| FAM83A | 740 | 9.27767377069907e-05 | FAM83B | 56 | 0.0351706701902461 | FAM83E | 80 | 0.475851966626484 |
| FAM83A | 741 | 0.000104888142554163 | FAM83B | 56 | 0.0295509793268997 | FAM83E | 81 | 0.530043824302455 |
| FAM83A | 745 | 0.000141830114610439 | FAM83B | 56 | 0.0247778964426999 | FAM83E | 82 | 0.568088738560408 |
| FAM83A | 746 | 0.00019080606928665 | FAM83B | 56 | 0.0337408154720746 | FAM83F | 50 | 0.92813782356075 |
| FAM83A | 748 | 0.000126275908778318 | FAM83B | 57 | 0.0300411888795365 | FAM83F | 50 | 0.885898265756349 |
| FAM83A | 751 | 0.000102844817382866 | FAM83B | 57 | 0.0262369768548096 | FAM83F | 52 | 0.656704342110778 |
| FAM83A | 751 | 0.000137749080015857 | FAM83B | 58 | 0.0217854848489383 | FAM83F | 52 | 0.462004651768751 |
| FAM83A | 754 | 0.000108039187809256 | FAM83B | 58 | 0.0179007810820248 | FAM83F | 52 | 0.581281269245498 |
| FAM83A | 756 | 9.21150028664938e-05 | FAM83B | 58 | 0.0146302883516002 | FAM83F | 53 | 0.696856018559889 |
| FAM83A | 757 | 5.71092202536563e-05 | FAM83B | 59 | 0.0119623491370408 | FAM83F | 54 | 0.512644990945282 |
| FAM83A | 757 | 8.32826277964764e-05 | FAM83B | 59 | 0.0163013279304131 | FAM83F | 54 | 0.587420318093091 |
| FAM83A | 766 | 0.000120627383033176 | FAM83B | 59 | 0.014518598772987 | FAM83F | 54 | 0.392105939354646 |
| FAM83A | 773 | 0.000158165418057155 | FAM83B | 60 | 0.0119098242835793 | FAM83F | 55 | 0.226401857842855 |
| FAM83A | 777 | 0.000119493430760929 | FAM83B | 60 | 0.0150081131108988 | FAM83F | 55 | 0.306066677756656 |
| FAM83A | 778 | 0.000167830301287407 | FAM83B | 61 | 0.0123147060828623 | FAM83F | 55 | 0.399473103868692 |
| FAM83A | 779 | 0.000209819357474267 | FAM83B | 61 | 0.0175398832634093 | FAM83F | 55 | 0.427275153837671 |
| FAM83A | 781 | 0.000282470391007003 | FAM83B | 62 | 0.01500687471999 | FAM83F | 56 | 0.549441006372794 |
| FAM83A | 781 | 0.000398473540841589 | FAM83B | 64 | 0.0147366907711166 | FAM83F | 56 | 0.587092391189467 |
| FAM83A | 784 | 0.000355617304921747 | FAM83B | 65 | 0.0189025533483266 | FAM83F | 57 | 0.452258515032134 |
| FAM83A | 785 | 0.000470878734671535 | FAM83B | 65 | 0.0163133760768747 | FAM83F | 57 | 0.447125733255034 |
| FAM83A | 786 | 0.000381406300198406 | FAM83B | 66 | 0.0217153641294575 | FAM83F | 57 | 0.581947171941933 |
| FAM83A | 788 | 0.000448880224240318 | FAM83B | 66 | 0.0210078198867953 | FAM83F | 57 | 0.693039238370893 |
| FAM83A | 789 | 0.000520727246828186 | FAM83B | 67 | 0.0184812206233135 | FAM83F | 58 | 0.823187473797624 |
| FAM83A | 790 | 0.000716299622544005 | FAM83B | 67 | 0.022389089436493 | FAM83F | 59 | 0.921049836664346 |
| FAM83A | 792 | 0.000872109330898186 | FAM83B | 68 | 0.018523805687354 | FAM83F | 60 | 0.763129742590352 |
| FAM83A | 792 | 0.00105500095483249 | FAM83B | 70 | 0.0151018906723253 | FAM83F | 60 | 0.703647102758053 |
| FAM83A | 793 | 0.00121984762072474 | FAM83B | 70 | 0.012314422964091 | FAM83F | 61 | 0.669966944054654 |
| FAM83A | 794 | 0.00129444448669077 | FAM83B | 71 | 0.0121454446566001 | FAM83F | 62 | 0.749900314181893 |
| FAM83A | 800 | 0.00158566806320143 | FAM83B | 71 | 0.010493969937795 | FAM83F | 64 | 0.746240406332731 |
| FAM83A | 802 | 0.0015530534292644 | FAM83B | 73 | 0.00847506022151132 | FAM83F | 70 | 0.533305888202621 |
| FAM83A | 804 | 0.00110664383832044 | FAM83B | 73 | 0.00683607088375636 | FAM83F | 70 | 0.665344029543788 |
| FAM83A | 808 | 0.000939495582219186 | FAM83B | 74 | 0.0066632932432782 | FAM83F | 71 | 0.746984113575951 |
| FAM83A | 815 | 0.00122704147439369 | FAM83B | 76 | 0.00589564920779006 | FAM83F | 73 | 0.619852045919989 |
| FAM83A | 817 | 0.00141628325861659 | FAM83B | 78 | 0.00521866787633837 | FAM83F | 74 | 0.726688794575983 |
| FAM83A | 818 | 0.00124057253719031 | FAM83B | 78 | 0.00735845707009207 | FAM83F | 74 | 0.487868145821824 |
| FAM83A | 819 | 0.000934358405979655 | FAM83B | 78 | 0.00584608558592764 | FAM83F | 74 | 0.336423607868355 |
| FAM83A | 820 | 0.000737734847199901 | FAM83B | 78 | 0.00482707742855708 | FAM83F | 75 | 0.22637513985145 |
| FAM83A | 823 | 0.00062189616216482 | FAM83B | 79 | 0.00374596050442918 | FAM83F | 76 | 0.282989165955345 |
| FAM83A | 826 | 0.000741498594683864 | FAM83B | 79 | 0.00546593490433181 | FAM83F | 76 | 0.288805084012405 |
| FAM83A | 834 | 0.000496406730115762 | FAM83B | 80 | 0.00425148117266034 | FAM83F | 76 | 0.327397048527428 |
| FAM83A | 835 | 0.000372024696495667 | FAM83B | 80 | 0.00363388681498705 | FAM83F | 77 | 0.234891966995375 |
| FAM83A | 836 | 0.000372991855399021 | FAM83B | 80 | 0.00295756589120667 | FAM83F | 78 | 0.189096175125378 |
| FAM83A | 837 | 0.000420093914916357 | FAM83B | 81 | 0.00237170583729999 | FAM83F | 83 | 0.116898064100925 |
| FAM83A | 838 | 0.000369889975012392 | FAM83B | 81 | 0.00206722429091454 | FAM83F | 86 | 0.174655019632134 |
| FAM83A | 838 | 0.000270530851609766 | FAM83B | 84 | 0.00150661745770696 | FAM83F | 89 | 0.101845021438049 |
| FAM83A | 840 | 0.000356554044602305 | FAM83B | 84 | 0.00116298036683205 | FAM83F | 89 | 0.101158462542319 |
| FAM83A | 842 | 0.000451042708659293 | FAM83B | 85 | 0.00182735892543662 | FAM83F | 90 | 0.0827451195010653 |
| FAM83A | 842 | 0.000630327488484794 | FAM83B | 86 | 0.0026729306596934 | FAM83F | 91 | 0.128845292555641 |
| FAM83A | 842 | 0.000531855640329162 | FAM83B | 87 | 0.00218871491545293 | FAM83F | 92 | 0.0814644881339078 |
| FAM83A | 842 | 0.000483174549913168 | FAM83B | 87 | 0.00172265428932393 | FAM83F | 94 | 0.112487765481215 |
| FAM83A | 843 | 0.000384018414014472 | FAM83B | 88 | 0.00132403530625892 | FAM83F | 96 | 0.101489398202026 |
| FAM83A | 843 | 0.000305791777476309 | FAM83B | 89 | 0.00101100704715748 | FAM83F | 97 | 0.056402462430949 |
| FAM83A | 851 | 0.000236320350818527 | FAM83B | 91 | 0.00143715361761557 | FAM83F | 97 | 0.0879876401698503 |
| FAM83A | 852 | 0.000335799487343871 | FAM83B | 92 | 0.00113369762036239 | FAM83F | 99 | 0.138905781028534 |
| FAM83A | 854 | 0.000439704593083557 | FAM83B | 96 | 0.000872608447846077 | FAM83F | 100 | 0.119715114775133 |
| FAM83A | 855 | 0.000384978778466849 | FAM83B | 97 | 0.00139241704382516 | FAM83F | 100 | 0.179605832986998 |
| FAM83A | 855 | 0.000517047200127298 | FAM83B | 97 | 0.00129766457246353 | FAM83F | 101 | 0.183245694753876 |
| FAM83A | 856 | 0.000461738354291689 | FAM83B | 100 | 0.000987863778625973 | FAM83F | 102 | 0.216740650977336 |
| FAM83A | 857 | 0.000368267612673097 | FAM83B | 102 | 0.000754131857734109 | FAM83F | 103 | 0.284275698015034 |
| FAM83A | 861 | 0.000501019990708082 | FAM83B | 102 | 0.0005696259640966 | FAM83F | 104 | 0.210280781991104 |
| FAM83A | 866 | 0.00062846937482937 | FAM83B | 103 | 0.000444193634010618 | FAM83F | 106 | 0.147601174238935 |
| FAM83A | 867 | 0.000717161874493858 | FAM83B | 103 | 0.000346937241708822 | FAM83F | 108 | 0.0640403510577907 |
| FAM83A | 870 | 0.000567799995856912 | FAM83B | 104 | 0.000511266431266937 | FAM83F | 110 | 0.0976675729925157 |
| FAM83A | 876 | 0.000703467810040065 | FAM83B | 104 | 0.000692951790921179 | FAM83F | 111 | 0.0433498389594003 |
| FAM83A | 883 | 0.000458723551852713 | FAM83B | 106 | 0.000520858453867329 | FAM83F | 111 | 0.0249317132952745 |
| FAM83A | 883 | 0.000592139665257451 | FAM83B | 108 | 0.000401402128132558 | FAM83F | 112 | 0.0286888056995541 |
| FAM83A | 884 | 0.00048588751987253 | FAM83B | 109 | 0.000295913616764389 | FAM83F | 115 | 0.0249137803575892 |
| FAM83A | 886 | 0.000388237682718688 | FAM83B | 111 | 0.000211032758215917 | FAM83F | 121 | 0.00885663741843722 |
| FAM83A | 889 | 0.000473525927040083 | FAM83B | 112 | 0.000165131662309535 | FAM83F | 121 | 0.0132512884800702 |
| FAM83A | 889 | 0.000623285602609557 | FAM83B | 112 | 0.000282763937927173 | FAM83F | 122 | 0.0129392586372059 |
| FAM83A | 898 | 0.000809778252168737 | FAM83B | 114 | 0.000209426148904415 | FAM83F | 127 | 0.021532784607585 |
| FAM83A | 901 | 0.000626810414885209 | FAM83B | 117 | 0.000309361917120177 | FAM83F | 131 | 0.0360911025692826 |
| FAM83A | 901 | 0.000840392069153204 | FAM83B | 120 | 0.000244596025318297 | FAM83F | 131 | 0.0634307412944012 |
| FAM83A | 902 | 0.000731375904883663 | FAM83B | 120 | 0.000175855252757158 | FAM83F | 133 | 0.0372413073519867 |
| FAM83A | 903 | 0.00102513462916334 | FAM83B | 121 | 0.000133725713585257 | FAM83H | 830 | 0.000645957356142973 |
| FAM83A | 905 | 0.000814067021103392 | FAM83B | 124 | 0.000132719380368511 | FAM83H | 831 | 0.00120542995929841 |
| FAM83A | 908 | 0.00113067691153144 | FAM83B | 130 | 0.00020640679553408 | FAM83H | 860 | 0.000679726113098758 |
| FAM83A | 909 | 0.00107563260907645 | FAM83B | 131 | 0.000173461538956501 | FAM83H | 877 | 0.000233279108272578 |
| FAM83A | 914 | 0.000875351428236691 | FAM83B | 133 | 0.000123376246473225 | FAM83H | 888 | 0.000311219114369987 |
| FAM83A | 915 | 0.00083301857691612 | FAM83B | 133 | 0.000167427273982269 | FAM83H | 892 | 0.000266844762866704 |
| FAM83A | 921 | 0.000865482727150783 | FAM83B | 133 | 0.000294794753939015 | FAM83H | 919 | 0.000400313729467659 |
| FAM83A | 924 | 0.000948333063534116 | FAM83B | 137 | 0.00047887893768994 | FAM83H | 932 | 0.000463615086297069 |
| FAM83A | 925 | 0.00120432369188507 | FAM83B | 141 | 0.000326309090327015 | FAM83H | 934 | 0.000717672873629111 |
| FAM83A | 926 | 0.000854925561699487 | FAM83B | 141 | 0.000252042956161167 | FAM83H | 936 | 0.00107707770710217 |
| FAM83A | 926 | 0.000638313820849353 | FAM83B | 142 | 0.000392115289399737 | FAM83H | 941 | 0.00200703878873404 |
| FAM83A | 928 | 0.000584498457816419 | FAM83B | 144 | 0.000651407034440349 | FAM83H | 952 | 0.000817054674397959 |
| FAM83A | 932 | 0.000502309498325863 | FAM83B | 145 | 0.00104122794512568 | FAM83H | 952 | 0.001724919761863 |
| FAM83A | 939 | 0.000328012116723785 | FAM83B | 146 | 0.000824089369645822 | FAM83H | 970 | 0.00179041599961443 |
| FAM83A | 939 | 0.000412548421004458 | FAM83B | 149 | 0.00136155448696035 | FAM83H | 970 | 0.000765307314099006 |
| FAM83A | 941 | 0.000505309058786921 | FAM83B | 153 | 0.00102498220113493 | FAM83H | 975 | 0.000970640670434057 |
| FAM83A | 945 | 0.000331050043987712 | FAM83B | 154 | 0.000768918551466583 | FAM83H | 1000 | 0.00156455453610767 |
| FAM83A | 945 | 0.00045549713505674 | FAM83B | 155 | 0.00127190815437372 | FAM83H | 1025 | 0.00220555312235829 |
| FAM83A | 949 | 0.000596792094538205 | FAM83B | 155 | 0.00104894961400604 | FAM83H | 1032 | 0.00412024281042537 |
| FAM83A | 950 | 0.000822439788492893 | FAM83B | 155 | 0.00163959814901087 | FAM83H | 1035 | 0.00387992108507398 |
| FAM83A | 952 | 0.000904188287460186 | FAM83B | 156 | 0.0012362825184256 | FAM83H | 1051 | 0.00648765128773164 |
| FAM83A | 954 | 0.000820304083358325 | FAM83B | 156 | 0.00091931432912036 | FAM83H | 1066 | 0.00746444587670278 |
| FAM83A | 954 | 0.00111237044366614 | FAM83B | 159 | 0.00143258941244159 | FAM83H | 1075 | 0.0119004160758797 |
| FAM83A | 955 | 0.000773307713051892 | FAM83B | 161 | 0.00110845229456498 | FAM83H | 1116 | 0.00835612934913541 |
| FAM83A | 957 | 0.000583174721011409 | FAM83B | 162 | 0.00156077837755604 | FAM83H | 1118 | 0.0127831542928279 |
| FAM83A | 960 | 0.000457958000857024 | FAM83B | 164 | 0.00108554809306824 | FAM83H | 1121 | 0.0100794693060226 |
| FAM83A | 961 | 0.00040282999420049 | FAM83B | 167 | 0.000832680893602316 | FAM83H | 1152 | 0.0151515636311386 |
| FAM83A | 962 | 0.000287452365015081 | FAM83B | 167 | 0.000625155787630934 | FAM83H | 1153 | 0.0254260441895979 |
| FAM83A | 962 | 0.00034466569320732 | FAM83B | 168 | 0.000449820394865703 | FAM83H | 1157 | 0.0214332880029463 |
| FAM83A | 963 | 0.000497227024802022 | FAM83B | 169 | 0.00060732518870628 | FAM83H | 1162 | 0.0120425853383459 |
| FAM83A | 965 | 0.000527617134706394 | FAM83B | 172 | 0.000484534630562856 | FAM83H | 1167 | 0.0129134335790054 |
| FAM83A | 966 | 0.000607420624901473 | FAM83B | 174 | 0.000662187215735214 | FAM83H | 1176 | 0.00603267587350474 |
| FAM83A | 968 | 0.000540928894124126 | FAM83B | 174 | 0.000487028611255718 | FAM83H | 1194 | 0.00333262982117783 |
| FAM83A | 969 | 0.000334062989520082 | FAM83B | 177 | 0.000783118536494 | FAM83H | 1196 | 0.000934695824474841 |
| FAM83A | 970 | 0.000234771563765181 | FAM83B | 177 | 0.00125198091940524 | FAM83H | 1206 | 0.00137272046625064 |
| FAM83A | 972 | 0.000313069089171681 | FAM83B | 178 | 0.001029388773949 | FAM83H | 1227 | 0.0026009188853314 |
| FAM83A | 973 | 0.000241873909651451 | FAM83B | 180 | 0.00075192404746685 | FAM83H | 1234 | 0.00219831088919412 |
| FAM83A | 975 | 0.00030548084506242 | FAM83B | 181 | 0.000582076160726558 | FAM83H | 1242 | 0.000667392967281995 |
| FAM83A | 977 | 0.000401430028229165 | FAM83B | 183 | 0.000980421421999589 | FAM83H | 1274 | 0.00116703136490198 |
| FAM83A | 993 | 0.000464725128864958 | FAM83B | 184 | 0.000724341639470715 | FAM83H | 1290 | 0.00031754642637551 |
| FAM83A | 995 | 0.000390656795375566 | FAM83B | 184 | 0.00119205661223538 | FAM83H | 1293 | 0.000146685941845451 |
| FAM83A | 996 | 0.000335969985323037 | FAM83B | 190 | 0.00166220333659606 | FAM83H | 1310 | 0.000135477530686617 |
| FAM83A | 999 | 0.000395536967228591 | FAM83B | 191 | 0.00124181061876737 | FAM83H | 1343 | 0.000187418236293722 |
| FAM83A | 100 | 0.000550439634587277 | FAM83B | 192 | 0.0016277841421129 | FAM83H | 1343 | 0.000184774451518766 |
| FAM83A | 100 | 0.000449264193127411 | FAM83B | 192 | 0.0013439222471542 | FAM83H | 1358 | 0.000131761476844997 |
| FAM83B | 18 | 9.33479326334673e-07 | FAM83B | 196 | 0.00106214230012132 | FAM83H | 1374 | 0.000197560646918287 |
| FAM83B | 18 | 6.59127439537832e-07 | FAM83B | 198 | 0.00181756069687693 | FAM83H | 1414 | 0.000235984986782582 |
| FAM83B | 19 | 5.47843717306667e-07 | FAM83B | 198 | 0.00301074985399496 | FAM83H | 1421 | 0.000100021557259168 |
| FAM83B | 19 | 8.08175436205427e-07 | FAM83B | 204 | 0.00242208316225611 | FAM83H | 1455 | 0.000157649163971151 |
| FAM83B | 19 | 1.21548122450727e-06 | FAM83B | 209 | 0.00196175016138659 | FAM83H | 1469 | 0.000349313153554225 |
| FAM83B | 19 | 1.90241125409672e-06 | FAM83B | 210 | 0.00146314290089929 | FAM83H | 1488 | 0.000328256058755914 |
| FAM83B | 19 | 2.88428103443971e-06 | FAM83B | 212 | 0.00107184459784605 | FAM83H | 1488 | 0.000631611384695905 |
| FAM83B | 19 | 3.7764226858761e-06 | FAM83B | 214 | 0.000896275048242132 | FAM83H | 1508 | 0.000515489581641241 |
| FAM83B | 19 | 2.93871529327115e-06 | FAM83B | 215 | 0.000688302695600098 | FAM83H | 1523 | 0.0011174406052292 |
| FAM83B | 20 | 2.30599842506402e-06 | FAM83B | 215 | 0.000514922494983903 | FAM83H | 1549 | 0.000909426943355237 |
| FAM83B | 20 | 3.11964446019925e-06 | FAM83B | 217 | 0.000852740529376973 | FAM83H | 1557 | 0.00182126974540011 |
| FAM83B | 20 | 4.13023344796185e-06 | FAM83B | 219 | 0.000730110982285659 | FAM83H | 1590 | 0.00252578231279982 |
| FAM83B | 20 | 3.76853712521789e-06 | FAM83B | 220 | 0.000477309091064693 | FAM83H | 1591 | 0.00287264657417062 |
| FAM83B | 20 | 4.80086656009901e-06 | FAM83B | 226 | 0.000389481760948998 | FAM83H | 1624 | 0.00443076617277589 |
| FAM83B | 20 | 3.71956643684634e-06 | FAM83B | 226 | 0.000276656378959624 | FAM83H | 1633 | 0.00398042138765551 |
| FAM83B | 20 | 2.87517869802467e-06 | FAM83B | 227 | 0.000184468923500471 | FAM83H | 1648 | 0.00290631114575944 |
| FAM83B | 20 | 4.12071273462365e-06 | FAM83B | 233 | 0.000123784789397009 | FAM83H | 1649 | 0.00494597106704965 |
| FAM83B | 20 | 5.86184932937033e-06 | FAM83B | 237 | 9.11307328330282e-05 | FAM83H | 1664 | 0.00714621897775793 |
| FAM83B | 20 | 4.58413717156363e-06 | FAM83B | 246 | 6.04539605763701e-05 | FAM83H | 1685 | 0.00217830246577818 |
| FAM83B | 20 | 3.90983809508266e-06 | FAM83B | 246 | 4.37325340088214e-05 | FAM83H | 1690 | 0.00213023091461697 |
| FAM83B | 20 | 3.06939594581185e-06 | FAM83B | 249 | 6.17728609151456e-05 | FAM83H | 1699 | 0.00476694468555219 |
| FAM83B | 20 | 2.26535528669404e-06 | FAM83B | 261 | 4.41956492577807e-05 | FAM83H | 1712 | 0.00973457677203462 |
| FAM83B | 21 | 1.58361789186575e-06 | FAM83B | 263 | 2.95439998419761e-05 | FAM83H | 1715 | 0.00680175123216097 |
| FAM83B | 21 | 1.91727334358485e-06 | FAM83B | 264 | 5.78039845520006e-05 | FAM83H | 1732 | 0.0106906766038812 |
| FAM83B | 21 | 1.16235844117887e-06 | FAM83B | 272 | 0.000105283952668824 | FAM83H | 1784 | 0.0201516734892825 |
| FAM83B | 21 | 1.63523337235805e-06 | FAM83C | 10 | 0.689850685449691 | FAM83H | 1816 | 0.0397819951740847 |
| FAM83B | 21 | 2.26668304411538e-06 | FAM83C | 10 | 0.777980730227992 |  |  |  |
| FAM83B | 21 | 3.50654795332957e-06 | FAM83C | 10 | 0.884818101166379 |  |  |  |
| FAM83B | 21 | 2.76598307716992e-06 | FAM83C | 10 | 0.823644279218592 |  |  |  |
| FAM83B | 22 | 2.11315345478328e-06 | FAM83C | 10 | 0.931188631352265 |  |  |  |
| FAM83B | 22 | 1.58379921281428e-06 | FAM83C | 10 | 0.878821864308764 |  |  |  |
| FAM83B | 22 | 2.16078538515671e-06 | FAM83C | 10 | 0.98336819775622 |  |  |  |
| FAM83B | 22 | 1.7601649311774e-06 | FAM83C | 10 | 0.900710686377505 |  |  |  |
| FAM83B | 22 | 2.49044237520897e-06 | FAM83C | 10 | 0.787860044993106 |  |  |  |
| FAM83B | 22 | 1.67042642694371e-06 | FAM83C | 10 | 0.864643647597131 |  |  |  |
| FAM83B | 22 | 1.28273298971028e-06 | FAM83C | 10 | 0.785646431462355 |  |  |  |
| FAM83B | 22 | 9.91344014749793e-07 | FAM83C | 11 | 0.694602905495664 |  |  |  |
| FAM83B | 22 | 8.31928586909827e-07 | FAM83C | 11 | 0.753357291876226 |  |  |  |
| FAM83B | 22 | 1.12056919743008e-06 | FAM83C | 11 | 0.800444475635644 |  |  |  |
| FAM83B | 23 | 8.64133067958891e-07 | FAM83C | 11 | 0.801872885584906 |  |  |  |
| FAM83B | 23 | 1.26541362490772e-06 | FAM83C | 11 | 0.849495155807827 |  |  |  |
| FAM83B | 23 | 1.92529819512391e-06 | FAM83C | 11 | 0.751753308397434 |  |  |  |
| FAM83B | 23 | 1.34666362392813e-06 | FAM83C | 11 | 0.804981951502409 |  |  |  |
| FAM83B | 23 | 1.96289644477545e-06 | FAM83C | 11 | 0.69336153432332 |  |  |  |
| FAM83B | 24 | 1.78430977478229e-06 | FAM83C | 11 | 0.721104682869642 |  |  |  |
| FAM83B | 24 | 1.10426915873002e-06 | FAM83C | 11 | 0.772111913662997 |  |  |  |
| FAM83B | 24 | 8.24931345205984e-07 | FAM83C | 11 | 0.679302492531511 |  |  |  |
| FAM83B | 24 | 6.18550113617606e-07 | FAM83C | 11 | 0.742650448576534 |  |  |  |
| FAM83B | 24 | 4.81668176024743e-07 | FAM83C | 11 | 0.804215151849446 |  |  |  |
| FAM83B | 24 | 3.39579116086834e-07 | FAM83C | 12 | 0.841343662676674 |  |  |  |
| FAM83B | 24 | 3.25207041159945e-07 | FAM83C | 12 | 0.90126665248265 |  |  |  |
| FAM83B | 24 | 3.84811298303709e-07 | FAM83C | 12 | 0.786782052886894 |  |  |  |
| FAM83B | 24 | 3.37360043972761e-07 | FAM83C | 12 | 0.831753204602589 |  |  |  |
| FAM83B | 24 | 5.14304765419888e-07 | FAM83C | 12 | 0.876399329424618 |  |  |  |
| FAM83B | 24 | 3.988284725945e-07 | FAM83C | 12 | 0.762701254016301 |  |  |  |
| FAM83B | 24 | 2.76669172882025e-07 | FAM83C | 12 | 0.797600860876474 |  |  |  |
| FAM83B | 24 | 2.29649408791045e-07 | FAM83C | 12 | 0.711078766755583 |  |  |  |
| FAM83B | 24 | 1.86850624964142e-07 | FAM83C | 13 | 0.783088327810362 |  |  |  |
| FAM83B | 24 | 1.45446421622978e-07 | FAM83C | 13 | 0.787087972826714 |  |  |  |
| FAM83B | 25 | 1.19042862186217e-07 | FAM83C | 13 | 0.839951584215026 |  |  |  |
| FAM83B | 25 | 1.28165020288347e-07 | FAM83C | 13 | 0.884950104372427 |  |  |  |
| FAM83B | 25 | 1.66188000082139e-07 | FAM83C | 13 | 0.792354025004637 |  |  |  |
| FAM83B | 25 | 2.53942167220228e-07 | FAM83C | 13 | 0.853504855950555 |  |  |  |
| FAM83B | 25 | 3.67214771014134e-07 | FAM83C | 13 | 0.741660828662201 |  |  |  |
| FAM83B | 25 | 4.81169044399514e-07 | FAM83C | 13 | 0.792907595589015 |  |  |  |
| FAM83B | 25 | 7.40488522797605e-07 | FAM83C | 13 | 0.831526317900699 |  |  |  |
| FAM83B | 25 | 6.66790555203847e-07 | FAM83C | 13 | 0.88841829195867 |  |  |  |
| FAM83B | 25 | 5.77213663656855e-07 | FAM83C | 13 | 0.948401981315124 |  |  |  |
| FAM83B | 25 | 4.44422290259204e-07 | FAM83C | 14 | 0.992727851250137 |  |  |  |
| FAM83B | 26 | 3.57906866256602e-07 | FAM83C | 14 | 0.94167104012681 |  |  |  |
| FAM83B | 26 | 5.30615464463011e-07 | FAM83C | 14 | 0.882430446092273 |  |  |  |
| FAM83B | 26 | 7.50645353304094e-07 | FAM83C | 14 | 0.827335977237062 |  |  |  |
| FAM83B | 26 | 7.47052741664574e-07 | FAM83C | 14 | 0.911485630081408 |  |  |  |
| FAM83B | 26 | 5.01236714461553e-07 | FAM83C | 14 | 0.853994206815959 |  |  |  |
| FAM83B | 26 | 3.14247130118635e-07 | FAM83C | 14 | 0.783045355081589 |  |  |  |
| FAM83B | 26 | 4.85509687132617e-07 | FAM83C | 14 | 0.851885797494231 |  |  |  |
| FAM83B | 26 | 3.31405528695602e-07 | FAM83C | 14 | 0.918120985868915 |  |  |  |
| FAM83B | 27 | 2.17062897796562e-07 | FAM83C | 14 | 0.999259594158359 |  |  |  |
| FAM83B | 27 | 3.44543765356679e-07 | FAM83C | 14 | 0.988689312089554 |  |  |  |
| FAM83B | 27 | 2.29736909307081e-07 | FAM83C | 14 | 0.972137670035541 |  |  |  |
| FAM83B | 27 | 1.75446107767958e-07 | FAM83C | 14 | 0.920064177823039 |  |  |  |
| FAM83B | 27 | 1.34626824677794e-07 | FAM83C | 15 | 0.84842585540039 |  |  |  |
| FAM83B | 27 | 1.0223360952316e-07 | FAM83C | 15 | 0.791430519404392 |  |  |  |
| FAM83B | 27 | 8.56475366411101e-08 | FAM83C | 15 | 0.734156836301609 |  |  |  |
| FAM83B | 27 | 1.02784145769362e-07 | FAM83C | 15 | 0.681780378556824 |  |  |  |
| FAM83B | 27 | 6.63131680337624e-08 | FAM83C | 15 | 0.635214196458692 |  |  |  |
| FAM83B | 27 | 7.74035220254842e-08 | FAM83C | 15 | 0.731983665968587 |  |  |  |
| FAM83B | 27 | 1.08499532396484e-07 | FAM83C | 15 | 0.680498493050271 |  |  |  |
| FAM83B | 27 | 8.32342336851047e-08 | FAM83C | 15 | 0.629229782040461 |  |  |  |
| FAM83B | 28 | 5.98990509493678e-08 | FAM83C | 15 | 0.579823886911741 |  |  |  |
| FAM83B | 28 | 5.24601198552215e-08 | FAM83C | 15 | 0.657348593673991 |  |  |  |
| FAM83B | 28 | 3.95964881007534e-08 | FAM83C | 15 | 0.607019898001915 |  |  |  |
| FAM83B | 28 | 2.85841343842923e-08 | FAM83C | 15 | 0.557674885512575 |  |  |  |
| FAM83B | 28 | 3.86335053884508e-08 | FAM83C | 15 | 0.506724054282478 |  |  |  |
| FAM83B | 28 | 2.53051021600055e-08 | FAM83C | 15 | 0.473094554652804 |  |  |  |
| FAM83B | 28 | 1.47235593598152e-08 | FAM83C | 16 | 0.440534868166752 |  |  |  |
| FAM83B | 29 | 1.12898759560945e-08 | FAM83C | 16 | 0.51838888914397 |  |  |  |
| FAM83B | 29 | 7.95959152208477e-09 | FAM83C | 16 | 0.492005745470588 |  |  |  |
| FAM83B | 29 | 9.72122543524627e-09 | FAM83C | 16 | 0.550204591993128 |  |  |  |
| FAM83B | 29 | 1.44097563564288e-08 | FAM83C | 16 | 0.504454766525055 |  |  |  |
| FAM83B | 30 | 1.94549493460477e-08 | FAM83C | 16 | 0.458824359575413 |  |  |  |
| FAM83B | 30 | 2.90219997839967e-08 | FAM83C | 16 | 0.427234713919411 |  |  |  |
| FAM83B | 30 | 2.04951217659022e-08 | FAM83C | 16 | 0.388882476620068 |  |  |  |
| FAM83B | 30 | 1.55281634687137e-08 | FAM83C | 17 | 0.351788166750541 |  |  |  |
| FAM83B | 30 | 2.55153341428831e-08 | FAM83C | 17 | 0.32671375922682 |  |  |  |
| FAM83B | 30 | 1.93627808845407e-08 | FAM83C | 17 | 0.290427681698741 |  |  |  |
| FAM83B | 30 | 1.58110826138234e-08 | FAM83C | 17 | 0.357559384967507 |  |  |  |
| FAM83B | 31 | 1.24701970428791e-08 | FAM83C | 17 | 0.430872072841548 |  |  |  |
| FAM83B | 31 | 1.28700786533811e-08 | FAM83C | 17 | 0.392441250105833 |  |  |  |
| FAM83B | 31 | 1.58068690822697e-08 | FAM83C | 17 | 0.361998946534305 |  |  |  |
| FAM83B | 31 | 2.24006187769145e-08 | FAM83C | 17 | 0.423820157683025 |  |  |  |
| FAM83B | 31 | 3.51866848159588e-08 | FAM83C | 17 | 0.480126084582224 |  |  |  |
| FAM83B | 31 | 2.75674671745974e-08 | FAM83C | 17 | 0.441687578905766 |  |  |  |
| FAM83B | 31 | 2.02274840944412e-08 | FAM83C | 17 | 0.397709692885179 |  |  |  |
| FAM83B | 31 | 2.24880520588077e-08 | FAM83C | 17 | 0.459153498190818 |  |  |  |
| FAM83B | 31 | 3.32930457656118e-08 | FAM83C | 17 | 0.505465609292233 |  |  |  |
| FAM83B | 31 | 2.50096757639011e-08 | FAM83C | 17 | 0.462277676497427 |  |  |  |
| FAM83B | 32 | 1.77592206875062e-08 | FAM83C | 17 | 0.426159231319646 |  |  |  |
| FAM83B | 32 | 1.38489213534788e-08 | FAM83C | 18 | 0.390841811944734 |  |  |  |
| FAM83B | 32 | 2.10776229066273e-08 | FAM83C | 18 | 0.441717272306237 |  |  |  |
| FAM83B | 32 | 1.46499443777046e-08 | FAM83C | 18 | 0.511055496740501 |  |  |  |
| FAM83B | 32 | 1.04161887845138e-08 | FAM83C | 18 | 0.469324695991349 |  |  |  |
| FAM83B | 33 | 1.58603324645183e-08 | FAM83C | 18 | 0.461839758875972 |  |  |  |
| FAM83B | 33 | 1.93415355486245e-08 | FAM83C | 18 | 0.426145510343139 |  |  |  |
| FAM83B | 33 | 2.73270689438257e-08 | FAM83C | 18 | 0.507726273368931 |  |  |  |
| FAM83B | 34 | 1.8554062404684e-08 | FAM83C | 18 | 0.464592933923637 |  |  |  |
| FAM83B | 34 | 2.73874070261213e-08 | FAM83C | 18 | 0.42283778775998 |  |  |  |
| FAM83B | 34 | 3.64010252332782e-08 | FAM83C | 18 | 0.386365824707528 |  |  |  |
| FAM83B | 34 | 4.54945253598456e-08 | FAM83C | 18 | 0.424124464082428 |  |  |  |
| FAM83B | 34 | 7.01524304591867e-08 | FAM83C | 18 | 0.389992507338679 |  |  |  |
| FAM83B | 35 | 5.39200924405699e-08 | FAM83C | 18 | 0.376854984597114 |  |  |  |
| FAM83B | 35 | 7.78892054325738e-08 | FAM83C | 18 | 0.341888707066275 |  |  |  |
| FAM83B | 35 | 9.59595701363285e-08 | FAM83C | 19 | 0.307560036550167 |  |  |  |
| FAM83B | 35 | 1.25652059906985e-07 | FAM83C | 19 | 0.27665377874459 |  |  |  |
| FAM83B | 35 | 9.66170140161245e-08 | FAM83C | 19 | 0.248323701024197 |  |  |  |
| FAM83B | 35 | 1.1377304223438e-07 | FAM83C | 19 | 0.222105907882813 |  |  |  |
| FAM83B | 35 | 1.54824897242784e-07 | FAM83C | 19 | 0.278303929609259 |  |  |  |
| FAM83B | 35 | 2.05999423540004e-07 | FAM83C | 19 | 0.320810159726115 |  |  |  |
| FAM83B | 35 | 2.59643727075159e-07 | FAM83C | 19 | 0.289020965262861 |  |  |  |
| FAM83B | 35 | 2.76312714044362e-07 | FAM83C | 19 | 0.256049958450021 |  |  |  |
| FAM83B | 35 | 3.0960643580529e-07 | FAM83C | 19 | 0.300294933659925 |  |  |  |
| FAM83B | 35 | 1.93185681143638e-07 | FAM83C | 19 | 0.269498170710989 |  |  |  |
| FAM83B | 35 | 1.97946442188598e-07 | FAM83C | 19 | 0.309157700673885 |  |  |  |
| FAM83B | 36 | 1.4903194647938e-07 | FAM83C | 19 | 0.277741514388731 |  |  |  |
| FAM83B | 36 | 1.07606597723381e-07 | FAM83C | 19 | 0.249354534612265 |  |  |  |
| FAM83B | 36 | 6.64432469612583e-08 | FAM83C | 20 | 0.223068865525926 |  |  |  |
| FAM83B | 36 | 4.7365987363677e-08 | FAM83C | 20 | 0.199346005598911 |  |  |  |
| FAM83B | 36 | 7.13586299128866e-08 | FAM83C | 20 | 0.189231735787478 |  |  |  |
| FAM83B | 37 | 6.16968841177932e-08 | FAM83C | 20 | 0.169459556895817 |  |  |  |
| FAM83B | 37 | 4.58293822940707e-08 | FAM83C | 20 | 0.211235803282659 |  |  |  |
| FAM83B | 37 | 6.10226941371661e-08 | FAM83C | 20 | 0.187328114950998 |  |  |  |
| FAM83B | 37 | 3.40037557027886e-08 | FAM83C | 20 | 0.22636416812073 |  |  |  |
| FAM83B | 38 | 2.4486706122238e-08 | FAM83C | 20 | 0.200024265670596 |  |  |  |
| FAM83B | 38 | 1.6733514150672e-08 | FAM83C | 20 | 0.177061217587423 |  |  |  |
| FAM83B | 38 | 2.73119599100901e-08 | FAM83C | 20 | 0.164306879286478 |  |  |  |
| FAM83B | 39 | 4.41071744038264e-08 | FAM83C | 20 | 0.205295298351965 |  |  |  |
| FAM83B | 39 | 3.91576656412386e-08 | FAM83C | 20 | 0.182490255948829 |  |  |  |
| FAM83B | 39 | 3.31124593379242e-08 | FAM83C | 20 | 0.164050759826358 |  |  |  |
| FAM83B | 40 | 2.48481537647648e-08 | FAM83C | 20 | 0.145017291570855 |  |  |  |
| FAM83B | 40 | 1.86731140900451e-08 | FAM83C | 21 | 0.126232031094854 |  |  |  |
| FAM83B | 40 | 2.90320283095465e-08 | FAM83C | 21 | 0.161092097377969 |  |  |  |
| FAM83B | 41 | 2.29282917738132e-08 | FAM83C | 21 | 0.206077887695263 |  |  |  |
| FAM83B | 41 | 2.86766256822661e-08 | FAM83C | 21 | 0.245270938706977 |  |  |  |
| FAM83B | 42 | 3.52882327529118e-08 | FAM83C | 21 | 0.293947877928892 |  |  |  |
| FAM83B | 42 | 5.28701179528438e-08 | FAM83C | 21 | 0.26436914402832 |  |  |  |
| FAM83B | 42 | 4.02961108725818e-08 | FAM83C | 21 | 0.2369025219579 |  |  |  |
| FAM83B | 43 | 2.97084674235779e-08 | FAM83C | 21 | 0.289977333219096 |  |  |  |
| FAM83B | 43 | 4.54939165895118e-08 | FAM83C | 21 | 0.34807346933141 |  |  |  |
| FAM83B | 43 | 4.24139191147391e-08 | FAM83C | 21 | 0.390375660326452 |  |  |  |
| FAM83B | 43 | 3.05837490026081e-08 | FAM83C | 21 | 0.357133014911343 |  |  |  |
| FAM83B | 43 | 4.30679479406996e-08 | FAM83C | 21 | 0.329665188889261 |  |  |  |
| FAM83B | 44 | 3.00861674857857e-08 | FAM83C | 22 | 0.297224345434166 |  |  |  |
| FAM83B | 44 | 4.16706292068132e-08 | FAM83C | 22 | 0.331592601572874 |  |  |  |
| FAM83B | 44 | 3.70726892166502e-08 | FAM83C | 22 | 0.37437171793026 |  |  |  |
| FAM83B | 44 | 5.34743683794165e-08 | FAM83C | 22 | 0.336900330538808 |  |  |  |
| FAM83B | 45 | 4.09596049630867e-08 | FAM83C | 22 | 0.317619996573821 |  |  |  |
| FAM83B | 45 | 5.51667890209943e-08 | FAM83C | 22 | 0.294237326115384 |  |  |  |
| FAM83B | 45 | 4.1365511506724e-08 | FAM83C | 22 | 0.36137684735149 |  |  |  |
| FAM83B | 45 | 3.14458971981778e-08 | FAM83C | 22 | 0.358009570001851 |  |  |  |
| FAM83B | 45 | 4.46192934797081e-08 | FAM83C | 22 | 0.424548390124454 |  |  |  |
| FAM83B | 45 | 6.63629113574757e-08 | FAM83C | 22 | 0.389457858056809 |  |  |  |
| FAM83B | 45 | 5.34186964649373e-08 | FAM83C | 22 | 0.45783586632281 |  |  |  |
| FAM83B | 46 | 5.10752315153907e-08 | FAM83C | 22 | 0.426507151337853 |  |  |  |
| FAM83B | 46 | 7.12402945044707e-08 | FAM83C | 22 | 0.388651090074485 |  |  |  |
| FAM83B | 47 | 1.01516239523831e-07 | FAM83C | 22 | 0.354714271935367 |  |  |  |
| FAM83B | 48 | 7.79639209769454e-08 | FAM83C | 22 | 0.320972810205857 |  |  |  |
| FAM83B | 49 | 8.77470199210153e-08 | FAM83C | 23 | 0.28789886013825 |  |  |  |
| FAM83B | 49 | 5.97998844793821e-08 | FAM83C | 23 | 0.262200977676509 |  |  |  |
| FAM83B | 50 | 7.85285948465062e-08 | FAM83C | 23 | 0.30195708427475 |  |  |  |
| FAM83B | 50 | 7.16936890101922e-08 | FAM83C | 23 | 0.273702578904326 |  |  |  |
| FAM83B | 51 | 1.06123512951368e-07 | FAM83C | 23 | 0.241684462494948 |  |  |  |
| FAM83B | 51 | 7.45697505547338e-08 | FAM83C | 23 | 0.297337246276608 |  |  |  |
| FAM83B | 51 | 5.41904982764617e-08 | FAM83C | 23 | 0.281382236361677 |  |  |  |
| FAM83B | 51 | 7.85549086770404e-08 | FAM83C | 23 | 0.25466962562725 |  |  |  |
| FAM83B | 52 | 1.15301949662084e-07 | FAM83C | 23 | 0.2915442118371 |  |  |  |
| FAM83B | 52 | 1.44142050516223e-07 | FAM83C | 23 | 0.335799868871807 |  |  |  |
| FAM83B | 53 | 1.16812831440348e-07 | FAM83C | 23 | 0.379652252235066 |  |  |  |
| FAM83B | 53 | 1.39238624091993e-07 | FAM83C | 23 | 0.34153790084655 |  |  |  |
| FAM83B | 53 | 1.07129735925842e-07 | FAM83C | 23 | 0.309447570416785 |  |  |  |
| FAM83B | 53 | 8.77143372316571e-08 | FAM83C | 24 | 0.276078379630052 |  |  |  |
| FAM83B | 55 | 7.58018262619803e-08 | FAM83C | 24 | 0.270685269091376 |  |  |  |
| FAM83B | 55 | 7.43109215184649e-08 | FAM83C | 24 | 0.327698833102588 |  |  |  |
| FAM83B | 55 | 1.1054547419986e-07 | FAM83C | 24 | 0.300769598579227 |  |  |  |
| FAM83B | 56 | 8.4416211303201e-08 | FAM83C | 24 | 0.276710785183738 |  |  |  |
| FAM83B | 56 | 9.22902116500922e-08 | FAM83C | 24 | 0.336695065749381 |  |  |  |
| FAM83B | 56 | 6.97665166273474e-08 | FAM83C | 24 | 0.392317518207011 |  |  |  |
| FAM83B | 56 | 5.29904191632035e-08 | FAM83C | 24 | 0.353237913454795 |  |  |  |
| FAM83B | 56 | 4.07865934834891e-08 | FAM83C | 24 | 0.316654011553494 |  |  |  |
| FAM83B | 57 | 3.43649402109663e-08 | FAM83C | 24 | 0.366649718471347 |  |  |  |
| FAM83B | 57 | 2.80894716743191e-08 | FAM83C | 24 | 0.331752215837585 |  |  |  |
| FAM83B | 57 | 2.05165282462198e-08 | FAM83C | 24 | 0.289750591321952 |  |  |  |
| FAM83B | 58 | 1.55583135859819e-08 | FAM83C | 24 | 0.259842182738875 |  |  |  |
| FAM83B | 58 | 2.43043310712915e-08 | FAM83C | 25 | 0.2300121752637 |  |  |  |
| FAM83B | 58 | 1.74753263180084e-08 | FAM83C | 25 | 0.279931058502486 |  |  |  |
| FAM83B | 58 | 1.23489107572033e-08 | FAM83C | 25 | 0.250277284635445 |  |  |  |
| FAM83B | 59 | 8.94699465905529e-09 | FAM83C | 25 | 0.225064775951647 |  |  |  |
| FAM83B | 59 | 7.65449660289086e-09 | FAM83C | 25 | 0.270160853878769 |  |  |  |
| FAM83B | 60 | 5.64297592404787e-09 | FAM83C | 25 | 0.241530447549481 |  |  |  |
| FAM83B | 60 | 7.77027483859372e-09 | FAM83C | 25 | 0.219436149904346 |  |  |  |
| FAM83B | 60 | 7.53439618344229e-09 | FAM83C | 26 | 0.193567120698251 |  |  |  |
| FAM83B | 60 | 3.74778461514683e-09 | FAM83C | 26 | 0.169320206116984 |  |  |  |
| FAM83B | 60 | 2.96253505578409e-09 | FAM83C | 26 | 0.147949785076859 |  |  |  |
| FAM83B | 61 | 2.17282117596086e-09 | FAM83C | 26 | 0.174757132527575 |  |  |  |
| FAM83B | 61 | 1.51681909344758e-09 | FAM83C | 26 | 0.158057683714829 |  |  |  |
| FAM83B | 61 | 1.04099281346859e-09 | FAM83C | 26 | 0.144138673029134 |  |  |  |
| FAM83B | 61 | 9.98392768896641e-10 | FAM83C | 26 | 0.125535994690111 |  |  |  |
| FAM83B | 61 | 6.78853016469021e-10 | FAM83C | 26 | 0.109595782121233 |  |  |  |
| FAM83B | 61 | 9.66681268907832e-10 | FAM83C | 26 | 0.100999574643432 |  |  |  |
| FAM83B | 62 | 7.53439245385299e-10 | FAM83C | 26 | 0.0888704690124775 |  |  |  |
| FAM83B | 62 | 5.93440468392195e-10 | FAM83C | 26 | 0.0767488083468664 |  |  |  |
| FAM83B | 62 | 9.63775142312428e-10 | FAM83C | 26 | 0.065754668176458 |  |  |  |
| FAM83B | 64 | 1.53535192386752e-09 | FAM83C | 26 | 0.0556251785840566 |  |  |  |
| FAM83B | 65 | 2.44943905667456e-09 | FAM83C | 27 | 0.046966301539833 |  |  |  |
| FAM83B | 66 | 1.99944695828783e-09 | FAM83C | 27 | 0.0401389418387609 |  |  |  |
| FAM83B | 66 | 3.19958789998463e-09 | FAM83C | 27 | 0.0342628639604969 |  |  |  |
| FAM83B | 67 | 2.64355989595793e-09 | FAM83C | 27 | 0.0291248810910173 |  |  |  |
| FAM83B | 67 | 1.95283977020289e-09 | FAM83C | 27 | 0.0238906372327165 |  |  |  |
| FAM83B | 67 | 2.4385991014622e-09 | FAM83C | 27 | 0.0206197727499944 |  |  |  |
| FAM83B | 68 | 1.78285520085309e-09 | FAM83C | 27 | 0.0168348898387157 |  |  |  |
| FAM83B | 68 | 2.6460378807583e-09 | FAM83C | 27 | 0.0144907177964363 |  |  |  |
| FAM83B | 68 | 1.68451326772625e-09 | FAM83C | 27 | 0.0121310345239108 |  |  |  |
| FAM83B | 69 | 1.17062749246741e-09 | FAM83C | 27 | 0.00985579413413596 |  |  |  |
| FAM83B | 70 | 1.80997481962216e-09 | FAM83C | 28 | 0.00791364151842964 |  |  |  |
| FAM83B | 70 | 1.29881562411159e-09 | FAM83C | 28 | 0.0110293155069286 |  |  |  |
| FAM83B | 71 | 2.13484103982368e-09 | FAM83C | 28 | 0.0146379938556359 |  |  |  |
| FAM83B | 71 | 3.15043446200698e-09 | FAM83C | 28 | 0.0123478443999996 |  |  |  |
| FAM83B | 71 | 2.57446469341445e-09 | FAM83C | 28 | 0.0175875330683503 |  |  |  |
| FAM83B | 71 | 1.84613061317258e-09 | FAM83C | 28 | 0.0232183655418016 |  |  |  |
| FAM83B | 72 | 1.36297231416313e-09 | FAM83C | 28 | 0.0298684675130921 |  |  |  |
| FAM83B | 72 | 2.02460874144741e-09 | FAM83C | 28 | 0.0252910048385884 |  |  |  |
| FAM83B | 72 | 1.48786853384739e-09 | FAM83C | 28 | 0.0205691194445743 |  |  |  |
| FAM83B | 72 | 1.32383543696042e-09 | FAM83C | 28 | 0.01643709703463 |  |  |  |
| FAM83B | 73 | 2.17503248710851e-09 | FAM83C | 28 | 0.013187813969238 |  |  |  |
| FAM83B | 73 | 2.66807490960852e-09 | FAM83C | 29 | 0.0108551190884857 |  |  |  |
| FAM83B | 73 | 2.22879647309831e-09 | FAM83C | 29 | 0.0150557928567655 |  |  |  |
| FAM83B | 73 | 2.16523294351016e-09 | FAM83C | 29 | 0.0118942668287619 |  |  |  |
| FAM83B | 74 | 3.00769151405308e-09 | FAM83C | 29 | 0.0157462102547873 |  |  |  |
| FAM83B | 74 | 3.58535664278003e-09 | FAM83C | 29 | 0.0227339888071835 |  |  |  |
| FAM83B | 75 | 3.08934030777201e-09 | FAM83C | 29 | 0.0297492477743232 |  |  |  |
| FAM83B | 75 | 2.39124964499548e-09 | FAM83C | 29 | 0.0414102813143573 |  |  |  |
| FAM83B | 76 | 1.49712349083469e-09 | FAM83C | 29 | 0.0337195435587385 |  |  |  |
| FAM83B | 76 | 2.17213223953302e-09 | FAM83C | 29 | 0.0283526708736862 |  |  |  |
| FAM83B | 76 | 3.49437486023832e-09 | FAM83C | 29 | 0.0382313617057633 |  |  |  |
| FAM83B | 76 | 2.43842982751351e-09 | FAM83C | 29 | 0.0484690369782244 |  |  |  |
| FAM83B | 77 | 1.91030192990656e-09 | FAM83C | 29 | 0.0403851082692809 |  |  |  |
| FAM83B | 77 | 1.18598324388436e-09 | FAM83C | 30 | 0.0333164617832283 |  |  |  |
| FAM83B | 77 | 1.04586919782987e-09 | FAM83C | 30 | 0.0287136413322498 |  |  |  |
| FAM83B | 77 | 1.60437291510412e-09 | FAM83C | 30 | 0.0347663712022876 |  |  |  |
| FAM83B | 78 | 2.32503794181239e-09 | FAM83C | 30 | 0.0298593820408094 |  |  |  |
| FAM83B | 78 | 3.86176650841634e-09 | FAM83C | 30 | 0.0384304173337836 |  |  |  |
| FAM83B | 78 | 5.17076004481349e-09 | FAM83C | 30 | 0.0505073006053729 |  |  |  |
| FAM83B | 78 | 3.49579987609871e-09 | FAM83C | 30 | 0.0660999703292305 |  |  |  |
| FAM83B | 79 | 2.28827571988285e-09 | FAM83C | 30 | 0.0599337486915669 |  |  |  |
| FAM83B | 79 | 1.66478863245835e-09 | FAM83C | 30 | 0.050118868915158 |  |  |  |
| FAM83B | 79 | 1.54847155939305e-09 | FAM83C | 30 | 0.0417631307685346 |  |  |  |
| FAM83B | 80 | 1.00364667107113e-09 | FAM83C | 30 | 0.0354304261680122 |  |  |  |
| FAM83B | 80 | 7.20375773636703e-10 | FAM83C | 31 | 0.0292569712811552 |  |  |  |
| FAM83B | 80 | 5.46266445584608e-10 | FAM83C | 31 | 0.0376618660849831 |  |  |  |
| FAM83B | 81 | 4.03563429918472e-10 | FAM83C | 31 | 0.0293726804478118 |  |  |  |
| FAM83B | 81 | 2.79204896009611e-10 | FAM83C | 32 | 0.0234279552769551 |  |  |  |
| FAM83B | 81 | 2.3768373507795e-10 | FAM83C | 32 | 0.0194263826945388 |  |  |  |
| FAM83B | 83 | 1.40327239582255e-10 | FAM83C | 32 | 0.0277246998406541 |  |  |  |
| FAM83B | 83 | 1.91562758770778e-10 | FAM83C | 32 | 0.0225245763834944 |  |  |  |
| FAM83B | 83 | 2.67501814170562e-10 | FAM83C | 32 | 0.0224769343539883 |  |  |  |
| FAM83B | 84 | 1.57097920637148e-10 | FAM83C | 32 | 0.0183695436633908 |  |  |  |
| FAM83B | 84 | 1.91635937967486e-10 | FAM83C | 33 | 0.0160755947502282 |  |  |  |
| FAM83B | 84 | 2.97139321917691e-10 | FAM83C | 33 | 0.013586969841873 |  |  |  |
| FAM83B | 85 | 2.14777897963206e-10 | FAM83C | 33 | 0.0124730255333577 |  |  |  |
| FAM83B | 85 | 1.48056300114321e-10 | FAM83C | 33 | 0.0163301420958933 |  |  |  |
| FAM83B | 85 | 2.34362127925221e-10 | FAM83C | 33 | 0.023902993387328 |  |  |  |
| FAM83B | 85 | 3.90495886785264e-10 | FAM83C | 33 | 0.0192718421846517 |  |  |  |
| FAM83B | 86 | 2.67171023125872e-10 | FAM83C | 33 | 0.0154235521775542 |  |  |  |
| FAM83B | 86 | 3.47016731775679e-10 | FAM83C | 33 | 0.0211715556441439 |  |  |  |
| FAM83B | 86 | 2.60369893636957e-10 | FAM83C | 33 | 0.0171630627532616 |  |  |  |
| FAM83B | 87 | 2.13257715987405e-10 | FAM83C | 34 | 0.0137492258643103 |  |  |  |
| FAM83B | 87 | 1.5352769562497e-10 | FAM83C | 34 | 0.0193026471664182 |  |  |  |
| FAM83B | 87 | 1.10930487121861e-10 | FAM83C | 34 | 0.0167326761228988 |  |  |  |
| FAM83B | 88 | 7.58816853952078e-11 | FAM83C | 34 | 0.013372935076833 |  |  |  |
| FAM83B | 89 | 5.17164381105559e-11 | FAM83C | 34 | 0.0194431621441287 |  |  |  |
| FAM83B | 89 | 7.26439608914392e-11 | FAM83C | 35 | 0.0157811750305477 |  |  |  |
| FAM83B | 91 | 8.23234009596569e-11 | FAM83C | 35 | 0.0125279038362901 |  |  |  |
| FAM83B | 91 | 1.0228111767375e-10 | FAM83C | 35 | 0.010115559358625 |  |  |  |
| FAM83B | 91 | 1.44052323909903e-10 | FAM83C | 35 | 0.00804142369247672 |  |  |  |
| FAM83B | 91 | 7.71282745509831e-11 | FAM83C | 35 | 0.00637825296314636 |  |  |  |
| FAM83B | 91 | 9.89024895955624e-11 | FAM83C | 37 | 0.00486566062678743 |  |  |  |
| FAM83B | 92 | 7.2009241472258e-11 | FAM83C | 37 | 0.00765815513542235 |  |  |  |
| FAM83B | 92 | 1.06499360700852e-10 | FAM83C | 37 | 0.0109776689846162 |  |  |  |
| FAM83B | 94 | 7.60305260218922e-11 | FAM83C | 37 | 0.0152763143898179 |  |  |  |
| FAM83B | 94 | 1.21299242458074e-10 | FAM83C | 38 | 0.0120894429876566 |  |  |  |
| FAM83B | 94 | 1.66079058101645e-10 | FAM83C | 38 | 0.00880874006741618 |  |  |  |
| FAM83B | 96 | 2.73664922262538e-10 | FAM83C | 38 | 0.00693391797960582 |  |  |  |
| FAM83B | 96 | 3.98111032808209e-10 | FAM83C | 38 | 0.00872304011010698 |  |  |  |
| FAM83B | 97 | 2.68665751141244e-10 | FAM83C | 39 | 0.00730472930445707 |  |  |  |
| FAM83B | 97 | 3.67081343288169e-10 | FAM83C | 40 | 0.0108371952235315 |  |  |  |
| FAM83B | 97 | 5.90902168021729e-10 | FAM83C | 41 | 0.0157012463722081 |  |  |  |
| FAM83B | 97 | 4.0698977154083e-10 | FAM83C | 41 | 0.02165133684146 |  |  |  |
| FAM83B | 98 | 2.83046977307867e-10 | FAM83C | 41 | 0.0177220097673746 |  |  |  |
| FAM83B | 99 | 1.57304006554367e-10 | FAM83C | 41 | 0.0145617499958903 |  |  |  |
| FAM83B | 100 | 2.5087048288723e-10 | FAM83C | 43 | 0.0125096321838447 |  |  |  |
| FAM83B | 101 | 1.7913891714431e-10 | FAM83C | 44 | 0.0162144899757911 |  |  |  |
| FAM83B | 101 | 3.06165441690406e-10 | FAM83C | 44 | 0.0126483712472642 |  |  |  |
| FAM83B | 102 | 1.57918914843686e-10 | FAM83C | 44 | 0.0183856861223537 |  |  |  |
| FAM83B | 102 | 1.11258017349631e-10 | FAM83C | 44 | 0.0149173371886452 |  |  |  |
| FAM83B | 102 | 9.41645240958286e-11 | FAM83C | 45 | 0.0114288046413201 |  |  |  |
| FAM83B | 103 | 6.98273179359732e-11 | FAM83D | 327 | 0.826736428551557 |  |  |  |
| FAM83B | 103 | 1.02616615508965e-10 | FAM83D | 327 | 0.839706340508616 |  |  |  |
| FAM83B | 103 | 1.3459238150274e-10 | FAM83D | 328 | 0.902374243246122 |  |  |  |
| FAM83B | 103 | 1.87971508719631e-10 | FAM83D | 329 | 0.963556952391905 |  |  |  |
| FAM83B | 103 | 1.35105794818074e-10 | FAM83D | 332 | 0.993925686071471 |  |  |  |
| FAM83B | 104 | 2.04320153577536e-10 | FAM83D | 333 | 0.933205889116155 |  |  |  |
| FAM83B | 104 | 2.541234352587e-10 | FAM83D | 336 | 0.887568289711477 |  |  |  |
| FAM83B | 104 | 1.82951964234129e-10 | FAM83D | 337 | 0.985382919436975 |  |  |  |
| FAM83B | 105 | 1.28530707338636e-10 | FAM83D | 338 | 0.92222034197708 |  |  |  |
| FAM83B | 105 | 1.62391787102302e-10 | FAM83D | 338 | 0.911288614180653 |  |  |  |
| FAM83B | 106 | 8.43468958667571e-11 | FAM83D | 340 | 0.864357381964985 |  |  |  |
| FAM83B | 108 | 6.1789094686218e-11 | FAM83D | 340 | 0.940515891635533 |  |  |  |
| FAM83B | 109 | 4.10651252222541e-11 | FAM83D | 340 | 0.893883378838362 |  |  |  |
| FAM83B | 109 | 2.58433180910093e-11 | FAM83D | 343 | 0.827466963255245 |  |  |  |
| FAM83B | 109 | 2.19494360868838e-11 | FAM83D | 346 | 0.76859350172935 |  |  |  |
| FAM83B | 110 | 3.19328597970688e-11 | FAM83D | 350 | 0.711321789170196 |  |  |  |
| FAM83B | 111 | 5.62369574552846e-11 | FAM83D | 351 | 0.812049611573925 |  |  |  |
| FAM83B | 111 | 7.54775511881161e-11 | FAM83D | 355 | 0.925288712344682 |  |  |  |
| FAM83B | 111 | 1.1274188992406e-10 | FAM83D | 357 | 0.866707077053394 |  |  |  |
| FAM83B | 112 | 1.89760776978322e-10 | FAM83D | 361 | 0.82149303390739 |  |  |  |
| FAM83B | 113 | 1.34574951513301e-10 | FAM83D | 361 | 0.769379699640479 |  |  |  |
| FAM83B | 113 | 2.14841623794876e-10 | FAM83D | 363 | 0.698352694427109 |  |  |  |
| FAM83B | 114 | 1.15613598759586e-10 | FAM83D | 364 | 0.803804248538805 |  |  |  |
| FAM83B | 114 | 8.0714621335948e-11 | FAM83D | 364 | 0.913867582510297 |  |  |  |
| FAM83B | 116 | 5.4067601536717e-11 | FAM83D | 364 | 0.878741091220863 |  |  |  |
| FAM83B | 117 | 3.60656320358626e-11 | FAM83D | 366 | 0.987353316724963 |  |  |  |
| FAM83B | 118 | 2.75941293216577e-11 | FAM83D | 368 | 0.928922440053344 |  |  |  |
| FAM83B | 119 | 3.58064975957764e-11 | FAM83D | 369 | 0.993052744657569 |  |  |  |
| FAM83B | 120 | 3.38998972236325e-11 | FAM83D | 373 | 0.953291167976439 |  |  |  |
| FAM83B | 120 | 4.57452036269037e-11 | FAM83D | 374 | 0.904862809358231 |  |  |  |
| FAM83B | 120 | 2.95316975998574e-11 | FAM83D | 374 | 0.847692382733408 |  |  |  |
| FAM83B | 120 | 3.67623032935854e-11 | FAM83D | 378 | 0.961586262629298 |  |  |  |
| FAM83B | 120 | 5.56499139422576e-11 | FAM83D | 378 | 0.901180184268469 |  |  |  |
| FAM83B | 121 | 4.12652531953854e-11 | FAM83D | 380 | 0.892692253834628 |  |  |  |
| FAM83B | 121 | 4.47145167841993e-11 | FAM83D | 380 | 0.968549432301547 |  |  |  |
| FAM83B | 122 | 4.41310340504205e-11 | FAM83D | 381 | 0.911076379949092 |  |  |  |
| FAM83B | 124 | 3.09471639850233e-11 | FAM83D | 383 | 0.989072521065501 |  |  |  |
| FAM83B | 124 | 1.81941675766509e-11 | FAM83D | 387 | 0.928920454760631 |  |  |  |
| FAM83B | 126 | 1.28953778056352e-11 | FAM83D | 388 | 0.872131320598993 |  |  |  |
| FAM83B | 127 | 2.25815441018181e-11 | FAM83D | 389 | 0.826783548902036 |  |  |  |
| FAM83B | 127 | 3.17807849166466e-11 | FAM83D | 393 | 0.775639508136679 |  |  |  |
| FAM83B | 130 | 1.90748748153975e-11 | FAM83D | 397 | 0.873129591670172 |  |  |  |
| FAM83B | 130 | 3.33399817630053e-11 | FAM83D | 399 | 0.960361525942013 |  |  |  |
| FAM83B | 131 | 5.00218578046635e-11 | FAM83D | 403 | 0.905477089650292 |  |  |  |
| FAM83B | 131 | 8.80639522070905e-11 | FAM83D | 405 | 0.977742489275264 |  |  |  |
| FAM83B | 133 | 5.67938119523457e-11 | FAM83D | 405 | 0.970367144850053 |  |  |  |
| FAM83B | 133 | 7.24878090681348e-11 | FAM83D | 406 | 0.916726716737628 |  |  |  |
| FAM83B | 133 | 4.91264847085815e-11 | FAM83D | 411 | 0.860009134887373 |  |  |  |
| FAM83B | 134 | 6.48449605736246e-11 | FAM83D | 412 | 0.808304049913273 |  |  |  |
| FAM83B | 137 | 8.10743991761229e-11 | FAM83D | 413 | 0.768430032281746 |  |  |  |
| FAM83B | 139 | 1.09471656792658e-10 | FAM83D | 416 | 0.715382173144818 |  |  |  |
| FAM83B | 140 | 1.74280765776226e-10 | FAM83D | 417 | 0.779782567967548 |  |  |  |
| FAM83B | 141 | 2.73660498140444e-10 | FAM83D | 422 | 0.725919420413026 |  |  |  |
| FAM83B | 142 | 3.65916767910699e-10 | FAM83D | 423 | 0.831519519072314 |  |  |  |
| FAM83B | 142 | 2.28390381628914e-10 | FAM83D | 424 | 0.773207323600969 |  |  |  |
| FAM83B | 142 | 3.21146298919967e-10 | FAM83D | 427 | 0.71633822165589 |  |  |  |
| FAM83B | 143 | 5.49744805419272e-10 | FAM83D | 427 | 0.663680414974643 |  |  |  |
| FAM83B | 144 | 4.66157606934974e-10 | FAM83D | 427 | 0.600029815856877 |  |  |  |
| FAM83B | 145 | 6.89019664007468e-10 | FAM83D | 431 | 0.550673744503689 |  |  |  |
| FAM83B | 146 | 8.98205384376085e-10 | FAM83D | 435 | 0.509177255291638 |  |  |  |
| FAM83B | 149 | 1.36749794816159e-09 | FAM83D | 435 | 0.466107801986892 |  |  |  |
| FAM83B | 151 | 9.65700469982094e-10 | FAM83D | 437 | 0.419974465582899 |  |  |  |
| FAM83B | 152 | 5.73995379733516e-10 | FAM83D | 438 | 0.378807969014233 |  |  |  |
| FAM83B | 153 | 6.23406251838159e-10 | FAM83D | 441 | 0.340317317834735 |  |  |  |
| FAM83B | 153 | 4.95053004478916e-10 | FAM83D | 442 | 0.312302651532548 |  |  |  |
| FAM83B | 155 | 3.48960131443063e-10 | FAM83D | 443 | 0.284817028176479 |  |  |  |
| FAM83B | 155 | 2.79305353068065e-10 | FAM83D | 443 | 0.331266428778805 |  |  |  |
| FAM83B | 155 | 2.21172012068209e-10 | FAM83D | 445 | 0.297759798936453 |  |  |  |
| FAM83B | 156 | 1.53188757086637e-10 | FAM83D | 445 | 0.263603336282396 |  |  |  |
| FAM83B | 156 | 1.02556311944355e-10 | FAM83D | 445 | 0.244674797434485 |  |  |  |
| FAM83B | 157 | 1.67186017646155e-10 | FAM83D | 450 | 0.218406192369524 |  |  |  |
| FAM83B | 158 | 1.09197165128892e-10 | FAM83D | 451 | 0.247526530163963 |  |  |  |
| FAM83B | 159 | 1.63229162024152e-10 | FAM83D | 455 | 0.219850066164921 |  |  |  |
| FAM83B | 161 | 1.18522838883395e-10 | FAM83D | 458 | 0.217431728377457 |  |  |  |
| FAM83B | 161 | 1.97115675282099e-10 | FAM83D | 459 | 0.192771076056183 |  |  |  |
| FAM83B | 162 | 2.60741662446832e-10 | FAM83D | 460 | 0.240956163810685 |  |  |  |
| FAM83B | 163 | 1.31831950865515e-10 | FAM83D | 463 | 0.225430335337073 |  |  |  |
| FAM83B | 164 | 6.8984139456966e-11 | FAM83D | 464 | 0.200783611611751 |  |  |  |
| FAM83B | 167 | 5.01829401326999e-11 | FAM83D | 464 | 0.250661350089726 |  |  |  |
| FAM83B | 167 | 3.49031050549009e-11 | FAM83D | 466 | 0.288362056090949 |  |  |  |
| FAM83B | 168 | 2.19727012645262e-11 | FAM83D | 468 | 0.35128779893341 |  |  |  |
| FAM83B | 169 | 2.85122059756807e-11 | FAM83D | 469 | 0.390023449327059 |  |  |  |
| FAM83B | 170 | 2.21177519854243e-11 | FAM83D | 469 | 0.357087442297366 |  |  |  |
| FAM83B | 171 | 2.8492199558658e-11 | FAM83D | 470 | 0.322160153448669 |  |  |  |
| FAM83B | 172 | 1.64378279976388e-11 | FAM83D | 470 | 0.301737974401026 |  |  |  |
| FAM83B | 172 | 2.66820907233516e-11 | FAM83D | 471 | 0.35501734058871 |  |  |  |
| FAM83B | 174 | 3.55575754885385e-11 | FAM83D | 471 | 0.320272875179528 |  |  |  |
| FAM83B | 174 | 2.43984905742361e-11 | FAM83D | 475 | 0.376461428505029 |  |  |  |
| FAM83B | 174 | 2.4223238817391e-11 | FAM83D | 477 | 0.336817972500365 |  |  |  |
| FAM83B | 177 | 1.72113874434606e-11 | FAM83D | 478 | 0.384676839221248 |  |  |  |
| FAM83B | 178 | 1.36070356209226e-11 | FAM83D | 481 | 0.462671634895291 |  |  |  |
| FAM83B | 183 | 8.73471135980411e-12 | FAM83D | 481 | 0.424034474624986 |  |  |  |
| FAM83B | 183 | 1.42726472430331e-11 | FAM83D | 481 | 0.477013995727707 |  |  |  |
| FAM83B | 184 | 9.69697989119262e-12 | FAM83D | 484 | 0.433379509014975 |  |  |  |
| FAM83B | 187 | 7.4812696180491e-12 | FAM83D | 485 | 0.493426652587888 |  |  |  |
| FAM83B | 189 | 1.11008592003636e-11 | FAM83D | 487 | 0.448969670695013 |  |  |  |
| FAM83B | 190 | 1.99002865967927e-11 | FAM83D | 491 | 0.417932060343812 |  |  |  |
| FAM83B | 190 | 3.4597396765977e-11 | FAM83D | 492 | 0.390001788605799 |  |  |  |
| FAM83B | 191 | 2.36588808211815e-11 | FAM83D | 495 | 0.454953940336927 |  |  |  |
| FAM83B | 191 | 2.77646883931225e-11 | FAM83D | 497 | 0.520707684752816 |  |  |  |
| FAM83B | 192 | 1.90695100202393e-11 | FAM83D | 502 | 0.493871857756783 |  |  |  |
| FAM83B | 192 | 1.50609952062716e-11 | FAM83D | 504 | 0.449487637598994 |  |  |  |
| FAM83B | 193 | 1.12386219552852e-11 | FAM83D | 505 | 0.399454424922866 |  |  |  |
| FAM83B | 195 | 1.57745657514901e-11 | FAM83D | 506 | 0.479129468436412 |  |  |  |
| FAM83B | 196 | 2.66618755661646e-11 | FAM83D | 510 | 0.536850322293544 |  |  |  |
| FAM83B | 196 | 1.56375446287658e-11 | FAM83D | 512 | 0.481413278294314 |  |  |  |
| FAM83B | 197 | 1.285053932962e-11 | FAM83D | 512 | 0.439714170434866 |  |  |  |
| FAM83B | 197 | 1.61020800495818e-11 | FAM83D | 513 | 0.398337036643319 |  |  |  |
| FAM83B | 198 | 2.65269631320127e-11 | FAM83D | 514 | 0.360465557408608 |  |  |  |
| FAM83B | 200 | 2.0035235361544e-11 | FAM83D | 520 | 0.415393174972582 |  |  |  |
| FAM83B | 202 | 3.51624480110516e-11 | FAM83D | 521 | 0.377260572868909 |  |  |  |
| FAM83B | 204 | 5.84415606618138e-11 | FAM83D | 524 | 0.454760605328276 |  |  |  |
| FAM83B | 205 | 4.52134773082001e-11 | FAM83D | 527 | 0.53819531344277 |  |  |  |
| FAM83B | 207 | 6.10666113251911e-11 | FAM83D | 534 | 0.604172969485211 |  |  |  |
| FAM83B | 207 | 1.06687154499651e-10 | FAM83D | 536 | 0.557180208099308 |  |  |  |
| FAM83B | 209 | 1.42330757443573e-10 | FAM83D | 538 | 0.512072794979668 |  |  |  |
| FAM83B | 209 | 9.81326074952746e-11 | FAM83D | 539 | 0.46897363929873 |  |  |  |
| FAM83B | 210 | 8.00069821657907e-11 | FAM83D | 539 | 0.541180918359761 |  |  |  |
| FAM83B | 212 | 5.33306989756338e-11 | FAM83D | 540 | 0.513697165018263 |  |  |  |
| FAM83B | 214 | 4.38618568509187e-11 | FAM83D | 541 | 0.602318370999063 |  |  |  |
| FAM83B | 214 | 1.78139815970762e-11 | FAM83D | 542 | 0.553214220277325 |  |  |  |
| FAM83B | 215 | 1.29074524182524e-11 | FAM83D | 545 | 0.636000024867871 |  |  |  |
| FAM83B | 217 | 2.13923243384413e-11 | FAM83D | 546 | 0.585628353082737 |  |  |  |
| FAM83B | 217 | 3.32675721283871e-11 | FAM83D | 546 | 0.683072373163141 |  |  |  |
| FAM83B | 218 | 5.54611678304234e-11 | FAM83D | 548 | 0.620923399243662 |  |  |  |
| FAM83B | 218 | 8.90700912398327e-11 | FAM83D | 552 | 0.575642153491419 |  |  |  |
| FAM83B | 219 | 8.98838079672499e-11 | FAM83D | 553 | 0.525211570371197 |  |  |  |
| FAM83B | 219 | 4.26850589635899e-11 | FAM83D | 556 | 0.585023505056844 |  |  |  |
| FAM83B | 219 | 2.91402835574661e-11 | FAM83D | 559 | 0.541492699940032 |  |  |  |
| FAM83B | 220 | 4.84528614252176e-11 | FAM83D | 560 | 0.618438755846984 |  |  |  |
| FAM83B | 222 | 3.94522000982411e-11 | FAM83D | 561 | 0.697421295423106 |  |  |  |
| FAM83B | 225 | 2.26552008942367e-11 | FAM83D | 564 | 0.663424084209833 |  |  |  |
| FAM83B | 226 | 3.73386363336919e-11 | FAM83D | 566 | 0.625975500529764 |  |  |  |
| FAM83B | 226 | 2.53368713938722e-11 | FAM83D | 568 | 0.69690904433635 |  |  |  |
| FAM83B | 227 | 2.71309314598377e-11 | FAM83D | 568 | 0.669251066580465 |  |  |  |
| FAM83B | 227 | 4.62419803179937e-11 | FAM83D | 573 | 0.730815503549153 |  |  |  |
| FAM83B | 233 | 2.83962494014477e-11 | FAM83D | 574 | 0.8207886925149 |  |  |  |
| FAM83B | 234 | 2.09022322921318e-11 | FAM83D | 574 | 0.780429871132572 |  |  |  |
| FAM83B | 234 | 2.05348745289227e-11 | FAM83D | 578 | 0.735446663617805 |  |  |  |
| FAM83B | 235 | 3.26969097281635e-11 | FAM83D | 578 | 0.813046716410129 |  |  |  |
| FAM83B | 237 | 1.41293743998599e-11 | FAM83D | 581 | 0.767906559714033 |  |  |  |
| FAM83B | 240 | 9.04580169724497e-12 | FAM83D | 581 | 0.728344058475401 |  |  |  |
| FAM83B | 245 | 7.98041645211705e-12 | FAM83D | 584 | 0.676545001008715 |  |  |  |
| FAM83B | 246 | 1.29174292062739e-11 | FAM83D | 585 | 0.631440140568916 |  |  |  |
| FAM83B | 246 | 9.37755172021554e-12 | FAM83D | 586 | 0.610564694722064 |  |  |  |
| FAM83B | 246 | 5.54155580416103e-12 | FAM83D | 591 | 0.708794861013339 |  |  |  |
| FAM83B | 247 | 3.88986975404064e-12 | FAM83D | 591 | 0.675123272968808 |  |  |  |
| FAM83B | 247 | 6.82065478228412e-12 | FAM83D | 593 | 0.767758857430894 |  |  |  |
| FAM83B | 254 | 1.03812603836654e-11 | FAM83D | 596 | 0.728188707105156 |  |  |  |
| FAM83B | 257 | 5.30257623007550e-12 | FAM83D | 598 | 0.676374335419335 |  |  |  |
| FAM83B | 259 | 2.33397145595234e-12 | FAM83D | 601 | 0.627681477387736 |  |  |  |
| FAM83B | 260 | 2.70531134237231e-12 | FAM83D | 607 | 0.578951110406099 |  |  |  |
| FAM83B | 261 | 4.42225155232424e-12 | FAM83D | 607 | 0.540753323568129 |  |  |  |
| FAM83B | 264 | 2.9206511026217e-12 | FAM83D | 614 | 0.584959096215734 |  |  |  |
| FAM83B | 270 | 2.26533168046643e-12 | FAM83D | 618 | 0.537792316808776 |  |  |  |
| FAM83B | 272 | 3.75506805611173e-12 | FAM83D | 618 | 0.596558989516652 |  |  |  |
| FAM83B | 273 | 5.34276220405621e-12 | FAM83D | 622 | 0.558694975325674 |  |  |  |
| FAM83B | 273 | 6.97869169906145e-12 | FAM83D | 622 | 0.512561369154573 |  |  |  |
| FAM83B | 274 | 1.19598666409132e-11 | FAM83D | 627 | 0.602244686893388 |  |  |  |
| FAM83B | 274 | 1.77831528262099e-11 | FAM83D | 627 | 0.555906593275572 |  |  |  |
| FAM83B | 275 | 1.13380434887027e-11 | FAM83D | 630 | 0.626801201451611 |  |  |  |
| FAM83B | 276 | 5.81457939854776e-12 | FAM83D | 631 | 0.692459997318997 |  |  |  |
| FAM83B | 279 | 1.00598040714249e-11 | FAM83D | 646 | 0.778816635700292 |  |  |  |
| FAM83B | 280 | 1.3470240823906e-11 | FAM83D | 646 | 0.869328047009391 |  |  |  |
| FAM83B | 287 | 1.84459355793562e-11 | FAM83D | 647 | 0.934419916322032 |  |  |  |
| FAM83B | 292 | 1.49120263015716e-11 | FAM83D | 647 | 0.901771094494906 |  |  |  |
| FAM83B | 293 | 1.87133680129705e-11 | FAM83D | 648 | 0.989720379141007 |  |  |  |
| FAM83B | 295 | 2.43478312022519e-11 | FAM83D | 651 | 0.954433534106415 |  |  |  |
| FAM83B | 296 | 1.60004982008179e-11 | FAM83D | 658 | 0.958996221381466 |  |  |  |
| FAM83B | 297 | 2.77293257488366e-11 | FAM83D | 661 | 0.994119623926806 |  |  |  |
| FAM83B | 297 | 4.78239199650184e-11 | FAM83D | 666 | 0.959045666576157 |  |  |  |
| FAM83B | 298 | 5.17786021763559e-11 | FAM83D | 669 | 0.926271140627235 |  |  |  |
| FAM83B | 299 | 3.12199526696979e-11 | FAM83D | 670 | 0.873294809269373 |  |  |  |
| FAM83B | 302 | 3.91982149473111e-11 | FAM83D | 673 | 0.819384174296156 |  |  |  |
| FAM83B | 303 | 2.2567142662088e-11 | FAM83D | 675 | 0.767007317872217 |  |  |  |
| FAM83B | 303 | 2.9671509415843e-11 | FAM83D | 678 | 0.73745009468512 |  |  |  |
| FAM83B | 306 | 2.22390674687286e-11 | FAM83D | 679 | 0.833322311338743 |  |  |  |
| FAM83B | 307 | 3.72463910205365e-11 | FAM83D | 680 | 0.826939169296971 |  |  |  |
| FAM83B | 308 | 5.36868241947125e-11 | FAM83D | 680 | 0.80023964089221 |  |  |  |
| FAM83B | 309 | 9.4112164602231e-11 | FAM83D | 682 | 0.906657086599715 |  |  |  |
| FAM83B | 313 | 3.61323575473576e-11 | FAM83D | 686 | 0.852273563857626 |  |  |  |
| FAM83B | 315 | 4.59624376483032e-11 | FAM83D | 689 | 0.918497256332586 |  |  |  |
| FAM83B | 315 | 3.71738668805795e-11 | FAM83D | 691 | 0.86541903415852 |  |  |  |
| FAM83B | 315 | 2.48368228096087e-11 | FAM83D | 695 | 0.843662586020007 |  |  |  |
| FAM83B | 316 | 1.45529463227264e-11 | FAM83D | 697 | 0.789971411574699 |  |  |  |
| FAM83B | 316 | 8.29689551070965e-12 | FAM83D | 698 | 0.760049570337293 |  |  |  |
| FAM83B | 317 | 1.2978371223705e-11 | FAM83D | 704 | 0.861555785193584 |  |  |  |
| FAM83B | 322 | 2.07290507477703e-11 | FAM83D | 704 | 0.94364617773163 |  |  |  |
| FAM83B | 324 | 1.23988958624223e-11 | FAM83D | 708 | 0.890263984644315 |  |  |  |
| FAM83B | 324 | 5.22536948005686e-12 | FAM83D | 710 | 0.86024809354362 |  |  |  |
| FAM83B | 328 | 6.91321184073334e-12 | FAM83D | 712 | 0.807635084576928 |  |  |  |
| FAM83B | 329 | 1.08962000528684e-11 | FAM83D | 717 | 0.876419512873034 |  |  |  |
| FAM83B | 330 | 1.65146572131831e-11 | FAM83D | 720 | 0.81945946337828 |  |  |  |
| FAM83B | 330 | 2.25004407247896e-11 | FAM83D | 728 | 0.786468570883673 |  |  |  |
| FAM83B | 332 | 1.49174533208922e-11 | FAM83D | 728 | 0.875478614110557 |  |  |  |
| FAM83B | 333 | 1.90411981073829e-11 | FAM83D | 731 | 0.964868295696543 |  |  |  |
| FAM83B | 333 | 1.93831742273643e-11 | FAM83D | 732 | 0.911153893012306 |  |  |  |
| FAM83B | 333 | 2.15529030146003e-11 | FAM83D | 733 | 0.875982342750224 |  |  |  |
| FAM83B | 336 | 2.44093938627537e-11 | FAM83D | 733 | 0.822228383644226 |  |  |  |
| FAM83B | 344 | 4.54958274262994e-11 | FAM83D | 744 | 0.767403702771206 |  |  |  |
| FAM83B | 345 | 6.68607399585905e-11 | FAM83D | 746 | 0.742620037386051 |  |  |  |
| FAM83B | 347 | 1.18252652415753e-10 | FAM83D | 746 | 0.842522099980157 |  |  |  |
| FAM83B | 348 | 1.90576762519021e-10 | FAM83D | 752 | 0.935433761070577 |  |  |  |
| FAM83B | 349 | 8.33636566540219e-11 | FAM83D | 754 | 0.983291663485918 |  |  |  |
| FAM83B | 353 | 1.29497356114759e-10 | FAM83D | 758 | 0.981441060971783 |  |  |  |
| FAM83B | 354 | 1.73515013857524e-10 | FAM83D | 761 | 0.937683284890455 |  |  |  |
| FAM83B | 357 | 2.74055262686147e-10 | FAM83D | 761 | 0.889452438783548 |  |  |  |
| FAM83B | 358 | 3.99727613455939e-10 | FAM83D | 762 | 0.837125556541766 |  |  |  |
| FAM83B | 359 | 5.64162322744039e-10 | FAM83D | 763 | 0.783571373565389 |  |  |  |
| FAM83B | 362 | 9.12555078134978e-10 | FAM83D | 763 | 0.729129586764682 |  |  |  |
| FAM83B | 362 | 1.11221423020437e-09 | FAM83D | 766 | 0.827092401488897 |  |  |  |
| FAM83B | 368 | 1.60023980619686e-09 | FAM83D | 766 | 0.885412648142039 |  |  |  |
| FAM83B | 368 | 2.15282471542093e-09 | FAM83D | 768 | 0.836913061926821 |  |  |  |
| FAM83B | 369 | 3.16139967820104e-09 | FAM83D | 768 | 0.801909857524774 |  |  |  |
| FAM83B | 369 | 5.27900315091589e-09 | FAM83D | 783 | 0.890869964840081 |  |  |  |
| FAM83B | 371 | 9.10037704324862e-09 | FAM83D | 791 | 0.997636058035252 |  |  |  |
| FAM83B | 372 | 1.12980974979908e-08 | FAM83D | 791 | 0.906441911420114 |  |  |  |
| FAM83B | 374 | 1.58045917635055e-08 | FAM83D | 795 | 0.843527910020968 |  |  |  |
| FAM83B | 378 | 2.52723449872365e-08 | FAM83D | 797 | 0.736546815826469 |  |  |  |
| FAM83B | 380 | 1.76412155868194e-08 | FAM83D | 797 | 0.744163151750353 |  |  |  |
| FAM83B | 382 | 1.22187309825103e-08 | FAM83D | 798 | 0.797679173006557 |  |  |  |
| FAM83B | 383 | 1.58104249259408e-08 | FAM83D | 816 | 0.82555615061785 |  |  |  |
| FAM83B | 386 | 1.11409405835251e-08 | FAM83D | 817 | 0.878528792953916 |  |  |  |
| FAM83B | 386 | 1.91614471272576e-08 | FAM83D | 826 | 0.913277734272498 |  |  |  |
| FAM83B | 388 | 1.37697918817867e-08 | FAM83D | 831 | 0.818630853413319 |  |  |  |
| FAM83B | 398 | 1.46482093337771e-08 | FAM83D | 834 | 0.871043112952679 |  |  |  |
| FAM83B | 402 | 1.10979777021209e-08 | FAM83D | 839 | 0.932612178045842 |  |  |  |
| FAM83B | 403 | 6.24222051095957e-09 | FAM83D | 841 | 0.986820224379031 |  |  |  |
| FAM83B | 404 | 2.86996485258994e-09 | FAM83D | 842 | 0.955523899783602 |  |  |  |
| FAM83B | 404 | 5.04375990580092e-09 | FAM83D | 846 | 0.917256141899455 |  |  |  |
| FAM83B | 407 | 2.29720917326533e-09 | FAM83D | 852 | 0.865356363289045 |  |  |  |
| FAM83B | 409 | 2.79601451422177e-09 | FAM83D | 853 | 0.945878230353273 |  |  |  |
| FAM83B | 412 | 3.30794911616125e-09 | FAM83D | 854 | 0.916040536222992 |  |  |  |
| FAM83B | 413 | 2.36768704236071e-09 | FAM83D | 858 | 0.8538771698276 |  |  |  |
| FAM83B | 416 | 4.12733771292122e-09 | FAM83D | 860 | 0.798085703295042 |  |  |  |
| FAM83B | 417 | 5.85309454355658e-09 | FAM83D | 864 | 0.861910764644906 |  |  |  |
| FAM83B | 417 | 3.78356792830643e-09 | FAM83D | 866 | 0.811227094873509 |  |  |  |
| FAM83B | 419 | 6.14632679278566e-09 | FAM83D | 871 | 0.757376563739502 |  |  |  |
| FAM83B | 420 | 3.90712882805437e-09 | FAM83D | 901 | 0.704517431741827 |  |  |  |
| FAM83B | 426 | 4.6762704758632e-09 | FAM83D | 909 | 0.638097962819152 |  |  |  |
| FAM83B | 426 | 1.39475459141743e-09 | FAM83D | 910 | 0.588383648105004 |  |  |  |
| FAM83B | 428 | 8.67804789874531e-10 | FAM83D | 917 | 0.545714857811737 |  |  |  |
| FAM83B | 438 | 1.24050828711225e-09 | FAM83D | 922 | 0.636201208873024 |  |  |  |
| FAM83B | 438 | 7.80323728809972e-10 | FAM83D | 923 | 0.585007499737898 |  |  |  |
| FAM83B | 440 | 1.15153196638932e-09 | FAM83D | 926 | 0.523250672266721 |  |  |  |
| FAM83B | 440 | 1.70055471589926e-09 | FAM83D | 927 | 0.606931862813264 |  |  |  |
| FAM83B | 441 | 1.48463502556127e-09 | FAM83D | 928 | 0.568373992170526 |  |  |  |
| FAM83B | 441 | 2.63239860648639e-09 | FAM83D | 929 | 0.554188391195383 |  |  |  |
| FAM83B | 444 | 1.89216568868162e-09 | FAM83D | 930 | 0.510452644851349 |  |  |  |
| FAM83C | 10 | 0.30582766846948 | FAM83D | 936 | 0.580135485527953 |  |  |  |
| FAM83C | 10 | 0.345466304315423 | FAM83D | 939 | 0.528237023394555 |  |  |  |
| FAM83C | 10 | 0.322566189374036 | FAM83D | 942 | 0.508342805170679 |  |  |  |
| FAM83C | 10 | 0.362039633141234 | FAM83D | 942 | 0.602975971467034 |  |  |  |
| FAM83C | 10 | 0.314333342156614 | FAM83D | 944 | 0.568221310551971 |  |  |  |
| FAM83C | 10 | 0.351132448377072 | FAM83D | 945 | 0.626357684230928 |  |  |  |
| FAM83C | 10 | 0.337667040144602 | FAM83D | 947 | 0.577676129855201 |  |  |  |
| FAM83C | 10 | 0.292275752622615 | FAM83D | 949 | 0.676552782038114 |  |  |  |
| FAM83C | 10 | 0.269615404234895 | FAM83D | 957 | 0.786151019528555 |  |  |  |
| FAM83C | 10 | 0.307729561826635 | FAM83D | 961 | 0.727150800033295 |  |  |  |
| FAM83C | 10 | 0.280434719286605 | FAM83D | 966 | 0.712151088897976 |  |  |  |
| FAM83C | 10 | 0.30733423924108 | FAM83D | 969 | 0.670016820325103 |  |  |  |
| FAM83C | 10 | 0.303818930178222 | FAM83D | 970 | 0.629093564722775 |  |  |  |
| FAM83C | 10 | 0.342864704510508 | FAM83D | 972 | 0.574432525829034 |  |  |  |
| FAM83C | 10 | 0.391559568406654 | FAM83D | 973 | 0.524266299228427 |  |  |  |
| FAM83C | 10 | 0.322536944626076 | FAM83D | 989 | 0.611304107335732 |  |  |  |
| FAM83C | 10 | 0.272223915773336 | FAM83D | 991 | 0.558506959639626 |  |  |  |
| FAM83C | 10 | 0.309268739646986 | FAM83D | 993 | 0.513030088409332 |  |  |  |
| FAM83C | 10 | 0.269285114868998 | FAM83D | 995 | 0.586218464578641 |  |  |  |
| FAM83C | 10 | 0.246835864302379 | FAM83D | 995 | 0.690649433309611 |  |  |  |
| FAM83C | 11 | 0.227824315482812 | FAM83D | 996 | 0.641056048079592 |  |  |  |
| FAM83C | 11 | 0.2439507220105 | FAM83D | 100 | 0.586714469883925 |  |  |  |
| FAM83C | 11 | 0.222316723578091 | FAM83D | 100 | 0.535351046111691 |  |  |  |
| FAM83C | 11 | 0.206883648407673 | FAM83D | 100 | 0.51382806888141 |  |  |  |
| FAM83C | 11 | 0.206169402104765 | FAM83D | 101 | 0.566302329747124 |  |  |  |
| FAM83C | 11 | 0.225013935724145 | FAM83D | 101 | 0.537607203002208 |  |  |  |
| FAM83C | 11 | 0.249368966784924 | FAM83D | 101 | 0.537094776093251 |  |  |  |
| FAM83C | 11 | 0.266822488170971 | FAM83D | 101 | 0.499708645909048 |  |  |  |
| FAM83C | 11 | 0.298239385353135 | FAM83D | 101 | 0.456418883204363 |  |  |  |
| FAM83C | 11 | 0.251103146738257 | FAM83D | 102 | 0.416598261053736 |  |  |  |
| FAM83C | 11 | 0.242030398640946 | FAM83D | 103 | 0.50702309623757 |  |  |  |
| FAM83C | 11 | 0.223873596405517 | FAM83D | 104 | 0.456328666899453 |  |  |  |
| FAM83C | 11 | 0.249248025781753 | FAM83D | 104 | 0.414415564673197 |  |  |  |
| FAM83C | 11 | 0.220158257280708 | FAM83D | 105 | 0.471745454232782 |  |  |  |
| FAM83C | 11 | 0.196605416490843 | FAM83D | 105 | 0.558649125692915 |  |  |  |
| FAM83C | 12 | 0.185892260094356 | FAM83D | 105 | 0.513874457524506 |  |  |  |
| FAM83C | 12 | 0.160359157213835 | FAM83D | 106 | 0.474875373923306 |  |  |  |
| FAM83C | 12 | 0.160979316590842 | FAM83D | 106 | 0.415768031059269 |  |  |  |
| FAM83C | 12 | 0.1569607439784 | FAM83D | 108 | 0.486931655691098 |  |  |  |
| FAM83C | 12 | 0.14179409728775 | FAM83D | 109 | 0.557216614352376 |  |  |  |
| FAM83C | 12 | 0.120617842123609 | FAM83D | 109 | 0.619816427087243 |  |  |  |
| FAM83C | 12 | 0.127464667073097 | FAM83D | 110 | 0.684999623308173 |  |  |  |
| FAM83C | 12 | 0.126860285999866 | FAM83D | 110 | 0.636524435893022 |  |  |  |
| FAM83C | 12 | 0.113554837013466 | FAM83D | 111 | 0.589325754710234 |  |  |  |
| FAM83C | 12 | 0.0914625517983092 | FAM83D | 112 | 0.688706149630119 |  |  |  |
| FAM83C | 12 | 0.0771072420868715 | FAM83D | 113 | 0.666908648060047 |  |  |  |
| FAM83C | 12 | 0.0600546015516651 | FAM83D | 113 | 0.61009693202396 |  |  |  |
| FAM83C | 12 | 0.0716391235893654 | FAM83D | 113 | 0.687862981335741 |  |  |  |
| FAM83C | 12 | 0.0826225657942344 | FAM83D | 114 | 0.782448630884288 |  |  |  |
| FAM83C | 12 | 0.0698055606904611 | FAM83D | 114 | 0.719164339134298 |  |  |  |
| FAM83C | 12 | 0.0642271028830941 | FAM83E | 10 | 0.130960148705149 |  |  |  |
| FAM83C | 12 | 0.0590988494227169 | FAM83E | 10 | 0.115062249552953 |  |  |  |
| FAM83C | 12 | 0.0691700868395969 | FAM83E | 10 | 0.107955218650091 |  |  |  |
| FAM83C | 12 | 0.0649980594522112 | FAM83E | 10 | 0.0908998401321739 |  |  |  |
| FAM83C | 12 | 0.072238565728261 | FAM83E | 10 | 0.0824000193360439 |  |  |  |
| FAM83C | 13 | 0.0607238938464355 | FAM83E | 10 | 0.0728279392266898 |  |  |  |
| FAM83C | 13 | 0.0733768877842064 | FAM83E | 10 | 0.085316063293233 |  |  |  |
| FAM83C | 13 | 0.0889089747168342 | FAM83E | 10 | 0.105476977683 |  |  |  |
| FAM83C | 13 | 0.102843069137659 | FAM83E | 10 | 0.126901387260972 |  |  |  |
| FAM83C | 13 | 0.0905348591430509 | FAM83E | 10 | 0.111302542003799 |  |  |  |
| FAM83C | 13 | 0.101632965209601 | FAM83E | 10 | 0.0991844822403349 |  |  |  |
| FAM83C | 13 | 0.120891938732398 | FAM83E | 10 | 0.0887657259394133 |  |  |  |
| FAM83C | 13 | 0.108571770595848 | FAM83E | 10 | 0.105292616614531 |  |  |  |
| FAM83C | 13 | 0.09527838634038 | FAM83E | 10 | 0.0974194883591061 |  |  |  |
| FAM83C | 13 | 0.0893337516994319 | FAM83E | 10 | 0.0871777484487938 |  |  |  |
| FAM83C | 13 | 0.10205402101498 | FAM83E | 10 | 0.0739268474690586 |  |  |  |
| FAM83C | 13 | 0.118772382727692 | FAM83E | 10 | 0.0636663282532045 |  |  |  |
| FAM83C | 13 | 0.107145787381072 | FAM83E | 10 | 0.0560484990430493 |  |  |  |
| FAM83C | 13 | 0.124158585468788 | FAM83E | 10 | 0.0508949660503229 |  |  |  |
| FAM83C | 13 | 0.142412716110033 | FAM83E | 10 | 0.0611210909912142 |  |  |  |
| FAM83C | 13 | 0.164828184831361 | FAM83E | 10 | 0.0662720569304579 |  |  |  |
| FAM83C | 13 | 0.185377560015275 | FAM83E | 11 | 0.0575139252874263 |  |  |  |
| FAM83C | 13 | 0.206822387895532 | FAM83E | 11 | 0.0688539219965728 |  |  |  |
| FAM83C | 13 | 0.203275704806728 | FAM83E | 11 | 0.0606833329707423 |  |  |  |
| FAM83C | 13 | 0.234608128935355 | FAM83E | 11 | 0.0715331779048533 |  |  |  |
| FAM83C | 13 | 0.210023967391381 | FAM83E | 11 | 0.0615288390075147 |  |  |  |
| FAM83C | 13 | 0.241347417491441 | FAM83E | 11 | 0.0748386218042407 |  |  |  |
| FAM83C | 13 | 0.223233262777048 | FAM83E | 11 | 0.0657636242536239 |  |  |  |
| FAM83C | 13 | 0.211314687045076 | FAM83E | 11 | 0.061825023140153 |  |  |  |
| FAM83C | 13 | 0.193270174828302 | FAM83E | 11 | 0.0572603879155788 |  |  |  |
| FAM83C | 13 | 0.174597873581119 | FAM83E | 11 | 0.0506611342983655 |  |  |  |
| FAM83C | 14 | 0.158420641519555 | FAM83E | 11 | 0.0450575675281454 |  |  |  |
| FAM83C | 14 | 0.136807843290111 | FAM83E | 11 | 0.0565135961291532 |  |  |  |
| FAM83C | 14 | 0.153983848584457 | FAM83E | 11 | 0.0672552697774977 |  |  |  |
| FAM83C | 14 | 0.12436302693772 | FAM83E | 11 | 0.0582401991735987 |  |  |  |
| FAM83C | 14 | 0.13776552734294 | FAM83E | 11 | 0.0676664934509202 |  |  |  |
| FAM83C | 14 | 0.149734499861094 | FAM83E | 11 | 0.0764797450054676 |  |  |  |
| FAM83C | 14 | 0.133577349070379 | FAM83E | 11 | 0.0845239419397287 |  |  |  |
| FAM83C | 14 | 0.121075076048219 | FAM83E | 11 | 0.0738561735166084 |  |  |  |
| FAM83C | 14 | 0.136945254981305 | FAM83E | 11 | 0.0643371223375265 |  |  |  |
| FAM83C | 14 | 0.161105162179515 | FAM83E | 11 | 0.0563891482655245 |  |  |  |
| FAM83C | 14 | 0.184922296840715 | FAM83E | 11 | 0.0487325661532216 |  |  |  |
| FAM83C | 14 | 0.171285771236997 | FAM83E | 11 | 0.0403256751900592 |  |  |  |
| FAM83C | 14 | 0.167136813678102 | FAM83E | 11 | 0.0340822691025485 |  |  |  |
| FAM83C | 14 | 0.194580131659326 | FAM83E | 11 | 0.0279370909656314 |  |  |  |
| FAM83C | 14 | 0.198685136150799 | FAM83E | 11 | 0.026039925144529 |  |  |  |
| FAM83C | 14 | 0.184930902347595 | FAM83E | 11 | 0.0335295997455043 |  |  |  |
| FAM83C | 14 | 0.198191735224608 | FAM83E | 11 | 0.0310448557586096 |  |  |  |
| FAM83C | 14 | 0.178639869915334 | FAM83E | 11 | 0.0285358708060154 |  |  |  |
| FAM83C | 14 | 0.205766502315282 | FAM83E | 11 | 0.0361050583619756 |  |  |  |
| FAM83C | 14 | 0.191670549689095 | FAM83E | 12 | 0.0456906478356623 |  |  |  |
| FAM83C | 14 | 0.176047672647567 | FAM83E | 12 | 0.0573316707568057 |  |  |  |
| FAM83C | 15 | 0.150936713283211 | FAM83E | 12 | 0.047702660710215 |  |  |  |
| FAM83C | 15 | 0.126820831331218 | FAM83E | 12 | 0.0408358888316505 |  |  |  |
| FAM83C | 15 | 0.131010125857177 | FAM83E | 12 | 0.04485737827205 |  |  |  |
| FAM83C | 15 | 0.154443718179199 | FAM83E | 12 | 0.05626538405627 |  |  |  |
| FAM83C | 15 | 0.139141369307262 | FAM83E | 12 | 0.049573157274441 |  |  |  |
| FAM83C | 15 | 0.113802117023153 | FAM83E | 12 | 0.0625014683373141 |  |  |  |
| FAM83C | 15 | 0.100080867160497 | FAM83E | 12 | 0.0605830111501062 |  |  |  |
| FAM83C | 15 | 0.106084950882923 | FAM83E | 12 | 0.0531176993153403 |  |  |  |
| FAM83C | 15 | 0.09352016463691 | FAM83E | 12 | 0.0470514256870213 |  |  |  |
| FAM83C | 15 | 0.0840138700381656 | FAM83E | 12 | 0.0417013721314894 |  |  |  |
| FAM83C | 15 | 0.0759214442316698 | FAM83E | 12 | 0.0372921912013189 |  |  |  |
| FAM83C | 15 | 0.0913029348280767 | FAM83E | 12 | 0.0327714413373888 |  |  |  |
| FAM83C | 15 | 0.103425545400891 | FAM83E | 12 | 0.0288680730760445 |  |  |  |
| FAM83C | 15 | 0.0932378041781654 | FAM83E | 12 | 0.0362805629730277 |  |  |  |
| FAM83C | 15 | 0.0840520128510921 | FAM83E | 12 | 0.0463606479418416 |  |  |  |
| FAM83C | 15 | 0.0980940003962009 | FAM83E | 12 | 0.0520660992974401 |  |  |  |
| FAM83C | 15 | 0.114283731554697 | FAM83E | 12 | 0.0455369774546392 |  |  |  |
| FAM83C | 15 | 0.128979440842262 | FAM83E | 12 | 0.0561594083490895 |  |  |  |
| FAM83C | 15 | 0.15150101727839 | FAM83E | 12 | 0.0659694743567144 |  |  |  |
| FAM83C | 15 | 0.172916191412415 | FAM83E | 12 | 0.0612976576623471 |  |  |  |
| FAM83C | 15 | 0.197980557183195 | FAM83E | 12 | 0.0541531082472142 |  |  |  |
| FAM83C | 15 | 0.218730103768226 | FAM83E | 12 | 0.0644867762247957 |  |  |  |
| FAM83C | 15 | 0.175876795535571 | FAM83E | 12 | 0.0772180689682348 |  |  |  |
| FAM83C | 15 | 0.179217596391525 | FAM83E | 12 | 0.0867508924899781 |  |  |  |
| FAM83C | 15 | 0.163540269780303 | FAM83E | 12 | 0.1062348217552 |  |  |  |
| FAM83C | 15 | 0.148382395416412 | FAM83E | 12 | 0.128836219158875 |  |  |  |
| FAM83C | 15 | 0.134335168945249 | FAM83E | 12 | 0.140851826844736 |  |  |  |
| FAM83C | 15 | 0.127011642878844 | FAM83E | 12 | 0.170708271316389 |  |  |  |
| FAM83C | 15 | 0.114596185272256 | FAM83E | 12 | 0.202466738639818 |  |  |  |
| FAM83C | 15 | 0.102391302882411 | FAM83E | 12 | 0.175875483803203 |  |  |  |
| FAM83C | 15 | 0.0888195554387783 | FAM83E | 12 | 0.156388653545039 |  |  |  |
| FAM83C | 15 | 0.0822704264119706 | FAM83E | 12 | 0.139170636071232 |  |  |  |
| FAM83C | 16 | 0.0758196769648814 | FAM83E | 13 | 0.119371683064181 |  |  |  |
| FAM83C | 16 | 0.0902995484046215 | FAM83E | 13 | 0.145023617300638 |  |  |  |
| FAM83C | 16 | 0.0726556800441179 | FAM83E | 13 | 0.127194632928152 |  |  |  |
| FAM83C | 16 | 0.0610035319107535 | FAM83E | 13 | 0.112615774573273 |  |  |  |
| FAM83C | 16 | 0.0729761186086942 | FAM83E | 13 | 0.0995648442591492 |  |  |  |
| FAM83C | 16 | 0.0624243862306994 | FAM83E | 13 | 0.0893134979023015 |  |  |  |
| FAM83C | 16 | 0.070467388179327 | FAM83E | 13 | 0.0843234482658699 |  |  |  |
| FAM83C | 16 | 0.0645480979714974 | FAM83E | 13 | 0.0741024011084622 |  |  |  |
| FAM83C | 16 | 0.0758105947862543 | FAM83E | 13 | 0.065910302456247 |  |  |  |
| FAM83C | 16 | 0.0715557649616491 | FAM83E | 13 | 0.0802881266362033 |  |  |  |
| FAM83C | 16 | 0.0634515472596764 | FAM83E | 13 | 0.099295917061062 |  |  |  |
| FAM83C | 16 | 0.0550233092497009 | FAM83E | 13 | 0.113432283280284 |  |  |  |
| FAM83C | 16 | 0.0454662690245583 | FAM83E | 13 | 0.101323215427316 |  |  |  |
| FAM83C | 16 | 0.0397894071708538 | FAM83E | 13 | 0.0880433027149014 |  |  |  |
| FAM83C | 16 | 0.0465110557752503 | FAM83E | 13 | 0.100327772301162 |  |  |  |
| FAM83C | 16 | 0.0411424974871901 | FAM83E | 13 | 0.0881976039839742 |  |  |  |
| FAM83C | 16 | 0.0354353843433557 | FAM83E | 13 | 0.0755044813204238 |  |  |  |
| FAM83C | 16 | 0.0324015755812489 | FAM83E | 13 | 0.0655854908401771 |  |  |  |
| FAM83C | 16 | 0.0285862347517816 | FAM83E | 13 | 0.0782805545506116 |  |  |  |
| FAM83C | 17 | 0.0250667176557463 | FAM83E | 13 | 0.0966166213193813 |  |  |  |
| FAM83C | 17 | 0.0305661992433131 | FAM83E | 13 | 0.11108053456338 |  |  |  |
| FAM83C | 17 | 0.0350338771430958 | FAM83E | 13 | 0.1305367054418 |  |  |  |
| FAM83C | 17 | 0.039794491569069 | FAM83E | 13 | 0.121882311195588 |  |  |  |
| FAM83C | 17 | 0.0431432740673209 | FAM83E | 13 | 0.104269127036758 |  |  |  |
| FAM83C | 17 | 0.0493507195486933 | FAM83E | 13 | 0.122165277045019 |  |  |  |
| FAM83C | 17 | 0.0490939429591821 | FAM83E | 14 | 0.110373930934262 |  |  |  |
| FAM83C | 17 | 0.0457943339193821 | FAM83E | 14 | 0.0960062299147514 |  |  |  |
| FAM83C | 17 | 0.0452213260146872 | FAM83E | 14 | 0.0833757909358559 |  |  |  |
| FAM83C | 17 | 0.0401194117406663 | FAM83E | 14 | 0.0740797587919233 |  |  |  |
| FAM83C | 17 | 0.0334402635993277 | FAM83E | 14 | 0.0658141946379982 |  |  |  |
| FAM83C | 17 | 0.0276895979703332 | FAM83E | 14 | 0.0574432625624561 |  |  |  |
| FAM83C | 17 | 0.0328174256581457 | FAM83E | 14 | 0.0506186053846831 |  |  |  |
| FAM83C | 17 | 0.0289966746306208 | FAM83E | 14 | 0.0446348104988983 |  |  |  |
| FAM83C | 17 | 0.0261625615096938 | FAM83E | 14 | 0.0393589018693519 |  |  |  |
| FAM83C | 17 | 0.0248882816076514 | FAM83E | 14 | 0.0477070103504416 |  |  |  |
| FAM83C | 17 | 0.0220918279858519 | FAM83E | 14 | 0.0566660909309668 |  |  |  |
| FAM83C | 17 | 0.019513423420507 | FAM83E | 14 | 0.0645941094351996 |  |  |  |
| FAM83C | 17 | 0.0160844619431432 | FAM83E | 14 | 0.0633403379858899 |  |  |  |
| FAM83C | 17 | 0.0189744437830127 | FAM83E | 14 | 0.0561777808042197 |  |  |  |
| FAM83C | 17 | 0.0159738421331351 | FAM83E | 14 | 0.0673931121489557 |  |  |  |
| FAM83C | 17 | 0.0138738599466631 | FAM83E | 14 | 0.0837421491080195 |  |  |  |
| FAM83C | 17 | 0.0121628402197863 | FAM83E | 14 | 0.0730158617570827 |  |  |  |
| FAM83C | 18 | 0.0106627143169419 | FAM83E | 14 | 0.0637839764776747 |  |  |  |
| FAM83C | 18 | 0.0132736657513132 | FAM83E | 14 | 0.0607518248365918 |  |  |  |
| FAM83C | 18 | 0.0160247448089727 | FAM83E | 14 | 0.0704066571843456 |  |  |  |
| FAM83C | 18 | 0.0200137839569 | FAM83E | 14 | 0.0870814522786727 |  |  |  |
| FAM83C | 18 | 0.0247601574092833 | FAM83E | 14 | 0.0766164697503743 |  |  |  |
| FAM83C | 18 | 0.0256708855512669 | FAM83E | 15 | 0.0887394140215792 |  |  |  |
| FAM83C | 18 | 0.0259743852649922 | FAM83E | 15 | 0.0752720935941706 |  |  |  |
| FAM83C | 18 | 0.0217913614815179 | FAM83E | 15 | 0.0648732450104697 |  |  |  |
| FAM83C | 18 | 0.0249849216743557 | FAM83E | 15 | 0.0565936436256179 |  |  |  |
| FAM83C | 18 | 0.0213684845901271 | FAM83E | 15 | 0.0681449903243262 |  |  |  |
| FAM83C | 18 | 0.0250782398348844 | FAM83E | 15 | 0.0595769903012474 |  |  |  |
| FAM83C | 18 | 0.0220966883639543 | FAM83E | 15 | 0.0722227568323665 |  |  |  |
| FAM83C | 18 | 0.0216182315233214 | FAM83E | 15 | 0.0869596474734503 |  |  |  |
| FAM83C | 18 | 0.0254044338011675 | FAM83E | 15 | 0.100161652427148 |  |  |  |
| FAM83C | 18 | 0.0310139490761524 | FAM83E | 15 | 0.0884548614342018 |  |  |  |
| FAM83C | 18 | 0.0379799295741758 | FAM83E | 15 | 0.0758939072088977 |  |  |  |
| FAM83C | 18 | 0.0326132234386718 | FAM83E | 15 | 0.0663983650125816 |  |  |  |
| FAM83C | 18 | 0.0394573341665269 | FAM83E | 15 | 0.0581720567687141 |  |  |  |
| FAM83C | 18 | 0.0342495839470487 | FAM83E | 15 | 0.0499433278438616 |  |  |  |
| FAM83C | 18 | 0.030072425766087 | FAM83E | 15 | 0.0456082893392382 |  |  |  |
| FAM83C | 18 | 0.0260850277523515 | FAM83E | 15 | 0.0557850799808476 |  |  |  |
| FAM83C | 18 | 0.0230221162607701 | FAM83E | 15 | 0.0486126072794853 |  |  |  |
| FAM83C | 18 | 0.0194181611045412 | FAM83E | 15 | 0.0588670005573008 |  |  |  |
| FAM83C | 18 | 0.017271498036122 | FAM83E | 15 | 0.0678683118020706 |  |  |  |
| FAM83C | 18 | 0.014707325359496 | FAM83E | 15 | 0.0756471930383808 |  |  |  |
| FAM83C | 18 | 0.0128133934920117 | FAM83E | 15 | 0.0717426400849053 |  |  |  |
| FAM83C | 19 | 0.0109393694342688 | FAM83E | 15 | 0.0669212829193185 |  |  |  |
| FAM83C | 19 | 0.0125105910041104 | FAM83E | 16 | 0.0585645999487872 |  |  |  |
| FAM83C | 19 | 0.0144658922547798 | FAM83E | 16 | 0.0509458402818684 |  |  |  |
| FAM83C | 19 | 0.0112157627849273 | FAM83E | 16 | 0.0448104773452602 |  |  |  |
| FAM83C | 19 | 0.00972377207746159 | FAM83E | 16 | 0.039737433709853 |  |  |  |
| FAM83C | 19 | 0.00843166806181745 | FAM83E | 16 | 0.0349407770349434 |  |  |  |
| FAM83C | 19 | 0.00729125007439895 | FAM83E | 16 | 0.0425427887874507 |  |  |  |
| FAM83C | 19 | 0.00917998335421977 | FAM83E | 16 | 0.0528831076284308 |  |  |  |
| FAM83C | 19 | 0.0107006562405164 | FAM83E | 16 | 0.04604228607694 |  |  |  |
| FAM83C | 19 | 0.00923465165120493 | FAM83E | 16 | 0.0399277281107034 |  |  |  |
| FAM83C | 19 | 0.0114975775983369 | FAM83E | 16 | 0.049986878697746 |  |  |  |
| FAM83C | 19 | 0.0104698312000772 | FAM83E | 16 | 0.0566159654107011 |  |  |  |
| FAM83C | 19 | 0.0120961788587735 | FAM83E | 16 | 0.051401748356464 |  |  |  |
| FAM83C | 19 | 0.010546383771134 | FAM83E | 17 | 0.0476313115352721 |  |  |  |
| FAM83C | 19 | 0.0088405515028921 | FAM83E | 17 | 0.0473960514615641 |  |  |  |
| FAM83C | 19 | 0.0106064647322667 | FAM83E | 17 | 0.0401662032806603 |  |  |  |
| FAM83C | 19 | 0.0120862960588397 | FAM83E | 17 | 0.0337630190206007 |  |  |  |
| FAM83C | 19 | 0.0121309692959821 | FAM83E | 17 | 0.0293339318621162 |  |  |  |
| FAM83C | 19 | 0.0105294483424067 | FAM83E | 17 | 0.0256360196160081 |  |  |  |
| FAM83C | 19 | 0.00854221134225678 | FAM83E | 17 | 0.0226307266772994 |  |  |  |
| FAM83C | 19 | 0.0090752089286307 | FAM83E | 17 | 0.0274409079813609 |  |  |  |
| FAM83C | 19 | 0.00779921146350702 | FAM83E | 17 | 0.0333298874487259 |  |  |  |
| FAM83C | 19 | 0.0070375918282726 | FAM83E | 17 | 0.037827010516332 |  |  |  |
| FAM83C | 19 | 0.00603258121590267 | FAM83E | 17 | 0.0473798754832626 |  |  |  |
| FAM83C | 19 | 0.00518323890617744 | FAM83E | 17 | 0.0593985036493736 |  |  |  |
| FAM83C | 20 | 0.00444344923703599 | FAM83E | 18 | 0.0715596467304112 |  |  |  |
| FAM83C | 20 | 0.00462299547595907 | FAM83E | 18 | 0.0827808146918576 |  |  |  |
| FAM83C | 20 | 0.00579712979796197 | FAM83E | 18 | 0.0740338160019206 |  |  |  |
| FAM83C | 20 | 0.00647244857093698 | FAM83E | 18 | 0.087077510015776 |  |  |  |
| FAM83C | 20 | 0.00611359727221227 | FAM83E | 18 | 0.105327308255145 |  |  |  |
| FAM83C | 20 | 0.00745625312767056 | FAM83E | 18 | 0.124205018264036 |  |  |  |
| FAM83C | 20 | 0.00883111855823632 | FAM83E | 18 | 0.139447722848424 |  |  |  |
| FAM83C | 20 | 0.00819885079415623 | FAM83E | 18 | 0.157491517480048 |  |  |  |
| FAM83C | 20 | 0.00701240726673702 | FAM83E | 19 | 0.144207515028315 |  |  |  |
| FAM83C | 20 | 0.00581660559199446 | FAM83E | 19 | 0.124438841053853 |  |  |  |
| FAM83C | 20 | 0.00431435220022171 | FAM83E | 19 | 0.10796616258843 |  |  |  |
| FAM83C | 20 | 0.00521086763357863 | FAM83E | 19 | 0.0970152569050757 |  |  |  |
| FAM83C | 20 | 0.00433879766922263 | FAM83E | 19 | 0.106843773986479 |  |  |  |
| FAM83C | 20 | 0.00369079478779158 | FAM83E | 19 | 0.0928443077935615 |  |  |  |
| FAM83C | 20 | 0.00328688618535798 | FAM83E | 19 | 0.111247303340057 |  |  |  |
| FAM83C | 20 | 0.00269187907491455 | FAM83E | 19 | 0.13342511056564 |  |  |  |
| FAM83C | 20 | 0.0022604162129046 | FAM83E | 19 | 0.156562327468 |  |  |  |
| FAM83C | 20 | 0.00206718525377089 | FAM83E | 19 | 0.136816401467312 |  |  |  |
| FAM83C | 20 | 0.00256346262485686 | FAM83E | 19 | 0.121035770816078 |  |  |  |
| FAM83C | 20 | 0.00217468573887817 | FAM83E | 19 | 0.112770546725328 |  |  |  |
| FAM83C | 20 | 0.0018898530740047 | FAM83E | 20 | 0.125310522953643 |  |  |  |
| FAM83C | 20 | 0.00160182397489251 | FAM83E | 20 | 0.140503392371106 |  |  |  |
| FAM83C | 21 | 0.00129359171181102 | FAM83E | 20 | 0.151320312313624 |  |  |  |
| FAM83C | 21 | 0.00150694737544285 | FAM83E | 20 | 0.136821292237469 |  |  |  |
| FAM83C | 21 | 0.00175715510570531 | FAM83E | 20 | 0.1609865524739 |  |  |  |
| FAM83C | 21 | 0.00220848449523023 | FAM83E | 20 | 0.140815385681649 |  |  |  |
| FAM83C | 21 | 0.00282513038031762 | FAM83E | 20 | 0.165977592275363 |  |  |  |
| FAM83C | 21 | 0.00356746539514161 | FAM83E | 20 | 0.187731350468143 |  |  |  |
| FAM83C | 21 | 0.0030247067566075 | FAM83E | 20 | 0.209535964076956 |  |  |  |
| FAM83C | 21 | 0.0035279572797976 | FAM83E | 20 | 0.184878239943213 |  |  |  |
| FAM83C | 21 | 0.00422068161013253 | FAM83E | 20 | 0.164006597399549 |  |  |  |
| FAM83C | 21 | 0.00524282189650164 | FAM83E | 21 | 0.153757664087351 |  |  |  |
| FAM83C | 21 | 0.0047199184905874 | FAM83E | 21 | 0.175247474150691 |  |  |  |
| FAM83C | 21 | 0.00282946146498721 | FAM83E | 21 | 0.187553008223881 |  |  |  |
| FAM83C | 21 | 0.00351530053629328 | FAM83E | 21 | 0.202259705728436 |  |  |  |
| FAM83C | 21 | 0.00427226836837197 | FAM83E | 21 | 0.181989239514347 |  |  |  |
| FAM83C | 21 | 0.00540602048747827 | FAM83E | 21 | 0.165512119220736 |  |  |  |
| FAM83C | 21 | 0.0068262318858775 | FAM83E | 21 | 0.182022538641624 |  |  |  |
| FAM83C | 21 | 0.00777972179826846 | FAM83E | 21 | 0.206423671135278 |  |  |  |
| FAM83C | 21 | 0.00670571216815187 | FAM83E | 21 | 0.188062535511474 |  |  |  |
| FAM83C | 21 | 0.00577386826753608 | FAM83E | 21 | 0.166917593201212 |  |  |  |
| FAM83C | 21 | 0.00709901239230612 | FAM83E | 21 | 0.15287464391803 |  |  |  |
| FAM83C | 21 | 0.0064291752694685 | FAM83E | 22 | 0.165617053613075 |  |  |  |
| FAM83C | 21 | 0.00725889573671383 | FAM83E | 22 | 0.192254597568986 |  |  |  |
| FAM83C | 21 | 0.00631800933177676 | FAM83E | 22 | 0.201991435849338 |  |  |  |
| FAM83C | 21 | 0.00560797327736042 | FAM83E | 22 | 0.222006700061462 |  |  |  |
| FAM83C | 22 | 0.00480565905501715 | FAM83E | 22 | 0.248443416797962 |  |  |  |
| FAM83C | 22 | 0.00617261574924779 | FAM83E | 22 | 0.226478039241397 |  |  |  |
| FAM83C | 22 | 0.00675168899411671 | FAM83E | 22 | 0.203292106958822 |  |  |  |
| FAM83C | 22 | 0.00465322934071905 | FAM83E | 22 | 0.182792897509978 |  |  |  |
| FAM83C | 22 | 0.00533509051692867 | FAM83E | 22 | 0.191596726768466 |  |  |  |
| FAM83C | 22 | 0.00457631701208984 | FAM83E | 22 | 0.173442692688938 |  |  |  |
| FAM83C | 22 | 0.00510325659172741 | FAM83E | 22 | 0.16052846561783 |  |  |  |
| FAM83C | 22 | 0.00416746122714205 | FAM83E | 22 | 0.153555987610646 |  |  |  |
| FAM83C | 22 | 0.00449820773291557 | FAM83E | 23 | 0.177989602055692 |  |  |  |
| FAM83C | 22 | 0.00366363276420688 | FAM83E | 23 | 0.20348677249993 |  |  |  |
| FAM83C | 22 | 0.00446664566253912 | FAM83E | 23 | 0.236587142876946 |  |  |  |
| FAM83C | 22 | 0.00532326347615653 | FAM83E | 23 | 0.262105659293073 |  |  |  |
| FAM83C | 22 | 0.00684453937779047 | FAM83E | 23 | 0.288378868398745 |  |  |  |
| FAM83C | 22 | 0.00789505405909615 | FAM83E | 23 | 0.2666945343857 |  |  |  |
| FAM83C | 22 | 0.00731867540888704 | FAM83E | 23 | 0.244138055629042 |  |  |  |
| FAM83C | 22 | 0.00661747771853022 | FAM83E | 23 | 0.264633618623804 |  |  |  |
| FAM83C | 22 | 0.00547436924348731 | FAM83E | 23 | 0.24793068954076 |  |  |  |
| FAM83C | 22 | 0.00545826530590461 | FAM83E | 23 | 0.247441557466519 |  |  |  |
| FAM83C | 22 | 0.00675424789290392 | FAM83E | 23 | 0.287775478728843 |  |  |  |
| FAM83C | 22 | 0.00857609745379179 | FAM83E | 23 | 0.326171718084881 |  |  |  |
| FAM83C | 22 | 0.00796434397227247 | FAM83E | 24 | 0.371242380362362 |  |  |  |
| FAM83C | 22 | 0.00990293689577346 | FAM83E | 24 | 0.33184614930147 |  |  |  |
| FAM83C | 22 | 0.0112003249076768 | FAM83E | 24 | 0.355677635791518 |  |  |  |
| FAM83C | 22 | 0.00978393605304093 | FAM83E | 24 | 0.342866700772463 |  |  |  |
| FAM83C | 22 | 0.0117879520679278 | FAM83E | 24 | 0.366698424562418 |  |  |  |
| FAM83C | 22 | 0.0105859372219034 | FAM83E | 24 | 0.391011092067111 |  |  |  |
| FAM83C | 22 | 0.0091755872443522 | FAM83E | 24 | 0.439295809026294 |  |  |  |
| FAM83C | 22 | 0.00798202948271422 | FAM83E | 24 | 0.496983305236674 |  |  |  |
| FAM83C | 22 | 0.00687482438194923 | FAM83E | 24 | 0.496225265033202 |  |  |  |
| FAM83C | 23 | 0.00583361508870784 | FAM83E | 24 | 0.536995734855931 |  |  |  |
| FAM83C | 23 | 0.00729826936544515 | FAM83E | 24 | 0.599216451061287 |  |  |  |
| FAM83C | 23 | 0.00910246063570726 | FAM83E | 24 | 0.659123409696947 |  |  |  |
| FAM83C | 23 | 0.0103940797547184 | FAM83E | 25 | 0.616229786991254 |  |  |  |
| FAM83C | 23 | 0.0116475171373043 | FAM83E | 25 | 0.57270913387635 |  |  |  |
| FAM83C | 23 | 0.0131156047132 | FAM83E | 25 | 0.538599667288544 |  |  |  |
| FAM83C | 23 | 0.0117900532735866 | FAM83E | 25 | 0.500171155038668 |  |  |  |
| FAM83C | 23 | 0.00994435338179198 | FAM83E | 25 | 0.522754212171882 |  |  |  |
| FAM83C | 23 | 0.0125774661625976 | FAM83E | 25 | 0.548946878111021 |  |  |  |
| FAM83C | 23 | 0.0146038037071092 | FAM83E | 25 | 0.523176807184144 |  |  |  |
| FAM83C | 23 | 0.0183212512318901 | FAM83E | 25 | 0.493145278224511 |  |  |  |
| FAM83C | 23 | 0.0161481025548357 | FAM83E | 25 | 0.540182376187819 |  |  |  |
| FAM83C | 23 | 0.0133326540836471 | FAM83E | 25 | 0.593440555084666 |  |  |  |
| FAM83C | 23 | 0.0155746026458824 | FAM83E | 25 | 0.653136013394725 |  |  |  |
| FAM83C | 23 | 0.0146579651256215 | FAM83E | 25 | 0.70561016353062 |  |  |  |
| FAM83C | 23 | 0.0129431486867299 | FAM83E | 26 | 0.663717509843999 |  |  |  |
| FAM83C | 23 | 0.0139344235315707 | FAM83E | 26 | 0.692608302491338 |  |  |  |
| FAM83C | 23 | 0.0121350820007892 | FAM83E | 26 | 0.649349318853765 |  |  |  |
| FAM83C | 23 | 0.0136331332657256 | FAM83E | 26 | 0.606326449299662 |  |  |  |
| FAM83C | 23 | 0.0114521301699883 | FAM83E | 26 | 0.605488537809822 |  |  |  |
| FAM83C | 23 | 0.00995070611152278 | FAM83E | 27 | 0.674723436506271 |  |  |  |
| FAM83C | 24 | 0.008284325422423 | FAM83E | 27 | 0.624566343016227 |  |  |  |
| FAM83C | 24 | 0.00686200756016724 | FAM83E | 27 | 0.591747930691929 |  |  |  |
| FAM83C | 24 | 0.00818240693906499 | FAM83E | 27 | 0.548947992009482 |  |  |  |
| FAM83C | 24 | 0.0102392410655316 | FAM83E | 27 | 0.509203542397585 |  |  |  |
| FAM83C | 24 | 0.0125054791891948 | FAM83E | 27 | 0.471797107064292 |  |  |  |
| FAM83C | 24 | 0.0139840751674169 | FAM83E | 27 | 0.491306251892177 |  |  |  |
| FAM83C | 24 | 0.0174195352156538 | FAM83E | 28 | 0.529696287052119 |  |  |  |
| FAM83C | 24 | 0.0192621125141655 | FAM83E | 28 | 0.489936223507509 |  |  |  |
| FAM83C | 24 | 0.0157104262612053 | FAM83E | 28 | 0.454086167162362 |  |  |  |
| FAM83C | 24 | 0.0182731509412106 | FAM83E | 28 | 0.505822374379454 |  |  |  |
| FAM83C | 24 | 0.0146634526030037 | FAM83E | 28 | 0.469910990126501 |  |  |  |
| FAM83C | 24 | 0.0173731272556324 | FAM83E | 28 | 0.437638348368018 |  |  |  |
| FAM83C | 24 | 0.0146852462204988 | FAM83E | 28 | 0.47498918510185 |  |  |  |
| FAM83C | 24 | 0.0124199322496059 | FAM83E | 28 | 0.428853639707857 |  |  |  |
| FAM83C | 24 | 0.0143140872822071 | FAM83E | 28 | 0.389115422810627 |  |  |  |
| FAM83C | 24 | 0.0124233066559188 | FAM83E | 28 | 0.438602713964287 |  |  |  |
| FAM83C | 24 | 0.00992823118308521 | FAM83E | 28 | 0.410553481211031 |  |  |  |
| FAM83C | 24 | 0.00857358098965996 | FAM83E | 29 | 0.456881439516724 |  |  |  |
| FAM83C | 25 | 0.00719369785519473 | FAM83E | 29 | 0.502906730072818 |  |  |  |
| FAM83C | 25 | 0.00873904521513899 | FAM83E | 29 | 0.465108905288285 |  |  |  |
| FAM83C | 25 | 0.0064479009793546 | FAM83E | 29 | 0.432157750482361 |  |  |  |
| FAM83C | 25 | 0.00470824693604941 | FAM83E | 29 | 0.483989213555506 |  |  |  |
| FAM83C | 25 | 0.00600400891577142 | FAM83E | 29 | 0.44667672469567 |  |  |  |
| FAM83C | 25 | 0.00643860894605304 | FAM83E | 29 | 0.414777325406278 |  |  |  |
| FAM83C | 25 | 0.00560846494857457 | FAM83E | 29 | 0.402321963383433 |  |  |  |
| FAM83C | 25 | 0.00600979305103474 | FAM83E | 29 | 0.360423716435529 |  |  |  |
| FAM83C | 25 | 0.00729728137515536 | FAM83E | 29 | 0.334513545337158 |  |  |  |
| FAM83C | 25 | 0.00704018089806794 | FAM83E | 30 | 0.355626211958037 |  |  |  |
| FAM83C | 25 | 0.00828009942484421 | FAM83E | 30 | 0.37159830925248 |  |  |  |
| FAM83C | 25 | 0.0101323365663151 | FAM83E | 30 | 0.336895706471505 |  |  |  |
| FAM83C | 25 | 0.0124943887875698 | FAM83E | 30 | 0.304317161787671 |  |  |  |
| FAM83C | 25 | 0.012389891638907 | FAM83E | 30 | 0.275272087985246 |  |  |  |
| FAM83C | 25 | 0.0152066779883105 | FAM83E | 30 | 0.249116752619211 |  |  |  |
| FAM83C | 25 | 0.0187115223120637 | FAM83E | 30 | 0.231749403722508 |  |  |  |
| FAM83C | 25 | 0.0219788078416451 | FAM83E | 30 | 0.252509157293061 |  |  |  |
| FAM83C | 25 | 0.0270127740820069 | FAM83E | 31 | 0.250091016716072 |  |  |  |
| FAM83C | 25 | 0.0236687180100367 | FAM83E | 31 | 0.225612465631772 |  |  |  |
| FAM83C | 25 | 0.0208648382085921 | FAM83E | 31 | 0.263124469001474 |  |  |  |
| FAM83C | 25 | 0.0235617558799459 | FAM83E | 31 | 0.292593770743931 |  |  |  |
| FAM83C | 25 | 0.0206014546435536 | FAM83E | 31 | 0.272974776128424 |  |  |  |
| FAM83C | 25 | 0.0184554827650898 | FAM83E | 31 | 0.298580407210354 |  |  |  |
| FAM83C | 26 | 0.0157539223009123 | FAM83E | 31 | 0.27633843951446 |  |  |  |
| FAM83C | 26 | 0.0195837118802215 | FAM83E | 31 | 0.311533596713573 |  |  |  |
| FAM83C | 26 | 0.0192756325576967 | FAM83E | 31 | 0.296943505313135 |  |  |  |
| FAM83C | 26 | 0.0163891402295013 | FAM83E | 31 | 0.34235789501313 |  |  |  |
| FAM83C | 26 | 0.0140448438065627 | FAM83E | 31 | 0.362653280336802 |  |  |  |
| FAM83C | 26 | 0.0161257734273219 | FAM83E | 31 | 0.40964710215406 |  |  |  |
| FAM83C | 26 | 0.0144970009636039 | FAM83E | 31 | 0.366935918594782 |  |  |  |
| FAM83C | 26 | 0.013167906391414 | FAM83E | 31 | 0.348416782164687 |  |  |  |
| FAM83C | 26 | 0.0119637698876379 | FAM83E | 31 | 0.320799282972586 |  |  |  |
| FAM83C | 26 | 0.0125787370793838 | FAM83E | 32 | 0.293688455836882 |  |  |  |
| FAM83C | 26 | 0.014750203490035 | FAM83E | 32 | 0.330790735524405 |  |  |  |
| FAM83C | 26 | 0.01445217005951 | FAM83E | 32 | 0.297860061026397 |  |  |  |
| FAM83C | 26 | 0.0175502456442071 | FAM83E | 32 | 0.332756836170214 |  |  |  |
| FAM83C | 26 | 0.0220170612594418 | FAM83E | 32 | 0.36132714453644 |  |  |  |
| FAM83C | 26 | 0.0253990683374638 | FAM83E | 32 | 0.340686271331167 |  |  |  |
| FAM83C | 26 | 0.0279483734318348 | FAM83E | 32 | 0.31194647699745 |  |  |  |
| FAM83C | 26 | 0.0303834914891246 | FAM83E | 32 | 0.350590363707261 |  |  |  |
| FAM83C | 26 | 0.0368646988414938 | FAM83E | 33 | 0.402963014603637 |  |  |  |
| FAM83C | 26 | 0.0321876064761995 | FAM83E | 33 | 0.454059510623334 |  |  |  |
| FAM83C | 26 | 0.028368135845095 | FAM83E | 33 | 0.413791697756489 |  |  |  |
| FAM83C | 26 | 0.0265514254747307 | FAM83E | 33 | 0.376694797760639 |  |  |  |
| FAM83C | 26 | 0.0237261896824974 | FAM83E | 33 | 0.346763446716342 |  |  |  |
| FAM83C | 26 | 0.0207919518451492 | FAM83E | 33 | 0.395831908274275 |  |  |  |
| FAM83C | 26 | 0.0181384078807464 | FAM83E | 33 | 0.360917926357245 |  |  |  |
| FAM83C | 26 | 0.0154744067290796 | FAM83E | 33 | 0.333133870194756 |  |  |  |
| FAM83C | 27 | 0.013334279332445 | FAM83E | 34 | 0.303171900657375 |  |  |  |
| FAM83C | 27 | 0.0162588936603231 | FAM83E | 34 | 0.348110882070849 |  |  |  |
| FAM83C | 27 | 0.014254430456591 | FAM83E | 34 | 0.321050521828904 |  |  |  |
| FAM83C | 27 | 0.012753277149747 | FAM83E | 34 | 0.291021945681187 |  |  |  |
| FAM83C | 27 | 0.0111942925780658 | FAM83E | 34 | 0.263396654950108 |  |  |  |
| FAM83C | 27 | 0.00993675831122613 | FAM83E | 35 | 0.303964123277383 |  |  |  |
| FAM83C | 27 | 0.0124989667705053 | FAM83E | 35 | 0.343243178204415 |  |  |  |
| FAM83C | 27 | 0.015734123685406 | FAM83E | 35 | 0.376350422895884 |  |  |  |
| FAM83C | 27 | 0.0194909291558317 | FAM83E | 35 | 0.356036872037829 |  |  |  |
| FAM83C | 27 | 0.0165367366205809 | FAM83E | 36 | 0.399418489092726 |  |  |  |
| FAM83C | 27 | 0.0148996304333409 | FAM83E | 36 | 0.454005916571457 |  |  |  |
| FAM83C | 27 | 0.0127344949111329 | FAM83E | 36 | 0.512573549849719 |  |  |  |
| FAM83C | 27 | 0.0114988596881947 | FAM83E | 36 | 0.469122066980827 |  |  |  |
| FAM83C | 27 | 0.0101407411749882 | FAM83E | 36 | 0.431906880381414 |  |  |  |
| FAM83C | 27 | 0.00871060511667837 | FAM83E | 36 | 0.464510038408257 |  |  |  |
| FAM83C | 28 | 0.00742626897326905 | FAM83E | 36 | 0.513152510332543 |  |  |  |
| FAM83C | 28 | 0.00504806797642621 | FAM83E | 36 | 0.567904599525205 |  |  |  |
| FAM83C | 28 | 0.00623939755916441 | FAM83E | 36 | 0.635593646517357 |  |  |  |
| FAM83C | 28 | 0.00758764288880082 | FAM83E | 36 | 0.600412529261819 |  |  |  |
| FAM83C | 28 | 0.00660744176248379 | FAM83E | 36 | 0.553173555458232 |  |  |  |
| FAM83C | 28 | 0.00798611601494325 | FAM83E | 37 | 0.595063641221696 |  |  |  |
| FAM83C | 28 | 0.00638202350098032 | FAM83E | 37 | 0.62922373966276 |  |  |  |
| FAM83C | 28 | 0.00763090927045254 | FAM83E | 37 | 0.594700604247384 |  |  |  |
| FAM83C | 28 | 0.00927804765763346 | FAM83E | 37 | 0.633197956889204 |  |  |  |
| FAM83C | 28 | 0.00747970120424676 | FAM83E | 37 | 0.606095360480466 |  |  |  |
| FAM83C | 28 | 0.00863751416938814 | FAM83E | 37 | 0.556688087332527 |  |  |  |
| FAM83C | 28 | 0.0100890208311149 | FAM83E | 37 | 0.504772855341647 |  |  |  |
| FAM83C | 28 | 0.0113681180666606 | FAM83E | 38 | 0.455426666039396 |  |  |  |
| FAM83C | 28 | 0.00996597466936435 | FAM83E | 38 | 0.416827674625541 |  |  |  |
| FAM83C | 28 | 0.00824640784225194 | FAM83E | 38 | 0.460406453477429 |  |  |  |
| FAM83C | 28 | 0.00662273935083043 | FAM83E | 38 | 0.504570244896521 |  |  |  |
| FAM83C | 28 | 0.00544429583009693 | FAM83E | 38 | 0.45509065669795 |  |  |  |
| FAM83C | 29 | 0.00467155410696651 | FAM83E | 38 | 0.421040916116105 |  |  |  |
| FAM83C | 29 | 0.0054389059869243 | FAM83E | 38 | 0.380210846061096 |  |  |  |
| FAM83C | 29 | 0.00434543558203604 | FAM83E | 39 | 0.404897412920439 |  |  |  |
| FAM83C | 29 | 0.00396863520343844 | FAM83E | 39 | 0.451923626596331 |  |  |  |
| FAM83C | 29 | 0.00496804772896444 | FAM83E | 39 | 0.415432211869244 |  |  |  |
| FAM83C | 29 | 0.00442674229548215 | FAM83E | 39 | 0.385507655623133 |  |  |  |
| FAM83C | 29 | 0.00524016869785069 | FAM83E | 39 | 0.442209373195296 |  |  |  |
| FAM83C | 29 | 0.00659306835502245 | FAM83E | 39 | 0.405345660328881 |  |  |  |
| FAM83C | 29 | 0.00711257486539407 | FAM83E | 40 | 0.37580644029196 |  |  |  |
| FAM83C | 29 | 0.00915111200629186 | FAM83E | 40 | 0.34235540444873 |  |  |  |
| FAM83C | 29 | 0.00735481099680746 | FAM83E | 40 | 0.31309542114387 |  |  |  |
| FAM83C | 29 | 0.00634407380748545 | FAM83E | 40 | 0.287414222984516 |  |  |  |
| FAM83C | 29 | 0.00536126311591132 | FAM83E | 40 | 0.314219746579206 |  |  |  |
| FAM83C | 29 | 0.00463748724567315 | FAM83E | 40 | 0.357028661074205 |  |  |  |
| FAM83C | 29 | 0.00389677337153006 | FAM83E | 40 | 0.334852370692874 |  |  |  |
| FAM83C | 30 | 0.00322622686329488 | FAM83E | 40 | 0.383898302863801 |  |  |  |
| FAM83C | 30 | 0.00338447720853512 | FAM83E | 40 | 0.349224615550942 |  |  |  |
| FAM83C | 30 | 0.00392621906124913 | FAM83E | 41 | 0.399597591845771 |  |  |  |
| FAM83C | 30 | 0.00287709616036152 | FAM83E | 41 | 0.456602439154214 |  |  |  |
| FAM83C | 30 | 0.00354754616605893 | FAM83E | 41 | 0.435039927797605 |  |  |  |
| FAM83C | 30 | 0.00298869997508611 | FAM83E | 41 | 0.480489800650814 |  |  |  |
| FAM83C | 30 | 0.00261833275337397 | FAM83E | 41 | 0.441785175467046 |  |  |  |
| FAM83C | 30 | 0.00311090123782857 | FAM83E | 41 | 0.395194352545722 |  |  |  |
| FAM83C | 30 | 0.00362720228218289 | FAM83E | 42 | 0.441290923090663 |  |  |  |
| FAM83C | 30 | 0.00303025037865371 | FAM83E | 42 | 0.397677477633303 |  |  |  |
| FAM83C | 30 | 0.00319257114881487 | FAM83E | 42 | 0.376614718256025 |  |  |  |
| FAM83C | 30 | 0.0022626808700071 | FAM83E | 42 | 0.339755518545253 |  |  |  |
| FAM83C | 30 | 0.00177377984032434 | FAM83E | 42 | 0.308814902157647 |  |  |  |
| FAM83C | 30 | 0.00153730858135175 | FAM83E | 43 | 0.337501291029658 |  |  |  |
| FAM83C | 30 | 0.00138855644128368 | FAM83E | 43 | 0.30426590321644 |  |  |  |
| FAM83C | 30 | 0.00165045714089675 | FAM83E | 43 | 0.331729326132823 |  |  |  |
| FAM83C | 30 | 0.00205415450707833 | FAM83E | 43 | 0.377415780961559 |  |  |  |
| FAM83C | 30 | 0.00187048332080962 | FAM83E | 43 | 0.342017947753008 |  |  |  |
| FAM83C | 30 | 0.00154024420210451 | FAM83E | 43 | 0.383522738926424 |  |  |  |
| FAM83C | 30 | 0.00127279105260347 | FAM83E | 43 | 0.340313482697657 |  |  |  |
| FAM83C | 30 | 0.00107800802249026 | FAM83E | 43 | 0.308927384538946 |  |  |  |
| FAM83C | 31 | 0.000886249321219099 | FAM83E | 43 | 0.288706927361902 |  |  |  |
| FAM83C | 31 | 0.00120361045182756 | FAM83E | 44 | 0.31714039974897 |  |  |  |
| FAM83C | 31 | 0.00160196060466334 | FAM83E | 44 | 0.367082853451903 |  |  |  |
| FAM83C | 31 | 0.00186674220799662 | FAM83E | 44 | 0.415622484788107 |  |  |  |
| FAM83C | 31 | 0.00151388944715053 | FAM83E | 44 | 0.379174249123056 |  |  |  |
| FAM83C | 31 | 0.00121717409895178 | FAM83E | 44 | 0.342571498087983 |  |  |  |
| FAM83C | 31 | 0.00153574376963904 | FAM83E | 45 | 0.390164357449687 |  |  |  |
| FAM83C | 31 | 0.00119710746676569 | FAM83E | 45 | 0.412872394109644 |  |  |  |
| FAM83C | 31 | 0.000872430717778293 | FAM83E | 45 | 0.38487674477931 |  |  |  |
| FAM83C | 32 | 0.00065776809644397 | FAM83E | 45 | 0.343970589395257 |  |  |  |
| FAM83C | 32 | 0.000497152044331491 | FAM83E | 45 | 0.372739128443244 |  |  |  |
| FAM83C | 32 | 0.000412864071821512 | FAM83E | 45 | 0.39139862951542 |  |  |  |
| FAM83C | 32 | 0.000520823083242281 | FAM83E | 45 | 0.358853887554861 |  |  |  |
| FAM83C | 32 | 0.00058358343767188 | FAM83E | 45 | 0.415091652585546 |  |  |  |
| FAM83C | 32 | 0.000679540131558008 | FAM83E | 46 | 0.383223374246725 |  |  |  |
| FAM83C | 32 | 0.000935319529415042 | FAM83E | 46 | 0.348135011535585 |  |  |  |
| FAM83C | 32 | 0.000695978843971125 | FAM83E | 46 | 0.316466075759504 |  |  |  |
| FAM83C | 32 | 0.000525522646551446 | FAM83E | 46 | 0.292156188016747 |  |  |  |
| FAM83C | 32 | 0.000627819179414874 | FAM83E | 46 | 0.313404355358869 |  |  |  |
| FAM83C | 32 | 0.000495044016449374 | FAM83E | 47 | 0.307377671243462 |  |  |  |
| FAM83C | 32 | 0.000679645106177207 | FAM83E | 47 | 0.272403647954213 |  |  |  |
| FAM83C | 32 | 0.000803722573292058 | FAM83E | 47 | 0.266023639856147 |  |  |  |
| FAM83C | 32 | 0.000658478907694979 | FAM83E | 47 | 0.246335029402646 |  |  |  |
| FAM83C | 33 | 0.000588751136997964 | FAM83E | 48 | 0.220550373804953 |  |  |  |
| FAM83C | 33 | 0.000641633296033991 | FAM83E | 48 | 0.201003715070051 |  |  |  |
| FAM83C | 33 | 0.00085335835826779 | FAM83E | 48 | 0.180578880957617 |  |  |  |
| FAM83C | 33 | 0.00102746283097032 | FAM83E | 48 | 0.159667247124913 |  |  |  |
| FAM83C | 33 | 0.00122596132672804 | FAM83E | 48 | 0.140444644963492 |  |  |  |
| FAM83C | 33 | 0.00114963103076032 | FAM83E | 48 | 0.128130722867276 |  |  |  |
| FAM83C | 33 | 0.001515022590814 | FAM83E | 48 | 0.155344984850224 |  |  |  |
| FAM83C | 33 | 0.00203558562647805 | FAM83E | 48 | 0.13965032741362 |  |  |  |
| FAM83C | 33 | 0.00234886942475806 | FAM83E | 49 | 0.127357705588091 |  |  |  |
| FAM83C | 33 | 0.00170521756788769 | FAM83E | 49 | 0.110550620568211 |  |  |  |
| FAM83C | 33 | 0.00139497751820196 | FAM83E | 49 | 0.108152929930807 |  |  |  |
| FAM83C | 33 | 0.00113477687874497 | FAM83E | 49 | 0.0989590158795741 |  |  |  |
| FAM83C | 33 | 0.000964631408270413 | FAM83E | 49 | 0.0883759503020054 |  |  |  |
| FAM83C | 33 | 0.000790436732862225 | FAM83E | 49 | 0.0732820691699925 |  |  |  |
| FAM83C | 34 | 0.000642947874688968 | FAM83E | 50 | 0.0625483674870112 |  |  |  |
| FAM83C | 34 | 0.000883654141035594 | FAM83E | 50 | 0.0553499576655131 |  |  |  |
| FAM83C | 34 | 0.00113064195160571 | FAM83E | 50 | 0.0527364540567333 |  |  |  |
| FAM83C | 34 | 0.000999833070467917 | FAM83E | 51 | 0.0606826022541688 |  |  |  |
| FAM83C | 34 | 0.00128760179750918 | FAM83E | 51 | 0.0512762459931002 |  |  |  |
| FAM83C | 34 | 0.00136597781369433 | FAM83E | 51 | 0.0436159553080611 |  |  |  |
| FAM83C | 34 | 0.00116848714734107 | FAM83E | 51 | 0.0550112187802924 |  |  |  |
| FAM83C | 34 | 0.000787574792790323 | FAM83E | 51 | 0.0492337605108493 |  |  |  |
| FAM83C | 34 | 0.000809991862074755 | FAM83E | 52 | 0.0562172564667784 |  |  |  |
| FAM83C | 34 | 0.000931379334443082 | FAM83E | 52 | 0.0457110242416147 |  |  |  |
| FAM83C | 35 | 0.000763625176696012 | FAM83E | 52 | 0.0399480477134884 |  |  |  |
| FAM83C | 35 | 0.00105805883321506 | FAM83E | 52 | 0.0494891192960615 |  |  |  |
| FAM83C | 35 | 0.00143886605160857 | FAM83E | 52 | 0.0437440564050896 |  |  |  |
| FAM83C | 35 | 0.00117561782080999 | FAM83E | 53 | 0.0505279188929039 |  |  |  |
| FAM83C | 35 | 0.000980754562478197 | FAM83E | 53 | 0.0495942594776602 |  |  |  |
| FAM83C | 35 | 0.00132227389255394 | FAM83E | 53 | 0.0476747049953287 |  |  |  |
| FAM83C | 35 | 0.00106800676251671 | FAM83E | 53 | 0.0402897142159496 |  |  |  |
| FAM83C | 35 | 0.000942096549975556 | FAM83E | 54 | 0.0479730994750472 |  |  |  |
| FAM83C | 35 | 0.000775406245881957 | FAM83E | 54 | 0.0577208621738124 |  |  |  |
| FAM83C | 36 | 0.000612966817086427 | FAM83E | 54 | 0.0686145614853556 |  |  |  |
| FAM83C | 36 | 0.000830338902021933 | FAM83E | 55 | 0.0586211163470283 |  |  |  |
| FAM83C | 36 | 0.000958903599360189 | FAM83E | 55 | 0.0519357988415783 |  |  |  |
| FAM83C | 36 | 0.000729538492753412 | FAM83E | 55 | 0.061208700654307 |  |  |  |
| FAM83C | 36 | 0.000921472407305059 | FAM83E | 55 | 0.0691530685277782 |  |  |  |
| FAM83C | 37 | 0.00122933895576822 | FAM83E | 56 | 0.0854743789990169 |  |  |  |
| FAM83C | 37 | 0.00114528933611366 | FAM83E | 56 | 0.073507697068128 |  |  |  |
| FAM83C | 37 | 0.000752953595134288 | FAM83E | 57 | 0.0630796427051401 |  |  |  |
| FAM83C | 37 | 0.000829027419713908 | FAM83E | 58 | 0.053281355238617 |  |  |  |
| FAM83C | 37 | 0.000671850127825434 | FAM83E | 59 | 0.0667688020725368 |  |  |  |
| FAM83C | 37 | 0.000841590723984742 | FAM83E | 59 | 0.056377512506729 |  |  |  |
| FAM83C | 37 | 0.000682955297604267 | FAM83E | 59 | 0.0483374617391956 |  |  |  |
| FAM83C | 37 | 0.000927709366261716 | FAM83E | 60 | 0.0408026958245081 |  |  |  |
| FAM83C | 37 | 0.00119754162598237 | FAM83E | 60 | 0.0348856475040269 |  |  |  |
| FAM83C | 37 | 0.00164349023309909 | FAM83E | 61 | 0.0281037281813334 |  |  |  |
| FAM83C | 37 | 0.00197670569942914 | FAM83E | 61 | 0.036431123257672 |  |  |  |
| FAM83C | 38 | 0.00162096733011834 | FAM83E | 61 | 0.0460921034012944 |  |  |  |
| FAM83C | 38 | 0.00182034643097005 | FAM83E | 62 | 0.0388028289710915 |  |  |  |
| FAM83C | 38 | 0.00121823434391105 | FAM83E | 62 | 0.0495515395455403 |  |  |  |
| FAM83C | 38 | 0.00148080047647514 | FAM83E | 62 | 0.0543953320302489 |  |  |  |
| FAM83C | 38 | 0.00121488784006659 | FAM83E | 63 | 0.0468831177564377 |  |  |  |
| FAM83C | 38 | 0.0016183659802006 | FAM83E | 63 | 0.0390905327263713 |  |  |  |
| FAM83C | 38 | 0.00194136929087236 | FAM83E | 63 | 0.0355855088272273 |  |  |  |
| FAM83C | 38 | 0.00260333675793282 | FAM83E | 63 | 0.0296792991877822 |  |  |  |
| FAM83C | 38 | 0.00212062833233713 | FAM83E | 63 | 0.027045543424563 |  |  |  |
| FAM83C | 39 | 0.0018559820773599 | FAM83E | 63 | 0.0346547811609251 |  |  |  |
| FAM83C | 39 | 0.00252766477784727 | FAM83E | 63 | 0.0288595224593912 |  |  |  |
| FAM83C | 40 | 0.00211685122633777 | FAM83E | 64 | 0.0242184297540257 |  |  |  |
| FAM83C | 40 | 0.00281601896531431 | FAM83F | 51 | 0.0587394283127292 |  |  |  |
| FAM83C | 40 | 0.00245815731573553 | FAM83F | 51 | 0.0509162595264963 |  |  |  |
| FAM83C | 41 | 0.00207381049035787 | FAM83F | 52 | 0.0421875558354086 |  |  |  |
| FAM83C | 41 | 0.00261876017987871 | FAM83F | 52 | 0.0552977039256385 |  |  |  |
| FAM83C | 41 | 0.00325190894804197 | FAM83F | 52 | 0.0486681250211206 |  |  |  |
| FAM83C | 41 | 0.00419447524625939 | FAM83F | 52 | 0.0423597061154905 |  |  |  |
| FAM83C | 42 | 0.00377872202746059 | FAM83F | 52 | 0.0589529972143815 |  |  |  |
| FAM83C | 42 | 0.00500661813845093 | FAM83F | 52 | 0.0794287810429863 |  |  |  |
| FAM83C | 42 | 0.00428456586191072 | FAM83F | 52 | 0.0672875250823714 |  |  |  |
| FAM83C | 42 | 0.00534688685103622 | FAM83F | 53 | 0.0581262863987029 |  |  |  |
| FAM83C | 42 | 0.00705185310556681 | FAM83F | 53 | 0.053008580519159 |  |  |  |
| FAM83C | 43 | 0.00899429324732964 | FAM83F | 53 | 0.0698227679858507 |  |  |  |
| FAM83C | 43 | 0.00812971631603861 | FAM83F | 54 | 0.0594545600858182 |  |  |  |
| FAM83C | 43 | 0.00942955390099123 | FAM83F | 54 | 0.0502681792947613 |  |  |  |
| FAM83C | 43 | 0.0118274737511239 | FAM83F | 54 | 0.0421282787999849 |  |  |  |
| FAM83C | 44 | 0.0153031398808729 | FAM83F | 54 | 0.0356227279894816 |  |  |  |
| FAM83C | 44 | 0.0167122059159074 | FAM83F | 54 | 0.0470132460533185 |  |  |  |
| FAM83C | 44 | 0.0146393347406724 | FAM83F | 54 | 0.0420167505852042 |  |  |  |
| FAM83C | 44 | 0.0186496611434471 | FAM83F | 54 | 0.0542761403761424 |  |  |  |
| FAM83C | 44 | 0.0219013128380074 | FAM83F | 55 | 0.0512395551793636 |  |  |  |
| FAM83C | 44 | 0.0270082441624513 | FAM83F | 55 | 0.0634454675589793 |  |  |  |
| FAM83C | 44 | 0.0324247539208672 | FAM83F | 55 | 0.0625080591511601 |  |  |  |
| FAM83C | 44 | 0.0285497572644939 | FAM83F | 55 | 0.0852125483900994 |  |  |  |
| FAM83C | 45 | 0.0236388424123044 | FAM83F | 55 | 0.102896619669545 |  |  |  |
| FAM83C | 45 | 0.0180552829186011 | FAM83F | 56 | 0.0906579050788972 |  |  |  |
| FAM83C | 45 | 0.0164387196051495 | FAM83F | 56 | 0.0776713264422604 |  |  |  |
| FAM83C | 46 | 0.0195142485074299 | FAM83F | 56 | 0.0707997302334369 |  |  |  |
| FAM83C | 46 | 0.0165885364927452 | FAM83F | 56 | 0.0612242242488094 |  |  |  |
| FAM83C | 47 | 0.0146370972400383 | FAM83F | 56 | 0.07969319499461 |  |  |  |
| FAM83C | 47 | 0.0138540290461463 | FAM83F | 56 | 0.0984650899792294 |  |  |  |
| FAM83C | 47 | 0.0126364933308226 | FAM83F | 56 | 0.0856231778767162 |  |  |  |
| FAM83C | 49 | 0.0106342408594188 | FAM83F | 57 | 0.0735376342235197 |  |  |  |
| FAM83D | 364 | 0.000102935019125622 | FAM83F | 57 | 0.0627229049127028 |  |  |  |
| FAM83D | 365 | 0.000141555107226712 | FAM83F | 57 | 0.0833353071327658 |  |  |  |
| FAM83D | 366 | 0.000119778975654911 | FAM83F | 57 | 0.109087346805986 |  |  |  |
| FAM83D | 367 | 9.72763043607628e-05 | FAM83F | 57 | 0.132230051698681 |  |  |  |
| FAM83D | 367 | 8.7704088510425e-05 | FAM83F | 57 | 0.166109235811548 |  |  |  |
| FAM83D | 368 | 6.37173285452929e-05 | FAM83F | 57 | 0.151805097795614 |  |  |  |
| FAM83D | 373 | 8.16974641434319e-05 | FAM83F | 58 | 0.132615101864807 |  |  |  |
| FAM83D | 374 | 6.87147773953698e-05 | FAM83F | 58 | 0.120107771180113 |  |  |  |
| FAM83D | 374 | 9.72781787925721e-05 | FAM83F | 58 | 0.119938581495905 |  |  |  |
| FAM83D | 376 | 7.89462719833016e-05 | FAM83F | 58 | 0.104497467131277 |  |  |  |
| FAM83D | 378 | 9.91969619209457e-05 | FAM83F | 58 | 0.0903534333570695 |  |  |  |
| FAM83D | 378 | 0.000137905994372731 | FAM83F | 58 | 0.118537512995628 |  |  |  |
| FAM83D | 378 | 0.000109405669223783 | FAM83F | 59 | 0.106799183387322 |  |  |  |
| FAM83D | 379 | 0.000155694649600182 | FAM83F | 59 | 0.0927973980457312 |  |  |  |
| FAM83D | 380 | 0.000129575628481329 | FAM83F | 59 | 0.120447820055712 |  |  |  |
| FAM83D | 380 | 0.000109858308347659 | FAM83F | 60 | 0.108219681361935 |  |  |  |
| FAM83D | 381 | 8.90960936413133e-05 | FAM83F | 60 | 0.134833761233242 |  |  |  |
| FAM83D | 381 | 0.000119623426983075 | FAM83F | 60 | 0.12135847849038 |  |  |  |
| FAM83D | 382 | 0.000104209042599185 | FAM83F | 60 | 0.14370227715539 |  |  |  |
| FAM83D | 383 | 8.56643573980786e-05 | FAM83F | 61 | 0.128865077469776 |  |  |  |
| FAM83D | 384 | 6.72879191376008e-05 | FAM83F | 61 | 0.115273397759997 |  |  |  |
| FAM83D | 384 | 5.35542518266826e-05 | FAM83F | 61 | 0.144139048528126 |  |  |  |
| FAM83D | 386 | 7.25920699079819e-05 | FAM83F | 61 | 0.121806122183437 |  |  |  |
| FAM83D | 387 | 6.26095984969759e-05 | FAM83F | 61 | 0.105644505035669 |  |  |  |
| FAM83D | 389 | 5.05169858745062e-05 | FAM83F | 62 | 0.0912648763999907 |  |  |  |
| FAM83D | 389 | 4.35803791408752e-05 | FAM83F | 62 | 0.117854450537509 |  |  |  |
| FAM83D | 389 | 6.38558297875561e-05 | FAM83F | 62 | 0.103713404079058 |  |  |  |
| FAM83D | 393 | 5.23126017404e-05 | FAM83F | 62 | 0.0911007395550764 |  |  |  |
| FAM83D | 394 | 4.18204166544775e-05 | FAM83F | 62 | 0.0802528926592654 |  |  |  |
| FAM83D | 399 | 5.01284059664373e-05 | FAM83F | 63 | 0.0700645560551131 |  |  |  |
| FAM83D | 400 | 4.06129421260993e-05 | FAM83F | 63 | 0.0591434599468466 |  |  |  |
| FAM83D | 401 | 4.33296586637986e-05 | FAM83F | 64 | 0.0507127349387412 |  |  |  |
| FAM83D | 402 | 5.64089022545329e-05 | FAM83F | 64 | 0.0434024772141904 |  |  |  |
| FAM83D | 402 | 7.78791022551669e-05 | FAM83F | 64 | 0.036487567515327 |  |  |  |
| FAM83D | 403 | 8.47698602128991e-05 | FAM83F | 64 | 0.0506098680575997 |  |  |  |
| FAM83D | 405 | 0.000118907650797895 | FAM83F | 64 | 0.0450549854551633 |  |  |  |
| FAM83D | 405 | 9.90089783285108e-05 | FAM83F | 65 | 0.0594122469871201 |  |  |  |
| FAM83D | 406 | 8.1240201403882e-05 | FAM83F | 65 | 0.0503633946645802 |  |  |  |
| FAM83D | 411 | 6.57479031443361e-05 | FAM83F | 65 | 0.0451620281694463 |  |  |  |
| FAM83D | 411 | 8.86013607277954e-05 | FAM83F | 65 | 0.0381948726301214 |  |  |  |
| FAM83D | 412 | 7.26874175521209e-05 | FAM83F | 66 | 0.0509888435349501 |  |  |  |
| FAM83D | 413 | 9.23822887270295e-05 | FAM83F | 66 | 0.0468637504282281 |  |  |  |
| FAM83D | 414 | 7.514490383561e-05 | FAM83F | 66 | 0.0394920341150926 |  |  |  |
| FAM83D | 416 | 0.000108537534357911 | FAM83F | 66 | 0.0328465267421999 |  |  |  |
| FAM83D | 416 | 8.38194462853289e-05 | FAM83F | 66 | 0.0287999862311155 |  |  |  |
| FAM83D | 417 | 9.92083309356768e-05 | FAM83F | 66 | 0.0239814807615009 |  |  |  |
| FAM83D | 417 | 7.7847552872082e-05 | FAM83F | 67 | 0.0192040584906819 |  |  |  |
| FAM83D | 418 | 6.3016721870672e-05 | FAM83F | 67 | 0.0156878030218532 |  |  |  |
| FAM83D | 422 | 5.02955911946747e-05 | FAM83F | 67 | 0.0128816860892583 |  |  |  |
| FAM83D | 422 | 4.07125361673217e-05 | FAM83F | 67 | 0.0108331220630435 |  |  |  |
| FAM83D | 423 | 5.85562887591507e-05 | FAM83F | 68 | 0.0157942321985542 |  |  |  |
| FAM83D | 423 | 7.30361435153376e-05 | FAM83F | 68 | 0.0131863158138052 |  |  |  |
| FAM83D | 424 | 5.68823119890768e-05 | FAM83F | 68 | 0.0112132319715169 |  |  |  |
| FAM83D | 427 | 4.37535833607079e-05 | FAM83F | 68 | 0.00913943272628365 |  |  |  |
| FAM83D | 427 | 3.50249763112077e-05 | FAM83F | 69 | 0.00751035975162396 |  |  |  |
| FAM83D | 427 | 2.52024438171299e-05 | FAM83F | 69 | 0.00622914020227929 |  |  |  |
| FAM83D | 431 | 1.97414596425148e-05 | FAM83F | 69 | 0.00826339961279939 |  |  |  |
| FAM83D | 434 | 1.61863974668492e-05 | FAM83F | 69 | 0.00677633119577363 |  |  |  |
| FAM83D | 435 | 1.52798243124128e-05 | FAM83F | 69 | 0.00563101743061352 |  |  |  |
| FAM83D | 435 | 1.22238104224415e-05 | FAM83F | 69 | 0.00459531764268926 |  |  |  |
| FAM83D | 437 | 8.79813558842854e-06 | FAM83F | 69 | 0.00373548898338286 |  |  |  |
| FAM83D | 437 | 6.60916736933011e-06 | FAM83F | 70 | 0.00297496131234575 |  |  |  |
| FAM83D | 438 | 9.09362866678108e-06 | FAM83F | 70 | 0.00430060150389185 |  |  |  |
| FAM83D | 438 | 5.71247633315757e-06 | FAM83F | 70 | 0.00369146228774385 |  |  |  |
| FAM83D | 440 | 4.30263141311792e-06 | FAM83F | 70 | 0.00499767168852715 |  |  |  |
| FAM83D | 441 | 6.53952659641171e-06 | FAM83F | 70 | 0.00407763567340646 |  |  |  |
| FAM83D | 442 | 5.3272422374192e-06 | FAM83F | 71 | 0.00327251686798346 |  |  |  |
| FAM83D | 443 | 4.37880843989708e-06 | FAM83F | 71 | 0.00464344102195374 |  |  |  |
| FAM83D | 443 | 5.58364920400108e-06 | FAM83F | 71 | 0.00379126973164196 |  |  |  |
| FAM83D | 445 | 4.348166087453e-06 | FAM83F | 72 | 0.00311049779778586 |  |  |  |
| FAM83D | 445 | 3.14958773418437e-06 | FAM83F | 72 | 0.00445202631100192 |  |  |  |
| FAM83D | 445 | 3.78194158411382e-06 | FAM83F | 72 | 0.0037632376581316 |  |  |  |
| FAM83D | 445 | 3.27162884515206e-06 | FAM83F | 73 | 0.00296155384541057 |  |  |  |
| FAM83D | 450 | 2.57551276358709e-06 | FAM83F | 73 | 0.00234564816265963 |  |  |  |
| FAM83D | 450 | 2.0059619024042e-06 | FAM83F | 73 | 0.00204975677296069 |  |  |  |
| FAM83D | 451 | 2.29354798907591e-06 | FAM83F | 73 | 0.00308738829522383 |  |  |  |
| FAM83D | 452 | 1.74523221368888e-06 | FAM83F | 73 | 0.00254678095382932 |  |  |  |
| FAM83D | 452 | 2.60276889572221e-06 | FAM83F | 74 | 0.00192587109841945 |  |  |  |
| FAM83D | 453 | 1.62852331515702e-06 | FAM83F | 74 | 0.00250901833226091 |  |  |  |
| FAM83D | 455 | 9.92986588505928e-07 | FAM83F | 74 | 0.00369001680059974 |  |  |  |
| FAM83D | 458 | 1.48027996619804e-06 | FAM83F | 75 | 0.00465409196038377 |  |  |  |
| FAM83D | 459 | 1.77590023402781e-06 | FAM83F | 75 | 0.00388301323269831 |  |  |  |
| FAM83D | 459 | 2.69108053453624e-06 | FAM83F | 75 | 0.00576537420884258 |  |  |  |
| FAM83D | 460 | 3.80824533575149e-06 | FAM83F | 76 | 0.00470031313009093 |  |  |  |
| FAM83D | 460 | 5.56308217356777e-06 | FAM83F | 76 | 0.00388880035112353 |  |  |  |
| FAM83D | 462 | 4.9372520053936e-06 | FAM83F | 76 | 0.00579397988784099 |  |  |  |
| FAM83D | 463 | 7.4649565450323e-06 | FAM83F | 76 | 0.00512397089917603 |  |  |  |
| FAM83D | 464 | 5.92505476755354e-06 | FAM83F | 76 | 0.00702174705564101 |  |  |  |
| FAM83D | 464 | 8.76477391697779e-06 | FAM83F | 76 | 0.00565500377997345 |  |  |  |
| FAM83D | 464 | 1.14331658247724e-05 | FAM83F | 76 | 0.00479259639727968 |  |  |  |
| FAM83D | 465 | 1.41355031535241e-05 | FAM83F | 77 | 0.00668250725451722 |  |  |  |
| FAM83D | 465 | 1.86600149959913e-05 | FAM83F | 77 | 0.0081432382140929 |  |  |  |
| FAM83D | 467 | 2.68510480983465e-05 | FAM83F | 78 | 0.00650520779768975 |  |  |  |
| FAM83D | 468 | 2.27111585635483e-05 | FAM83F | 79 | 0.0090357330730902 |  |  |  |
| FAM83D | 469 | 2.68598184049561e-05 | FAM83F | 79 | 0.00745859357161691 |  |  |  |
| FAM83D | 469 | 2.21373477485954e-05 | FAM83F | 79 | 0.00620550840964177 |  |  |  |
| FAM83D | 470 | 1.76742616858931e-05 | FAM83F | 80 | 0.00518796535693802 |  |  |  |
| FAM83D | 470 | 1.55656079624363e-05 | FAM83F | 81 | 0.00436853387004932 |  |  |  |
| FAM83D | 471 | 1.37055578668094e-05 | FAM83F | 81 | 0.00607053771015001 |  |  |  |
| FAM83D | 471 | 6.59691711647976e-06 | FAM83F | 81 | 0.0049456600303104 |  |  |  |
| FAM83D | 471 | 5.18525618343736e-06 | FAM83F | 82 | 0.00419502447823804 |  |  |  |
| FAM83D | 475 | 4.21038784321741e-06 | FAM83F | 82 | 0.00408947222147003 |  |  |  |
| FAM83D | 475 | 3.05589453259135e-06 | FAM83F | 82 | 0.00327039006108093 |  |  |  |
| FAM83D | 477 | 4.19492459138705e-06 | FAM83F | 82 | 0.00260281231062384 |  |  |  |
| FAM83D | 477 | 6.09969398807273e-06 | FAM83F | 83 | 0.00207096081890356 |  |  |  |
| FAM83D | 477 | 5.12218072905067e-06 | FAM83F | 83 | 0.00278175321886581 |  |  |  |
| FAM83D | 479 | 6.78659241645456e-06 | FAM83F | 83 | 0.00416879477090731 |  |  |  |
| FAM83D | 481 | 9.75174759220645e-06 | FAM83F | 84 | 0.0062600358325008 |  |  |  |
| FAM83D | 481 | 7.81563673874357e-06 | FAM83F | 84 | 0.00563936819843375 |  |  |  |
| FAM83D | 481 | 6.66144166321458e-06 | FAM83F | 84 | 0.00478936879513856 |  |  |  |
| FAM83D | 482 | 5.0133392767628e-06 | FAM83F | 84 | 0.00406184827329748 |  |  |  |
| FAM83D | 482 | 3.22677153563056e-06 | FAM83F | 85 | 0.0030880143304197 |  |  |  |
| FAM83D | 484 | 3.18274040634257e-06 | FAM83F | 86 | 0.002435304494422 |  |  |  |
| FAM83D | 484 | 4.21298602383901e-06 | FAM83F | 86 | 0.00192340929007718 |  |  |  |
| FAM83D | 484 | 6.27121481898165e-06 | FAM83F | 86 | 0.00246688741714755 |  |  |  |
| FAM83D | 485 | 5.00677130690015e-06 | FAM83F | 86 | 0.00196938212478853 |  |  |  |
| FAM83D | 486 | 3.79547460492304e-06 | FAM83F | 87 | 0.00158795357788604 |  |  |  |
| FAM83D | 487 | 5.52954231493428e-06 | FAM83F | 88 | 0.00124207555562292 |  |  |  |
| FAM83D | 487 | 7.94259713734667e-06 | FAM83F | 88 | 0.00103079751272644 |  |  |  |
| FAM83D | 490 | 6.68349503570168e-06 | FAM83F | 88 | 0.000803084802066262 |  |  |  |
| FAM83D | 491 | 9.57822848933527e-06 | FAM83F | 89 | 0.000624814657618533 |  |  |  |
| FAM83D | 493 | 8.22234813586165e-06 | FAM83F | 89 | 0.000985255899749799 |  |  |  |
| FAM83D | 495 | 6.02235876938448e-06 | FAM83F | 89 | 0.00153680923568119 |  |  |  |
| FAM83D | 497 | 4.74260503882703e-06 | FAM83F | 90 | 0.00196884498513111 |  |  |  |
| FAM83D | 502 | 4.18123498453226e-06 | FAM83F | 90 | 0.00160097708952838 |  |  |  |
| FAM83D | 502 | 3.13104393919654e-06 | FAM83F | 90 | 0.00248584480951702 |  |  |  |
| FAM83D | 503 | 3.83564796422666e-06 | FAM83F | 90 | 0.00196731413472191 |  |  |  |
| FAM83D | 504 | 5.33386615236419e-06 | FAM83F | 91 | 0.00170300930932036 |  |  |  |
| FAM83D | 505 | 3.72925465222282e-06 | FAM83F | 91 | 0.00133690023203474 |  |  |  |
| FAM83D | 506 | 5.4602033412407e-06 | FAM83F | 91 | 0.00183202205795177 |  |  |  |
| FAM83D | 508 | 6.75662150726159e-06 | FAM83F | 92 | 0.00160086432782134 |  |  |  |
| FAM83D | 510 | 9.61329747178484e-06 | FAM83F | 92 | 0.00130915605595735 |  |  |  |
| FAM83D | 510 | 6.13064620541853e-06 | FAM83F | 92 | 0.00181058929505555 |  |  |  |
| FAM83D | 512 | 4.88659472554609e-06 | FAM83F | 92 | 0.00274146629668044 |  |  |  |
| FAM83D | 512 | 3.84117578619636e-06 | FAM83F | 94 | 0.00220051797207267 |  |  |  |
| FAM83D | 512 | 5.71972988296265e-06 | FAM83F | 94 | 0.00173342478286165 |  |  |  |
| FAM83D | 513 | 4.29918359967217e-06 | FAM83F | 94 | 0.00229738870945739 |  |  |  |
| FAM83D | 514 | 3.31770313792736e-06 | FAM83F | 95 | 0.00176706238997681 |  |  |  |
| FAM83D | 520 | 3.71716883633895e-06 | FAM83F | 95 | 0.00139360860342384 |  |  |  |
| FAM83D | 520 | 3.0860527327186e-06 | FAM83F | 96 | 0.00109354055757513 |  |  |  |
| FAM83D | 521 | 2.41242654698274e-06 | FAM83F | 96 | 0.000867248214130662 |  |  |  |
| FAM83D | 526 | 1.86061449152591e-06 | FAM83F | 96 | 0.000692329089225254 |  |  |  |
| FAM83D | 526 | 1.14124536153535e-06 | FAM83F | 97 | 0.00064275022130317 |  |  |  |
| FAM83D | 527 | 1.37394975247122e-06 | FAM83F | 97 | 0.000816466720019847 |  |  |  |
| FAM83D | 528 | 1.67973068619702e-06 | FAM83F | 97 | 0.00124249257373426 |  |  |  |
| FAM83D | 528 | 1.73272843348837e-06 | FAM83F | 98 | 0.00195093877506731 |  |  |  |
| FAM83D | 534 | 2.30520313001942e-06 | FAM83F | 99 | 0.0014401073259536 |  |  |  |
| FAM83D | 536 | 1.7991208681526e-06 | FAM83F | 99 | 0.00182530835241337 |  |  |  |
| FAM83D | 536 | 1.40084769869298e-06 | FAM83F | 99 | 0.00166586236014848 |  |  |  |
| FAM83D | 538 | 1.98745502005577e-06 | FAM83F | 100 | 0.00130795754152501 |  |  |  |
| FAM83D | 539 | 1.5415028941631e-06 | FAM83F | 100 | 0.00187716664098795 |  |  |  |
| FAM83D | 539 | 1.34681016631432e-06 | FAM83F | 101 | 0.0027087318327321 |  |  |  |
| FAM83D | 540 | 1.02427361808341e-06 | FAM83F | 101 | 0.0038616532208859 |  |  |  |
| FAM83D | 541 | 7.9331556213692e-07 | FAM83F | 101 | 0.00586107499020007 |  |  |  |
| FAM83D | 541 | 6.19015689001368e-07 | FAM83F | 102 | 0.00467077813572949 |  |  |  |
| FAM83D | 542 | 4.55586271195665e-07 | FAM83F | 102 | 0.00429174549939207 |  |  |  |
| FAM83D | 545 | 5.91580121451475e-07 | FAM83F | 102 | 0.00617225230779967 |  |  |  |
| FAM83D | 546 | 4.31665037196157e-07 | FAM83F | 102 | 0.0083902192106175 |  |  |  |
| FAM83D | 547 | 2.84294612293367e-07 | FAM83F | 103 | 0.010698911077809 |  |  |  |
| FAM83D | 548 | 3.96233805999033e-07 | FAM83F | 104 | 0.0134413305558376 |  |  |  |
| FAM83D | 548 | 5.7755270324593e-07 | FAM83F | 104 | 0.017356351738201 |  |  |  |
| FAM83D | 548 | 7.46199940160043e-07 | FAM83F | 105 | 0.0224183161946159 |  |  |  |
| FAM83D | 553 | 5.84658704652458e-07 | FAM83F | 105 | 0.0193805690078711 |  |  |  |
| FAM83D | 556 | 6.73483933661843e-07 | FAM83F | 106 | 0.0173505471377387 |  |  |  |
| FAM83D | 559 | 5.27172752513995e-07 | FAM83F | 106 | 0.0245265555624133 |  |  |  |
| FAM83D | 561 | 6.68124519149201e-07 | FAM83F | 108 | 0.0200938348633388 |  |  |  |
| FAM83D | 563 | 8.79512753720796e-07 | FAM83F | 109 | 0.0286519428121679 |  |  |  |
| FAM83D | 564 | 1.12767532709858e-06 | FAM83F | 110 | 0.0258827870646248 |  |  |  |
| FAM83D | 566 | 9.43465687284416e-07 | FAM83F | 111 | 0.0321845042532175 |  |  |  |
| FAM83D | 566 | 1.21774127982987e-06 | FAM83F | 111 | 0.0440915993890994 |  |  |  |
| FAM83D | 568 | 9.19242425660683e-07 | FAM83F | 111 | 0.0600257288766074 |  |  |  |
| FAM83D | 568 | 1.25732157086351e-06 | FAM83F | 111 | 0.0789931357263833 |  |  |  |
| FAM83D | 569 | 1.03529428527822e-06 | FAM83F | 111 | 0.0732682179286593 |  |  |  |
| FAM83D | 569 | 1.55683339233553e-06 | FAM83F | 111 | 0.0619174134678806 |  |  |  |
| FAM83D | 571 | 2.07487833214762e-06 | FAM83F | 112 | 0.0785173832531707 |  |  |  |
| FAM83D | 571 | 2.03465726083114e-06 | FAM83F | 112 | 0.0671420305024324 |  |  |  |
| FAM83D | 573 | 2.1729458047739e-06 | FAM83F | 112 | 0.0607100637354942 |  |  |  |
| FAM83D | 574 | 3.09731096469241e-06 | FAM83F | 113 | 0.0761397465414113 |  |  |  |
| FAM83D | 574 | 2.27193625632486e-06 | FAM83F | 114 | 0.0999025297228035 |  |  |  |
| FAM83D | 574 | 1.7884472446487e-06 | FAM83F | 114 | 0.0858190328427122 |  |  |  |
| FAM83D | 575 | 1.45037163295018e-06 | FAM83F | 115 | 0.072786029468315 |  |  |  |
| FAM83D | 578 | 2.05327153266393e-06 | FAM83F | 115 | 0.0913032882042645 |  |  |  |
| FAM83D | 578 | 2.53380115207971e-06 | FAM83F | 115 | 0.117529021680628 |  |  |  |
| FAM83D | 578 | 3.5926345517029e-06 | FAM83F | 116 | 0.102151484567742 |  |  |  |
| FAM83D | 581 | 2.94195223553358e-06 | FAM83F | 116 | 0.087719197813326 |  |  |  |
| FAM83D | 581 | 4.37607218530371e-06 | FAM83F | 116 | 0.11632859574087 |  |  |  |
| FAM83D | 584 | 3.69303383769943e-06 | FAM83F | 117 | 0.105943313371032 |  |  |  |
| FAM83D | 585 | 2.95032319101836e-06 | FAM83F | 117 | 0.105784299643242 |  |  |  |
| FAM83D | 586 | 2.6952974136964e-06 | FAM83F | 118 | 0.129353706084173 |  |  |  |
| FAM83D | 586 | 1.82171722542195e-06 | FAM83F | 118 | 0.111543279728681 |  |  |  |
| FAM83D | 588 | 2.67894660762256e-06 | FAM83F | 119 | 0.0981126548689598 |  |  |  |
| FAM83D | 590 | 1.49960600198099e-06 | FAM83F | 120 | 0.0853272752138679 |  |  |  |
| FAM83D | 591 | 2.10055459668153e-06 | FAM83F | 121 | 0.0725054498372151 |  |  |  |
| FAM83D | 591 | 1.80934519670497e-06 | FAM83F | 121 | 0.0982571700773337 |  |  |  |
| FAM83D | 592 | 2.49857189092388e-06 | FAM83F | 121 | 0.0885011813304297 |  |  |  |
| FAM83D | 595 | 3.29358501094956e-06 | FAM83F | 121 | 0.0856656018664297 |  |  |  |
| FAM83D | 596 | 4.8536999128898e-06 | FAM83F | 122 | 0.107142836679211 |  |  |  |
| FAM83D | 597 | 3.75706769488885e-06 | FAM83F | 124 | 0.137238717235695 |  |  |  |
| FAM83D | 598 | 4.87052140749695e-06 | FAM83F | 125 | 0.114471337925089 |  |  |  |
| FAM83D | 599 | 3.59105638934313e-06 | FAM83F | 126 | 0.104227975251007 |  |  |  |
| FAM83D | 601 | 5.21206406565831e-06 | FAM83F | 127 | 0.135039521876849 |  |  |  |
| FAM83D | 606 | 4.03677195767239e-06 | FAM83F | 127 | 0.171594598600593 |  |  |  |
| FAM83D | 607 | 4.36305065018871e-06 | FAM83F | 128 | 0.152599675025892 |  |  |  |
| FAM83D | 609 | 3.21130880019497e-06 | FAM83F | 128 | 0.132673108811334 |  |  |  |
| FAM83D | 614 | 2.26980839214165e-06 | FAM83F | 128 | 0.116187940263455 |  |  |  |
| FAM83D | 614 | 2.89203017463245e-06 | FAM83F | 129 | 0.143037687906098 |  |  |  |
| FAM83D | 614 | 2.22534294583871e-06 | FAM83F | 131 | 0.123728944404178 |  |  |  |
| FAM83D | 618 | 3.33077125576217e-06 | FAM83F | 131 | 0.156072336181623 |  |  |  |
| FAM83D | 621 | 2.49720160466433e-06 | FAM83F | 133 | 0.18555282205025 |  |  |  |
| FAM83D | 622 | 1.94722359624713e-06 | FAM83F | 133 | 0.167188177382904 |  |  |  |
| FAM83D | 625 | 1.46558392778631e-06 | FAM83F | 133 | 0.210829223935905 |  |  |  |
| FAM83D | 627 | 9.40096933502775e-07 | FAM83F | 134 | 0.184791045515782 |  |  |  |
| FAM83D | 627 | 7.30921184601039e-07 | FAM83F | 138 | 0.218126247383484 |  |  |  |
| FAM83D | 627 | 5.87962270628627e-07 | FAM83F | 139 | 0.188768943680038 |  |  |  |
| FAM83D | 631 | 7.65038297078731e-07 | FAM83F | 139 | 0.188513485009607 |  |  |  |
| FAM83D | 631 | 1.00260836102937e-06 | FAM83F | 140 | 0.158729899389214 |  |  |  |
| FAM83D | 635 | 1.29356754039452e-06 | FAM83F | 141 | 0.140582272701832 |  |  |  |
| FAM83D | 636 | 1.92836434226103e-06 | FAM83F | 142 | 0.116655076135524 |  |  |  |
| FAM83D | 638 | 1.52060049584059e-06 | FAM83F | 143 | 0.103281388944594 |  |  |  |
| FAM83D | 640 | 1.97821939593602e-06 | FAM83F | 144 | 0.134789545106576 |  |  |  |
| FAM83D | 642 | 1.54806527320011e-06 | FAM83F | 144 | 0.148747787964812 |  |  |  |
| FAM83D | 643 | 1.93926449288635e-06 | FAM83F | 145 | 0.146212483351864 |  |  |  |
| FAM83D | 644 | 2.50861226886717e-06 | FAM83F | 145 | 0.126344900122879 |  |  |  |
| FAM83D | 646 | 2.81419597347151e-06 | FAM83F | 145 | 0.1108113036495 |  |  |  |
| FAM83D | 646 | 3.49380076050216e-06 | FAM83F | 147 | 0.130021384475624 |  |  |  |
| FAM83D | 647 | 4.9256263738505e-06 | FAM83F | 147 | 0.117265957071976 |  |  |  |
| FAM83D | 647 | 4.34100854814218e-06 | FAM83F | 147 | 0.153552730702539 |  |  |  |
| FAM83D | 648 | 3.85566777605268e-06 | FAM83F | 149 | 0.142555712755129 |  |  |  |
| FAM83D | 651 | 2.95601409686794e-06 | FAM83F | 149 | 0.120701019744223 |  |  |  |
| FAM83D | 651 | 3.99680848456611e-06 | FAM83F | 149 | 0.106702852525788 |  |  |  |
| FAM83D | 653 | 5.06856276371441e-06 | FAM83F | 150 | 0.141148382751365 |  |  |  |
| FAM83D | 653 | 6.98414191164265e-06 | FAM83F | 151 | 0.121250629125743 |  |  |  |
| FAM83D | 656 | 5.57038403566909e-06 | FAM83F | 151 | 0.102726145990577 |  |  |  |
| FAM83D | 656 | 7.99496213355884e-06 | FAM83F | 151 | 0.136957419641693 |  |  |  |
| FAM83D | 657 | 8.92597632701429e-06 | FAM83F | 153 | 0.12263773419922 |  |  |  |
| FAM83D | 657 | 7.00817001584239e-06 | FAM83F | 154 | 0.111265708095464 |  |  |  |
| FAM83D | 658 | 6.85949639618093e-06 | FAM83F | 154 | 0.108155560763593 |  |  |  |
| FAM83D | 658 | 9.58982821148652e-06 | FAM83F | 155 | 0.131927312969199 |  |  |  |
| FAM83D | 661 | 7.90198904837987e-06 | FAM83F | 155 | 0.164164659345295 |  |  |  |
| FAM83D | 666 | 6.86484819580368e-06 | FAM83F | 155 | 0.144331188322919 |  |  |  |
| FAM83D | 668 | 6.0549367391744e-06 | FAM83F | 156 | 0.125993634806663 |  |  |  |
| FAM83D | 669 | 7.00339916358912e-06 | FAM83F | 157 | 0.161426667969091 |  |  |  |
| FAM83D | 669 | 9.68484105503367e-06 | FAM83F | 158 | 0.142054641720326 |  |  |  |
| FAM83D | 670 | 7.71046416171136e-06 | FAM83F | 159 | 0.130334191845047 |  |  |  |
| FAM83D | 670 | 1.11255185052324e-05 | FAM83F | 159 | 0.111884820151574 |  |  |  |
| FAM83D | 673 | 8.77076458181792e-06 | FAM83F | 160 | 0.132205698563014 |  |  |  |
| FAM83D | 673 | 1.17303729299451e-05 | FAM83F | 162 | 0.166907854575015 |  |  |  |
| FAM83D | 675 | 9.32845481957515e-06 | FAM83F | 163 | 0.213395407863879 |  |  |  |
| FAM83D | 675 | 1.35180924937906e-05 | FAM83F | 164 | 0.178689056824104 |  |  |  |
| FAM83D | 679 | 1.76375543866003e-05 | FAM83F | 164 | 0.214792619717076 |  |  |  |
| FAM83D | 680 | 1.72248862920858e-05 | FAM83F | 165 | 0.195355381673623 |  |  |  |
| FAM83D | 680 | 2.01067964073969e-05 | FAM83F | 165 | 0.249782364998546 |  |  |  |
| FAM83D | 680 | 1.82530270837826e-05 | FAM83F | 166 | 0.21904308304477 |  |  |  |
| FAM83D | 681 | 2.62241713364173e-05 | FAM83F | 167 | 0.188891644061737 |  |  |  |
| FAM83D | 682 | 1.75655770068014e-05 | FAM83F | 168 | 0.23961333338544 |  |  |  |
| FAM83D | 683 | 1.38781406583397e-05 | FAM83F | 168 | 0.305324602338267 |  |  |  |
| FAM83D | 684 | 9.0647277091555e-06 | FAM83F | 170 | 0.369504563239945 |  |  |  |
| FAM83D | 685 | 5.69605237263516e-06 | FAM83F | 172 | 0.429978464198165 |  |  |  |
| FAM83D | 689 | 7.91821323159293e-06 | FAM83F | 174 | 0.383738184152333 |  |  |  |
| FAM83D | 689 | 1.12554487654062e-05 | FAM83F | 176 | 0.471564605614893 |  |  |  |
| FAM83D | 689 | 1.49601312956643e-05 | FAM83F | 179 | 0.418815649283792 |  |  |  |
| FAM83D | 690 | 1.19523486593007e-05 | FAM83F | 179 | 0.466032264664072 |  |  |  |
| FAM83D | 691 | 1.55803042298319e-05 | FAM83F | 179 | 0.422553140331602 |  |  |  |
| FAM83D | 692 | 1.44413300065489e-05 | FAM83F | 179 | 0.388141966322352 |  |  |  |
| FAM83D | 693 | 1.54795422395349e-05 | FAM83F | 180 | 0.344783215416015 |  |  |  |
| FAM83D | 695 | 2.1761303638718e-05 | FAM83F | 181 | 0.313958475610274 |  |  |  |
| FAM83D | 695 | 1.57034398180448e-05 | FAM83F | 182 | 0.372603460901182 |  |  |  |
| FAM83D | 697 | 1.24372498931338e-05 | FAM83H | 795 | 0.691385475419035 |  |  |  |
| FAM83D | 701 | 1.10758731599011e-05 | FAM83H | 801 | 0.750685664246301 |  |  |  |
| FAM83D | 701 | 1.35502579850322e-05 | FAM83H | 802 | 0.644434625470011 |  |  |  |
| FAM83D | 701 | 1.04649993077496e-05 | FAM83H | 803 | 0.705898293887072 |  |  |  |
| FAM83D | 702 | 7.87929904904039e-06 | FAM83H | 804 | 0.781628542022705 |  |  |  |
| FAM83D | 704 | 8.17279918227697e-06 | FAM83H | 810 | 0.816753547567311 |  |  |  |
| FAM83D | 704 | 1.07741198768626e-05 | FAM83H | 810 | 0.858485701456213 |  |  |  |
| FAM83D | 706 | 8.55358685115626e-06 | FAM83H | 811 | 0.920503659467565 |  |  |  |
| FAM83D | 708 | 6.4866910339091e-06 | FAM83H | 817 | 0.967882395583546 |  |  |  |
| FAM83D | 708 | 8.59673439246279e-06 | FAM83H | 821 | 0.973697750998242 |  |  |  |
| FAM83D | 710 | 1.09131152127328e-05 | FAM83H | 823 | 0.978656711242299 |  |  |  |
| FAM83D | 713 | 8.66200858546388e-06 | FAM83H | 824 | 0.914155601095988 |  |  |  |
| FAM83D | 714 | 1.07926925763727e-05 | FAM83H | 830 | 0.867431664382356 |  |  |  |
| FAM83D | 714 | 9.53812179871166e-06 | FAM83H | 830 | 0.946088283848092 |  |  |  |
| FAM83D | 715 | 1.12344743425256e-05 | FAM83H | 831 | 0.892099665856367 |  |  |  |
| FAM83D | 717 | 1.4379478268639e-05 | FAM83H | 831 | 0.833739140154326 |  |  |  |
| FAM83D | 717 | 2.00356515659749e-05 | FAM83H | 832 | 0.917944539142493 |  |  |  |
| FAM83D | 720 | 1.51657305447187e-05 | FAM83H | 840 | 0.863584410293714 |  |  |  |
| FAM83D | 722 | 1.33859387562474e-05 | FAM83H | 846 | 0.803326385707161 |  |  |  |
| FAM83D | 722 | 1.89017067901449e-05 | FAM83H | 848 | 0.74837300577835 |  |  |  |
| FAM83D | 725 | 1.30780892348569e-05 | FAM83H | 850 | 0.686623031111179 |  |  |  |
| FAM83D | 728 | 1.67502140968472e-05 | FAM83H | 851 | 0.644915621792128 |  |  |  |
| FAM83D | 728 | 2.1429060011731e-05 | FAM83H | 852 | 0.61504976711436 |  |  |  |
| FAM83D | 730 | 2.76829918981343e-05 | FAM83H | 860 | 0.563915747322553 |  |  |  |
| FAM83D | 731 | 3.68409977740029e-05 | FAM83H | 861 | 0.639144250762193 |  |  |  |
| FAM83D | 732 | 2.96524059656864e-05 | FAM83H | 864 | 0.582028008502356 |  |  |  |
| FAM83D | 732 | 2.58673641616271e-05 | FAM83H | 868 | 0.681604730023557 |  |  |  |
| FAM83D | 733 | 2.35147866320809e-05 | FAM83H | 876 | 0.622897296144176 |  |  |  |
| FAM83D | 733 | 1.99684988345609e-05 | FAM83H | 877 | 0.619312576041556 |  |  |  |
| FAM83D | 735 | 1.56381027291346e-05 | FAM83H | 880 | 0.705296093773108 |  |  |  |
| FAM83D | 744 | 1.18042173036956e-05 | FAM83H | 886 | 0.69878812131892 |  |  |  |
| FAM83D | 744 | 1.52458151999936e-05 | FAM83H | 887 | 0.657618558073267 |  |  |  |
| FAM83D | 746 | 2.03407894711746e-05 | FAM83H | 888 | 0.625006244884821 |  |  |  |
| FAM83D | 752 | 2.25202961723169e-05 | FAM83H | 888 | 0.688593129851031 |  |  |  |
| FAM83D | 752 | 3.22975142624181e-05 | FAM83H | 891 | 0.639959582279955 |  |  |  |
| FAM83D | 753 | 2.866772619737e-05 | FAM83H | 892 | 0.588613349979142 |  |  |  |
| FAM83D | 754 | 2.19839302969242e-05 | FAM83H | 892 | 0.677088379170913 |  |  |  |
| FAM83D | 754 | 3.07905682606283e-05 | FAM83H | 897 | 0.623508114864886 |  |  |  |
| FAM83D | 755 | 2.6946211227096e-05 | FAM83H | 901 | 0.577780467122614 |  |  |  |
| FAM83D | 757 | 3.65995826677466e-05 | FAM83H | 903 | 0.53055933771744 |  |  |  |
| FAM83D | 758 | 4.40446683908448e-05 | FAM83H | 906 | 0.508231672008119 |  |  |  |
| FAM83D | 759 | 3.71063959645808e-05 | FAM83H | 911 | 0.473882845935302 |  |  |  |
| FAM83D | 760 | 4.43947442952481e-05 | FAM83H | 916 | 0.431247342299764 |  |  |  |
| FAM83D | 761 | 6.04005677769617e-05 | FAM83H | 919 | 0.485065615379213 |  |  |  |
| FAM83D | 761 | 5.01093825973507e-05 | FAM83H | 921 | 0.561258749416004 |  |  |  |
| FAM83D | 762 | 4.0575150940238e-05 | FAM83H | 923 | 0.513840404972639 |  |  |  |
| FAM83D | 763 | 3.25477864119245e-05 | FAM83H | 923 | 0.464276379924113 |  |  |  |
| FAM83D | 763 | 2.54059938689483e-05 | FAM83H | 926 | 0.438548990762722 |  |  |  |
| FAM83D | 763 | 3.29584550544457e-05 | FAM83H | 932 | 0.523646803723007 |  |  |  |
| FAM83D | 766 | 4.69562321197209e-05 | FAM83H | 934 | 0.587378540678669 |  |  |  |
| FAM83D | 766 | 5.4559575663053e-05 | FAM83H | 935 | 0.663956024326422 |  |  |  |
| FAM83D | 767 | 4.51004038918903e-05 | FAM83H | 936 | 0.768892749773425 |  |  |  |
| FAM83D | 768 | 6.0268790622628e-05 | FAM83H | 937 | 0.880796032638189 |  |  |  |
| FAM83D | 768 | 7.54118240120619e-05 | FAM83H | 937 | 0.823095922221442 |  |  |  |
| FAM83D | 768 | 8.93025339197212e-05 | FAM83H | 941 | 0.770804640390797 |  |  |  |
| FAM83D | 772 | 0.00011530933180433 | FAM83H | 945 | 0.865663063483009 |  |  |  |
| FAM83D | 778 | 9.36382157422175e-05 | FAM83H | 949 | 0.809508531372949 |  |  |  |
| FAM83D | 780 | 0.000119287170512522 | FAM83H | 950 | 0.778545432478331 |  |  |  |
| FAM83D | 781 | 9.62738154969618e-05 | FAM83H | 952 | 0.723224223881625 |  |  |  |
| FAM83D | 782 | 0.000132937194103277 | FAM83H | 952 | 0.819005588711928 |  |  |  |
| FAM83D | 783 | 9.74264556854493e-05 | FAM83H | 957 | 0.924013844139879 |  |  |  |
| FAM83D | 785 | 0.0001281590548592 | FAM83H | 960 | 0.867325173558865 |  |  |  |
| FAM83D | 785 | 0.00016542276050335 | FAM83H | 966 | 0.826921602244258 |  |  |  |
| FAM83D | 787 | 0.000131713977148709 | FAM83H | 968 | 0.778884839544219 |  |  |  |
| FAM83D | 787 | 0.000166816573923168 | FAM83H | 970 | 0.737271157820122 |  |  |  |
| FAM83D | 790 | 0.000189914622071096 | FAM83H | 970 | 0.809918491144151 |  |  |  |
| FAM83D | 791 | 0.000240007942704217 | FAM83H | 970 | 0.880285392652312 |  |  |  |
| FAM83D | 791 | 0.000289389871170565 | FAM83H | 971 | 0.946290650463414 |  |  |  |
| FAM83D | 791 | 0.000258421192173282 | FAM83H | 975 | 0.889832492910612 |  |  |  |
| FAM83D | 793 | 0.000179796486838016 | FAM83H | 975 | 0.855199268565309 |  |  |  |
| FAM83D | 795 | 0.000242502504258495 | FAM83H | 977 | 0.9358182167316 |  |  |  |
| FAM83D | 797 | 0.000183573071844569 | FAM83H | 987 | 0.959460058531568 |  |  |  |
| FAM83D | 797 | 0.000249407650065141 | FAM83H | 997 | 0.978029753211399 |  |  |  |
| FAM83D | 798 | 0.000202921893407301 | FAM83H | 998 | 0.921599737818467 |  |  |  |
| FAM83D | 801 | 0.000153064679264319 | FAM83H | 999 | 0.87925249558164 |  |  |  |
| FAM83D | 802 | 0.000207982025311398 | FAM83H | 100 | 0.830148021716332 |  |  |  |
| FAM83D | 803 | 0.000251503404308333 | FAM83H | 100 | 0.937689727512886 |  |  |  |
| FAM83D | 804 | 0.000325188179924435 | FAM83H | 101 | 0.88710818046273 |  |  |  |
| FAM83D | 809 | 0.000360570926913791 | FAM83H | 101 | 0.982369867928584 |  |  |  |
| FAM83D | 811 | 0.00025467368190817 | FAM83H | 101 | 0.927701015247844 |  |  |  |
| FAM83D | 814 | 0.000185517314901441 | FAM83H | 101 | 0.87998677778817 |  |  |  |
| FAM83D | 816 | 0.000217446413316558 | FAM83H | 102 | 0.826389113658836 |  |  |  |
| FAM83D | 817 | 0.000178233232859802 | FAM83H | 102 | 0.789596432686612 |  |  |  |
| FAM83D | 817 | 0.000218066895451466 | FAM83H | 102 | 0.88016452719776 |  |  |  |
| FAM83D | 821 | 0.0001932728887361 | FAM83H | 103 | 0.822572554320579 |  |  |  |
| FAM83D | 823 | 0.000168444794437618 | FAM83H | 103 | 0.913126283237204 |  |  |  |
| FAM83D | 825 | 0.000207213526068767 | FAM83H | 103 | 0.985237330856853 |  |  |  |
| FAM83D | 829 | 0.000165055893324304 | FAM83H | 104 | 0.944478434686103 |  |  |  |
| FAM83D | 834 | 0.00022253299902485 | FAM83H | 104 | 0.910320202634878 |  |  |  |
| FAM83D | 834 | 0.000170573804497772 | FAM83H | 104 | 0.868211844713997 |  |  |  |
| FAM83D | 836 | 0.000237332052239701 | FAM83H | 105 | 0.817865051361445 |  |  |  |
| FAM83D | 836 | 0.000212764147144672 | FAM83H | 105 | 0.894575067827897 |  |  |  |
| FAM83D | 838 | 0.0002017838076317 | FAM83H | 105 | 0.837108686425369 |  |  |  |
| FAM83D | 839 | 0.000235575629346812 | FAM83H | 106 | 0.810070621314801 |  |  |  |
| FAM83D | 839 | 0.000193084515408751 | FAM83H | 106 | 0.877023099197343 |  |  |  |
| FAM83D | 840 | 0.000168255803022917 | FAM83H | 107 | 0.835389468872632 |  |  |  |
| FAM83D | 841 | 0.000233723370568285 | FAM83H | 107 | 0.887944536410272 |  |  |  |
| FAM83D | 842 | 0.000185872813340256 | FAM83H | 108 | 0.833379440597706 |  |  |  |
| FAM83D | 846 | 0.000163008393387607 | FAM83H | 108 | 0.777129289573994 |  |  |  |
| FAM83D | 850 | 0.0001336329478222 | FAM83H | 108 | 0.72657862739847 |  |  |  |
| FAM83D | 851 | 0.000177367540001932 | FAM83H | 108 | 0.798123632362633 |  |  |  |
| FAM83D | 852 | 0.000144413543783443 | FAM83H | 108 | 0.759814840871952 |  |  |  |
| FAM83D | 853 | 0.000176807852575221 | FAM83H | 109 | 0.72062715491616 |  |  |  |
| FAM83D | 854 | 0.00022548253110416 | FAM83H | 109 | 0.667202063246935 |  |  |  |
| FAM83D | 855 | 0.000167631101854266 | FAM83H | 109 | 0.629387246892776 |  |  |  |
| FAM83D | 856 | 0.000228612025874361 | FAM83H | 110 | 0.580386333052033 |  |  |  |
| FAM83D | 856 | 0.000212905055073274 | FAM83H | 110 | 0.534193199500958 |  |  |  |
| FAM83D | 858 | 0.000274934571722802 | FAM83H | 111 | 0.49114782849825 |  |  |  |
| FAM83D | 860 | 0.000311530645662666 | FAM83H | 111 | 0.552369617230228 |  |  |  |
| FAM83D | 864 | 0.000356995030525455 | FAM83H | 111 | 0.638230062544041 |  |  |  |
| FAM83D | 866 | 0.00029714994518398 | FAM83H | 111 | 0.600932999198096 |  |  |  |
| FAM83D | 871 | 0.000243433588047329 | FAM83H | 112 | 0.675424052410155 |  |  |  |
| FAM83D | 871 | 0.000331514717233781 | FAM83H | 112 | 0.633300469520103 |  |  |  |
| FAM83D | 878 | 0.000271709863346661 | FAM83H | 112 | 0.720298818708961 |  |  |  |
| FAM83D | 881 | 0.00017570855913837 | FAM83H | 112 | 0.796501657038531 |  |  |  |
| FAM83D | 883 | 0.000222169205175398 | FAM83H | 112 | 0.750622240207565 |  |  |  |
| FAM83D | 885 | 0.000237628446323486 | FAM83H | 112 | 0.703026948490407 |  |  |  |
| FAM83D | 885 | 0.000286177391711743 | FAM83H | 113 | 0.650373735274662 |  |  |  |
| FAM83D | 887 | 0.0003764119983634 | FAM83H | 114 | 0.603046580368993 |  |  |  |
| FAM83D | 891 | 0.00051650454227608 | FAM83H | 114 | 0.55144134515679 |  |  |  |
| FAM83D | 898 | 0.000585826535019283 | FAM83H | 114 | 0.523192052129585 |  |  |  |
| FAM83D | 900 | 0.000774686511497741 | FAM83H | 115 | 0.5956546035535 |  |  |  |
| FAM83D | 900 | 0.000598537386588778 | FAM83H | 115 | 0.682292566351619 |  |  |  |
| FAM83D | 901 | 0.000565502603554586 | FAM83H | 115 | 0.638619717615963 |  |  |  |
| FAM83D | 909 | 0.000414622915911476 | FAM83H | 115 | 0.735812599470683 |  |  |  |
| FAM83D | 915 | 0.00034035522378739 | FAM83H | 115 | 0.695455316271924 |  |  |  |
| FAM83D | 917 | 0.000388733448743045 | FAM83H | 116 | 0.777002385774037 |  |  |  |
| FAM83D | 919 | 0.0002602453199699 | FAM83H | 116 | 0.864187256835354 |  |  |  |
| FAM83D | 920 | 0.000329037177505659 | FAM83H | 116 | 0.818632077753739 |  |  |  |
| FAM83D | 920 | 0.000436041083269941 | FAM83H | 116 | 0.771693491099742 |  |  |  |
| FAM83D | 921 | 0.000537264880577429 | FAM83H | 116 | 0.756725929062774 |  |  |  |
| FAM83D | 922 | 0.000672057207151619 | FAM83H | 117 | 0.702718396654475 |  |  |  |
| FAM83D | 923 | 0.000550721175620423 | FAM83H | 117 | 0.668580638339562 |  |  |  |
| FAM83D | 925 | 0.000376708425125686 | FAM83H | 119 | 0.77044662724154 |  |  |  |
| FAM83D | 926 | 0.000449567286826227 | FAM83H | 119 | 0.739736736684628 |  |  |  |
| FAM83D | 928 | 0.000592701924915495 | FAM83H | 119 | 0.799301181846253 |  |  |  |
| FAM83D | 928 | 0.000772463380313117 | FAM83H | 119 | 0.764759710965845 |  |  |  |
| FAM83D | 929 | 0.000664005825106426 | FAM83H | 119 | 0.845244073098652 |  |  |  |
| FAM83D | 929 | 0.000461153880678356 | FAM83H | 119 | 0.804206899435411 |  |  |  |
| FAM83D | 929 | 0.000581564561108123 | FAM83H | 120 | 0.759156670914787 |  |  |  |
| FAM83D | 931 | 0.000484787674013419 | FAM83H | 120 | 0.714531835973534 |  |  |  |
| FAM83D | 935 | 0.000405459961829118 | FAM83H | 120 | 0.774596511305784 |  |  |  |
| FAM83D | 936 | 0.000270461927822168 | FAM83H | 120 | 0.720184356361242 |  |  |  |
| FAM83D | 936 | 0.000209949863687652 | FAM83H | 121 | 0.778782096444397 |  |  |  |
| FAM83D | 942 | 0.000185289591102229 | FAM83H | 122 | 0.725745797769117 |  |  |  |
| FAM83D | 942 | 0.000213766616879569 | FAM83H | 122 | 0.811741572730675 |  |  |  |
| FAM83D | 944 | 0.000185886618423704 | FAM83H | 122 | 0.908622178378144 |  |  |  |
| FAM83D | 945 | 0.000217502551303406 | FAM83H | 123 | 0.867325622656832 |  |  |  |
| FAM83D | 952 | 0.000177117709867457 | FAM83H | 123 | 0.956010664357903 |  |  |  |
| FAM83D | 957 | 0.000218376103299216 | FAM83H | 123 | 0.9004655523118 |  |  |  |
| FAM83D | 959 | 0.000170484858274643 | FAM83H | 123 | 0.843869508018701 |  |  |  |
| FAM83D | 961 | 0.000193135540604598 | FAM83H | 124 | 0.790615582161836 |  |  |  |
| FAM83D | 966 | 0.000260764622550358 | FAM83H | 124 | 0.739714012392767 |  |  |  |
| FAM83D | 967 | 0.000222688068058856 | FAM83H | 124 | 0.834983404012514 |  |  |  |
| FAM83D | 968 | 0.000289543704826488 | FAM83H | 124 | 0.780824820001796 |  |  |  |
| FAM83D | 969 | 0.000373895367753117 | FAM83H | 125 | 0.730102170576218 |  |  |  |
| FAM83D | 970 | 0.000321975462840001 | FAM83H | 126 | 0.678174246073839 |  |  |  |
| FAM83D | 971 | 0.000249664265388484 | FAM83H | 126 | 0.629356086293934 |  |  |  |
| FAM83D | 972 | 0.000224323354803019 | FAM83H | 126 | 0.581421535508352 |  |  |  |
| FAM83D | 972 | 0.000271852244683241 | FAM83H | 127 | 0.547100435160455 |  |  |  |
| FAM83D | 973 | 0.000218977322767688 | FAM83H | 127 | 0.500204242896904 |  |  |  |
| FAM83D | 974 | 0.000293348324954496 | FAM83H | 127 | 0.56321131652953 |  |  |  |
| FAM83D | 976 | 0.000346837180926614 | FAM83H | 128 | 0.521597895798473 |  |  |  |
| FAM83D | 979 | 0.000398932036951942 | FAM83H | 128 | 0.475821939172752 |  |  |  |
| FAM83D | 980 | 0.000440129252545721 | FAM83H | 128 | 0.439165094548256 |  |  |  |
| FAM83D | 981 | 0.00060162857817183 | FAM83H | 128 | 0.400025792024782 |  |  |  |
| FAM83D | 986 | 0.000470819014445602 | FAM83H | 129 | 0.358837278584096 |  |  |  |
| FAM83D | 989 | 0.000522513417591613 | FAM83H | 129 | 0.40279041833704 |  |  |  |
| FAM83D | 990 | 0.000412961126618288 | FAM83H | 129 | 0.478355843746656 |  |  |  |
| FAM83D | 991 | 0.00055576234309388 | FAM83H | 130 | 0.445245284933322 |  |  |  |
| FAM83D | 992 | 0.000459496023491682 | FAM83H | 131 | 0.520245695101005 |  |  |  |
| FAM83D | 992 | 0.000538183815067463 | FAM83H | 131 | 0.59685243749521 |  |  |  |
| FAM83D | 993 | 0.000678264921656223 | FAM83H | 132 | 0.55110717326426 |  |  |  |
| FAM83D | 995 | 0.000823156129822055 | FAM83H | 133 | 0.507112923451204 |  |  |  |
| FAM83D | 995 | 0.00081141078406735 | FAM83H | 133 | 0.486632037601313 |  |  |  |
| FAM83D | 996 | 0.000675775181391579 | FAM83H | 134 | 0.573749004250094 |  |  |  |
| FAM83D | 100 | 0.000539807281816809 | FAM83H | 134 | 0.639844857238328 |  |  |  |
| FAM83D | 100 | 0.000435302066345721 | FAM83H | 134 | 0.703955952144331 |  |  |  |
| FAM83D | 100 | 0.000559025822655536 | FAM83H | 134 | 0.654688923495249 |  |  |  |
| FAM83D | 101 | 0.000453185388676787 | FAM83H | 135 | 0.606828091892785 |  |  |  |
| FAM83D | 101 | 0.000409235681466816 | FAM83H | 135 | 0.570678475328569 |  |  |  |
| FAM83D | 101 | 0.000498292334101699 | FAM83H | 135 | 0.535819808501447 |  |  |  |
| FAM83D | 101 | 0.000617017155351163 | FAM83H | 135 | 0.491082333710814 |  |  |  |
| FAM83D | 101 | 0.00050700650702058 | FAM83H | 136 | 0.552434516131535 |  |  |  |
| FAM83D | 101 | 0.000505033219837991 | FAM83H | 136 | 0.504779039638646 |  |  |  |
| FAM83D | 101 | 0.000436450643619309 | FAM83H | 137 | 0.461354995012665 |  |  |  |
| FAM83D | 101 | 0.000320642062841915 | FAM83H | 137 | 0.437936358120204 |  |  |  |
| FAM83D | 101 | 0.000268155470382747 | FAM83H | 138 | 0.503448960611057 |  |  |  |
| FAM83D | 101 | 0.000219092597804705 | FAM83H | 139 | 0.591841288983754 |  |  |  |
| FAM83D | 102 | 0.000181263238725817 | FAM83H | 140 | 0.551634453730733 |  |  |  |
| FAM83D | 102 | 0.000231531752506661 | FAM83H | 141 | 0.503848001749506 |  |  |  |
| FAM83D | 102 | 0.000318881934975063 | FAM83H | 141 | 0.45947693867682 |  |  |  |
| FAM83D | 102 | 0.000347335734422908 | FAM83H | 142 | 0.517074777270708 |  |  |  |
| FAM83D | 103 | 0.000474637112277833 | FAM83H | 142 | 0.591941300826263 |  |  |  |
| FAM83D | 103 | 0.000512075077753231 | FAM83H | 142 | 0.542300206556113 |  |  |  |
| FAM83D | 103 | 0.000402595726500438 | FAM83H | 144 | 0.49750605807339 |  |  |  |
| FAM83D | 103 | 0.00051031986021152 | FAM83H | 144 | 0.455231212482132 |  |  |  |
| FAM83D | 104 | 0.000680456893717417 | FAM83H | 144 | 0.409830610582843 |  |  |  |
| FAM83D | 104 | 0.000527775843044869 | FAM83H | 144 | 0.37096189739898 |  |  |  |
| FAM83D | 104 | 0.000434119884130892 | FAM83H | 144 | 0.330815641704992 |  |  |  |
| FAM83D | 104 | 0.00054241738168781 | FAM83H | 145 | 0.30621688124987 |  |  |  |
| FAM83D | 104 | 0.00069392323844165 | FAM83H | 145 | 0.36815712688565 |  |  |  |
| FAM83D | 105 | 0.000820647530337114 | FAM83H | 145 | 0.438029564982343 |  |  |  |
| FAM83D | 105 | 0.000678375983960715 | FAM83H | 146 | 0.393417176571986 |  |  |  |
| FAM83D | 105 | 0.000890770831208311 | FAM83H | 146 | 0.346123888210885 |  |  |  |
| FAM83D | 105 | 0.00121390853477219 | FAM83H | 146 | 0.311887855973638 |  |  |  |
| FAM83D | 105 | 0.00139852822212132 | FAM83H | 146 | 0.284511762345056 |  |  |  |
| FAM83D | 105 | 0.00185576079923684 | FAM83H | 148 | 0.337391862448722 |  |  |  |
| FAM83D | 105 | 0.00216563105278663 | FAM83H | 148 | 0.402958627096732 |  |  |  |
| FAM83D | 106 | 0.00239650561215699 | FAM83H | 148 | 0.462980667646683 |  |  |  |
| FAM83D | 106 | 0.00171999425534079 | FAM83H | 149 | 0.429532230256807 |  |  |  |
| FAM83D | 106 | 0.00220072910375515 | FAM83H | 149 | 0.495926780098293 |  |  |  |
| FAM83D | 106 | 0.00244929919565218 | FAM83H | 150 | 0.587669409934123 |  |  |  |
| FAM83D | 107 | 0.00306896158713197 | FAM83H | 151 | 0.635945708342482 |  |  |  |
| FAM83D | 107 | 0.00301458230641401 | FAM83H | 151 | 0.586142547591764 |  |  |  |
| FAM83D | 107 | 0.00339562833733511 | FAM83H | 152 | 0.551273031725509 |  |  |  |
| FAM83D | 107 | 0.00420485551600324 | FAM83H | 152 | 0.493125193261636 |  |  |  |
| FAM83D | 107 | 0.00364690389771914 | FAM83H | 152 | 0.461354726540044 |  |  |  |
| FAM83D | 107 | 0.00439768092939276 | FAM83H | 152 | 0.426598262579145 |  |  |  |
| FAM83D | 108 | 0.00372341781121573 | FAM83H | 154 | 0.398594294490269 |  |  |  |
| FAM83D | 108 | 0.00492757358198098 | FAM83H | 155 | 0.446513191272117 |  |  |  |
| FAM83D | 108 | 0.00605090384639838 | FAM83H | 155 | 0.408614387544095 |  |  |  |
| FAM83D | 108 | 0.00551807608988157 | FAM83H | 155 | 0.36497211639653 |  |  |  |
| FAM83D | 108 | 0.00713586294046583 | FAM83H | 155 | 0.331013470125442 |  |  |  |
| FAM83D | 109 | 0.0079919499902144 | FAM83H | 156 | 0.3893324180106 |  |  |  |
| FAM83D | 109 | 0.0090432309219928 | FAM83H | 156 | 0.369161302939933 |  |  |  |
| FAM83D | 109 | 0.0111270021943332 | FAM83H | 156 | 0.352665963535298 |  |  |  |
| FAM83D | 109 | 0.00866083847115741 | FAM83H | 156 | 0.319633760316094 |  |  |  |
| FAM83D | 109 | 0.00731482231982585 | FAM83H | 157 | 0.288604645665502 |  |  |  |
| FAM83D | 110 | 0.00758113226130342 | FAM83H | 158 | 0.256626564543131 |  |  |  |
| FAM83D | 110 | 0.00682289347576673 | FAM83H | 158 | 0.314534209320222 |  |  |  |
| FAM83D | 110 | 0.00487947512903521 | FAM83H | 159 | 0.279923876015876 |  |  |  |
| FAM83D | 111 | 0.00353710032036107 | FAM83H | 159 | 0.342357931821461 |  |  |  |
| FAM83D | 111 | 0.00350067420814667 | FAM83H | 159 | 0.319187814251738 |  |  |  |
| FAM83D | 112 | 0.00415752004663328 | FAM83H | 160 | 0.393898258392374 |  |  |  |
| FAM83D | 112 | 0.00392200434166253 | FAM83H | 160 | 0.470006506288447 |  |  |  |
| FAM83D | 112 | 0.00291816034833992 | FAM83H | 161 | 0.559889067749661 |  |  |  |
| FAM83D | 113 | 0.00274763979904305 | FAM83H | 161 | 0.517934373368477 |  |  |  |
| FAM83D | 113 | 0.00194860990269007 | FAM83H | 161 | 0.477547444638083 |  |  |  |
| FAM83D | 113 | 0.00215683236385856 | FAM83H | 162 | 0.44010161030742 |  |  |  |
| FAM83D | 113 | 0.00176750461626354 | FAM83H | 162 | 0.529073627071815 |  |  |  |
| FAM83D | 113 | 0.00239361460187524 | FAM83H | 162 | 0.47752250898571 |  |  |  |
| FAM83D | 113 | 0.00319430794988694 | FAM83H | 162 | 0.55270766371679 |  |  |  |
| FAM83D | 113 | 0.00271392470462092 | FAM83H | 162 | 0.603759224677397 |  |  |  |
| FAM83D | 113 | 0.00312929410997226 | FAM83H | 163 | 0.559076321366409 |  |  |  |
| FAM83D | 114 | 0.00266283828352434 | FAM83H | 163 | 0.508256877784877 |  |  |  |
| FAM83D | 114 | 0.00287301393733113 | FAM83H | 163 | 0.579716874054383 |  |  |  |
| FAM83D | 114 | 0.00370814673618139 | FAM83H | 163 | 0.530852595371128 |  |  |  |
| FAM83D | 114 | 0.00237361489701253 | FAM83H | 163 | 0.584663703711965 |  |  |  |
| FAM83D | 114 | 0.00297299899382705 | FAM83H | 164 | 0.583455693962491 |  |  |  |
| FAM83D | 114 | 0.00393232625766607 | FAM83H | 164 | 0.534881703105375 |  |  |  |
| FAM83D | 114 | 0.00316793116666893 | FAM83H | 164 | 0.526215793572552 |  |  |  |
| FAM83D | 114 | 0.00400602792645238 | FAM83H | 164 | 0.620104029307414 |  |  |  |
| FAM83D | 115 | 0.00327450124567534 | FAM83H | 164 | 0.716268650846003 |  |  |  |
| FAM83D | 115 | 0.00420522783776226 | FAM83H | 165 | 0.685532872911821 |  |  |  |
| FAM83D | 115 | 0.00496100107500774 | FAM83H | 166 | 0.795617378746755 |  |  |  |
| FAM83D | 115 | 0.00578428567597793 | FAM83H | 166 | 0.741229435276155 |  |  |  |
| FAM83D | 116 | 0.00480770587256676 | FAM83H | 166 | 0.824238945333006 |  |  |  |
| FAM83D | 116 | 0.00619070257142469 | FAM83H | 166 | 0.890027305865036 |  |  |  |
| FAM83D | 118 | 0.00545076294986837 | FAM83H | 167 | 0.997759386033602 |  |  |  |
| FAM83D | 118 | 0.00452683523054906 | FAM83H | 167 | 0.944945234847439 |  |  |  |
| FAM83D | 118 | 0.00567564058582123 | FAM83H | 168 | 0.941397398898471 |  |  |  |
| FAM83D | 119 | 0.00722931890882455 | FAM83H | 168 | 0.99460432889648 |  |  |  |
| FAM83D | 119 | 0.00587325323178641 | FAM83H | 169 | 0.892598160558224 |  |  |  |
| FAM83D | 119 | 0.00703805150508446 | FAM83H | 169 | 0.793241371331988 |  |  |  |
| FAM83D | 119 | 0.00915063036488162 | FAM83H | 169 | 0.865525940112355 |  |  |  |
| FAM83D | 119 | 0.00766994028150882 | FAM83H | 170 | 0.768308518625364 |  |  |  |
| FAM83D | 119 | 0.00888508785649883 | FAM83H | 170 | 0.820273583329477 |  |  |  |
| FAM83D | 120 | 0.0086335724351866 | FAM83H | 171 | 0.865928400339631 |  |  |  |
| FAM83D | 120 | 0.0107082509481614 | FAM83H | 171 | 0.80137866779545 |  |  |  |
| FAM83D | 120 | 0.00815480250187826 | FAM83H | 171 | 0.701506693610259 |  |  |  |
| FAM83D | 121 | 0.00932895535562708 | FAM83H | 171 | 0.746316416501174 |  |  |  |
| FAM83D | 121 | 0.0110147436224534 | FAM83H | 171 | 0.653805921271418 |  |  |  |
| FAM83D | 121 | 0.00948458912162947 | FAM83H | 172 | 0.661523594048918 |  |  |  |
| FAM83D | 121 | 0.0117746006133921 | FAM83H | 172 | 0.717240098864902 |  |  |  |
| FAM83D | 121 | 0.00783972063958441 | FAM83H | 172 | 0.779520102258714 |  |  |  |
| FAM83D | 122 | 0.00951187714425303 | FAM83H | 172 | 0.837539298868494 |  |  |  |
| FAM83D | 122 | 0.0109433230886726 | FAM83H | 173 | 0.902860377342172 |  |  |  |
| FAM83D | 122 | 0.0130089860055782 | FAM83H | 173 | 0.962992420724333 |  |  |  |
| FAM83D | 122 | 0.0158586112682003 | FAM83H | 173 | 0.870164955912827 |  |  |  |
| FAM83D | 122 | 0.0135913669787269 | FAM83H | 174 | 0.92237801384441 |  |  |  |
| FAM83D | 123 | 0.0138167807372171 | FAM83H | 175 | 0.974240447341867 |  |  |  |
| FAM83D | 124 | 0.0161055212092628 | FAM83H | 176 | 0.963541117761633 |  |  |  |
| FAM83D | 124 | 0.0167812758554889 | FAM83H | 177 | 0.896158077683466 |  |  |  |
| FAM83D | 125 | 0.0198452785149565 | FAM83H | 177 | 0.862622167367261 |  |  |  |
| FAM83D | 126 | 0.024074262141222 | FAM83H | 177 | 0.810778639353795 |  |  |  |
| FAM83D | 126 | 0.0194950296632057 | FAM83H | 178 | 0.912372915980693 |  |  |  |
| FAM83D | 126 | 0.0141205304172539 | FAM83H | 179 | 0.982274004067242 |  |  |  |
| FAM83D | 126 | 0.0157996995395992 | FAM83H | 180 | 0.957681105846267 |  |  |  |
| FAM83D | 126 | 0.0129892958145429 | FAM83H | 180 | 0.92649604453869 |  |  |  |
| FAM83D | 127 | 0.0163064203776111 | FAM83H | 181 | 0.988168208451809 |  |  |  |
| FAM83D | 128 | 0.0132770650317012 | FAM83H | 181 | 0.95142210586836 |  |  |  |
| FAM83D | 128 | 0.0164383593713911 | FAM83H | 181 | 0.897281354988945 |  |  |  |
| FAM83D | 128 | 0.0109995127158102 | FAM83H | 182 | 0.987689865639625 |  |  |  |
| FAM83D | 128 | 0.0142131009077998 | FAM83H | 182 | 0.964711898370497 |  |  |  |
| FAM83D | 128 | 0.0167502845279256 | FAM83H | 182 | 0.903858274862438 |  |  |  |
| FAM83D | 129 | 0.0208393737418195 | FAM83H | 183 | 0.973244380787119 |  |  |  |
| FAM83D | 129 | 0.0187363729976702 | FAM83H | 183 | 0.960946992401756 |  |  |  |
| FAM83D | 129 | 0.0175853355665966 | FAM83H | 185 | 0.898019900870476 |  |  |  |
| FAM83D | 130 | 0.0155938751415252 |  |  |  |  |  |  |
| FAM83D | 131 | 0.0134297585767726 |  |  |  |  |  |  |
| FAM83D | 131 | 0.0114537559777896 |  |  |  |  |  |  |
| FAM83D | 131 | 0.0149828470984772 |  |  |  |  |  |  |
| FAM83D | 132 | 0.0165868543400248 |  |  |  |  |  |  |
| FAM83D | 132 | 0.0210751333269624 |  |  |  |  |  |  |
| FAM83D | 132 | 0.0264023134816307 |  |  |  |  |  |  |
| FAM83E | 8 | 0.128835410867297 |  |  |  |  |  |  |
| FAM83E | 8 | 0.13876955263741 |  |  |  |  |  |  |
| FAM83E | 8 | 0.149016076685475 |  |  |  |  |  |  |
| FAM83E | 8 | 0.140931277507458 |  |  |  |  |  |  |
| FAM83E | 8 | 0.153001402806589 |  |  |  |  |  |  |
| FAM83E | 8 | 0.137872887189837 |  |  |  |  |  |  |
| FAM83E | 8 | 0.151535308242729 |  |  |  |  |  |  |
| FAM83E | 8 | 0.141171368105599 |  |  |  |  |  |  |
| FAM83E | 8 | 0.161086607521739 |  |  |  |  |  |  |
| FAM83E | 8 | 0.171462318104468 |  |  |  |  |  |  |
| FAM83E | 8 | 0.182880243559744 |  |  |  |  |  |  |
| FAM83E | 8 | 0.192432508539423 |  |  |  |  |  |  |
| FAM83E | 8 | 0.208852163268602 |  |  |  |  |  |  |
| FAM83E | 8 | 0.229824708271521 |  |  |  |  |  |  |
| FAM83E | 8 | 0.250975662575777 |  |  |  |  |  |  |
| FAM83E | 8 | 0.271471166320219 |  |  |  |  |  |  |
| FAM83E | 8 | 0.292868125636555 |  |  |  |  |  |  |
| FAM83E | 8 | 0.261134937106088 |  |  |  |  |  |  |
| FAM83E | 8 | 0.292134169588428 |  |  |  |  |  |  |
| FAM83E | 8 | 0.261460911137072 |  |  |  |  |  |  |
| FAM83E | 8 | 0.264112081014249 |  |  |  |  |  |  |
| FAM83E | 8 | 0.265031330747896 |  |  |  |  |  |  |
| FAM83E | 8 | 0.28621756820764 |  |  |  |  |  |  |
| FAM83E | 8 | 0.262922232401626 |  |  |  |  |  |  |
| FAM83E | 8 | 0.244609240354159 |  |  |  |  |  |  |
| FAM83E | 8 | 0.221350753115919 |  |  |  |  |  |  |
| FAM83E | 8 | 0.196445166399269 |  |  |  |  |  |  |
| FAM83E | 8 | 0.192085271917952 |  |  |  |  |  |  |
| FAM83E | 8 | 0.207407951441049 |  |  |  |  |  |  |
| FAM83E | 8 | 0.225356020309664 |  |  |  |  |  |  |
| FAM83E | 8 | 0.238016315315894 |  |  |  |  |  |  |
| FAM83E | 8 | 0.239313245075027 |  |  |  |  |  |  |
| FAM83E | 8 | 0.221830488702965 |  |  |  |  |  |  |
| FAM83E | 9 | 0.197039095625808 |  |  |  |  |  |  |
| FAM83E | 9 | 0.174925704805805 |  |  |  |  |  |  |
| FAM83E | 9 | 0.183766715688951 |  |  |  |  |  |  |
| FAM83E | 9 | 0.170819336245438 |  |  |  |  |  |  |
| FAM83E | 9 | 0.20065061684939 |  |  |  |  |  |  |
| FAM83E | 9 | 0.236433841752861 |  |  |  |  |  |  |
| FAM83E | 9 | 0.210361968992481 |  |  |  |  |  |  |
| FAM83E | 9 | 0.200114480342661 |  |  |  |  |  |  |
| FAM83E | 9 | 0.177925670737944 |  |  |  |  |  |  |
| FAM83E | 9 | 0.20231322275115 |  |  |  |  |  |  |
| FAM83E | 9 | 0.20680679201879 |  |  |  |  |  |  |
| FAM83E | 9 | 0.225692635251272 |  |  |  |  |  |  |
| FAM83E | 9 | 0.243770679446957 |  |  |  |  |  |  |
| FAM83E | 9 | 0.263607683039869 |  |  |  |  |  |  |
| FAM83E | 9 | 0.242779361848703 |  |  |  |  |  |  |
| FAM83E | 9 | 0.215899696530624 |  |  |  |  |  |  |
| FAM83E | 9 | 0.1925952629069 |  |  |  |  |  |  |
| FAM83E | 9 | 0.17480404393619 |  |  |  |  |  |  |
| FAM83E | 9 | 0.156106604614218 |  |  |  |  |  |  |
| FAM83E | 9 | 0.162756326715436 |  |  |  |  |  |  |
| FAM83E | 9 | 0.150636701593602 |  |  |  |  |  |  |
| FAM83E | 9 | 0.166543642517342 |  |  |  |  |  |  |
| FAM83E | 9 | 0.182144991159724 |  |  |  |  |  |  |
| FAM83E | 9 | 0.167924116386133 |  |  |  |  |  |  |
| FAM83E | 9 | 0.174876656035677 |  |  |  |  |  |  |
| FAM83E | 9 | 0.158559158283181 |  |  |  |  |  |  |
| FAM83E | 9 | 0.147922276324694 |  |  |  |  |  |  |
| FAM83E | 9 | 0.160698294086804 |  |  |  |  |  |  |
| FAM83E | 9 | 0.144476025352671 |  |  |  |  |  |  |
| FAM83E | 9 | 0.126005418837786 |  |  |  |  |  |  |
| FAM83E | 9 | 0.150712621597619 |  |  |  |  |  |  |
| FAM83E | 9 | 0.160606019278092 |  |  |  |  |  |  |
| FAM83E | 9 | 0.147289065575452 |  |  |  |  |  |  |
| FAM83E | 9 | 0.133226363913264 |  |  |  |  |  |  |
| FAM83E | 9 | 0.120771729170412 |  |  |  |  |  |  |
| FAM83E | 9 | 0.113581522306263 |  |  |  |  |  |  |
| FAM83E | 9 | 0.122940788497803 |  |  |  |  |  |  |
| FAM83E | 9 | 0.13530470097032 |  |  |  |  |  |  |
| FAM83E | 9 | 0.141957629411757 |  |  |  |  |  |  |
| FAM83E | 9 | 0.152710003056384 |  |  |  |  |  |  |
| FAM83E | 9 | 0.16165172494966 |  |  |  |  |  |  |
| FAM83E | 9 | 0.168815026529586 |  |  |  |  |  |  |
| FAM83E | 9 | 0.183206368411812 |  |  |  |  |  |  |
| FAM83E | 9 | 0.198923101256668 |  |  |  |  |  |  |
| FAM83E | 9 | 0.210686400389838 |  |  |  |  |  |  |
| FAM83E | 9 | 0.229340098069725 |  |  |  |  |  |  |
| FAM83E | 9 | 0.241793349992197 |  |  |  |  |  |  |
| FAM83E | 9 | 0.25923767242965 |  |  |  |  |  |  |
| FAM83E | 9 | 0.27717646849049 |  |  |  |  |  |  |
| FAM83E | 9 | 0.294129768437961 |  |  |  |  |  |  |
| FAM83E | 9 | 0.321099979720058 |  |  |  |  |  |  |
| FAM83E | 9 | 0.346563262107478 |  |  |  |  |  |  |
| FAM83E | 9 | 0.319551961943043 |  |  |  |  |  |  |
| FAM83E | 9 | 0.297628899520037 |  |  |  |  |  |  |
| FAM83E | 9 | 0.28445316044217 |  |  |  |  |  |  |
| FAM83E | 9 | 0.284653033888716 |  |  |  |  |  |  |
| FAM83E | 9 | 0.312476859513048 |  |  |  |  |  |  |
| FAM83E | 9 | 0.285329459652249 |  |  |  |  |  |  |
| FAM83E | 9 | 0.258471361943356 |  |  |  |  |  |  |
| FAM83E | 9 | 0.283647262380596 |  |  |  |  |  |  |
| FAM83E | 9 | 0.293823558935512 |  |  |  |  |  |  |
| FAM83E | 10 | 0.311880764413623 |  |  |  |  |  |  |
| FAM83E | 10 | 0.290018068899878 |  |  |  |  |  |  |
| FAM83E | 10 | 0.320233854678732 |  |  |  |  |  |  |
| FAM83E | 10 | 0.289614617145122 |  |  |  |  |  |  |
| FAM83E | 10 | 0.267175151903387 |  |  |  |  |  |  |
| FAM83E | 10 | 0.244419588678483 |  |  |  |  |  |  |
| FAM83E | 10 | 0.224928589111446 |  |  |  |  |  |  |
| FAM83E | 10 | 0.207675453893715 |  |  |  |  |  |  |
| FAM83E | 10 | 0.1914502891841 |  |  |  |  |  |  |
| FAM83E | 10 | 0.171702604791682 |  |  |  |  |  |  |
| FAM83E | 10 | 0.19121567740112 |  |  |  |  |  |  |
| FAM83E | 10 | 0.201749444015206 |  |  |  |  |  |  |
| FAM83E | 10 | 0.215232156557147 |  |  |  |  |  |  |
| FAM83E | 10 | 0.219740912515713 |  |  |  |  |  |  |
| FAM83E | 10 | 0.24135104449502 |  |  |  |  |  |  |
| FAM83E | 10 | 0.266065420632885 |  |  |  |  |  |  |
| FAM83E | 10 | 0.284360327910681 |  |  |  |  |  |  |
| FAM83E | 10 | 0.304398300404158 |  |  |  |  |  |  |
| FAM83E | 10 | 0.325706974289741 |  |  |  |  |  |  |
| FAM83E | 10 | 0.329300311466197 |  |  |  |  |  |  |
| FAM83E | 10 | 0.296802229322863 |  |  |  |  |  |  |
| FAM83E | 10 | 0.299989286897351 |  |  |  |  |  |  |
| FAM83E | 10 | 0.335379428061929 |  |  |  |  |  |  |
| FAM83E | 10 | 0.355343352499501 |  |  |  |  |  |  |
| FAM83E | 10 | 0.336474781709143 |  |  |  |  |  |  |
| FAM83E | 10 | 0.311707017681344 |  |  |  |  |  |  |
| FAM83E | 10 | 0.290051363002179 |  |  |  |  |  |  |
| FAM83E | 10 | 0.333765847044606 |  |  |  |  |  |  |
| FAM83E | 10 | 0.37928158909878 |  |  |  |  |  |  |
| FAM83E | 10 | 0.394575416793556 |  |  |  |  |  |  |
| FAM83E | 10 | 0.370295615124917 |  |  |  |  |  |  |
| FAM83E | 10 | 0.345360693024637 |  |  |  |  |  |  |
| FAM83E | 10 | 0.315217740975015 |  |  |  |  |  |  |
| FAM83E | 10 | 0.324743953164187 |  |  |  |  |  |  |
| FAM83E | 10 | 0.339468714182542 |  |  |  |  |  |  |
| FAM83E | 10 | 0.363384828481019 |  |  |  |  |  |  |
| FAM83E | 10 | 0.382923641757662 |  |  |  |  |  |  |
| FAM83E | 10 | 0.401470875024746 |  |  |  |  |  |  |
| FAM83E | 10 | 0.42300766113309 |  |  |  |  |  |  |
| FAM83E | 10 | 0.435918200584071 |  |  |  |  |  |  |
| FAM83E | 10 | 0.456355596076819 |  |  |  |  |  |  |
| FAM83E | 10 | 0.495148071814901 |  |  |  |  |  |  |
| FAM83E | 10 | 0.526782089308174 |  |  |  |  |  |  |
| FAM83E | 10 | 0.550977081965656 |  |  |  |  |  |  |
| FAM83E | 10 | 0.567287627297066 |  |  |  |  |  |  |
| FAM83E | 10 | 0.533603251677524 |  |  |  |  |  |  |
| FAM83E | 10 | 0.572880368884304 |  |  |  |  |  |  |
| FAM83E | 10 | 0.542608401227577 |  |  |  |  |  |  |
| FAM83E | 11 | 0.525498801853573 |  |  |  |  |  |  |
| FAM83E | 11 | 0.525779642345209 |  |  |  |  |  |  |
| FAM83E | 11 | 0.492771555891718 |  |  |  |  |  |  |
| FAM83E | 11 | 0.457496602385 |  |  |  |  |  |  |
| FAM83E | 11 | 0.512807701410983 |  |  |  |  |  |  |
| FAM83E | 11 | 0.553753613545787 |  |  |  |  |  |  |
| FAM83E | 11 | 0.527758327516586 |  |  |  |  |  |  |
| FAM83E | 11 | 0.508088537557775 |  |  |  |  |  |  |
| FAM83E | 11 | 0.488446210464566 |  |  |  |  |  |  |
| FAM83E | 11 | 0.478145781868208 |  |  |  |  |  |  |
| FAM83E | 11 | 0.443859051865121 |  |  |  |  |  |  |
| FAM83E | 11 | 0.467780239397728 |  |  |  |  |  |  |
| FAM83E | 11 | 0.47704273555655 |  |  |  |  |  |  |
| FAM83E | 11 | 0.436688527339172 |  |  |  |  |  |  |
| FAM83E | 11 | 0.441068350132248 |  |  |  |  |  |  |
| FAM83E | 11 | 0.445055609639826 |  |  |  |  |  |  |
| FAM83E | 11 | 0.407327107235642 |  |  |  |  |  |  |
| FAM83E | 11 | 0.370343676989292 |  |  |  |  |  |  |
| FAM83E | 11 | 0.373486163521681 |  |  |  |  |  |  |
| FAM83E | 11 | 0.344252714497019 |  |  |  |  |  |  |
| FAM83E | 11 | 0.380171965673415 |  |  |  |  |  |  |
| FAM83E | 11 | 0.346740849231767 |  |  |  |  |  |  |
| FAM83E | 11 | 0.318852043506909 |  |  |  |  |  |  |
| FAM83E | 11 | 0.296744730815093 |  |  |  |  |  |  |
| FAM83E | 11 | 0.278895786892879 |  |  |  |  |  |  |
| FAM83E | 11 | 0.262351822327743 |  |  |  |  |  |  |
| FAM83E | 11 | 0.279757931299081 |  |  |  |  |  |  |
| FAM83E | 11 | 0.275415284269957 |  |  |  |  |  |  |
| FAM83E | 11 | 0.250941499113116 |  |  |  |  |  |  |
| FAM83E | 11 | 0.224399295113269 |  |  |  |  |  |  |
| FAM83E | 11 | 0.234538606896907 |  |  |  |  |  |  |
| FAM83E | 11 | 0.234305323090093 |  |  |  |  |  |  |
| FAM83E | 11 | 0.214627024896382 |  |  |  |  |  |  |
| FAM83E | 11 | 0.196651041098601 |  |  |  |  |  |  |
| FAM83E | 11 | 0.181553292048445 |  |  |  |  |  |  |
| FAM83E | 11 | 0.162510588819023 |  |  |  |  |  |  |
| FAM83E | 11 | 0.176696969243163 |  |  |  |  |  |  |
| FAM83E | 11 | 0.195876283739331 |  |  |  |  |  |  |
| FAM83E | 11 | 0.190197261596333 |  |  |  |  |  |  |
| FAM83E | 11 | 0.206026416169202 |  |  |  |  |  |  |
| FAM83E | 11 | 0.221835298643302 |  |  |  |  |  |  |
| FAM83E | 11 | 0.23826643065784 |  |  |  |  |  |  |
| FAM83E | 11 | 0.254566069114817 |  |  |  |  |  |  |
| FAM83E | 11 | 0.273437856126086 |  |  |  |  |  |  |
| FAM83E | 11 | 0.303768575260616 |  |  |  |  |  |  |
| FAM83E | 11 | 0.330278951078885 |  |  |  |  |  |  |
| FAM83E | 11 | 0.375483534744394 |  |  |  |  |  |  |
| FAM83E | 11 | 0.346026362979731 |  |  |  |  |  |  |
| FAM83E | 11 | 0.316635169575386 |  |  |  |  |  |  |
| FAM83E | 11 | 0.292260918584792 |  |  |  |  |  |  |
| FAM83E | 11 | 0.269257859965841 |  |  |  |  |  |  |
| FAM83E | 11 | 0.248218394973108 |  |  |  |  |  |  |
| FAM83E | 11 | 0.256333518489334 |  |  |  |  |  |  |
| FAM83E | 11 | 0.234811839736262 |  |  |  |  |  |  |
| FAM83E | 12 | 0.214607405682388 |  |  |  |  |  |  |
| FAM83E | 12 | 0.214672682272677 |  |  |  |  |  |  |
| FAM83E | 12 | 0.235612697307319 |  |  |  |  |  |  |
| FAM83E | 12 | 0.212365973615145 |  |  |  |  |  |  |
| FAM83E | 12 | 0.237811233677224 |  |  |  |  |  |  |
| FAM83E | 12 | 0.23111643670589 |  |  |  |  |  |  |
| FAM83E | 12 | 0.25320337361339 |  |  |  |  |  |  |
| FAM83E | 12 | 0.229470422953717 |  |  |  |  |  |  |
| FAM83E | 12 | 0.244077831332393 |  |  |  |  |  |  |
| FAM83E | 12 | 0.275905137170414 |  |  |  |  |  |  |
| FAM83E | 12 | 0.253177162028241 |  |  |  |  |  |  |
| FAM83E | 12 | 0.286361599209038 |  |  |  |  |  |  |
| FAM83E | 12 | 0.328156970703426 |  |  |  |  |  |  |
| FAM83E | 12 | 0.361310041209795 |  |  |  |  |  |  |
| FAM83E | 12 | 0.341468178970857 |  |  |  |  |  |  |
| FAM83E | 12 | 0.330022568372059 |  |  |  |  |  |  |
| FAM83E | 12 | 0.351120365541909 |  |  |  |  |  |  |
| FAM83E | 12 | 0.324962351722305 |  |  |  |  |  |  |
| FAM83E | 12 | 0.348370207787888 |  |  |  |  |  |  |
| FAM83E | 12 | 0.318445361656145 |  |  |  |  |  |  |
| FAM83E | 12 | 0.322323967763432 |  |  |  |  |  |  |
| FAM83E | 12 | 0.323603512937706 |  |  |  |  |  |  |
| FAM83E | 12 | 0.327038112994586 |  |  |  |  |  |  |
| FAM83E | 12 | 0.296262847473853 |  |  |  |  |  |  |
| FAM83E | 12 | 0.273121136323404 |  |  |  |  |  |  |
| FAM83E | 12 | 0.263127924852718 |  |  |  |  |  |  |
| FAM83E | 12 | 0.243122687249174 |  |  |  |  |  |  |
| FAM83E | 12 | 0.217665259447151 |  |  |  |  |  |  |
| FAM83E | 12 | 0.206901089195155 |  |  |  |  |  |  |
| FAM83E | 12 | 0.223075321651847 |  |  |  |  |  |  |
| FAM83E | 12 | 0.231904363550931 |  |  |  |  |  |  |
| FAM83E | 12 | 0.21716621576524 |  |  |  |  |  |  |
| FAM83E | 12 | 0.258336073463238 |  |  |  |  |  |  |
| FAM83E | 12 | 0.233707707914863 |  |  |  |  |  |  |
| FAM83E | 12 | 0.220725856342084 |  |  |  |  |  |  |
| FAM83E | 12 | 0.214754723224279 |  |  |  |  |  |  |
| FAM83E | 12 | 0.233650742385307 |  |  |  |  |  |  |
| FAM83E | 12 | 0.265403520369192 |  |  |  |  |  |  |
| FAM83E | 12 | 0.282481998552762 |  |  |  |  |  |  |
| FAM83E | 12 | 0.25947053913826 |  |  |  |  |  |  |
| FAM83E | 12 | 0.24395962063981 |  |  |  |  |  |  |
| FAM83E | 12 | 0.25174226038996 |  |  |  |  |  |  |
| FAM83E | 12 | 0.267133544019567 |  |  |  |  |  |  |
| FAM83E | 12 | 0.246305672503138 |  |  |  |  |  |  |
| FAM83E | 12 | 0.225164955461612 |  |  |  |  |  |  |
| FAM83E | 12 | 0.219306790856865 |  |  |  |  |  |  |
| FAM83E | 12 | 0.197071245488468 |  |  |  |  |  |  |
| FAM83E | 12 | 0.215553716516069 |  |  |  |  |  |  |
| FAM83E | 12 | 0.230314292647807 |  |  |  |  |  |  |
| FAM83E | 12 | 0.234843844139036 |  |  |  |  |  |  |
| FAM83E | 12 | 0.22977632681631 |  |  |  |  |  |  |
| FAM83E | 12 | 0.214770769685519 |  |  |  |  |  |  |
| FAM83E | 12 | 0.19762615876309 |  |  |  |  |  |  |
| FAM83E | 12 | 0.229736529902693 |  |  |  |  |  |  |
| FAM83E | 12 | 0.24813821519628 |  |  |  |  |  |  |
| FAM83E | 12 | 0.265790000717509 |  |  |  |  |  |  |
| FAM83E | 13 | 0.286825929644956 |  |  |  |  |  |  |
| FAM83E | 13 | 0.315028927903993 |  |  |  |  |  |  |
| FAM83E | 13 | 0.29070412207757 |  |  |  |  |  |  |
| FAM83E | 13 | 0.332233642826436 |  |  |  |  |  |  |
| FAM83E | 13 | 0.324465020630243 |  |  |  |  |  |  |
| FAM83E | 13 | 0.310403938955416 |  |  |  |  |  |  |
| FAM83E | 13 | 0.307034317706165 |  |  |  |  |  |  |
| FAM83E | 13 | 0.280443341233607 |  |  |  |  |  |  |
| FAM83E | 13 | 0.313782168382547 |  |  |  |  |  |  |
| FAM83E | 13 | 0.310181665247808 |  |  |  |  |  |  |
| FAM83E | 13 | 0.336173968848905 |  |  |  |  |  |  |
| FAM83E | 13 | 0.35761094508315 |  |  |  |  |  |  |
| FAM83E | 13 | 0.3793187297135 |  |  |  |  |  |  |
| FAM83E | 13 | 0.362147012007148 |  |  |  |  |  |  |
| FAM83E | 13 | 0.337983417617429 |  |  |  |  |  |  |
| FAM83E | 13 | 0.371956481451084 |  |  |  |  |  |  |
| FAM83E | 13 | 0.343106722489784 |  |  |  |  |  |  |
| FAM83E | 13 | 0.328796302798794 |  |  |  |  |  |  |
| FAM83E | 13 | 0.309181485723847 |  |  |  |  |  |  |
| FAM83E | 13 | 0.328816667667291 |  |  |  |  |  |  |
| FAM83E | 13 | 0.341218501960017 |  |  |  |  |  |  |
| FAM83E | 13 | 0.314785921487054 |  |  |  |  |  |  |
| FAM83E | 13 | 0.35082831789114 |  |  |  |  |  |  |
| FAM83E | 13 | 0.336142000174259 |  |  |  |  |  |  |
| FAM83E | 13 | 0.31564661298728 |  |  |  |  |  |  |
| FAM83E | 13 | 0.333763262016257 |  |  |  |  |  |  |
| FAM83E | 13 | 0.361064672173185 |  |  |  |  |  |  |
| FAM83E | 13 | 0.345339070102913 |  |  |  |  |  |  |
| FAM83E | 13 | 0.3680574199314 |  |  |  |  |  |  |
| FAM83E | 13 | 0.400893717694117 |  |  |  |  |  |  |
| FAM83E | 13 | 0.427656283899656 |  |  |  |  |  |  |
| FAM83E | 13 | 0.400217093423328 |  |  |  |  |  |  |
| FAM83E | 13 | 0.366343971095383 |  |  |  |  |  |  |
| FAM83E | 13 | 0.343224696659725 |  |  |  |  |  |  |
| FAM83E | 13 | 0.32195693634581 |  |  |  |  |  |  |
| FAM83E | 13 | 0.331964233945404 |  |  |  |  |  |  |
| FAM83E | 13 | 0.35078794840533 |  |  |  |  |  |  |
| FAM83E | 13 | 0.321447992397093 |  |  |  |  |  |  |
| FAM83E | 13 | 0.340391562458178 |  |  |  |  |  |  |
| FAM83E | 13 | 0.384925822786981 |  |  |  |  |  |  |
| FAM83E | 13 | 0.359242409327417 |  |  |  |  |  |  |
| FAM83E | 13 | 0.376744242938545 |  |  |  |  |  |  |
| FAM83E | 14 | 0.384427566394145 |  |  |  |  |  |  |
| FAM83E | 14 | 0.414422137287992 |  |  |  |  |  |  |
| FAM83E | 14 | 0.382483548016697 |  |  |  |  |  |  |
| FAM83E | 14 | 0.35489228440953 |  |  |  |  |  |  |
| FAM83E | 14 | 0.336746734673612 |  |  |  |  |  |  |
| FAM83E | 14 | 0.322556559914091 |  |  |  |  |  |  |
| FAM83E | 14 | 0.29477081855163 |  |  |  |  |  |  |
| FAM83E | 14 | 0.318342528129506 |  |  |  |  |  |  |
| FAM83E | 14 | 0.354097514741784 |  |  |  |  |  |  |
| FAM83E | 14 | 0.322200143614889 |  |  |  |  |  |  |
| FAM83E | 14 | 0.350843730949909 |  |  |  |  |  |  |
| FAM83E | 14 | 0.329055906979283 |  |  |  |  |  |  |
| FAM83E | 14 | 0.355948821479906 |  |  |  |  |  |  |
| FAM83E | 14 | 0.375308982514683 |  |  |  |  |  |  |
| FAM83E | 14 | 0.379567436597653 |  |  |  |  |  |  |
| FAM83E | 14 | 0.351217620971378 |  |  |  |  |  |  |
| FAM83E | 14 | 0.391216230679511 |  |  |  |  |  |  |
| FAM83E | 14 | 0.411832825337305 |  |  |  |  |  |  |
| FAM83E | 14 | 0.428068014334702 |  |  |  |  |  |  |
| FAM83E | 14 | 0.412889704344187 |  |  |  |  |  |  |
| FAM83E | 14 | 0.415443919402998 |  |  |  |  |  |  |
| FAM83E | 14 | 0.457242597850592 |  |  |  |  |  |  |
| FAM83E | 14 | 0.510616848964986 |  |  |  |  |  |  |
| FAM83E | 14 | 0.554416522534625 |  |  |  |  |  |  |
| FAM83E | 14 | 0.511734394031777 |  |  |  |  |  |  |
| FAM83E | 14 | 0.471790302403015 |  |  |  |  |  |  |
| FAM83E | 14 | 0.495935566026749 |  |  |  |  |  |  |
| FAM83E | 14 | 0.51810690418576 |  |  |  |  |  |  |
| FAM83E | 14 | 0.485549541912923 |  |  |  |  |  |  |
| FAM83E | 14 | 0.515338487148607 |  |  |  |  |  |  |
| FAM83E | 14 | 0.543636006510203 |  |  |  |  |  |  |
| FAM83E | 14 | 0.501620736301944 |  |  |  |  |  |  |
| FAM83E | 14 | 0.462448389930389 |  |  |  |  |  |  |
| FAM83E | 14 | 0.430498623988826 |  |  |  |  |  |  |
| FAM83E | 14 | 0.410629401650193 |  |  |  |  |  |  |
| FAM83E | 14 | 0.411845162921783 |  |  |  |  |  |  |
| FAM83E | 14 | 0.435285867192475 |  |  |  |  |  |  |
| FAM83E | 15 | 0.409150969686108 |  |  |  |  |  |  |
| FAM83E | 15 | 0.446916985350534 |  |  |  |  |  |  |
| FAM83E | 15 | 0.415360062565789 |  |  |  |  |  |  |
| FAM83E | 15 | 0.437452837311213 |  |  |  |  |  |  |
| FAM83E | 15 | 0.413170796471026 |  |  |  |  |  |  |
| FAM83E | 15 | 0.451643245232486 |  |  |  |  |  |  |
| FAM83E | 15 | 0.438289064736231 |  |  |  |  |  |  |
| FAM83E | 15 | 0.414051669101176 |  |  |  |  |  |  |
| FAM83E | 15 | 0.444853348799263 |  |  |  |  |  |  |
| FAM83E | 15 | 0.470470602553646 |  |  |  |  |  |  |
| FAM83E | 15 | 0.437293499151157 |  |  |  |  |  |  |
| FAM83E | 15 | 0.441549596408336 |  |  |  |  |  |  |
| FAM83E | 15 | 0.445664403007409 |  |  |  |  |  |  |
| FAM83E | 15 | 0.449242694843157 |  |  |  |  |  |  |
| FAM83E | 15 | 0.452584746009955 |  |  |  |  |  |  |
| FAM83E | 15 | 0.490321001133632 |  |  |  |  |  |  |
| FAM83E | 15 | 0.453104715198909 |  |  |  |  |  |  |
| FAM83E | 15 | 0.476630426955025 |  |  |  |  |  |  |
| FAM83E | 15 | 0.444831348461977 |  |  |  |  |  |  |
| FAM83E | 15 | 0.414182779264946 |  |  |  |  |  |  |
| FAM83E | 15 | 0.437629971099436 |  |  |  |  |  |  |
| FAM83E | 15 | 0.485298965886792 |  |  |  |  |  |  |
| FAM83E | 15 | 0.505894896212428 |  |  |  |  |  |  |
| FAM83E | 15 | 0.52416189125263 |  |  |  |  |  |  |
| FAM83E | 15 | 0.580043842963566 |  |  |  |  |  |  |
| FAM83E | 15 | 0.549524657315408 |  |  |  |  |  |  |
| FAM83E | 15 | 0.576804574232703 |  |  |  |  |  |  |
| FAM83E | 15 | 0.617314999315538 |  |  |  |  |  |  |
| FAM83E | 15 | 0.647509473785396 |  |  |  |  |  |  |
| FAM83E | 15 | 0.676293928377957 |  |  |  |  |  |  |
| FAM83E | 15 | 0.71500064794568 |  |  |  |  |  |  |
| FAM83E | 15 | 0.731844016903737 |  |  |  |  |  |  |
| FAM83E | 15 | 0.697054544862123 |  |  |  |  |  |  |
| FAM83E | 15 | 0.727116666690487 |  |  |  |  |  |  |
| FAM83E | 15 | 0.701987570022122 |  |  |  |  |  |  |
| FAM83E | 15 | 0.670770474137771 |  |  |  |  |  |  |
| FAM83E | 15 | 0.697618016201039 |  |  |  |  |  |  |
| FAM83E | 15 | 0.679706066355064 |  |  |  |  |  |  |
| FAM83E | 15 | 0.720665957866158 |  |  |  |  |  |  |
| FAM83E | 15 | 0.682535203952131 |  |  |  |  |  |  |
| FAM83E | 15 | 0.64868700128182 |  |  |  |  |  |  |
| FAM83E | 16 | 0.677862584375836 |  |  |  |  |  |  |
| FAM83E | 16 | 0.680416235049148 |  |  |  |  |  |  |
| FAM83E | 16 | 0.671200669544743 |  |  |  |  |  |  |
| FAM83E | 16 | 0.745768934604947 |  |  |  |  |  |  |
| FAM83E | 16 | 0.742510139289071 |  |  |  |  |  |  |
| FAM83E | 16 | 0.769367882905268 |  |  |  |  |  |  |
| FAM83E | 16 | 0.72275478089272 |  |  |  |  |  |  |
| FAM83E | 16 | 0.743377637474541 |  |  |  |  |  |  |
| FAM83E | 16 | 0.80632768945055 |  |  |  |  |  |  |
| FAM83E | 16 | 0.787903037118332 |  |  |  |  |  |  |
| FAM83E | 16 | 0.739001605420199 |  |  |  |  |  |  |
| FAM83E | 16 | 0.691690083036358 |  |  |  |  |  |  |
| FAM83E | 16 | 0.731423988073059 |  |  |  |  |  |  |
| FAM83E | 16 | 0.684015422366057 |  |  |  |  |  |  |
| FAM83E | 16 | 0.649199512928177 |  |  |  |  |  |  |
| FAM83E | 16 | 0.621183587928945 |  |  |  |  |  |  |
| FAM83E | 16 | 0.649822023249275 |  |  |  |  |  |  |
| FAM83E | 16 | 0.609239825376368 |  |  |  |  |  |  |
| FAM83E | 16 | 0.668748605151983 |  |  |  |  |  |  |
| FAM83E | 16 | 0.698226156340749 |  |  |  |  |  |  |
| FAM83E | 16 | 0.728695290729125 |  |  |  |  |  |  |
| FAM83E | 16 | 0.682832432287525 |  |  |  |  |  |  |
| FAM83E | 16 | 0.661485371517938 |  |  |  |  |  |  |
| FAM83E | 16 | 0.691052979707988 |  |  |  |  |  |  |
| FAM83E | 16 | 0.645467585296448 |  |  |  |  |  |  |
| FAM83E | 16 | 0.611266818950952 |  |  |  |  |  |  |
| FAM83E | 16 | 0.629395675806571 |  |  |  |  |  |  |
| FAM83E | 16 | 0.642879069000043 |  |  |  |  |  |  |
| FAM83E | 16 | 0.654443641584178 |  |  |  |  |  |  |
| FAM83E | 17 | 0.668884733621621 |  |  |  |  |  |  |
| FAM83E | 17 | 0.631307100736127 |  |  |  |  |  |  |
| FAM83E | 17 | 0.588790761296558 |  |  |  |  |  |  |
| FAM83E | 17 | 0.619837339457893 |  |  |  |  |  |  |
| FAM83E | 17 | 0.664229682496736 |  |  |  |  |  |  |
| FAM83E | 17 | 0.624606764224783 |  |  |  |  |  |  |
| FAM83E | 17 | 0.5984938063292 |  |  |  |  |  |  |
| FAM83E | 17 | 0.614741110293639 |  |  |  |  |  |  |
| FAM83E | 17 | 0.629052594517676 |  |  |  |  |  |  |
| FAM83E | 17 | 0.600985240489841 |  |  |  |  |  |  |
| FAM83E | 17 | 0.60159867754378 |  |  |  |  |  |  |
| FAM83E | 17 | 0.641268472919871 |  |  |  |  |  |  |
| FAM83E | 17 | 0.684278421302687 |  |  |  |  |  |  |
| FAM83E | 17 | 0.700796939450595 |  |  |  |  |  |  |
| FAM83E | 17 | 0.730796557670984 |  |  |  |  |  |  |
| FAM83E | 17 | 0.767098579037773 |  |  |  |  |  |  |
| FAM83E | 17 | 0.80768840518546 |  |  |  |  |  |  |
| FAM83E | 17 | 0.763056113744647 |  |  |  |  |  |  |
| FAM83E | 17 | 0.798644847022155 |  |  |  |  |  |  |
| FAM83E | 17 | 0.864625627421487 |  |  |  |  |  |  |
| FAM83E | 17 | 0.817884238551994 |  |  |  |  |  |  |
| FAM83E | 17 | 0.798469532496273 |  |  |  |  |  |  |
| FAM83E | 17 | 0.753797496575918 |  |  |  |  |  |  |
| FAM83E | 17 | 0.707079655674878 |  |  |  |  |  |  |
| FAM83E | 17 | 0.694385542816137 |  |  |  |  |  |  |
| FAM83E | 18 | 0.659172489559022 |  |  |  |  |  |  |
| FAM83E | 18 | 0.681303754464698 |  |  |  |  |  |  |
| FAM83E | 18 | 0.663737085186241 |  |  |  |  |  |  |
| FAM83E | 18 | 0.657262753222921 |  |  |  |  |  |  |
| FAM83E | 18 | 0.719491171772867 |  |  |  |  |  |  |
| FAM83E | 18 | 0.713006030597072 |  |  |  |  |  |  |
| FAM83E | 18 | 0.697971091854633 |  |  |  |  |  |  |
| FAM83E | 18 | 0.653376928240346 |  |  |  |  |  |  |
| FAM83E | 18 | 0.675764935392005 |  |  |  |  |  |  |
| FAM83E | 18 | 0.635834328946534 |  |  |  |  |  |  |
| FAM83E | 18 | 0.651386854253734 |  |  |  |  |  |  |
| FAM83E | 18 | 0.636737641253178 |  |  |  |  |  |  |
| FAM83E | 18 | 0.692363277023421 |  |  |  |  |  |  |
| FAM83E | 18 | 0.712444992077291 |  |  |  |  |  |  |
| FAM83E | 18 | 0.731089199721089 |  |  |  |  |  |  |
| FAM83E | 18 | 0.716163902124282 |  |  |  |  |  |  |
| FAM83E | 18 | 0.721117474945222 |  |  |  |  |  |  |
| FAM83E | 18 | 0.725822311330884 |  |  |  |  |  |  |
| FAM83E | 18 | 0.679515858207027 |  |  |  |  |  |  |
| FAM83E | 18 | 0.633943385987071 |  |  |  |  |  |  |
| FAM83E | 18 | 0.590210019184588 |  |  |  |  |  |  |
| FAM83E | 18 | 0.592954246146276 |  |  |  |  |  |  |
| FAM83E | 18 | 0.555166730951512 |  |  |  |  |  |  |
| FAM83E | 18 | 0.584197613259083 |  |  |  |  |  |  |
| FAM83E | 18 | 0.6147046981198 |  |  |  |  |  |  |
| FAM83E | 18 | 0.578928568688355 |  |  |  |  |  |  |
| FAM83E | 18 | 0.541129338268213 |  |  |  |  |  |  |
| FAM83E | 18 | 0.558650000765006 |  |  |  |  |  |  |
| FAM83E | 18 | 0.527850282525163 |  |  |  |  |  |  |
| FAM83E | 18 | 0.525442514102327 |  |  |  |  |  |  |
| FAM83E | 18 | 0.505195392856748 |  |  |  |  |  |  |
| FAM83E | 18 | 0.528208150723887 |  |  |  |  |  |  |
| FAM83E | 18 | 0.510291498693146 |  |  |  |  |  |  |
| FAM83E | 19 | 0.529226088802062 |  |  |  |  |  |  |
| FAM83E | 19 | 0.529335938043525 |  |  |  |  |  |  |
| FAM83E | 19 | 0.536299135174152 |  |  |  |  |  |  |
| FAM83E | 19 | 0.579398099531352 |  |  |  |  |  |  |
| FAM83E | 19 | 0.619710402367028 |  |  |  |  |  |  |
| FAM83E | 19 | 0.637733827311558 |  |  |  |  |  |  |
| FAM83E | 19 | 0.683628864855187 |  |  |  |  |  |  |
| FAM83E | 19 | 0.746687404346567 |  |  |  |  |  |  |
| FAM83E | 19 | 0.755291848711627 |  |  |  |  |  |  |
| FAM83E | 19 | 0.793198635429758 |  |  |  |  |  |  |
| FAM83E | 19 | 0.74864683949048 |  |  |  |  |  |  |
| FAM83E | 19 | 0.742458076304747 |  |  |  |  |  |  |
| FAM83E | 19 | 0.698755053023077 |  |  |  |  |  |  |
| FAM83E | 19 | 0.676239556935036 |  |  |  |  |  |  |
| FAM83E | 19 | 0.706927827445539 |  |  |  |  |  |  |
| FAM83E | 19 | 0.747704572196951 |  |  |  |  |  |  |
| FAM83E | 19 | 0.725359805169656 |  |  |  |  |  |  |
| FAM83E | 19 | 0.729475318164752 |  |  |  |  |  |  |
| FAM83E | 19 | 0.694452489298662 |  |  |  |  |  |  |
| FAM83E | 19 | 0.657265595275536 |  |  |  |  |  |  |
| FAM83E | 19 | 0.620478460074441 |  |  |  |  |  |  |
| FAM83E | 19 | 0.583996795451816 |  |  |  |  |  |  |
| FAM83E | 19 | 0.544028600416226 |  |  |  |  |  |  |
| FAM83E | 19 | 0.57520766148985 |  |  |  |  |  |  |
| FAM83E | 19 | 0.544923093542087 |  |  |  |  |  |  |
| FAM83E | 19 | 0.513272736346328 |  |  |  |  |  |  |
| FAM83E | 19 | 0.551727446956616 |  |  |  |  |  |  |
| FAM83E | 19 | 0.582066224667291 |  |  |  |  |  |  |
| FAM83E | 19 | 0.635127626687173 |  |  |  |  |  |  |
| FAM83E | 19 | 0.668314619883026 |  |  |  |  |  |  |
| FAM83E | 19 | 0.683688676133019 |  |  |  |  |  |  |
| FAM83E | 20 | 0.722811810106478 |  |  |  |  |  |  |
| FAM83E | 20 | 0.689710896029401 |  |  |  |  |  |  |
| FAM83E | 20 | 0.662084810416131 |  |  |  |  |  |  |
| FAM83E | 20 | 0.625752723590007 |  |  |  |  |  |  |
| FAM83E | 20 | 0.609166967340286 |  |  |  |  |  |  |
| FAM83E | 20 | 0.578663569378582 |  |  |  |  |  |  |
| FAM83E | 20 | 0.550635534955587 |  |  |  |  |  |  |
| FAM83E | 20 | 0.60525706777346 |  |  |  |  |  |  |
| FAM83E | 20 | 0.652076868296962 |  |  |  |  |  |  |
| FAM83E | 20 | 0.733679708905597 |  |  |  |  |  |  |
| FAM83E | 20 | 0.764151968909143 |  |  |  |  |  |  |
| FAM83E | 20 | 0.768342912400088 |  |  |  |  |  |  |
| FAM83E | 20 | 0.734716512576856 |  |  |  |  |  |  |
| FAM83E | 20 | 0.739487111615589 |  |  |  |  |  |  |
| FAM83E | 20 | 0.758771971452705 |  |  |  |  |  |  |
| FAM83E | 20 | 0.731866415456587 |  |  |  |  |  |  |
| FAM83E | 20 | 0.693140121543014 |  |  |  |  |  |  |
| FAM83E | 20 | 0.736945986801631 |  |  |  |  |  |  |
| FAM83E | 20 | 0.775330334809047 |  |  |  |  |  |  |
| FAM83E | 20 | 0.728299335693584 |  |  |  |  |  |  |
| FAM83E | 20 | 0.693407105903863 |  |  |  |  |  |  |
| FAM83E | 20 | 0.666270312936638 |  |  |  |  |  |  |
| FAM83E | 20 | 0.623748093728309 |  |  |  |  |  |  |
| FAM83E | 20 | 0.60261672769958 |  |  |  |  |  |  |
| FAM83E | 20 | 0.644783533352378 |  |  |  |  |  |  |
| FAM83E | 20 | 0.681820387094048 |  |  |  |  |  |  |
| FAM83E | 21 | 0.649468166157413 |  |  |  |  |  |  |
| FAM83E | 21 | 0.620074937762542 |  |  |  |  |  |  |
| FAM83E | 21 | 0.584097828148703 |  |  |  |  |  |  |
| FAM83E | 21 | 0.541704727314979 |  |  |  |  |  |  |
| FAM83E | 21 | 0.499933819143748 |  |  |  |  |  |  |
| FAM83E | 21 | 0.554210903163463 |  |  |  |  |  |  |
| FAM83E | 21 | 0.537229852033726 |  |  |  |  |  |  |
| FAM83E | 21 | 0.499953328808828 |  |  |  |  |  |  |
| FAM83E | 21 | 0.473180317989904 |  |  |  |  |  |  |
| FAM83E | 21 | 0.485616541553874 |  |  |  |  |  |  |
| FAM83E | 21 | 0.45652913410978 |  |  |  |  |  |  |
| FAM83E | 21 | 0.444855363280561 |  |  |  |  |  |  |
| FAM83E | 21 | 0.412359184420691 |  |  |  |  |  |  |
| FAM83E | 21 | 0.436217001343865 |  |  |  |  |  |  |
| FAM83E | 21 | 0.401381852136842 |  |  |  |  |  |  |
| FAM83E | 21 | 0.369758970069227 |  |  |  |  |  |  |
| FAM83E | 21 | 0.352726968640298 |  |  |  |  |  |  |
| FAM83E | 21 | 0.377529871322989 |  |  |  |  |  |  |
| FAM83E | 21 | 0.362675736945225 |  |  |  |  |  |  |
| FAM83E | 21 | 0.384601478038268 |  |  |  |  |  |  |
| FAM83E | 21 | 0.387902550407872 |  |  |  |  |  |  |
| FAM83E | 21 | 0.391389947246499 |  |  |  |  |  |  |
| FAM83E | 21 | 0.357143882822707 |  |  |  |  |  |  |
| FAM83E | 21 | 0.35411711862623 |  |  |  |  |  |  |
| FAM83E | 21 | 0.328806120172249 |  |  |  |  |  |  |
| FAM83E | 21 | 0.346860450583618 |  |  |  |  |  |  |
| FAM83E | 21 | 0.338167382790217 |  |  |  |  |  |  |
| FAM83E | 21 | 0.356426433096245 |  |  |  |  |  |  |
| FAM83E | 21 | 0.38429436278244 |  |  |  |  |  |  |
| FAM83E | 21 | 0.365272927152562 |  |  |  |  |  |  |
| FAM83E | 21 | 0.334626372269864 |  |  |  |  |  |  |
| FAM83E | 21 | 0.333372823833178 |  |  |  |  |  |  |
| FAM83E | 21 | 0.380172489913389 |  |  |  |  |  |  |
| FAM83E | 22 | 0.424175120058055 |  |  |  |  |  |  |
| FAM83E | 22 | 0.411406756639341 |  |  |  |  |  |  |
| FAM83E | 22 | 0.382145264142902 |  |  |  |  |  |  |
| FAM83E | 22 | 0.360006841269092 |  |  |  |  |  |  |
| FAM83E | 22 | 0.378324560544924 |  |  |  |  |  |  |
| FAM83E | 22 | 0.396322277716208 |  |  |  |  |  |  |
| FAM83E | 22 | 0.37055044617041 |  |  |  |  |  |  |
| FAM83E | 22 | 0.393597590954442 |  |  |  |  |  |  |
| FAM83E | 22 | 0.421727721816493 |  |  |  |  |  |  |
| FAM83E | 22 | 0.395598699401143 |  |  |  |  |  |  |
| FAM83E | 22 | 0.360980198924452 |  |  |  |  |  |  |
| FAM83E | 22 | 0.364241273536481 |  |  |  |  |  |  |
| FAM83E | 22 | 0.331878947521216 |  |  |  |  |  |  |
| FAM83E | 22 | 0.301386526381136 |  |  |  |  |  |  |
| FAM83E | 22 | 0.313119006792371 |  |  |  |  |  |  |
| FAM83E | 22 | 0.331789439626035 |  |  |  |  |  |  |
| FAM83E | 22 | 0.355634316534401 |  |  |  |  |  |  |
| FAM83E | 22 | 0.377931902400596 |  |  |  |  |  |  |
| FAM83E | 22 | 0.404116522205993 |  |  |  |  |  |  |
| FAM83E | 22 | 0.395661202000022 |  |  |  |  |  |  |
| FAM83E | 22 | 0.402737438909354 |  |  |  |  |  |  |
| FAM83E | 22 | 0.424277617874125 |  |  |  |  |  |  |
| FAM83E | 22 | 0.439503313982137 |  |  |  |  |  |  |
| FAM83E | 22 | 0.409199397741726 |  |  |  |  |  |  |
| FAM83E | 22 | 0.425410918610169 |  |  |  |  |  |  |
| FAM83E | 23 | 0.443000434843495 |  |  |  |  |  |  |
| FAM83E | 23 | 0.43427493529561 |  |  |  |  |  |  |
| FAM83E | 23 | 0.401227300447155 |  |  |  |  |  |  |
| FAM83E | 23 | 0.383470115858283 |  |  |  |  |  |  |
| FAM83E | 23 | 0.406108761975397 |  |  |  |  |  |  |
| FAM83E | 23 | 0.389636099255336 |  |  |  |  |  |  |
| FAM83E | 23 | 0.424210519348958 |  |  |  |  |  |  |
| FAM83E | 23 | 0.447879566051038 |  |  |  |  |  |  |
| FAM83E | 23 | 0.469885482344355 |  |  |  |  |  |  |
| FAM83E | 23 | 0.490545465620714 |  |  |  |  |  |  |
| FAM83E | 23 | 0.458072509344489 |  |  |  |  |  |  |
| FAM83E | 23 | 0.471660472186196 |  |  |  |  |  |  |
| FAM83E | 23 | 0.447091822640133 |  |  |  |  |  |  |
| FAM83E | 23 | 0.427338045902459 |  |  |  |  |  |  |
| FAM83E | 23 | 0.390734576245748 |  |  |  |  |  |  |
| FAM83E | 23 | 0.392863388504235 |  |  |  |  |  |  |
| FAM83E | 23 | 0.373156574288926 |  |  |  |  |  |  |
| FAM83E | 23 | 0.348476586201217 |  |  |  |  |  |  |
| FAM83E | 23 | 0.319651675981933 |  |  |  |  |  |  |
| FAM83E | 23 | 0.303197730578485 |  |  |  |  |  |  |
| FAM83E | 23 | 0.31457855509579 |  |  |  |  |  |  |
| FAM83E | 23 | 0.288673664686025 |  |  |  |  |  |  |
| FAM83E | 23 | 0.260171855409619 |  |  |  |  |  |  |
| FAM83E | 23 | 0.269651760469767 |  |  |  |  |  |  |
| FAM83E | 23 | 0.274085532208265 |  |  |  |  |  |  |
| FAM83E | 23 | 0.250843737171503 |  |  |  |  |  |  |
| FAM83E | 24 | 0.234722396570481 |  |  |  |  |  |  |
| FAM83E | 24 | 0.24132161004811 |  |  |  |  |  |  |
| FAM83E | 24 | 0.26720191413964 |  |  |  |  |  |  |
| FAM83E | 24 | 0.28887654386386 |  |  |  |  |  |  |
| FAM83E | 24 | 0.307453542543616 |  |  |  |  |  |  |
| FAM83E | 24 | 0.278151567453178 |  |  |  |  |  |  |
| FAM83E | 24 | 0.31233262730258 |  |  |  |  |  |  |
| FAM83E | 24 | 0.286897472052062 |  |  |  |  |  |  |
| FAM83E | 24 | 0.293153416896648 |  |  |  |  |  |  |
| FAM83E | 24 | 0.26468958513728 |  |  |  |  |  |  |
| FAM83E | 24 | 0.268033210497051 |  |  |  |  |  |  |
| FAM83E | 24 | 0.251021500727372 |  |  |  |  |  |  |
| FAM83E | 24 | 0.268009878521499 |  |  |  |  |  |  |
| FAM83E | 24 | 0.250073703431175 |  |  |  |  |  |  |
| FAM83E | 24 | 0.236791008953729 |  |  |  |  |  |  |
| FAM83E | 24 | 0.258131136304815 |  |  |  |  |  |  |
| FAM83E | 24 | 0.240737484735006 |  |  |  |  |  |  |
| FAM83E | 24 | 0.255481872484842 |  |  |  |  |  |  |
| FAM83E | 24 | 0.244556753428982 |  |  |  |  |  |  |
| FAM83E | 24 | 0.224193988509909 |  |  |  |  |  |  |
| FAM83E | 24 | 0.212271135063663 |  |  |  |  |  |  |
| FAM83E | 24 | 0.197506246252483 |  |  |  |  |  |  |
| FAM83E | 24 | 0.183576341339796 |  |  |  |  |  |  |
| FAM83E | 24 | 0.167321403816861 |  |  |  |  |  |  |
| FAM83E | 24 | 0.181628605565833 |  |  |  |  |  |  |
| FAM83E | 24 | 0.165023982621738 |  |  |  |  |  |  |
| FAM83E | 25 | 0.178259354003986 |  |  |  |  |  |  |
| FAM83E | 25 | 0.185337983416776 |  |  |  |  |  |  |
| FAM83E | 25 | 0.208341551667068 |  |  |  |  |  |  |
| FAM83E | 25 | 0.186642471971058 |  |  |  |  |  |  |
| FAM83E | 25 | 0.194696478191614 |  |  |  |  |  |  |
| FAM83E | 25 | 0.184112475804542 |  |  |  |  |  |  |
| FAM83E | 25 | 0.20001731136374 |  |  |  |  |  |  |
| FAM83E | 25 | 0.178057058781579 |  |  |  |  |  |  |
| FAM83E | 25 | 0.180319030089318 |  |  |  |  |  |  |
| FAM83E | 25 | 0.192325356378168 |  |  |  |  |  |  |
| FAM83E | 25 | 0.178125133628447 |  |  |  |  |  |  |
| FAM83E | 25 | 0.194456079705198 |  |  |  |  |  |  |
| FAM83E | 25 | 0.208745350891982 |  |  |  |  |  |  |
| FAM83E | 25 | 0.203529377317863 |  |  |  |  |  |  |
| FAM83E | 25 | 0.220815164561671 |  |  |  |  |  |  |
| FAM83E | 25 | 0.205140018988351 |  |  |  |  |  |  |
| FAM83E | 25 | 0.215902152020179 |  |  |  |  |  |  |
| FAM83E | 25 | 0.202536722619593 |  |  |  |  |  |  |
| FAM83E | 25 | 0.191991009057088 |  |  |  |  |  |  |
| FAM83E | 25 | 0.175075041151852 |  |  |  |  |  |  |
| FAM83E | 25 | 0.185451995050701 |  |  |  |  |  |  |
| FAM83E | 26 | 0.198655545028614 |  |  |  |  |  |  |
| FAM83E | 26 | 0.18804732339287 |  |  |  |  |  |  |
| FAM83E | 26 | 0.20866457137261 |  |  |  |  |  |  |
| FAM83E | 26 | 0.222254327065538 |  |  |  |  |  |  |
| FAM83E | 26 | 0.203459694323023 |  |  |  |  |  |  |
| FAM83E | 26 | 0.200743905600823 |  |  |  |  |  |  |
| FAM83E | 26 | 0.1912552697653 |  |  |  |  |  |  |
| FAM83E | 26 | 0.170888712494997 |  |  |  |  |  |  |
| FAM83E | 26 | 0.162843620539177 |  |  |  |  |  |  |
| FAM83E | 26 | 0.163694753069626 |  |  |  |  |  |  |
| FAM83E | 26 | 0.145091579994652 |  |  |  |  |  |  |
| FAM83E | 26 | 0.146753054688924 |  |  |  |  |  |  |
| FAM83E | 26 | 0.130709220248419 |  |  |  |  |  |  |
| FAM83E | 26 | 0.140013556758305 |  |  |  |  |  |  |
| FAM83E | 26 | 0.124795382404539 |  |  |  |  |  |  |
| FAM83E | 26 | 0.135175434398528 |  |  |  |  |  |  |
| FAM83E | 26 | 0.14690711673511 |  |  |  |  |  |  |
| FAM83E | 26 | 0.139923918144691 |  |  |  |  |  |  |
| FAM83E | 26 | 0.128400773898006 |  |  |  |  |  |  |
| FAM83E | 27 | 0.121814721894566 |  |  |  |  |  |  |
| FAM83E | 27 | 0.133911939114223 |  |  |  |  |  |  |
| FAM83E | 27 | 0.148817013084514 |  |  |  |  |  |  |
| FAM83E | 27 | 0.160899422950584 |  |  |  |  |  |  |
| FAM83E | 27 | 0.181176224134875 |  |  |  |  |  |  |
| FAM83E | 27 | 0.19536601092461 |  |  |  |  |  |  |
| FAM83E | 27 | 0.190620620268529 |  |  |  |  |  |  |
| FAM83E | 27 | 0.178544984474478 |  |  |  |  |  |  |
| FAM83E | 27 | 0.20490160558683 |  |  |  |  |  |  |
| FAM83E | 27 | 0.222203723466725 |  |  |  |  |  |  |
| FAM83E | 27 | 0.224260759300725 |  |  |  |  |  |  |
| FAM83E | 27 | 0.225858106241206 |  |  |  |  |  |  |
| FAM83E | 27 | 0.251986951760489 |  |  |  |  |  |  |
| FAM83E | 27 | 0.23564861726737 |  |  |  |  |  |  |
| FAM83E | 27 | 0.252109978286304 |  |  |  |  |  |  |
| FAM83E | 27 | 0.240065144374569 |  |  |  |  |  |  |
| FAM83E | 27 | 0.234983415094362 |  |  |  |  |  |  |
| FAM83E | 28 | 0.221782378030535 |  |  |  |  |  |  |
| FAM83E | 28 | 0.248574065072858 |  |  |  |  |  |  |
| FAM83E | 28 | 0.224219838474395 |  |  |  |  |  |  |
| FAM83E | 28 | 0.216371651931148 |  |  |  |  |  |  |
| FAM83E | 28 | 0.2180011596074 |  |  |  |  |  |  |
| FAM83E | 28 | 0.220920510858664 |  |  |  |  |  |  |
| FAM83E | 28 | 0.200639013376026 |  |  |  |  |  |  |
| FAM83E | 28 | 0.216802188862846 |  |  |  |  |  |  |
| FAM83E | 28 | 0.196356997137904 |  |  |  |  |  |  |
| FAM83E | 28 | 0.177538609499597 |  |  |  |  |  |  |
| FAM83E | 28 | 0.190801181283602 |  |  |  |  |  |  |
| FAM83E | 28 | 0.169314749902535 |  |  |  |  |  |  |
| FAM83E | 28 | 0.158262135888098 |  |  |  |  |  |  |
| FAM83E | 28 | 0.170184404958728 |  |  |  |  |  |  |
| FAM83E | 28 | 0.182654607113695 |  |  |  |  |  |  |
| FAM83E | 28 | 0.202115210435055 |  |  |  |  |  |  |
| FAM83E | 28 | 0.183455355449461 |  |  |  |  |  |  |
| FAM83E | 28 | 0.206074881144678 |  |  |  |  |  |  |
| FAM83E | 28 | 0.226922761783963 |  |  |  |  |  |  |
| FAM83E | 28 | 0.206712193437316 |  |  |  |  |  |  |
| FAM83E | 28 | 0.218068609919441 |  |  |  |  |  |  |
| FAM83E | 29 | 0.206496047353075 |  |  |  |  |  |  |
| FAM83E | 29 | 0.190726898556498 |  |  |  |  |  |  |
| FAM83E | 29 | 0.209226314050452 |  |  |  |  |  |  |
| FAM83E | 29 | 0.191055600968815 |  |  |  |  |  |  |
| FAM83E | 29 | 0.192335857539543 |  |  |  |  |  |  |
| FAM83E | 29 | 0.171721922990813 |  |  |  |  |  |  |
| FAM83E | 29 | 0.185436446623509 |  |  |  |  |  |  |
| FAM83E | 29 | 0.164619277693011 |  |  |  |  |  |  |
| FAM83E | 29 | 0.177333645411554 |  |  |  |  |  |  |
| FAM83E | 29 | 0.184334554456784 |  |  |  |  |  |  |
| FAM83E | 29 | 0.200483596710184 |  |  |  |  |  |  |
| FAM83E | 29 | 0.182857329901486 |  |  |  |  |  |  |
| FAM83E | 29 | 0.197821282624094 |  |  |  |  |  |  |
| FAM83E | 29 | 0.210976756787902 |  |  |  |  |  |  |
| FAM83E | 29 | 0.191630548491587 |  |  |  |  |  |  |
| FAM83E | 29 | 0.214611262017369 |  |  |  |  |  |  |
| FAM83E | 29 | 0.227091162775674 |  |  |  |  |  |  |
| FAM83E | 30 | 0.217582335269065 |  |  |  |  |  |  |
| FAM83E | 30 | 0.242156471934705 |  |  |  |  |  |  |
| FAM83E | 30 | 0.251608049416859 |  |  |  |  |  |  |
| FAM83E | 30 | 0.262258784791312 |  |  |  |  |  |  |
| FAM83E | 30 | 0.279860098783052 |  |  |  |  |  |  |
| FAM83E | 30 | 0.256119313055452 |  |  |  |  |  |  |
| FAM83E | 30 | 0.263711972035244 |  |  |  |  |  |  |
| FAM83E | 30 | 0.236585367147834 |  |  |  |  |  |  |
| FAM83E | 30 | 0.258703858824203 |  |  |  |  |  |  |
| FAM83E | 30 | 0.244384440605814 |  |  |  |  |  |  |
| FAM83E | 30 | 0.26632110302035 |  |  |  |  |  |  |
| FAM83E | 30 | 0.289597599090174 |  |  |  |  |  |  |
| FAM83E | 30 | 0.312084221973114 |  |  |  |  |  |  |
| FAM83E | 30 | 0.317742108400373 |  |  |  |  |  |  |
| FAM83E | 30 | 0.339263896630826 |  |  |  |  |  |  |
| FAM83E | 30 | 0.353539642955546 |  |  |  |  |  |  |
| FAM83E | 30 | 0.383791807724526 |  |  |  |  |  |  |
| FAM83E | 31 | 0.350050326701012 |  |  |  |  |  |  |
| FAM83E | 31 | 0.338074712277904 |  |  |  |  |  |  |
| FAM83E | 31 | 0.310034012091267 |  |  |  |  |  |  |
| FAM83E | 31 | 0.333167558806134 |  |  |  |  |  |  |
| FAM83E | 31 | 0.329793194614125 |  |  |  |  |  |  |
| FAM83E | 31 | 0.31243505143464 |  |  |  |  |  |  |
| FAM83E | 31 | 0.285107851544272 |  |  |  |  |  |  |
| FAM83E | 31 | 0.30531784577167 |  |  |  |  |  |  |
| FAM83E | 31 | 0.274841917149548 |  |  |  |  |  |  |
| FAM83E | 31 | 0.289689582537979 |  |  |  |  |  |  |
| FAM83E | 31 | 0.302802905920216 |  |  |  |  |  |  |
| FAM83E | 31 | 0.287005971725864 |  |  |  |  |  |  |
| FAM83E | 31 | 0.301586275893734 |  |  |  |  |  |  |
| FAM83E | 31 | 0.280829352362892 |  |  |  |  |  |  |
| FAM83E | 31 | 0.288953112679249 |  |  |  |  |  |  |
| FAM83E | 31 | 0.266437259095469 |  |  |  |  |  |  |
| FAM83E | 31 | 0.291144412362476 |  |  |  |  |  |  |
| FAM83E | 31 | 0.26498998262799 |  |  |  |  |  |  |
| FAM83E | 31 | 0.304303324859439 |  |  |  |  |  |  |
| FAM83E | 31 | 0.314282948002472 |  |  |  |  |  |  |
| FAM83E | 31 | 0.332575352069987 |  |  |  |  |  |  |
| FAM83E | 32 | 0.352752239444214 |  |  |  |  |  |  |
| FAM83E | 32 | 0.331722093279881 |  |  |  |  |  |  |
| FAM83E | 32 | 0.359717832266465 |  |  |  |  |  |  |
| FAM83E | 32 | 0.343634259310419 |  |  |  |  |  |  |
| FAM83E | 32 | 0.317500701705282 |  |  |  |  |  |  |
| FAM83E | 32 | 0.307155374337525 |  |  |  |  |  |  |
| FAM83E | 32 | 0.285226185409813 |  |  |  |  |  |  |
| FAM83E | 32 | 0.268348652329075 |  |  |  |  |  |  |
| FAM83E | 32 | 0.271196269923836 |  |  |  |  |  |  |
| FAM83E | 32 | 0.250874259638349 |  |  |  |  |  |  |
| FAM83E | 32 | 0.269262918467101 |  |  |  |  |  |  |
| FAM83E | 32 | 0.287634346911588 |  |  |  |  |  |  |
| FAM83E | 32 | 0.304534339875997 |  |  |  |  |  |  |
| FAM83E | 32 | 0.315951684284832 |  |  |  |  |  |  |
| FAM83E | 32 | 0.335734119913388 |  |  |  |  |  |  |
| FAM83E | 32 | 0.348514167895729 |  |  |  |  |  |  |
| FAM83E | 32 | 0.336949315546251 |  |  |  |  |  |  |
| FAM83E | 33 | 0.304657745705758 |  |  |  |  |  |  |
| FAM83E | 33 | 0.324747211869315 |  |  |  |  |  |  |
| FAM83E | 33 | 0.298934624241103 |  |  |  |  |  |  |
| FAM83E | 33 | 0.325467165012264 |  |  |  |  |  |  |
| FAM83E | 33 | 0.352322164482102 |  |  |  |  |  |  |
| FAM83E | 33 | 0.378729226282937 |  |  |  |  |  |  |
| FAM83E | 33 | 0.381672800912134 |  |  |  |  |  |  |
| FAM83E | 33 | 0.410097265058412 |  |  |  |  |  |  |
| FAM83E | 33 | 0.432856638932666 |  |  |  |  |  |  |
| FAM83E | 33 | 0.450064987814446 |  |  |  |  |  |  |
| FAM83E | 33 | 0.422514914734653 |  |  |  |  |  |  |
| FAM83E | 33 | 0.387138778064295 |  |  |  |  |  |  |
| FAM83E | 33 | 0.414102577870388 |  |  |  |  |  |  |
| FAM83E | 33 | 0.438174652039528 |  |  |  |  |  |  |
| FAM83E | 33 | 0.419680034753096 |  |  |  |  |  |  |
| FAM83E | 34 | 0.445299607857759 |  |  |  |  |  |  |
| FAM83E | 34 | 0.471812886227514 |  |  |  |  |  |  |
| FAM83E | 34 | 0.436015462169697 |  |  |  |  |  |  |
| FAM83E | 34 | 0.478917648113885 |  |  |  |  |  |  |
| FAM83E | 34 | 0.468576539427129 |  |  |  |  |  |  |
| FAM83E | 34 | 0.491368466112151 |  |  |  |  |  |  |
| FAM83E | 34 | 0.502124170602697 |  |  |  |  |  |  |
| FAM83E | 34 | 0.463705825649775 |  |  |  |  |  |  |
| FAM83E | 34 | 0.468156051536301 |  |  |  |  |  |  |
| FAM83E | 34 | 0.45216241528421 |  |  |  |  |  |  |
| FAM83E | 34 | 0.455293391910205 |  |  |  |  |  |  |
| FAM83E | 34 | 0.477413457019618 |  |  |  |  |  |  |
| FAM83E | 34 | 0.507253668128304 |  |  |  |  |  |  |
| FAM83E | 34 | 0.53673774819251 |  |  |  |  |  |  |
| FAM83E | 35 | 0.501997368643105 |  |  |  |  |  |  |
| FAM83E | 35 | 0.466856082987585 |  |  |  |  |  |  |
| FAM83E | 35 | 0.522365781338146 |  |  |  |  |  |  |
| FAM83E | 35 | 0.489112960768314 |  |  |  |  |  |  |
| FAM83E | 35 | 0.466862940741824 |  |  |  |  |  |  |
| FAM83E | 35 | 0.428886193576567 |  |  |  |  |  |  |
| FAM83E | 35 | 0.44129875227147 |  |  |  |  |  |  |
| FAM83E | 35 | 0.468638359564891 |  |  |  |  |  |  |
| FAM83E | 35 | 0.427552782846326 |  |  |  |  |  |  |
| FAM83E | 35 | 0.390365320063098 |  |  |  |  |  |  |
| FAM83E | 35 | 0.392540227731244 |  |  |  |  |  |  |
| FAM83E | 35 | 0.405377020239157 |  |  |  |  |  |  |
| FAM83E | 35 | 0.427382471741177 |  |  |  |  |  |  |
| FAM83E | 36 | 0.417718945299156 |  |  |  |  |  |  |
| FAM83E | 36 | 0.385995158192583 |  |  |  |  |  |  |
| FAM83E | 36 | 0.351182974603904 |  |  |  |  |  |  |
| FAM83E | 36 | 0.319803782790007 |  |  |  |  |  |  |
| FAM83E | 36 | 0.347942846566148 |  |  |  |  |  |  |
| FAM83E | 36 | 0.358199165063173 |  |  |  |  |  |  |
| FAM83E | 36 | 0.388719748861821 |  |  |  |  |  |  |
| FAM83E | 36 | 0.374970099665225 |  |  |  |  |  |  |
| FAM83E | 36 | 0.357476304289005 |  |  |  |  |  |  |
| FAM83E | 36 | 0.325075187846704 |  |  |  |  |  |  |
| FAM83E | 36 | 0.327322253830537 |  |  |  |  |  |  |
| FAM83E | 36 | 0.308209104214894 |  |  |  |  |  |  |
| FAM83E | 36 | 0.282957158589115 |  |  |  |  |  |  |
| FAM83E | 36 | 0.312722819547015 |  |  |  |  |  |  |
| FAM83E | 36 | 0.323367743489469 |  |  |  |  |  |  |
| FAM83E | 36 | 0.339680761050629 |  |  |  |  |  |  |
| FAM83E | 36 | 0.368930932769575 |  |  |  |  |  |  |
| FAM83E | 36 | 0.354056100254792 |  |  |  |  |  |  |
| FAM83E | 37 | 0.330772845515259 |  |  |  |  |  |  |
| FAM83E | 37 | 0.348517753511838 |  |  |  |  |  |  |
| FAM83E | 37 | 0.334628768365167 |  |  |  |  |  |  |
| FAM83E | 37 | 0.350670797753521 |  |  |  |  |  |  |
| FAM83E | 37 | 0.367905239333109 |  |  |  |  |  |  |
| FAM83E | 37 | 0.380293387060466 |  |  |  |  |  |  |
| FAM83E | 37 | 0.364029533253454 |  |  |  |  |  |  |
| FAM83E | 37 | 0.360581243018847 |  |  |  |  |  |  |
| FAM83E | 37 | 0.392951515973372 |  |  |  |  |  |  |
| FAM83E | 37 | 0.436222166361376 |  |  |  |  |  |  |
| FAM83E | 37 | 0.408617811758401 |  |  |  |  |  |  |
| FAM83E | 38 | 0.461965117412734 |  |  |  |  |  |  |
| FAM83E | 38 | 0.493778418670684 |  |  |  |  |  |  |
| FAM83E | 38 | 0.468874029477698 |  |  |  |  |  |  |
| FAM83E | 38 | 0.488478288948274 |  |  |  |  |  |  |
| FAM83E | 38 | 0.511329436659751 |  |  |  |  |  |  |
| FAM83E | 38 | 0.473932329983801 |  |  |  |  |  |  |
| FAM83E | 38 | 0.498377121952372 |  |  |  |  |  |  |
| FAM83E | 38 | 0.54530457547893 |  |  |  |  |  |  |
| FAM83E | 38 | 0.581194145871035 |  |  |  |  |  |  |
| FAM83E | 38 | 0.609162937444459 |  |  |  |  |  |  |
| FAM83E | 38 | 0.652391416008715 |  |  |  |  |  |  |
| FAM83E | 38 | 0.630562904877397 |  |  |  |  |  |  |
| FAM83E | 39 | 0.649639480508489 |  |  |  |  |  |  |
| FAM83E | 39 | 0.604921209428405 |  |  |  |  |  |  |
| FAM83E | 39 | 0.5756409468708 |  |  |  |  |  |  |
| FAM83E | 39 | 0.584901699863664 |  |  |  |  |  |  |
| FAM83E | 39 | 0.600083467477847 |  |  |  |  |  |  |
| FAM83E | 39 | 0.623879725531292 |  |  |  |  |  |  |
| FAM83E | 39 | 0.686031876350687 |  |  |  |  |  |  |
| FAM83E | 39 | 0.718825065903364 |  |  |  |  |  |  |
| FAM83E | 39 | 0.745826753607508 |  |  |  |  |  |  |
| FAM83E | 39 | 0.698607650164905 |  |  |  |  |  |  |
| FAM83E | 39 | 0.734308828941903 |  |  |  |  |  |  |
| FAM83E | 40 | 0.761538212199824 |  |  |  |  |  |  |
| FAM83E | 40 | 0.71009778878229 |  |  |  |  |  |  |
| FAM83E | 40 | 0.659872596065242 |  |  |  |  |  |  |
| FAM83E | 40 | 0.645903962370017 |  |  |  |  |  |  |
| FAM83E | 40 | 0.629244035400507 |  |  |  |  |  |  |
| FAM83E | 40 | 0.659209705630306 |  |  |  |  |  |  |
| FAM83E | 40 | 0.66397762355305 |  |  |  |  |  |  |
| FAM83E | 40 | 0.667367104260931 |  |  |  |  |  |  |
| FAM83E | 40 | 0.687043397803713 |  |  |  |  |  |  |
| FAM83E | 40 | 0.707310887858141 |  |  |  |  |  |  |
| FAM83E | 40 | 0.734702734424159 |  |  |  |  |  |  |
| FAM83E | 40 | 0.771956652244815 |  |  |  |  |  |  |
| FAM83E | 41 | 0.750412907850347 |  |  |  |  |  |  |
| FAM83E | 41 | 0.70308604326221 |  |  |  |  |  |  |
| FAM83E | 41 | 0.726112416585436 |  |  |  |  |  |  |
| FAM83E | 41 | 0.689053238686592 |  |  |  |  |  |  |
| FAM83E | 41 | 0.669662020954351 |  |  |  |  |  |  |
| FAM83E | 41 | 0.633700468559392 |  |  |  |  |  |  |
| FAM83E | 41 | 0.638560076989568 |  |  |  |  |  |  |
| FAM83E | 41 | 0.642898934459684 |  |  |  |  |  |  |
| FAM83E | 41 | 0.67430580216071 |  |  |  |  |  |  |
| FAM83E | 41 | 0.639751534483818 |  |  |  |  |  |  |
| FAM83E | 41 | 0.672935845872672 |  |  |  |  |  |  |
| FAM83E | 41 | 0.72403726535783 |  |  |  |  |  |  |
| FAM83E | 42 | 0.691738109734977 |  |  |  |  |  |  |
| FAM83E | 42 | 0.738661673679516 |  |  |  |  |  |  |
| FAM83E | 42 | 0.768567678566912 |  |  |  |  |  |  |
| FAM83E | 42 | 0.773207860699271 |  |  |  |  |  |  |
| FAM83E | 42 | 0.776452424430882 |  |  |  |  |  |  |
| FAM83E | 42 | 0.727631153940365 |  |  |  |  |  |  |
| FAM83E | 42 | 0.745214024834698 |  |  |  |  |  |  |
| FAM83E | 42 | 0.789298841807381 |  |  |  |  |  |  |
| FAM83E | 42 | 0.840618187863363 |  |  |  |  |  |  |
| FAM83E | 42 | 0.89450338061568 |  |  |  |  |  |  |
| FAM83E | 42 | 0.929725074876029 |  |  |  |  |  |  |
| FAM83E | 42 | 0.971719066422125 |  |  |  |  |  |  |
| FAM83E | 42 | 0.983626834283281 |  |  |  |  |  |  |
| FAM83E | 43 | 0.978103220744549 |  |  |  |  |  |  |
| FAM83E | 43 | 0.973298846778241 |  |  |  |  |  |  |
| FAM83E | 43 | 0.947543192142892 |  |  |  |  |  |  |
| FAM83E | 43 | 0.991046903275067 |  |  |  |  |  |  |
| FAM83E | 43 | 0.93388746803341 |  |  |  |  |  |  |
| FAM83E | 43 | 0.958314091076124 |  |  |  |  |  |  |
| FAM83E | 43 | 0.935523933674063 |  |  |  |  |  |  |
| FAM83E | 43 | 0.949322915572486 |  |  |  |  |  |  |
| FAM83E | 43 | 0.903361332826732 |  |  |  |  |  |  |
| FAM83E | 43 | 0.945148998840733 |  |  |  |  |  |  |
| FAM83E | 43 | 0.906649844718143 |  |  |  |  |  |  |
| FAM83E | 43 | 0.963808055084027 |  |  |  |  |  |  |
| FAM83E | 43 | 0.999854855084184 |  |  |  |  |  |  |
| FAM83E | 43 | 0.963315196530364 |  |  |  |  |  |  |
| FAM83E | 44 | 0.997914838783886 |  |  |  |  |  |  |
| FAM83E | 44 | 0.96022432947073 |  |  |  |  |  |  |
| FAM83E | 44 | 0.97053315069927 |  |  |  |  |  |  |
| FAM83E | 44 | 0.98907708791424 |  |  |  |  |  |  |
| FAM83E | 44 | 0.992050127143136 |  |  |  |  |  |  |
| FAM83E | 44 | 0.957459556210235 |  |  |  |  |  |  |
| FAM83E | 44 | 0.999808781978869 |  |  |  |  |  |  |
| FAM83E | 44 | 0.954437695243185 |  |  |  |  |  |  |
| FAM83E | 44 | 0.992623529501843 |  |  |  |  |  |  |
| FAM83E | 44 | 0.998349758458392 |  |  |  |  |  |  |
| FAM83E | 44 | 0.957337585005614 |  |  |  |  |  |  |
| FAM83E | 45 | 0.997626317238823 |  |  |  |  |  |  |
| FAM83E | 45 | 0.985314513108053 |  |  |  |  |  |  |
| FAM83E | 45 | 0.941009774141486 |  |  |  |  |  |  |
| FAM83E | 45 | 0.902604640731718 |  |  |  |  |  |  |
| FAM83E | 45 | 0.871968471584785 |  |  |  |  |  |  |
| FAM83E | 45 | 0.897588944674418 |  |  |  |  |  |  |
| FAM83E | 45 | 0.951519711880342 |  |  |  |  |  |  |
| FAM83E | 45 | 0.955295398340722 |  |  |  |  |  |  |
| FAM83E | 45 | 0.959942505610468 |  |  |  |  |  |  |
| FAM83E | 45 | 0.98931403402609 |  |  |  |  |  |  |
| FAM83E | 45 | 0.936388404159104 |  |  |  |  |  |  |
| FAM83E | 45 | 0.985900528691009 |  |  |  |  |  |  |
| FAM83E | 45 | 0.979718307316464 |  |  |  |  |  |  |
| FAM83E | 45 | 0.936660744530104 |  |  |  |  |  |  |
| FAM83E | 46 | 0.905683051439166 |  |  |  |  |  |  |
| FAM83E | 46 | 0.866795645787054 |  |  |  |  |  |  |
| FAM83E | 46 | 0.865280669272488 |  |  |  |  |  |  |
| FAM83E | 46 | 0.826156006112383 |  |  |  |  |  |  |
| FAM83E | 46 | 0.790850471362152 |  |  |  |  |  |  |
| FAM83E | 46 | 0.750145829751143 |  |  |  |  |  |  |
| FAM83E | 46 | 0.72452666386753 |  |  |  |  |  |  |
| FAM83E | 46 | 0.690396221733499 |  |  |  |  |  |  |
| FAM83E | 47 | 0.685345508476925 |  |  |  |  |  |  |
| FAM83E | 47 | 0.62083892235434 |  |  |  |  |  |  |
| FAM83E | 47 | 0.552627791856543 |  |  |  |  |  |  |
| FAM83E | 47 | 0.511961942070354 |  |  |  |  |  |  |
| FAM83E | 47 | 0.556009232001435 |  |  |  |  |  |  |
| FAM83E | 47 | 0.513761683547764 |  |  |  |  |  |  |
| FAM83E | 47 | 0.507979512977413 |  |  |  |  |  |  |
| FAM83E | 47 | 0.556301716547784 |  |  |  |  |  |  |
| FAM83E | 47 | 0.604600501352614 |  |  |  |  |  |  |
| FAM83E | 47 | 0.583066242714282 |  |  |  |  |  |  |
| FAM83F | 56 | 0.000125867724005585 |  |  |  |  |  |  |
| FAM83F | 56 | 0.000160028617379253 |  |  |  |  |  |  |
| FAM83F | 56 | 0.000130046664970959 |  |  |  |  |  |  |
| FAM83F | 56 | 0.00010588225455211 |  |  |  |  |  |  |
| FAM83F | 57 | 8.49801429222578e-05 |  |  |  |  |  |  |
| FAM83F | 57 | 6.61273001701967e-05 |  |  |  |  |  |  |
| FAM83F | 57 | 8.4219873992202e-05 |  |  |  |  |  |  |
| FAM83F | 57 | 0.000118027139284544 |  |  |  |  |  |  |
| FAM83F | 57 | 0.000139486918224812 |  |  |  |  |  |  |
| FAM83F | 57 | 0.000197585265472543 |  |  |  |  |  |  |
| FAM83F | 57 | 0.000262447521839563 |  |  |  |  |  |  |
| FAM83F | 57 | 0.000324582588889223 |  |  |  |  |  |  |
| FAM83F | 57 | 0.000427092132804203 |  |  |  |  |  |  |
| FAM83F | 57 | 0.000375029997280606 |  |  |  |  |  |  |
| FAM83F | 58 | 0.000305968553535107 |  |  |  |  |  |  |
| FAM83F | 58 | 0.000267500991896783 |  |  |  |  |  |  |
| FAM83F | 58 | 0.000266474470266152 |  |  |  |  |  |  |
| FAM83F | 58 | 0.000207286072055957 |  |  |  |  |  |  |
| FAM83F | 58 | 0.000139709462083962 |  |  |  |  |  |  |
| FAM83F | 58 | 0.000113389569324468 |  |  |  |  |  |  |
| FAM83F | 58 | 9.05736535949937e-05 |  |  |  |  |  |  |
| FAM83F | 58 | 0.000122643181657321 |  |  |  |  |  |  |
| FAM83F | 59 | 0.000106063524181452 |  |  |  |  |  |  |
| FAM83F | 59 | 8.60783224794435e-05 |  |  |  |  |  |  |
| FAM83F | 59 | 7.05123827956711e-05 |  |  |  |  |  |  |
| FAM83F | 59 | 0.000103399831026542 |  |  |  |  |  |  |
| FAM83F | 59 | 0.000115347188659608 |  |  |  |  |  |  |
| FAM83F | 59 | 0.000100361042550901 |  |  |  |  |  |  |
| FAM83F | 60 | 8.60932005621281e-05 |  |  |  |  |  |  |
| FAM83F | 60 | 8.95819862737814e-05 |  |  |  |  |  |  |
| FAM83F | 60 | 0.000104765489429771 |  |  |  |  |  |  |
| FAM83F | 60 | 0.000125630703132296 |  |  |  |  |  |  |
| FAM83F | 60 | 0.000107473893110919 |  |  |  |  |  |  |
| FAM83F | 60 | 8.93356267833273e-05 |  |  |  |  |  |  |
| FAM83F | 60 | 7.01907018901033e-05 |  |  |  |  |  |  |
| FAM83F | 61 | 5.90485119362761e-05 |  |  |  |  |  |  |
| FAM83F | 61 | 7.68785045175511e-05 |  |  |  |  |  |  |
| FAM83F | 61 | 0.000101577853691546 |  |  |  |  |  |  |
| FAM83F | 61 | 0.000131797800201722 |  |  |  |  |  |  |
| FAM83F | 61 | 0.000106002912566213 |  |  |  |  |  |  |
| FAM83F | 61 | 0.000151923499795539 |  |  |  |  |  |  |
| FAM83F | 61 | 0.000109198288153511 |  |  |  |  |  |  |
| FAM83F | 61 | 8.62931066314283e-05 |  |  |  |  |  |  |
| FAM83F | 62 | 6.81754003540789e-05 |  |  |  |  |  |  |
| FAM83F | 62 | 6.48508026610542e-05 |  |  |  |  |  |  |
| FAM83F | 62 | 4.523756751211e-05 |  |  |  |  |  |  |
| FAM83F | 62 | 6.26322808711708e-05 |  |  |  |  |  |  |
| FAM83F | 62 | 6.18591506611027e-05 |  |  |  |  |  |  |
| FAM83F | 62 | 5.05078155758783e-05 |  |  |  |  |  |  |
| FAM83F | 62 | 4.11907925904329e-05 |  |  |  |  |  |  |
| FAM83F | 62 | 3.41183646815209e-05 |  |  |  |  |  |  |
| FAM83F | 63 | 2.77853444410033e-05 |  |  |  |  |  |  |
| FAM83F | 63 | 3.23083231654968e-05 |  |  |  |  |  |  |
| FAM83F | 63 | 4.49874845870921e-05 |  |  |  |  |  |  |
| FAM83F | 63 | 5.36524480336914e-05 |  |  |  |  |  |  |
| FAM83F | 63 | 5.78615939039559e-05 |  |  |  |  |  |  |
| FAM83F | 63 | 4.29737661211487e-05 |  |  |  |  |  |  |
| FAM83F | 64 | 3.4571286212074e-05 |  |  |  |  |  |  |
| FAM83F | 64 | 4.90286174218283e-05 |  |  |  |  |  |  |
| FAM83F | 64 | 6.6036005227538e-05 |  |  |  |  |  |  |
| FAM83F | 64 | 8.40489536909617e-05 |  |  |  |  |  |  |
| FAM83F | 64 | 6.47145026504349e-05 |  |  |  |  |  |  |
| FAM83F | 64 | 5.25177599122569e-05 |  |  |  |  |  |  |
| FAM83F | 64 | 4.08340409754534e-05 |  |  |  |  |  |  |
| FAM83F | 64 | 3.52773182822494e-05 |  |  |  |  |  |  |
| FAM83F | 64 | 2.68215398892141e-05 |  |  |  |  |  |  |
| FAM83F | 64 | 2.46583652779756e-05 |  |  |  |  |  |  |
| FAM83F | 65 | 1.96051137835625e-05 |  |  |  |  |  |  |
| FAM83F | 66 | 1.50287359091948e-05 |  |  |  |  |  |  |
| FAM83F | 66 | 2.03663065464957e-05 |  |  |  |  |  |  |
| FAM83F | 66 | 1.55470965424713e-05 |  |  |  |  |  |  |
| FAM83F | 66 | 1.14374788666128e-05 |  |  |  |  |  |  |
| FAM83F | 66 | 9.63130507445502e-06 |  |  |  |  |  |  |
| FAM83F | 66 | 1.28440121775081e-05 |  |  |  |  |  |  |
| FAM83F | 66 | 9.73878081667509e-06 |  |  |  |  |  |  |
| FAM83F | 67 | 6.70011217697689e-06 |  |  |  |  |  |  |
| FAM83F | 67 | 9.81346155418644e-06 |  |  |  |  |  |  |
| FAM83F | 67 | 7.22943255277832e-06 |  |  |  |  |  |  |
| FAM83F | 67 | 5.34158173698583e-06 |  |  |  |  |  |  |
| FAM83F | 68 | 4.28680200451279e-06 |  |  |  |  |  |  |
| FAM83F | 68 | 3.39392780764738e-06 |  |  |  |  |  |  |
| FAM83F | 68 | 1.91831930877534e-06 |  |  |  |  |  |  |
| FAM83F | 68 | 1.49987021859163e-06 |  |  |  |  |  |  |
| FAM83F | 68 | 2.32052656900451e-06 |  |  |  |  |  |  |
| FAM83F | 68 | 1.8954384416214e-06 |  |  |  |  |  |  |
| FAM83F | 68 | 1.40323133253495e-06 |  |  |  |  |  |  |
| FAM83F | 69 | 1.09423031245768e-06 |  |  |  |  |  |  |
| FAM83F | 69 | 1.6693365528604e-06 |  |  |  |  |  |  |
| FAM83F | 69 | 1.02423865956694e-06 |  |  |  |  |  |  |
| FAM83F | 69 | 1.3257029192651e-06 |  |  |  |  |  |  |
| FAM83F | 69 | 1.26789421366207e-06 |  |  |  |  |  |  |
| FAM83F | 69 | 9.88311496733271e-07 |  |  |  |  |  |  |
| FAM83F | 69 | 7.83588309718746e-07 |  |  |  |  |  |  |
| FAM83F | 69 | 6.10171259490856e-07 |  |  |  |  |  |  |
| FAM83F | 69 | 4.73145564330307e-07 |  |  |  |  |  |  |
| FAM83F | 70 | 3.51099066737054e-07 |  |  |  |  |  |  |
| FAM83F | 70 | 5.23108227791978e-07 |  |  |  |  |  |  |
| FAM83F | 70 | 7.49375583678102e-07 |  |  |  |  |  |  |
| FAM83F | 70 | 9.93215271720946e-07 |  |  |  |  |  |  |
| FAM83F | 70 | 1.39468649322082e-06 |  |  |  |  |  |  |
| FAM83F | 70 | 1.78222167322603e-06 |  |  |  |  |  |  |
| FAM83F | 70 | 2.60698148502508e-06 |  |  |  |  |  |  |
| FAM83F | 70 | 2.05745139222068e-06 |  |  |  |  |  |  |
| FAM83F | 71 | 1.57275307232203e-06 |  |  |  |  |  |  |
| FAM83F | 71 | 1.81379592666431e-06 |  |  |  |  |  |  |
| FAM83F | 71 | 1.45586028324702e-06 |  |  |  |  |  |  |
| FAM83F | 71 | 1.21608650022714e-06 |  |  |  |  |  |  |
| FAM83F | 71 | 9.55202959340205e-07 |  |  |  |  |  |  |
| FAM83F | 72 | 7.53410632436924e-07 |  |  |  |  |  |  |
| FAM83F | 72 | 1.15870297363016e-06 |  |  |  |  |  |  |
| FAM83F | 72 | 8.96799616746863e-07 |  |  |  |  |  |  |
| FAM83F | 72 | 7.36292527418347e-07 |  |  |  |  |  |  |
| FAM83F | 73 | 5.11497956751197e-07 |  |  |  |  |  |  |
| FAM83F | 73 | 3.78039528316619e-07 |  |  |  |  |  |  |
| FAM83F | 73 | 4.85878961830694e-07 |  |  |  |  |  |  |
| FAM83F | 73 | 3.60136003119072e-07 |  |  |  |  |  |  |
| FAM83F | 73 | 4.17094137192852e-07 |  |  |  |  |  |  |
| FAM83F | 73 | 3.63300873802343e-07 |  |  |  |  |  |  |
| FAM83F | 73 | 5.26116955678403e-07 |  |  |  |  |  |  |
| FAM83F | 73 | 3.3216239270222e-07 |  |  |  |  |  |  |
| FAM83F | 73 | 2.65106079910511e-07 |  |  |  |  |  |  |
| FAM83F | 74 | 1.77518596616669e-07 |  |  |  |  |  |  |
| FAM83F | 74 | 1.35024333693213e-07 |  |  |  |  |  |  |
| FAM83F | 74 | 8.35185276975157e-08 |  |  |  |  |  |  |
| FAM83F | 74 | 6.56250173569914e-08 |  |  |  |  |  |  |
| FAM83F | 75 | 4.6351098343256e-08 |  |  |  |  |  |  |
| FAM83F | 75 | 5.9975334594151e-08 |  |  |  |  |  |  |
| FAM83F | 75 | 8.35434907395788e-08 |  |  |  |  |  |  |
| FAM83F | 75 | 1.22702520327581e-07 |  |  |  |  |  |  |
| FAM83F | 75 | 9.9156561665432e-08 |  |  |  |  |  |  |
| FAM83F | 76 | 7.57662579427091e-08 |  |  |  |  |  |  |
| FAM83F | 76 | 1.14248256916576e-07 |  |  |  |  |  |  |
| FAM83F | 76 | 7.56903501000171e-08 |  |  |  |  |  |  |
| FAM83F | 76 | 9.40411279636642e-08 |  |  |  |  |  |  |
| FAM83F | 76 | 1.47003360515373e-07 |  |  |  |  |  |  |
| FAM83F | 76 | 1.99347786710016e-07 |  |  |  |  |  |  |
| FAM83F | 76 | 1.73466883012229e-07 |  |  |  |  |  |  |
| FAM83F | 76 | 2.60235791054241e-07 |  |  |  |  |  |  |
| FAM83F | 76 | 3.45368524832508e-07 |  |  |  |  |  |  |
| FAM83F | 76 | 2.50950393061997e-07 |  |  |  |  |  |  |
| FAM83F | 76 | 2.05352277110349e-07 |  |  |  |  |  |  |
| FAM83F | 77 | 1.6611177168176e-07 |  |  |  |  |  |  |
| FAM83F | 77 | 1.94273363172021e-07 |  |  |  |  |  |  |
| FAM83F | 77 | 1.38085077241169e-07 |  |  |  |  |  |  |
| FAM83F | 78 | 9.51951762216409e-08 |  |  |  |  |  |  |
| FAM83F | 78 | 8.23267951888598e-08 |  |  |  |  |  |  |
| FAM83F | 78 | 7.57306983229383e-08 |  |  |  |  |  |  |
| FAM83F | 78 | 1.19215844596137e-07 |  |  |  |  |  |  |
| FAM83F | 79 | 9.46766861840464e-08 |  |  |  |  |  |  |
| FAM83F | 79 | 1.17067970536259e-07 |  |  |  |  |  |  |
| FAM83F | 79 | 9.22628634546358e-08 |  |  |  |  |  |  |
| FAM83F | 80 | 7.35988586788344e-08 |  |  |  |  |  |  |
| FAM83F | 80 | 1.0107747378034e-07 |  |  |  |  |  |  |
| FAM83F | 80 | 6.54158992457571e-08 |  |  |  |  |  |  |
| FAM83F | 80 | 5.12878243703462e-08 |  |  |  |  |  |  |
| FAM83F | 80 | 8.1680066544414e-08 |  |  |  |  |  |  |
| FAM83F | 81 | 5.92782479379943e-08 |  |  |  |  |  |  |
| FAM83F | 81 | 4.52374073534373e-08 |  |  |  |  |  |  |
| FAM83F | 82 | 3.67476271888478e-08 |  |  |  |  |  |  |
| FAM83F | 82 | 5.35316266880906e-08 |  |  |  |  |  |  |
| FAM83F | 82 | 5.20072879693506e-08 |  |  |  |  |  |  |
| FAM83F | 82 | 3.84128633502274e-08 |  |  |  |  |  |  |
| FAM83F | 82 | 2.84648327780807e-08 |  |  |  |  |  |  |
| FAM83F | 83 | 2.12777952979302e-08 |  |  |  |  |  |  |
| FAM83F | 83 | 2.35973495338498e-08 |  |  |  |  |  |  |
| FAM83F | 83 | 3.814670202513e-08 |  |  |  |  |  |  |
| FAM83F | 83 | 5.83428445251625e-08 |  |  |  |  |  |  |
| FAM83F | 83 | 7.79789514015653e-08 |  |  |  |  |  |  |
| FAM83F | 84 | 1.17622248974974e-07 |  |  |  |  |  |  |
| FAM83F | 84 | 1.04683648567734e-07 |  |  |  |  |  |  |
| FAM83F | 84 | 8.73620563483832e-08 |  |  |  |  |  |  |
| FAM83F | 84 | 6.69851086700667e-08 |  |  |  |  |  |  |
| FAM83F | 84 | 1.01701772372325e-07 |  |  |  |  |  |  |
| FAM83F | 84 | 8.37049590064427e-08 |  |  |  |  |  |  |
| FAM83F | 85 | 5.5396068155015e-08 |  |  |  |  |  |  |
| FAM83F | 85 | 8.47163745930946e-08 |  |  |  |  |  |  |
| FAM83F | 85 | 6.03377784215111e-08 |  |  |  |  |  |  |
| FAM83F | 85 | 7.59956938633964e-08 |  |  |  |  |  |  |
| FAM83F | 85 | 5.95997398011182e-08 |  |  |  |  |  |  |
| FAM83F | 85 | 7.57635169658941e-08 |  |  |  |  |  |  |
| FAM83F | 86 | 5.44626372649261e-08 |  |  |  |  |  |  |
| FAM83F | 86 | 6.44332658661227e-08 |  |  |  |  |  |  |
| FAM83F | 86 | 8.13042394169404e-08 |  |  |  |  |  |  |
| FAM83F | 86 | 9.50601735542413e-08 |  |  |  |  |  |  |
| FAM83F | 86 | 6.64223548823092e-08 |  |  |  |  |  |  |
| FAM83F | 86 | 5.05011595626835e-08 |  |  |  |  |  |  |
| FAM83F | 87 | 3.88595386274984e-08 |  |  |  |  |  |  |
| FAM83F | 87 | 3.31429466845906e-08 |  |  |  |  |  |  |
| FAM83F | 88 | 2.43783506640091e-08 |  |  |  |  |  |  |
| FAM83F | 88 | 1.86673509977011e-08 |  |  |  |  |  |  |
| FAM83F | 88 | 1.51656130092527e-08 |  |  |  |  |  |  |
| FAM83F | 88 | 1.14759854547767e-08 |  |  |  |  |  |  |
| FAM83F | 88 | 1.69946918576566e-08 |  |  |  |  |  |  |
| FAM83F | 88 | 1.26599534059767e-08 |  |  |  |  |  |  |
| FAM83F | 89 | 9.47816997847835e-09 |  |  |  |  |  |  |
| FAM83F | 89 | 5.9359531601058e-09 |  |  |  |  |  |  |
| FAM83F | 89 | 5.70463108664306e-09 |  |  |  |  |  |  |
| FAM83F | 90 | 4.20479398072231e-09 |  |  |  |  |  |  |
| FAM83F | 90 | 6.31897037671348e-09 |  |  |  |  |  |  |
| FAM83F | 90 | 1.01019171189431e-08 |  |  |  |  |  |  |
| FAM83F | 90 | 7.48958672393484e-09 |  |  |  |  |  |  |
| FAM83F | 91 | 6.39764229541715e-09 |  |  |  |  |  |  |
| FAM83F | 91 | 8.45322213145391e-09 |  |  |  |  |  |  |
| FAM83F | 91 | 6.2477583682195e-09 |  |  |  |  |  |  |
| FAM83F | 91 | 7.17388889288135e-09 |  |  |  |  |  |  |
| FAM83F | 91 | 1.08364769027014e-08 |  |  |  |  |  |  |
| FAM83F | 91 | 8.15217411894556e-09 |  |  |  |  |  |  |
| FAM83F | 92 | 7.02896019736965e-09 |  |  |  |  |  |  |
| FAM83F | 92 | 9.51278968259275e-09 |  |  |  |  |  |  |
| FAM83F | 92 | 1.42587183090523e-08 |  |  |  |  |  |  |
| FAM83F | 93 | 1.07697561958551e-08 |  |  |  |  |  |  |
| FAM83F | 93 | 1.48079707767652e-08 |  |  |  |  |  |  |
| FAM83F | 93 | 1.02013262716857e-08 |  |  |  |  |  |  |
| FAM83F | 93 | 1.35890857438082e-08 |  |  |  |  |  |  |
| FAM83F | 93 | 9.49133887299162e-09 |  |  |  |  |  |  |
| FAM83F | 93 | 7.63952393605676e-09 |  |  |  |  |  |  |
| FAM83F | 94 | 6.62213120123997e-09 |  |  |  |  |  |  |
| FAM83F | 94 | 5.5582577250144e-09 |  |  |  |  |  |  |
| FAM83F | 94 | 4.09479265829238e-09 |  |  |  |  |  |  |
| FAM83F | 94 | 2.27646882801471e-09 |  |  |  |  |  |  |
| FAM83F | 94 | 2.69953864250714e-09 |  |  |  |  |  |  |
| FAM83F | 95 | 1.77754899913511e-09 |  |  |  |  |  |  |
| FAM83F | 95 | 1.94278360285633e-09 |  |  |  |  |  |  |
| FAM83F | 95 | 1.43682436495583e-09 |  |  |  |  |  |  |
| FAM83F | 95 | 9.38916379860168e-10 |  |  |  |  |  |  |
| FAM83F | 96 | 6.90350124871799e-10 |  |  |  |  |  |  |
| FAM83F | 96 | 6.81834559583545e-10 |  |  |  |  |  |  |
| FAM83F | 96 | 5.06846891675534e-10 |  |  |  |  |  |  |
| FAM83F | 96 | 3.21350760486564e-10 |  |  |  |  |  |  |
| FAM83F | 96 | 1.5227187015226e-10 |  |  |  |  |  |  |
| FAM83F | 97 | 1.17818581635874e-10 |  |  |  |  |  |  |
| FAM83F | 97 | 6.18487416171111e-11 |  |  |  |  |  |  |
| FAM83F | 97 | 7.50212595261712e-11 |  |  |  |  |  |  |
| FAM83F | 97 | 1.21824029050934e-10 |  |  |  |  |  |  |
| FAM83F | 98 | 1.78375821339172e-10 |  |  |  |  |  |  |
| FAM83F | 98 | 2.92959118936112e-10 |  |  |  |  |  |  |
| FAM83F | 98 | 1.37612070109657e-10 |  |  |  |  |  |  |
| FAM83F | 98 | 1.69091626377407e-10 |  |  |  |  |  |  |
| FAM83F | 98 | 2.17521187134421e-10 |  |  |  |  |  |  |
| FAM83F | 99 | 3.21704860936306e-10 |  |  |  |  |  |  |
| FAM83F | 99 | 2.30759383020133e-10 |  |  |  |  |  |  |
| FAM83F | 99 | 3.57808678251376e-10 |  |  |  |  |  |  |
| FAM83F | 100 | 2.60400295212317e-10 |  |  |  |  |  |  |
| FAM83F | 100 | 4.25744006347791e-10 |  |  |  |  |  |  |
| FAM83F | 100 | 2.59614327773919e-10 |  |  |  |  |  |  |
| FAM83F | 100 | 3.96565604878037e-10 |  |  |  |  |  |  |
| FAM83F | 100 | 2.23103790721106e-10 |  |  |  |  |  |  |
| FAM83F | 100 | 3.33041337599559e-10 |  |  |  |  |  |  |
| FAM83F | 101 | 4.48152403738724e-10 |  |  |  |  |  |  |
| FAM83F | 101 | 6.84987456995723e-10 |  |  |  |  |  |  |
| FAM83F | 101 | 1.03210113949743e-09 |  |  |  |  |  |  |
| FAM83F | 102 | 7.31978290758376e-10 |  |  |  |  |  |  |
| FAM83F | 102 | 6.61197653211902e-10 |  |  |  |  |  |  |
| FAM83F | 102 | 5.78152093522495e-10 |  |  |  |  |  |  |
| FAM83F | 102 | 5.02449047925523e-10 |  |  |  |  |  |  |
| FAM83F | 102 | 8.16877389999498e-10 |  |  |  |  |  |  |
| FAM83F | 102 | 4.47571168470841e-10 |  |  |  |  |  |  |
| FAM83F | 102 | 7.28543761129651e-10 |  |  |  |  |  |  |
| FAM83F | 103 | 9.46185473903538e-10 |  |  |  |  |  |  |
| FAM83F | 103 | 6.86069943399835e-10 |  |  |  |  |  |  |
| FAM83F | 104 | 5.83871779106242e-10 |  |  |  |  |  |  |
| FAM83F | 104 | 9.67669255322961e-10 |  |  |  |  |  |  |
| FAM83F | 104 | 1.59597624102251e-09 |  |  |  |  |  |  |
| FAM83F | 105 | 1.21660871820226e-09 |  |  |  |  |  |  |
| FAM83F | 106 | 1.0375305487024e-09 |  |  |  |  |  |  |
| FAM83F | 106 | 1.55961065059766e-09 |  |  |  |  |  |  |
| FAM83F | 106 | 1.87099260126943e-09 |  |  |  |  |  |  |
| FAM83F | 106 | 2.39983512644491e-09 |  |  |  |  |  |  |
| FAM83F | 106 | 2.6057238134811e-09 |  |  |  |  |  |  |
| FAM83F | 106 | 1.80659690645536e-09 |  |  |  |  |  |  |
| FAM83F | 107 | 1.23948505193045e-09 |  |  |  |  |  |  |
| FAM83F | 107 | 1.91915031077803e-09 |  |  |  |  |  |  |
| FAM83F | 107 | 1.03813773536351e-09 |  |  |  |  |  |  |
| FAM83F | 107 | 1.32515107158028e-09 |  |  |  |  |  |  |
| FAM83F | 108 | 6.29690869935698e-10 |  |  |  |  |  |  |
| FAM83F | 109 | 1.02303178097821e-09 |  |  |  |  |  |  |
| FAM83F | 109 | 1.48748609540284e-09 |  |  |  |  |  |  |
| FAM83F | 109 | 2.1916778596226e-09 |  |  |  |  |  |  |
| FAM83F | 110 | 1.49775006818058e-09 |  |  |  |  |  |  |
| FAM83F | 110 | 2.47126547617429e-09 |  |  |  |  |  |  |
| FAM83F | 110 | 1.70937278380324e-09 |  |  |  |  |  |  |
| FAM83F | 110 | 8.11106507740476e-10 |  |  |  |  |  |  |
| FAM83F | 111 | 5.17580385932647e-10 |  |  |  |  |  |  |
| FAM83F | 111 | 4.01321409369768e-10 |  |  |  |  |  |  |
| FAM83F | 111 | 3.31247786261975e-10 |  |  |  |  |  |  |
| FAM83F | 111 | 5.37177282333507e-10 |  |  |  |  |  |  |
| FAM83F | 111 | 4.69964289144816e-10 |  |  |  |  |  |  |
| FAM83F | 111 | 3.20275825152962e-10 |  |  |  |  |  |  |
| FAM83F | 112 | 4.42694190801271e-10 |  |  |  |  |  |  |
| FAM83F | 112 | 3.19464417583704e-10 |  |  |  |  |  |  |
| FAM83F | 112 | 4.88167418567896e-10 |  |  |  |  |  |  |
| FAM83F | 112 | 7.32542377498362e-10 |  |  |  |  |  |  |
| FAM83F | 112 | 9.22669165663474e-10 |  |  |  |  |  |  |
| FAM83F | 112 | 1.05951764362117e-09 |  |  |  |  |  |  |
| FAM83F | 113 | 8.9251547228689e-10 |  |  |  |  |  |  |
| FAM83F | 113 | 5.89369523725837e-10 |  |  |  |  |  |  |
| FAM83F | 114 | 2.71717862223221e-10 |  |  |  |  |  |  |
| FAM83F | 114 | 3.07894315711803e-10 |  |  |  |  |  |  |
| FAM83F | 114 | 2.1921658111125e-10 |  |  |  |  |  |  |
| FAM83F | 115 | 1.44953258577515e-10 |  |  |  |  |  |  |
| FAM83F | 115 | 1.02511794394389e-10 |  |  |  |  |  |  |
| FAM83F | 115 | 1.00946863812017e-10 |  |  |  |  |  |  |
| FAM83F | 115 | 6.37609561578226e-11 |  |  |  |  |  |  |
| FAM83F | 115 | 8.70373189738424e-11 |  |  |  |  |  |  |
| FAM83F | 115 | 6.21509742272363e-11 |  |  |  |  |  |  |
| FAM83F | 116 | 4.44835325171927e-11 |  |  |  |  |  |  |
| FAM83F | 116 | 2.45628205809413e-11 |  |  |  |  |  |  |
| FAM83F | 117 | 1.98974440857949e-11 |  |  |  |  |  |  |
| FAM83F | 118 | 1.97656688248883e-11 |  |  |  |  |  |  |
| FAM83F | 118 | 3.06887133864903e-11 |  |  |  |  |  |  |
| FAM83F | 118 | 2.09368588747417e-11 |  |  |  |  |  |  |
| FAM83F | 118 | 3.59683959169125e-11 |  |  |  |  |  |  |
| FAM83F | 118 | 5.78768667233141e-11 |  |  |  |  |  |  |
| FAM83F | 118 | 1.00188427796569e-10 |  |  |  |  |  |  |
| FAM83F | 118 | 5.95689772573056e-11 |  |  |  |  |  |  |
| FAM83F | 119 | 4.45583717966613e-11 |  |  |  |  |  |  |
| FAM83F | 119 | 6.57869504424439e-11 |  |  |  |  |  |  |
| FAM83F | 119 | 4.67668605066066e-11 |  |  |  |  |  |  |
| FAM83F | 119 | 3.48688074615052e-11 |  |  |  |  |  |  |
| FAM83F | 120 | 2.51218391774877e-11 |  |  |  |  |  |  |
| FAM83F | 120 | 1.66424715552946e-11 |  |  |  |  |  |  |
| FAM83F | 121 | 2.66241964336209e-11 |  |  |  |  |  |  |
| FAM83F | 121 | 1.84885570689629e-11 |  |  |  |  |  |  |
| FAM83F | 121 | 3.07610393722408e-11 |  |  |  |  |  |  |
| FAM83F | 121 | 2.45910484049513e-11 |  |  |  |  |  |  |
| FAM83F | 121 | 4.12374537291194e-11 |  |  |  |  |  |  |
| FAM83F | 122 | 5.46595729785076e-11 |  |  |  |  |  |  |
| FAM83F | 122 | 2.73897348573391e-11 |  |  |  |  |  |  |
| FAM83F | 122 | 3.79642670126478e-11 |  |  |  |  |  |  |
| FAM83F | 122 | 2.97387460055662e-11 |  |  |  |  |  |  |
| FAM83F | 123 | 4.68851725712875e-11 |  |  |  |  |  |  |
| FAM83F | 123 | 5.32193295386485e-11 |  |  |  |  |  |  |
| FAM83F | 124 | 8.25907136397989e-11 |  |  |  |  |  |  |
| FAM83F | 124 | 1.23830573915319e-10 |  |  |  |  |  |  |
| FAM83F | 124 | 6.4133986412843e-11 |  |  |  |  |  |  |
| FAM83F | 124 | 9.46498736894272e-11 |  |  |  |  |  |  |
| FAM83F | 125 | 1.32395222397786e-10 |  |  |  |  |  |  |
| FAM83F | 125 | 1.51040307919174e-10 |  |  |  |  |  |  |
| FAM83F | 126 | 1.23201901919842e-10 |  |  |  |  |  |  |
| FAM83F | 126 | 1.74657110317157e-10 |  |  |  |  |  |  |
| FAM83F | 127 | 2.99061874522197e-10 |  |  |  |  |  |  |
| FAM83F | 127 | 4.69060806516112e-10 |  |  |  |  |  |  |
| FAM83F | 127 | 6.13546820880903e-10 |  |  |  |  |  |  |
| FAM83F | 127 | 2.76474291058984e-10 |  |  |  |  |  |  |
| FAM83F | 128 | 1.44111987157826e-10 |  |  |  |  |  |  |
| FAM83F | 128 | 1.01869400351892e-10 |  |  |  |  |  |  |
| FAM83F | 128 | 7.79858733697479e-11 |  |  |  |  |  |  |
| FAM83F | 129 | 5.95886736506204e-11 |  |  |  |  |  |  |
| FAM83F | 129 | 7.87660335654478e-11 |  |  |  |  |  |  |
| FAM83F | 129 | 9.86108742514992e-11 |  |  |  |  |  |  |
| FAM83F | 130 | 1.19449498978108e-10 |  |  |  |  |  |  |
| FAM83F | 130 | 7.09091571556132e-11 |  |  |  |  |  |  |
| FAM83F | 131 | 8.303165656794e-11 |  |  |  |  |  |  |
| FAM83F | 131 | 1.30970891161097e-10 |  |  |  |  |  |  |
| FAM83F | 131 | 6.70450496509371e-11 |  |  |  |  |  |  |
| FAM83F | 131 | 4.37026917204267e-11 |  |  |  |  |  |  |
| FAM83F | 132 | 3.14025365125544e-11 |  |  |  |  |  |  |
| FAM83F | 133 | 1.74794947905677e-11 |  |  |  |  |  |  |
| FAM83F | 133 | 3.07876889111381e-11 |  |  |  |  |  |  |
| FAM83F | 133 | 4.23227372951614e-11 |  |  |  |  |  |  |
| FAM83F | 134 | 2.91943771937321e-11 |  |  |  |  |  |  |
| FAM83F | 135 | 4.4008451001869e-11 |  |  |  |  |  |  |
| FAM83F | 135 | 5.69494102329809e-11 |  |  |  |  |  |  |
| FAM83F | 138 | 8.71508641641647e-11 |  |  |  |  |  |  |
| FAM83F | 138 | 1.30863947201806e-10 |  |  |  |  |  |  |
| FAM83F | 139 | 8.03200170283178e-11 |  |  |  |  |  |  |
| FAM83F | 139 | 1.4018067602443e-10 |  |  |  |  |  |  |
| FAM83F | 139 | 2.22195173323542e-10 |  |  |  |  |  |  |
| FAM83F | 140 | 1.26410839513192e-10 |  |  |  |  |  |  |
| FAM83F | 140 | 2.14195539305759e-10 |  |  |  |  |  |  |
| FAM83F | 141 | 2.61930856295574e-10 |  |  |  |  |  |  |
| FAM83F | 141 | 1.71789048019147e-10 |  |  |  |  |  |  |
| FAM83F | 142 | 9.82987098411864e-11 |  |  |  |  |  |  |
| FAM83F | 142 | 1.23739960478083e-10 |  |  |  |  |  |  |
| FAM83F | 143 | 2.02077761440776e-10 |  |  |  |  |  |  |
| FAM83F | 143 | 2.96705503043733e-10 |  |  |  |  |  |  |
| FAM83F | 143 | 4.65680523702517e-10 |  |  |  |  |  |  |
| FAM83F | 144 | 6.0531454624752e-10 |  |  |  |  |  |  |
| FAM83F | 144 | 6.10236738004272e-10 |  |  |  |  |  |  |
| FAM83F | 144 | 9.99513516948997e-10 |  |  |  |  |  |  |
| FAM83F | 144 | 9.68165004851384e-10 |  |  |  |  |  |  |
| FAM83F | 145 | 6.9459659273754e-10 |  |  |  |  |  |  |
| FAM83F | 145 | 4.92628369718848e-10 |  |  |  |  |  |  |
| FAM83F | 145 | 8.31856121213966e-10 |  |  |  |  |  |  |
| FAM83F | 146 | 1.18822815996744e-09 |  |  |  |  |  |  |
| FAM83F | 147 | 6.33249046741554e-10 |  |  |  |  |  |  |
| FAM83F | 147 | 7.64549673845135e-10 |  |  |  |  |  |  |
| FAM83F | 147 | 1.05319008796011e-09 |  |  |  |  |  |  |
| FAM83F | 147 | 7.20836366352101e-10 |  |  |  |  |  |  |
| FAM83F | 148 | 6.20543039611117e-10 |  |  |  |  |  |  |
| FAM83F | 148 | 7.01382222776673e-10 |  |  |  |  |  |  |
| FAM83F | 149 | 1.11776869577894e-09 |  |  |  |  |  |  |
| FAM83F | 150 | 1.29982355572184e-09 |  |  |  |  |  |  |
| FAM83F | 150 | 2.13389376721326e-09 |  |  |  |  |  |  |
| FAM83F | 150 | 2.42707826480926e-09 |  |  |  |  |  |  |
| FAM83F | 150 | 3.85393000925271e-09 |  |  |  |  |  |  |
| FAM83F | 150 | 5.24416178840177e-09 |  |  |  |  |  |  |
| FAM83F | 150 | 8.41422353357117e-09 |  |  |  |  |  |  |
| FAM83F | 151 | 6.01068303725512e-09 |  |  |  |  |  |  |
| FAM83F | 151 | 3.32726966468614e-09 |  |  |  |  |  |  |
| FAM83F | 151 | 5.21414542029351e-09 |  |  |  |  |  |  |
| FAM83F | 151 | 3.53161760376229e-09 |  |  |  |  |  |  |
| FAM83F | 151 | 2.82528347746179e-09 |  |  |  |  |  |  |
| FAM83F | 152 | 1.87128251905405e-09 |  |  |  |  |  |  |
| FAM83F | 152 | 1.28139852703959e-09 |  |  |  |  |  |  |
| FAM83F | 152 | 1.50795516428375e-09 |  |  |  |  |  |  |
| FAM83F | 153 | 2.29517260393792e-09 |  |  |  |  |  |  |
| FAM83F | 153 | 1.89199800845411e-09 |  |  |  |  |  |  |
| FAM83F | 154 | 2.47832636706309e-09 |  |  |  |  |  |  |
| FAM83F | 155 | 2.34302776829497e-09 |  |  |  |  |  |  |
| FAM83F | 155 | 3.28796216449806e-09 |  |  |  |  |  |  |
| FAM83F | 155 | 5.02141186875422e-09 |  |  |  |  |  |  |
| FAM83F | 155 | 3.76115238925094e-09 |  |  |  |  |  |  |
| FAM83F | 156 | 2.79202943663107e-09 |  |  |  |  |  |  |
| FAM83F | 156 | 4.01093610005992e-09 |  |  |  |  |  |  |
| FAM83F | 157 | 3.87192288316583e-09 |  |  |  |  |  |  |
| FAM83F | 158 | 2.91779941515667e-09 |  |  |  |  |  |  |
| FAM83F | 158 | 4.83482355544232e-09 |  |  |  |  |  |  |
| FAM83F | 159 | 4.10225129113907e-09 |  |  |  |  |  |  |
| FAM83F | 159 | 4.96856662407834e-09 |  |  |  |  |  |  |
| FAM83F | 159 | 3.58043511416248e-09 |  |  |  |  |  |  |
| FAM83F | 159 | 5.62293726292592e-09 |  |  |  |  |  |  |
| FAM83F | 159 | 5.86671753884449e-09 |  |  |  |  |  |  |
| FAM83F | 160 | 7.19284321366786e-09 |  |  |  |  |  |  |
| FAM83F | 160 | 8.85410309561818e-09 |  |  |  |  |  |  |
| FAM83F | 160 | 1.1735698807543e-08 |  |  |  |  |  |  |
| FAM83F | 161 | 8.07821169704775e-09 |  |  |  |  |  |  |
| FAM83F | 161 | 4.71785030491076e-09 |  |  |  |  |  |  |
| FAM83F | 161 | 7.50292330649421e-09 |  |  |  |  |  |  |
| FAM83F | 162 | 1.1479824713537e-08 |  |  |  |  |  |  |
| FAM83F | 162 | 1.80545084696538e-08 |  |  |  |  |  |  |
| FAM83F | 162 | 1.31614059636674e-08 |  |  |  |  |  |  |
| FAM83F | 163 | 1.33831841642815e-08 |  |  |  |  |  |  |
| FAM83F | 163 | 8.17694259599401e-09 |  |  |  |  |  |  |
| FAM83F | 163 | 1.30693065823715e-08 |  |  |  |  |  |  |
| FAM83F | 163 | 1.83633833025619e-08 |  |  |  |  |  |  |
| FAM83F | 163 | 2.98708598594532e-08 |  |  |  |  |  |  |
| FAM83F | 164 | 1.84464703759707e-08 |  |  |  |  |  |  |
| FAM83F | 164 | 2.68029760045006e-08 |  |  |  |  |  |  |
| FAM83F | 164 | 1.56677339218928e-08 |  |  |  |  |  |  |
| FAM83F | 164 | 1.2500854286727e-08 |  |  |  |  |  |  |
| FAM83F | 165 | 1.01254983353271e-08 |  |  |  |  |  |  |
| FAM83F | 165 | 4.96304001038229e-09 |  |  |  |  |  |  |
| FAM83F | 166 | 3.52426097515516e-09 |  |  |  |  |  |  |
| FAM83F | 166 | 4.62484362932223e-09 |  |  |  |  |  |  |
| FAM83F | 166 | 3.03106944070024e-09 |  |  |  |  |  |  |
| FAM83F | 167 | 1.68703759816448e-09 |  |  |  |  |  |  |
| FAM83F | 167 | 2.57958180674942e-09 |  |  |  |  |  |  |
| FAM83F | 167 | 3.99807710308554e-09 |  |  |  |  |  |  |
| FAM83F | 167 | 6.01908623216799e-09 |  |  |  |  |  |  |
| FAM83F | 168 | 3.03999168941313e-09 |  |  |  |  |  |  |
| FAM83F | 168 | 4.65837417088156e-09 |  |  |  |  |  |  |
| FAM83F | 168 | 5.69019594626625e-09 |  |  |  |  |  |  |
| FAM83F | 170 | 8.06311758068897e-09 |  |  |  |  |  |  |
| FAM83F | 170 | 1.11544253765797e-08 |  |  |  |  |  |  |
| FAM83F | 171 | 1.53017858820721e-08 |  |  |  |  |  |  |
| FAM83F | 171 | 2.26301733266173e-08 |  |  |  |  |  |  |
| FAM83F | 171 | 3.67962756486092e-08 |  |  |  |  |  |  |
| FAM83F | 172 | 5.72450383107946e-08 |  |  |  |  |  |  |
| FAM83F | 172 | 8.25772080730627e-08 |  |  |  |  |  |  |
| FAM83F | 172 | 1.31062333970983e-07 |  |  |  |  |  |  |
| FAM83F | 173 | 9.21121774505778e-08 |  |  |  |  |  |  |
| FAM83F | 175 | 1.44108723251526e-07 |  |  |  |  |  |  |
| FAM83F | 175 | 1.89879425207386e-07 |  |  |  |  |  |  |
| FAM83F | 175 | 3.01676076385643e-07 |  |  |  |  |  |  |
| FAM83F | 175 | 2.23031013022601e-07 |  |  |  |  |  |  |
| FAM83F | 176 | 2.05908452670703e-07 |  |  |  |  |  |  |
| FAM83F | 176 | 1.35330813652334e-07 |  |  |  |  |  |  |
| FAM83F | 176 | 1.89416087645576e-07 |  |  |  |  |  |  |
| FAM83F | 177 | 3.02191010700614e-07 |  |  |  |  |  |  |
| FAM83F | 177 | 2.07522022896515e-07 |  |  |  |  |  |  |
| FAM83F | 178 | 2.44985237810301e-07 |  |  |  |  |  |  |
| FAM83F | 178 | 3.92229371836293e-07 |  |  |  |  |  |  |
| FAM83F | 178 | 3.51774592977729e-07 |  |  |  |  |  |  |
| FAM83F | 178 | 2.18305279297877e-07 |  |  |  |  |  |  |
| FAM83F | 178 | 2.40080794126563e-07 |  |  |  |  |  |  |
| FAM83F | 178 | 2.45499448076844e-07 |  |  |  |  |  |  |
| FAM83F | 179 | 2.20894372435816e-07 |  |  |  |  |  |  |
| FAM83F | 179 | 1.61855740368189e-07 |  |  |  |  |  |  |
| FAM83F | 179 | 1.20820763396431e-07 |  |  |  |  |  |  |
| FAM83F | 179 | 1.89381924639319e-07 |  |  |  |  |  |  |
| FAM83F | 179 | 2.26230466794494e-07 |  |  |  |  |  |  |
| FAM83F | 179 | 1.80897876217898e-07 |  |  |  |  |  |  |
| FAM83F | 180 | 1.30387054268759e-07 |  |  |  |  |  |  |
| FAM83F | 180 | 1.03643461006687e-07 |  |  |  |  |  |  |
| FAM83F | 180 | 6.54843895246601e-08 |  |  |  |  |  |  |
| FAM83F | 181 | 5.16962488316081e-08 |  |  |  |  |  |  |
| FAM83F | 181 | 4.1353202006719e-08 |  |  |  |  |  |  |
| FAM83F | 182 | 6.13992079855661e-08 |  |  |  |  |  |  |
| FAM83F | 182 | 4.42512659457571e-08 |  |  |  |  |  |  |
| FAM83F | 182 | 4.23786464306278e-08 |  |  |  |  |  |  |
| FAM83F | 184 | 3.09657072968793e-08 |  |  |  |  |  |  |
| FAM83F | 185 | 2.24110741774514e-08 |  |  |  |  |  |  |
| FAM83F | 186 | 2.99122112078305e-08 |  |  |  |  |  |  |
| FAM83F | 186 | 4.50761338367957e-08 |  |  |  |  |  |  |
| FAM83F | 186 | 6.270826770871e-08 |  |  |  |  |  |  |
| FAM83F | 187 | 3.20349497082577e-08 |  |  |  |  |  |  |
| FAM83F | 188 | 4.87802521955752e-08 |  |  |  |  |  |  |
| FAM83F | 188 | 7.48079061736473e-08 |  |  |  |  |  |  |
| FAM83F | 188 | 1.13637479881574e-07 |  |  |  |  |  |  |
| FAM83F | 188 | 1.57542184441672e-07 |  |  |  |  |  |  |
| FAM83F | 189 | 2.37968250129747e-07 |  |  |  |  |  |  |
| FAM83F | 189 | 1.53662026958903e-07 |  |  |  |  |  |  |
| FAM83F | 189 | 1.75356755562717e-07 |  |  |  |  |  |  |
| FAM83F | 191 | 1.40845845798726e-07 |  |  |  |  |  |  |
| FAM83F | 191 | 2.09913465104039e-07 |  |  |  |  |  |  |
| FAM83F | 192 | 3.03974399089647e-07 |  |  |  |  |  |  |
| FAM83F | 192 | 1.80163837019328e-07 |  |  |  |  |  |  |
| FAM83F | 193 | 1.28065567846978e-07 |  |  |  |  |  |  |
| FAM83F | 193 | 6.65559937452818e-08 |  |  |  |  |  |  |
| FAM83F | 193 | 3.50712884493141e-08 |  |  |  |  |  |  |
| FAM83F | 194 | 2.37385931081217e-08 |  |  |  |  |  |  |
| FAM83F | 194 | 3.96465335051728e-08 |  |  |  |  |  |  |
| FAM83F | 194 | 5.56196093473231e-08 |  |  |  |  |  |  |
| FAM83F | 195 | 8.65790779468721e-08 |  |  |  |  |  |  |
| FAM83F | 195 | 1.39786855160592e-07 |  |  |  |  |  |  |
| FAM83F | 195 | 2.13865555940792e-07 |  |  |  |  |  |  |
| FAM83F | 196 | 2.76131697529243e-07 |  |  |  |  |  |  |
| FAM83F | 197 | 2.7809437208099e-07 |  |  |  |  |  |  |
| FAM83F | 197 | 1.81025967586472e-07 |  |  |  |  |  |  |
| FAM83F | 197 | 1.59555986892698e-07 |  |  |  |  |  |  |
| FAM83F | 198 | 2.36723346708445e-07 |  |  |  |  |  |  |
| FAM83F | 198 | 1.31495881623671e-07 |  |  |  |  |  |  |
| FAM83F | 198 | 1.65081578315579e-07 |  |  |  |  |  |  |
| FAM83F | 198 | 2.46125135977523e-07 |  |  |  |  |  |  |
| FAM83F | 198 | 1.73068317618081e-07 |  |  |  |  |  |  |
| FAM83F | 199 | 2.80945839144639e-07 |  |  |  |  |  |  |
| FAM83F | 199 | 2.03606962015617e-07 |  |  |  |  |  |  |
| FAM83F | 199 | 3.23025565309964e-07 |  |  |  |  |  |  |
| FAM83F | 199 | 4.41673108595478e-07 |  |  |  |  |  |  |
| FAM83F | 199 | 5.99080570084651e-07 |  |  |  |  |  |  |
| FAM83F | 200 | 4.1034456270819e-07 |  |  |  |  |  |  |
| FAM83F | 200 | 2.27243049345259e-07 |  |  |  |  |  |  |
| FAM83F | 200 | 1.63768468839975e-07 |  |  |  |  |  |  |
| FAM83F | 200 | 2.69120372981484e-07 |  |  |  |  |  |  |
| FAM83F | 201 | 4.42651195060294e-07 |  |  |  |  |  |  |
| FAM83F | 201 | 7.19644436463189e-07 |  |  |  |  |  |  |
| FAM83F | 201 | 5.79645867121816e-07 |  |  |  |  |  |  |
| FAM83F | 201 | 9.32827975910766e-07 |  |  |  |  |  |  |
| FAM83F | 201 | 1.471061985853e-06 |  |  |  |  |  |  |
| FAM83F | 202 | 1.07902740451793e-06 |  |  |  |  |  |  |
| FAM83F | 202 | 7.77083153442788e-07 |  |  |  |  |  |  |
| FAM83F | 202 | 3.55685465529972e-07 |  |  |  |  |  |  |
| FAM83F | 204 | 4.48949303674057e-07 |  |  |  |  |  |  |
| FAM83F | 204 | 6.00095771046167e-07 |  |  |  |  |  |  |
| FAM83F | 204 | 9.34493297452971e-07 |  |  |  |  |  |  |
| FAM83F | 205 | 6.9258301049859e-07 |  |  |  |  |  |  |
| FAM83F | 205 | 9.68775490523165e-07 |  |  |  |  |  |  |
| FAM83F | 206 | 7.0724902004728e-07 |  |  |  |  |  |  |
| FAM83F | 207 | 1.10591615485741e-06 |  |  |  |  |  |  |
| FAM83F | 207 | 8.42858056266784e-07 |  |  |  |  |  |  |
| FAM83F | 208 | 6.17053560264325e-07 |  |  |  |  |  |  |
| FAM83F | 208 | 4.95704995505179e-07 |  |  |  |  |  |  |
| FAM83F | 208 | 7.14514098988602e-07 |  |  |  |  |  |  |
| FAM83F | 209 | 2.5651520741349e-07 |  |  |  |  |  |  |
| FAM83F | 209 | 3.46397763292919e-07 |  |  |  |  |  |  |
| FAM83F | 211 | 5.14649419901686e-07 |  |  |  |  |  |  |
| FAM83F | 211 | 8.24581311477027e-07 |  |  |  |  |  |  |
| FAM83F | 211 | 5.87015916008483e-07 |  |  |  |  |  |  |
| FAM83F | 212 | 9.27165177275139e-07 |  |  |  |  |  |  |
| FAM83F | 212 | 7.72873879371323e-07 |  |  |  |  |  |  |
| FAM83F | 213 | 4.495822692292e-07 |  |  |  |  |  |  |
| FAM83F | 213 | 5.19322627571599e-07 |  |  |  |  |  |  |
| FAM83F | 213 | 8.40247928522312e-07 |  |  |  |  |  |  |
| FAM83F | 214 | 5.75326913094103e-07 |  |  |  |  |  |  |
| FAM83F | 214 | 3.1012895572674e-07 |  |  |  |  |  |  |
| FAM83F | 214 | 5.07268708103916e-07 |  |  |  |  |  |  |
| FAM83F | 214 | 6.97436908614624e-07 |  |  |  |  |  |  |
| FAM83F | 214 | 7.81262930033672e-07 |  |  |  |  |  |  |
| FAM83F | 215 | 1.28053143492458e-06 |  |  |  |  |  |  |
| FAM83F | 216 | 1.66077327446258e-06 |  |  |  |  |  |  |
| FAM83F | 217 | 1.47223334731481e-06 |  |  |  |  |  |  |
| FAM83F | 217 | 1.10880037388878e-06 |  |  |  |  |  |  |
| FAM83H | 766 | 0.045445227451989 |  |  |  |  |  |  |
| FAM83H | 766 | 0.0397593804336925 |  |  |  |  |  |  |
| FAM83H | 766 | 0.0460347008545035 |  |  |  |  |  |  |
| FAM83H | 767 | 0.0559544954879752 |  |  |  |  |  |  |
| FAM83H | 768 | 0.0482140571738295 |  |  |  |  |  |  |
| FAM83H | 768 | 0.0476307828524003 |  |  |  |  |  |  |
| FAM83H | 775 | 0.0504651545035334 |  |  |  |  |  |  |
| FAM83H | 776 | 0.0445549505462027 |  |  |  |  |  |  |
| FAM83H | 776 | 0.0379532495984137 |  |  |  |  |  |  |
| FAM83H | 777 | 0.0335614292677058 |  |  |  |  |  |  |
| FAM83H | 778 | 0.0293456872286373 |  |  |  |  |  |  |
| FAM83H | 778 | 0.0365880704367319 |  |  |  |  |  |  |
| FAM83H | 779 | 0.0322690099085521 |  |  |  |  |  |  |
| FAM83H | 780 | 0.0358908057207389 |  |  |  |  |  |  |
| FAM83H | 781 | 0.028487735854375 |  |  |  |  |  |  |
| FAM83H | 781 | 0.0265154813537666 |  |  |  |  |  |  |
| FAM83H | 783 | 0.0249707369920843 |  |  |  |  |  |  |
| FAM83H | 783 | 0.0216345152828415 |  |  |  |  |  |  |
| FAM83H | 790 | 0.025473702195523 |  |  |  |  |  |  |
| FAM83H | 791 | 0.0214931758050349 |  |  |  |  |  |  |
| FAM83H | 791 | 0.026353860831108 |  |  |  |  |  |  |
| FAM83H | 794 | 0.0325739861654199 |  |  |  |  |  |  |
| FAM83H | 795 | 0.0271568822964344 |  |  |  |  |  |  |
| FAM83H | 797 | 0.0236792465468718 |  |  |  |  |  |  |
| FAM83H | 797 | 0.027208625445294 |  |  |  |  |  |  |
| FAM83H | 797 | 0.0312560740304378 |  |  |  |  |  |  |
| FAM83H | 797 | 0.0374741669694336 |  |  |  |  |  |  |
| FAM83H | 799 | 0.045128380684084 |  |  |  |  |  |  |
| FAM83H | 801 | 0.0536369799956686 |  |  |  |  |  |  |
| FAM83H | 802 | 0.0612547737511538 |  |  |  |  |  |  |
| FAM83H | 803 | 0.0530569832934992 |  |  |  |  |  |  |
| FAM83H | 804 | 0.0438545128550834 |  |  |  |  |  |  |
| FAM83H | 804 | 0.0496741531009037 |  |  |  |  |  |  |
| FAM83H | 806 | 0.0466064000216049 |  |  |  |  |  |  |
| FAM83H | 810 | 0.0390995604196615 |  |  |  |  |  |  |
| FAM83H | 810 | 0.0360253376318275 |  |  |  |  |  |  |
| FAM83H | 817 | 0.0314970914096989 |  |  |  |  |  |  |
| FAM83H | 821 | 0.0386097522501945 |  |  |  |  |  |  |
| FAM83H | 822 | 0.0350377446994013 |  |  |  |  |  |  |
| FAM83H | 823 | 0.031185961077703 |  |  |  |  |  |  |
| FAM83H | 825 | 0.0267174195027993 |  |  |  |  |  |  |
| FAM83H | 825 | 0.0298432583037101 |  |  |  |  |  |  |
| FAM83H | 826 | 0.0364623715018258 |  |  |  |  |  |  |
| FAM83H | 828 | 0.044708859987051 |  |  |  |  |  |  |
| FAM83H | 830 | 0.0382894670648642 |  |  |  |  |  |  |
| FAM83H | 830 | 0.0433773426036012 |  |  |  |  |  |  |
| FAM83H | 831 | 0.0387760557493807 |  |  |  |  |  |  |
| FAM83H | 831 | 0.0341652366765959 |  |  |  |  |  |  |
| FAM83H | 834 | 0.0304907559180331 |  |  |  |  |  |  |
| FAM83H | 840 | 0.0263068529607818 |  |  |  |  |  |  |
| FAM83H | 841 | 0.0224816580178617 |  |  |  |  |  |  |
| FAM83H | 842 | 0.0142235019042384 |  |  |  |  |  |  |
| FAM83H | 843 | 0.0179417488612732 |  |  |  |  |  |  |
| FAM83H | 844 | 0.0217651225809041 |  |  |  |  |  |  |
| FAM83H | 846 | 0.024970621814782 |  |  |  |  |  |  |
| FAM83H | 847 | 0.0218979920881823 |  |  |  |  |  |  |
| FAM83H | 847 | 0.0230104687775717 |  |  |  |  |  |  |
| FAM83H | 847 | 0.0200815871891068 |  |  |  |  |  |  |
| FAM83H | 848 | 0.0156364468974741 |  |  |  |  |  |  |
| FAM83H | 850 | 0.0127102035068158 |  |  |  |  |  |  |
| FAM83H | 851 | 0.0114011976855901 |  |  |  |  |  |  |
| FAM83H | 852 | 0.0136652633758417 |  |  |  |  |  |  |
| FAM83H | 854 | 0.0117188988363417 |  |  |  |  |  |  |
| FAM83H | 855 | 0.00871159089076499 |  |  |  |  |  |  |
| FAM83H | 860 | 0.0100449999275159 |  |  |  |  |  |  |
| FAM83H | 861 | 0.00847958084236982 |  |  |  |  |  |  |
| FAM83H | 865 | 0.00680473853445002 |  |  |  |  |  |  |
| FAM83H | 868 | 0.0046445928985534 |  |  |  |  |  |  |
| FAM83H | 869 | 0.00373705511658407 |  |  |  |  |  |  |
| FAM83H | 870 | 0.00480244834132115 |  |  |  |  |  |  |
| FAM83H | 870 | 0.0053275462490007 |  |  |  |  |  |  |
| FAM83H | 871 | 0.0063349468155998 |  |  |  |  |  |  |
| FAM83H | 873 | 0.00809802112094841 |  |  |  |  |  |  |
| FAM83H | 873 | 0.0101247504907839 |  |  |  |  |  |  |
| FAM83H | 876 | 0.00750371947930101 |  |  |  |  |  |  |
| FAM83H | 876 | 0.00551084171812885 |  |  |  |  |  |  |
| FAM83H | 877 | 0.00714148701181454 |  |  |  |  |  |  |
| FAM83H | 878 | 0.00837931281902507 |  |  |  |  |  |  |
| FAM83H | 880 | 0.0107997270212495 |  |  |  |  |  |  |
| FAM83H | 883 | 0.0106334743139757 |  |  |  |  |  |  |
| FAM83H | 884 | 0.0100647514100396 |  |  |  |  |  |  |
| FAM83H | 886 | 0.0104953672799362 |  |  |  |  |  |  |
| FAM83H | 887 | 0.00944287811824854 |  |  |  |  |  |  |
| FAM83H | 887 | 0.00867687472883563 |  |  |  |  |  |  |
| FAM83H | 888 | 0.00798456183306321 |  |  |  |  |  |  |
| FAM83H | 888 | 0.00699580317802569 |  |  |  |  |  |  |
| FAM83H | 890 | 0.00605891338838893 |  |  |  |  |  |  |
| FAM83H | 891 | 0.00667113655817297 |  |  |  |  |  |  |
| FAM83H | 892 | 0.00568152616414011 |  |  |  |  |  |  |
| FAM83H | 892 | 0.00686269254404796 |  |  |  |  |  |  |
| FAM83H | 897 | 0.00581977979615645 |  |  |  |  |  |  |
| FAM83H | 898 | 0.00503972117410043 |  |  |  |  |  |  |
| FAM83H | 898 | 0.00366446925701201 |  |  |  |  |  |  |
| FAM83H | 901 | 0.00284446523885002 |  |  |  |  |  |  |
| FAM83H | 903 | 0.00241679870791629 |  |  |  |  |  |  |
| FAM83H | 906 | 0.00301473236763851 |  |  |  |  |  |  |
| FAM83H | 911 | 0.00268294063258171 |  |  |  |  |  |  |
| FAM83H | 911 | 0.00292360244929444 |  |  |  |  |  |  |
| FAM83H | 913 | 0.0024803805161605 |  |  |  |  |  |  |
| FAM83H | 913 | 0.0031111002936826 |  |  |  |  |  |  |
| FAM83H | 916 | 0.00265547873228921 |  |  |  |  |  |  |
| FAM83H | 916 | 0.00347409007056843 |  |  |  |  |  |  |
| FAM83H | 919 | 0.00404220415343218 |  |  |  |  |  |  |
| FAM83H | 921 | 0.00472656298653803 |  |  |  |  |  |  |
| FAM83H | 923 | 0.00402086377959395 |  |  |  |  |  |  |
| FAM83H | 923 | 0.00500488398207925 |  |  |  |  |  |  |
| FAM83H | 924 | 0.00460312289244336 |  |  |  |  |  |  |
| FAM83H | 926 | 0.0055071638670756 |  |  |  |  |  |  |
| FAM83H | 926 | 0.00446043195156425 |  |  |  |  |  |  |
| FAM83H | 926 | 0.00390608336099978 |  |  |  |  |  |  |
| FAM83H | 932 | 0.0036018495973055 |  |  |  |  |  |  |
| FAM83H | 933 | 0.00415634590073525 |  |  |  |  |  |  |
| FAM83H | 933 | 0.00301206640226171 |  |  |  |  |  |  |
| FAM83H | 933 | 0.00391258783660048 |  |  |  |  |  |  |
| FAM83H | 933 | 0.00336689288384238 |  |  |  |  |  |  |
| FAM83H | 934 | 0.00427887679510899 |  |  |  |  |  |  |
| FAM83H | 936 | 0.00509183657372754 |  |  |  |  |  |  |
| FAM83H | 937 | 0.00651515038464595 |  |  |  |  |  |  |
| FAM83H | 937 | 0.00545806687554196 |  |  |  |  |  |  |
| FAM83H | 937 | 0.00658420780257709 |  |  |  |  |  |  |
| FAM83H | 938 | 0.00568203252407431 |  |  |  |  |  |  |
| FAM83H | 941 | 0.00665476828540473 |  |  |  |  |  |  |
| FAM83H | 945 | 0.00585283625038952 |  |  |  |  |  |  |
| FAM83H | 946 | 0.00499845760219835 |  |  |  |  |  |  |
| FAM83H | 947 | 0.00576402051549717 |  |  |  |  |  |  |
| FAM83H | 949 | 0.00451892763507587 |  |  |  |  |  |  |
| FAM83H | 950 | 0.00418335592604449 |  |  |  |  |  |  |
| FAM83H | 950 | 0.00350831989458105 |  |  |  |  |  |  |
| FAM83H | 952 | 0.00404462114483146 |  |  |  |  |  |  |
| FAM83H | 952 | 0.00512239770579259 |  |  |  |  |  |  |
| FAM83H | 953 | 0.00607163515264777 |  |  |  |  |  |  |
| FAM83H | 954 | 0.00776223401496718 |  |  |  |  |  |  |
| FAM83H | 957 | 0.00920945524084207 |  |  |  |  |  |  |
| FAM83H | 959 | 0.0079219567139024 |  |  |  |  |  |  |
| FAM83H | 959 | 0.00598153043525522 |  |  |  |  |  |  |
| FAM83H | 960 | 0.00685842897446072 |  |  |  |  |  |  |
| FAM83H | 961 | 0.00620019616085017 |  |  |  |  |  |  |
| FAM83H | 965 | 0.00611445497573763 |  |  |  |  |  |  |
| FAM83H | 965 | 0.00493696046576607 |  |  |  |  |  |  |
| FAM83H | 966 | 0.00429565458265429 |  |  |  |  |  |  |
| FAM83H | 970 | 0.00363716875888755 |  |  |  |  |  |  |
| FAM83H | 970 | 0.00306180924878508 |  |  |  |  |  |  |
| FAM83H | 971 | 0.00344323653418878 |  |  |  |  |  |  |
| FAM83H | 971 | 0.00364107345006413 |  |  |  |  |  |  |
| FAM83H | 973 | 0.00309280049952092 |  |  |  |  |  |  |
| FAM83H | 975 | 0.0025288570792152 |  |  |  |  |  |  |
| FAM83H | 975 | 0.00313770698385756 |  |  |  |  |  |  |
| FAM83H | 975 | 0.00385594451359263 |  |  |  |  |  |  |
| FAM83H | 977 | 0.00455489640017094 |  |  |  |  |  |  |
| FAM83H | 981 | 0.00547140549123712 |  |  |  |  |  |  |
| FAM83H | 987 | 0.00666243544810339 |  |  |  |  |  |  |
| FAM83H | 992 | 0.00546962224925096 |  |  |  |  |  |  |
| FAM83H | 995 | 0.00647749167511147 |  |  |  |  |  |  |
| FAM83H | 997 | 0.0073714564039311 |  |  |  |  |  |  |
| FAM83H | 998 | 0.00632368766309509 |  |  |  |  |  |  |
| FAM83H | 1000 | 0.00771935209773832 |  |  |  |  |  |  |
| FAM83H | 1001 | 0.00943476853681375 |  |  |  |  |  |  |
| FAM83H | 1010 | 0.0113782753476894 |  |  |  |  |  |  |
| FAM83H | 1012 | 0.0121522423660277 |  |  |  |  |  |  |
| FAM83H | 1017 | 0.0105533010234604 |  |  |  |  |  |  |
| FAM83H | 1017 | 0.0131128624795444 |  |  |  |  |  |  |
| FAM83H | 1018 | 0.0116544407619271 |  |  |  |  |  |  |
| FAM83H | 1020 | 0.010132351508289 |  |  |  |  |  |  |
| FAM83H | 1022 | 0.011006328540571 |  |  |  |  |  |  |
| FAM83H | 1024 | 0.010053504108921 |  |  |  |  |  |  |
| FAM83H | 1025 | 0.0118947527524311 |  |  |  |  |  |  |
| FAM83H | 1025 | 0.0143303058382341 |  |  |  |  |  |  |
| FAM83H | 1026 | 0.0119669442939662 |  |  |  |  |  |  |
| FAM83H | 1026 | 0.00941562372828571 |  |  |  |  |  |  |
| FAM83H | 1028 | 0.0118051787357134 |  |  |  |  |  |  |
| FAM83H | 1032 | 0.01188540798786 |  |  |  |  |  |  |
| FAM83H | 1032 | 0.0150366229338357 |  |  |  |  |  |  |
| FAM83H | 1035 | 0.0170092626596879 |  |  |  |  |  |  |
| FAM83H | 1036 | 0.0204969353537417 |  |  |  |  |  |  |
| FAM83H | 1039 | 0.0225382460993791 |  |  |  |  |  |  |
| FAM83H | 1039 | 0.0264318581552625 |  |  |  |  |  |  |
| FAM83H | 1040 | 0.0215169694523812 |  |  |  |  |  |  |
| FAM83H | 1042 | 0.0197229088176939 |  |  |  |  |  |  |
| FAM83H | 1043 | 0.0183214140301705 |  |  |  |  |  |  |
| FAM83H | 1047 | 0.0166417137838838 |  |  |  |  |  |  |
| FAM83H | 1051 | 0.0146181796143606 |  |  |  |  |  |  |
| FAM83H | 1053 | 0.0164382206926062 |  |  |  |  |  |  |
| FAM83H | 1055 | 0.0139473725485968 |  |  |  |  |  |  |
| FAM83H | 1055 | 0.0124795182171764 |  |  |  |  |  |  |
| FAM83H | 1056 | 0.00964566848325421 |  |  |  |  |  |  |
| FAM83H | 1057 | 0.00905940631236548 |  |  |  |  |  |  |
| FAM83H | 1059 | 0.0105541771614715 |  |  |  |  |  |  |
| FAM83H | 1061 | 0.00789766410252741 |  |  |  |  |  |  |
| FAM83H | 1063 | 0.00572178910206812 |  |  |  |  |  |  |
| FAM83H | 1064 | 0.0049213954546152 |  |  |  |  |  |  |
| FAM83H | 1066 | 0.0050340610691484 |  |  |  |  |  |  |
| FAM83H | 1067 | 0.00574174178900268 |  |  |  |  |  |  |
| FAM83H | 1068 | 0.00464409964581184 |  |  |  |  |  |  |
| FAM83H | 1070 | 0.00415288181899666 |  |  |  |  |  |  |
| FAM83H | 1073 | 0.00467036086559367 |  |  |  |  |  |  |
| FAM83H | 1075 | 0.00404128312822553 |  |  |  |  |  |  |
| FAM83H | 1078 | 0.00329388207525095 |  |  |  |  |  |  |
| FAM83H | 1082 | 0.00279369722816105 |  |  |  |  |  |  |
| FAM83H | 1083 | 0.00289664593673365 |  |  |  |  |  |  |
| FAM83H | 1084 | 0.00236733577562955 |  |  |  |  |  |  |
| FAM83H | 1084 | 0.00201514013560858 |  |  |  |  |  |  |
| FAM83H | 1085 | 0.00242250094914895 |  |  |  |  |  |  |
| FAM83H | 1089 | 0.00289902359534059 |  |  |  |  |  |  |
| FAM83H | 1089 | 0.00225959243595861 |  |  |  |  |  |  |
| FAM83H | 1089 | 0.00190617757580715 |  |  |  |  |  |  |
| FAM83H | 1093 | 0.00224862909357837 |  |  |  |  |  |  |
| FAM83H | 1093 | 0.00216288468022152 |  |  |  |  |  |  |
| FAM83H | 1095 | 0.00175084650901144 |  |  |  |  |  |  |
| FAM83H | 1095 | 0.00198128954476217 |  |  |  |  |  |  |
| FAM83H | 1096 | 0.00175582949289183 |  |  |  |  |  |  |
| FAM83H | 1100 | 0.0014576868109648 |  |  |  |  |  |  |
| FAM83H | 1101 | 0.00111679978632892 |  |  |  |  |  |  |
| FAM83H | 1101 | 0.0013753403052813 |  |  |  |  |  |  |
| FAM83H | 1104 | 0.00150944489005043 |  |  |  |  |  |  |
| FAM83H | 1105 | 0.00168149989660529 |  |  |  |  |  |  |
| FAM83H | 1105 | 0.00217662287489829 |  |  |  |  |  |  |
| FAM83H | 1106 | 0.00175082249587362 |  |  |  |  |  |  |
| FAM83H | 1107 | 0.00147140779727606 |  |  |  |  |  |  |
| FAM83H | 1111 | 0.00124273507925261 |  |  |  |  |  |  |
| FAM83H | 1113 | 0.00163552349607917 |  |  |  |  |  |  |
| FAM83H | 1115 | 0.00138675129537115 |  |  |  |  |  |  |
| FAM83H | 1116 | 0.00173730366568396 |  |  |  |  |  |  |
| FAM83H | 1117 | 0.00218091822989944 |  |  |  |  |  |  |
| FAM83H | 1118 | 0.00192498347615007 |  |  |  |  |  |  |
| FAM83H | 1119 | 0.00213407278407609 |  |  |  |  |  |  |
| FAM83H | 1119 | 0.00241737735595563 |  |  |  |  |  |  |
| FAM83H | 1121 | 0.00188920591238944 |  |  |  |  |  |  |
| FAM83H | 1121 | 0.00164239347699796 |  |  |  |  |  |  |
| FAM83H | 1121 | 0.00159199939108917 |  |  |  |  |  |  |
| FAM83H | 1126 | 0.00198702548034062 |  |  |  |  |  |  |
| FAM83H | 1126 | 0.00143191776928474 |  |  |  |  |  |  |
| FAM83H | 1127 | 0.00121539715396098 |  |  |  |  |  |  |
| FAM83H | 1127 | 0.00146446677002677 |  |  |  |  |  |  |
| FAM83H | 1128 | 0.0010332033012312 |  |  |  |  |  |  |
| FAM83H | 1130 | 0.000838242133145627 |  |  |  |  |  |  |
| FAM83H | 1136 | 0.0010030985363515 |  |  |  |  |  |  |
| FAM83H | 1137 | 0.00115664163241519 |  |  |  |  |  |  |
| FAM83H | 1138 | 0.00144342196287224 |  |  |  |  |  |  |
| FAM83H | 1138 | 0.00114165032552997 |  |  |  |  |  |  |
| FAM83H | 1139 | 0.00148729444070734 |  |  |  |  |  |  |
| FAM83H | 1141 | 0.00125554595718595 |  |  |  |  |  |  |
| FAM83H | 1142 | 0.00162428534442264 |  |  |  |  |  |  |
| FAM83H | 1146 | 0.00124210744716875 |  |  |  |  |  |  |
| FAM83H | 1146 | 0.000988227754360926 |  |  |  |  |  |  |
| FAM83H | 1147 | 0.000831900623884109 |  |  |  |  |  |  |
| FAM83H | 1152 | 0.000747934042576717 |  |  |  |  |  |  |
| FAM83H | 1153 | 0.000965178285580951 |  |  |  |  |  |  |
| FAM83H | 1155 | 0.000815793567602552 |  |  |  |  |  |  |
| FAM83H | 1155 | 0.000900876252576754 |  |  |  |  |  |  |
| FAM83H | 1157 | 0.000783845992436836 |  |  |  |  |  |  |
| FAM83H | 1160 | 0.000677955345680825 |  |  |  |  |  |  |
| FAM83H | 1162 | 0.000873616275808643 |  |  |  |  |  |  |
| FAM83H | 1163 | 0.00103892045699015 |  |  |  |  |  |  |
| FAM83H | 1164 | 0.000888471530739276 |  |  |  |  |  |  |
| FAM83H | 1167 | 0.00115831973790696 |  |  |  |  |  |  |
| FAM83H | 1168 | 0.00100879382460448 |  |  |  |  |  |  |
| FAM83H | 1169 | 0.000810935721277107 |  |  |  |  |  |  |
| FAM83H | 1172 | 0.000891066049683509 |  |  |  |  |  |  |
| FAM83H | 1174 | 0.0011762432818882 |  |  |  |  |  |  |
| FAM83H | 1176 | 0.000967223959436842 |  |  |  |  |  |  |
| FAM83H | 1181 | 0.000884825530551626 |  |  |  |  |  |  |
| FAM83H | 1183 | 0.00101484632632867 |  |  |  |  |  |  |
| FAM83H | 1184 | 0.00132326589013806 |  |  |  |  |  |  |
| FAM83H | 1184 | 0.00158842642059028 |  |  |  |  |  |  |
| FAM83H | 1186 | 0.00136325111182524 |  |  |  |  |  |  |
| FAM83H | 1190 | 0.00143041948048821 |  |  |  |  |  |  |
| FAM83H | 1192 | 0.00130561896449572 |  |  |  |  |  |  |
| FAM83H | 1193 | 0.00149122394782001 |  |  |  |  |  |  |
| FAM83H | 1194 | 0.00193616950402278 |  |  |  |  |  |  |
| FAM83H | 1195 | 0.0014193447845561 |  |  |  |  |  |  |
| FAM83H | 1196 | 0.00127711484320961 |  |  |  |  |  |  |
| FAM83H | 1197 | 0.00153954605309889 |  |  |  |  |  |  |
| FAM83H | 1197 | 0.00135230625565606 |  |  |  |  |  |  |
| FAM83H | 1200 | 0.00116779543547066 |  |  |  |  |  |  |
| FAM83H | 1201 | 0.00100356805587001 |  |  |  |  |  |  |
| FAM83H | 1202 | 0.000872075374814508 |  |  |  |  |  |  |
| FAM83H | 1206 | 0.000913994429327621 |  |  |  |  |  |  |
| FAM83H | 1207 | 0.00105435245470111 |  |  |  |  |  |  |
| FAM83H | 1207 | 0.001386998587439 |  |  |  |  |  |  |
| FAM83H | 1210 | 0.00111443414503771 |  |  |  |  |  |  |
| FAM83H | 1211 | 0.00133288988685885 |  |  |  |  |  |  |
| FAM83H | 1214 | 0.00109908617254714 |  |  |  |  |  |  |
| FAM83H | 1216 | 0.00130355098737453 |  |  |  |  |  |  |
| FAM83H | 1217 | 0.00118626732653966 |  |  |  |  |  |  |
| FAM83H | 1218 | 0.000861684625530162 |  |  |  |  |  |  |
| FAM83H | 1221 | 0.000774016776500834 |  |  |  |  |  |  |
| FAM83H | 1224 | 0.000931542029969719 |  |  |  |  |  |  |
| FAM83H | 1225 | 0.00075251524753538 |  |  |  |  |  |  |
| FAM83H | 1226 | 0.00062641259128545 |  |  |  |  |  |  |
| FAM83H | 1227 | 0.00084105023756579 |  |  |  |  |  |  |
| FAM83H | 1229 | 0.000998527014300922 |  |  |  |  |  |  |
| FAM83H | 1234 | 0.00126709397488424 |  |  |  |  |  |  |
| FAM83H | 1234 | 0.000820632065291271 |  |  |  |  |  |  |
| FAM83H | 1235 | 0.000668437889920204 |  |  |  |  |  |  |
| FAM83H | 1236 | 0.000547395207920215 |  |  |  |  |  |  |
| FAM83H | 1238 | 0.000428792270001199 |  |  |  |  |  |  |
| FAM83H | 1238 | 0.00054110739357662 |  |  |  |  |  |  |
| FAM83H | 1241 | 0.000444320548367334 |  |  |  |  |  |  |
| FAM83H | 1242 | 0.000367368154669126 |  |  |  |  |  |  |
| FAM83H | 1247 | 0.000474933473229515 |  |  |  |  |  |  |
| FAM83H | 1247 | 0.000607714025929429 |  |  |  |  |  |  |
| FAM83H | 1250 | 0.000505189113006264 |  |  |  |  |  |  |
| FAM83H | 1254 | 0.000410541190619083 |  |  |  |  |  |  |
| FAM83H | 1258 | 0.000514480622666977 |  |  |  |  |  |  |
| FAM83H | 1263 | 0.000694853426080085 |  |  |  |  |  |  |
| FAM83H | 1264 | 0.000577167697669599 |  |  |  |  |  |  |
| FAM83H | 1266 | 0.000475823823761677 |  |  |  |  |  |  |
| FAM83H | 1267 | 0.000417313156486128 |  |  |  |  |  |  |
| FAM83H | 1268 | 0.000536091164049999 |  |  |  |  |  |  |
| FAM83H | 1269 | 0.000663045504989759 |  |  |  |  |  |  |
| FAM83H | 1270 | 0.000565050623211049 |  |  |  |  |  |  |
| FAM83H | 1271 | 0.000756805786930112 |  |  |  |  |  |  |
| FAM83H | 1271 | 0.00101440340897421 |  |  |  |  |  |  |
| FAM83H | 1271 | 0.000813635563327549 |  |  |  |  |  |  |
| FAM83H | 1274 | 0.00071948183283549 |  |  |  |  |  |  |
| FAM83H | 1274 | 0.000670824410285202 |  |  |  |  |  |  |
| FAM83H | 1278 | 0.00052650244985918 |  |  |  |  |  |  |
| FAM83H | 1279 | 0.000694426660872557 |  |  |  |  |  |  |
| FAM83H | 1279 | 0.00090406530640691 |  |  |  |  |  |  |
| FAM83H | 1280 | 0.000761245080401075 |  |  |  |  |  |  |
| FAM83H | 1284 | 0.000607366313438921 |  |  |  |  |  |  |
| FAM83H | 1287 | 0.000733136330236532 |  |  |  |  |  |  |
| FAM83H | 1287 | 0.000608754151852442 |  |  |  |  |  |  |
| FAM83H | 1288 | 0.000471262919710133 |  |  |  |  |  |  |
| FAM83H | 1289 | 0.000551765879873129 |  |  |  |  |  |  |
| FAM83H | 1290 | 0.000493335382209086 |  |  |  |  |  |  |
| FAM83H | 1290 | 0.000583538698131814 |  |  |  |  |  |  |
| FAM83H | 1293 | 0.000481239417243566 |  |  |  |  |  |  |
| FAM83H | 1294 | 0.00058609500236218 |  |  |  |  |  |  |
| FAM83H | 1296 | 0.000746369247084834 |  |  |  |  |  |  |
| FAM83H | 1297 | 0.000720179053862752 |  |  |  |  |  |  |
| FAM83H | 1298 | 0.000846934387963717 |  |  |  |  |  |  |
| FAM83H | 1299 | 0.00102998564746588 |  |  |  |  |  |  |
| FAM83H | 1304 | 0.00133548307004213 |  |  |  |  |  |  |
| FAM83H | 1305 | 0.00170808618240741 |  |  |  |  |  |  |
| FAM83H | 1310 | 0.00213549327775373 |  |  |  |  |  |  |
| FAM83H | 1310 | 0.00258464478204819 |  |  |  |  |  |  |
| FAM83H | 1311 | 0.00218661523490283 |  |  |  |  |  |  |
| FAM83H | 1311 | 0.00265304370224466 |  |  |  |  |  |  |
| FAM83H | 1312 | 0.00218956150829294 |  |  |  |  |  |  |
| FAM83H | 1312 | 0.00227689299886313 |  |  |  |  |  |  |
| FAM83H | 1313 | 0.00183949252895436 |  |  |  |  |  |  |
| FAM83H | 1317 | 0.00225285908208861 |  |  |  |  |  |  |
| FAM83H | 1321 | 0.00286053196890135 |  |  |  |  |  |  |
| FAM83H | 1323 | 0.00241890146384911 |  |  |  |  |  |  |
| FAM83H | 1324 | 0.00293870487168725 |  |  |  |  |  |  |
| FAM83H | 1327 | 0.00380494105664685 |  |  |  |  |  |  |
| FAM83H | 1332 | 0.00435834388647483 |  |  |  |  |  |  |
| FAM83H | 1332 | 0.00358065493078744 |  |  |  |  |  |  |
| FAM83H | 1332 | 0.00417538698796064 |  |  |  |  |  |  |
| FAM83H | 1333 | 0.00531347455030305 |  |  |  |  |  |  |
| FAM83H | 1336 | 0.00436983785573323 |  |  |  |  |  |  |
| FAM83H | 1337 | 0.00534113209684325 |  |  |  |  |  |  |
| FAM83H | 1337 | 0.00658141480989075 |  |  |  |  |  |  |
| FAM83H | 1343 | 0.00846073014755934 |  |  |  |  |  |  |
| FAM83H | 1343 | 0.00771394995450953 |  |  |  |  |  |  |
| FAM83H | 1343 | 0.00683091995215491 |  |  |  |  |  |  |
| FAM83H | 1349 | 0.0058497425489598 |  |  |  |  |  |  |
| FAM83H | 1349 | 0.00500627388481153 |  |  |  |  |  |  |
| FAM83H | 1351 | 0.00644942016155291 |  |  |  |  |  |  |
| FAM83H | 1351 | 0.00576886380087908 |  |  |  |  |  |  |
| FAM83H | 1351 | 0.00515539652662409 |  |  |  |  |  |  |
| FAM83H | 1358 | 0.00436889945529768 |  |  |  |  |  |  |
| FAM83H | 1358 | 0.00501621962679677 |  |  |  |  |  |  |
| FAM83H | 1363 | 0.0065003592524422 |  |  |  |  |  |  |
| FAM83H | 1364 | 0.00534781150406296 |  |  |  |  |  |  |
| FAM83H | 1365 | 0.00387204856216404 |  |  |  |  |  |  |
| FAM83H | 1365 | 0.00257929569250982 |  |  |  |  |  |  |
| FAM83H | 1366 | 0.00216209920788163 |  |  |  |  |  |  |
| FAM83H | 1367 | 0.00268688400138621 |  |  |  |  |  |  |
| FAM83H | 1373 | 0.00331223191964162 |  |  |  |  |  |  |
| FAM83H | 1374 | 0.0030486095540499 |  |  |  |  |  |  |
| FAM83H | 1375 | 0.00359273866165867 |  |  |  |  |  |  |
| FAM83H | 1376 | 0.00400220429788366 |  |  |  |  |  |  |
| FAM83H | 1378 | 0.00447249548740152 |  |  |  |  |  |  |
| FAM83H | 1381 | 0.00512815053929299 |  |  |  |  |  |  |
| FAM83H | 1384 | 0.00415662879549254 |  |  |  |  |  |  |
| FAM83H | 1389 | 0.00531364830264377 |  |  |  |  |  |  |
| FAM83H | 1391 | 0.00626507313911574 |  |  |  |  |  |  |
| FAM83H | 1392 | 0.0075686733384567 |  |  |  |  |  |  |
| FAM83H | 1397 | 0.00803067186209348 |  |  |  |  |  |  |
| FAM83H | 1403 | 0.00971295266621713 |  |  |  |  |  |  |
| FAM83H | 1405 | 0.0123809953400746 |  |  |  |  |  |  |
| FAM83H | 1407 | 0.0145727663604614 |  |  |  |  |  |  |
| FAM83H | 1408 | 0.0173442932501655 |  |  |  |  |  |  |
| FAM83H | 1408 | 0.0188427175136437 |  |  |  |  |  |  |
| FAM83H | 1412 | 0.0158920925896814 |  |  |  |  |  |  |
| FAM83H | 1413 | 0.012571424581316 |  |  |  |  |  |  |
| FAM83H | 1414 | 0.0106827463414578 |  |  |  |  |  |  |
| FAM83H | 1415 | 0.00930172566541293 |  |  |  |  |  |  |
| FAM83H | 1421 | 0.011787336568218 |  |  |  |  |  |  |
| FAM83H | 1425 | 0.0138856124113457 |  |  |  |  |  |  |
| FAM83H | 1425 | 0.0171475425636377 |  |  |  |  |  |  |
| FAM83H | 1426 | 0.0202524241668283 |  |  |  |  |  |  |
| FAM83H | 1428 | 0.017198771445268 |  |  |  |  |  |  |
| FAM83H | 1431 | 0.0149076522379257 |  |  |  |  |  |  |
| FAM83H | 1439 | 0.0187433919535736 |  |  |  |  |  |  |
| FAM83H | 1442 | 0.02227209225424 |  |  |  |  |  |  |
| FAM83H | 1443 | 0.0194263751321187 |  |  |  |  |  |  |
| FAM83H | 1444 | 0.0158616611715893 |  |  |  |  |  |  |
| FAM83H | 1444 | 0.0172478102392215 |  |  |  |  |  |  |
| FAM83H | 1446 | 0.0141308576902261 |  |  |  |  |  |  |
| FAM83H | 1451 | 0.0162628884861314 |  |  |  |  |  |  |
| FAM83H | 1455 | 0.0178651712046894 |  |  |  |  |  |  |
| FAM83H | 1456 | 0.0216255892159157 |  |  |  |  |  |  |
| FAM83H | 1458 | 0.0232349100721365 |  |  |  |  |  |  |
| FAM83H | 1460 | 0.0242823375627621 |  |  |  |  |  |  |
| FAM83H | 1461 | 0.0185788870188983 |  |  |  |  |  |  |
| FAM83H | 1461 | 0.0161332534520114 |  |  |  |  |  |  |
| FAM83H | 1463 | 0.0140689663321538 |  |  |  |  |  |  |
| FAM83H | 1465 | 0.0178165814546171 |  |  |  |  |  |  |
| FAM83H | 1469 | 0.0140901129529359 |  |  |  |  |  |  |
| FAM83H | 1470 | 0.0133050935760106 |  |  |  |  |  |  |
| FAM83H | 1472 | 0.015322728777357 |  |  |  |  |  |  |
| FAM83H | 1473 | 0.0191363430889688 |  |  |  |  |  |  |
| FAM83H | 1473 | 0.0162840200315905 |  |  |  |  |  |  |
| FAM83H | 1473 | 0.0140178247773805 |  |  |  |  |  |  |
| FAM83H | 1475 | 0.0173614437404444 |  |  |  |  |  |  |
| FAM83H | 1480 | 0.0137060311501164 |  |  |  |  |  |  |
| FAM83H | 1482 | 0.0170245010483556 |  |  |  |  |  |  |
| FAM83H | 1488 | 0.0151720482862951 |  |  |  |  |  |  |
| FAM83H | 1488 | 0.0183300123617783 |  |  |  |  |  |  |
| FAM83H | 1489 | 0.0169634006903705 |  |  |  |  |  |  |
| FAM83H | 1499 | 0.0151751293258224 |  |  |  |  |  |  |
| FAM83H | 1500 | 0.0190806859384706 |  |  |  |  |  |  |
| FAM83H | 1501 | 0.0215955851282059 |  |  |  |  |  |  |
| FAM83H | 1503 | 0.0189021665923194 |  |  |  |  |  |  |
| FAM83H | 1508 | 0.0197278369630721 |  |  |  |  |  |  |
| FAM83H | 1508 | 0.0246128903256764 |  |  |  |  |  |  |
| FAM83H | 1512 | 0.02646187980284 |  |  |  |  |  |  |
| FAM83H | 1516 | 0.0229308765748245 |  |  |  |  |  |  |
| FAM83H | 1517 | 0.0208679738028604 |  |  |  |  |  |  |
| FAM83H | 1521 | 0.0254921909868268 |  |  |  |  |  |  |
| FAM83H | 1523 | 0.0193515492140256 |  |  |  |  |  |  |
| FAM83H | 1523 | 0.0175749882194722 |  |  |  |  |  |  |
| FAM83H | 1526 | 0.0154591921734276 |  |  |  |  |  |  |
| FAM83H | 1526 | 0.0169539629291048 |  |  |  |  |  |  |
| FAM83H | 1537 | 0.015390432704731 |  |  |  |  |  |  |
| FAM83H | 1547 | 0.015360009238963 |  |  |  |  |  |  |
| FAM83H | 1549 | 0.015409645785619 |  |  |  |  |  |  |
| FAM83H | 1549 | 0.0172383276160617 |  |  |  |  |  |  |
| FAM83H | 1551 | 0.0182607459618517 |  |  |  |  |  |  |
| FAM83H | 1552 | 0.0214824454491767 |  |  |  |  |  |  |
| FAM83H | 1554 | 0.0188098642414915 |  |  |  |  |  |  |
| FAM83H | 1555 | 0.0153164673424414 |  |  |  |  |  |  |
| FAM83H | 1555 | 0.0132942765216606 |  |  |  |  |  |  |
| FAM83H | 1556 | 0.00975459882202528 |  |  |  |  |  |  |
| FAM83H | 1557 | 0.0118815803098914 |  |  |  |  |  |  |
| FAM83H | 1557 | 0.0148027915556176 |  |  |  |  |  |  |
| FAM83H | 1565 | 0.0173466656407627 |  |  |  |  |  |  |
| FAM83H | 1566 | 0.016400344629824 |  |  |  |  |  |  |
| FAM83H | 1567 | 0.0142724865535202 |  |  |  |  |  |  |
| FAM83H | 1571 | 0.0123700323501923 |  |  |  |  |  |  |
| FAM83H | 1572 | 0.0105130077424071 |  |  |  |  |  |  |
| FAM83H | 1581 | 0.0131040453410883 |  |  |  |  |  |  |
| FAM83H | 1586 | 0.0156606619932439 |  |  |  |  |  |  |
| FAM83H | 1590 | 0.0169940807644817 |  |  |  |  |  |  |
| FAM83H | 1590 | 0.0200778958807448 |  |  |  |  |  |  |
| FAM83H | 1590 | 0.0183902236986445 |  |  |  |  |  |  |
| FAM83H | 1591 | 0.021455244474705 |  |  |  |  |  |  |
| FAM83H | 1597 | 0.0263223213560361 |  |  |  |  |  |  |
| FAM83H | 1597 | 0.0282961951004711 |  |  |  |  |  |  |
| FAM83H | 1610 | 0.0343189984599341 |  |  |  |  |  |  |
| FAM83H | 1610 | 0.0272381433896252 |  |  |  |  |  |  |
| FAM83H | 1610 | 0.0247467336267948 |  |  |  |  |  |  |
| FAM83H | 1610 | 0.0218539698274936 |  |  |  |  |  |  |
| FAM83H | 1612 | 0.019255986620728 |  |  |  |  |  |  |
| FAM83H | 1616 | 0.0220327575109394 |  |  |  |  |  |  |
| FAM83H | 1617 | 0.0174860292911224 |  |  |  |  |  |  |
| FAM83H | 1617 | 0.0150733980835269 |  |  |  |  |  |  |
| FAM83H | 1618 | 0.0191261028333097 |  |  |  |  |  |  |
| FAM83H | 1622 | 0.0233759658913194 |  |  |  |  |  |  |
| FAM83H | 1622 | 0.0247991167476917 |  |  |  |  |  |  |
| FAM83H | 1624 | 0.0202852936043892 |  |  |  |  |  |  |
| FAM83H | 1625 | 0.0175148772443559 |  |  |  |  |  |  |
| FAM83H | 1629 | 0.0222091314889821 |  |  |  |  |  |  |
| FAM83H | 1630 | 0.0228180861465121 |  |  |  |  |  |  |
| FAM83H | 1631 | 0.0191007153082868 |  |  |  |  |  |  |
| FAM83H | 1632 | 0.0178763523724948 |  |  |  |  |  |  |
| FAM83H | 1633 | 0.0153575199544682 |  |  |  |  |  |  |
| FAM83H | 1638 | 0.0132163920343255 |  |  |  |  |  |  |
| FAM83H | 1639 | 0.0131400964923971 |  |  |  |  |  |  |
| FAM83H | 1643 | 0.0166869522463503 |  |  |  |  |  |  |
| FAM83H | 1645 | 0.0143383166683956 |  |  |  |  |  |  |
| FAM83H | 1646 | 0.0179541130791594 |  |  |  |  |  |  |
| FAM83H | 1647 | 0.0215113117005244 |  |  |  |  |  |  |
| FAM83H | 1648 | 0.0247556295484745 |  |  |  |  |  |  |
| FAM83H | 1648 | 0.018952761536277 |  |  |  |  |  |  |
| FAM83H | 1649 | 0.0232433925059801 |  |  |  |  |  |  |
| FAM83H | 1649 | 0.0277590134900315 |  |  |  |  |  |  |
| FAM83H | 1650 | 0.0258342167333233 |  |  |  |  |  |  |
| FAM83H | 1656 | 0.0324514450572957 |  |  |  |  |  |  |
| FAM83H | 1660 | 0.0368145560453 |  |  |  |  |  |  |
| FAM83H | 1661 | 0.0321607978742039 |  |  |  |  |  |  |
| FAM83H | 1664 | 0.0383413839157738 |  |  |  |  |  |  |
| FAM83H | 1664 | 0.032184292895835 |  |  |  |  |  |  |
| FAM83H | 1673 | 0.0393410872787486 |  |  |  |  |  |  |
| FAM83H | 1674 | 0.0431568343382736 |  |  |  |  |  |  |
| FAM83H | 1674 | 0.0532151283218003 |  |  |  |  |  |  |
| FAM83H | 1682 | 0.0467801028225865 |  |  |  |  |  |  |
| FAM83H | 1682 | 0.0573688176793444 |  |  |  |  |  |  |
| FAM83H | 1685 | 0.0509023964548724 |  |  |  |  |  |  |
| FAM83H | 1688 | 0.049361969986405 |  |  |  |  |  |  |
| FAM83H | 1690 | 0.0547346823336186 |  |  |  |  |  |  |
| FAM83H | 1696 | 0.0651805591257451 |  |  |  |  |  |  |
| FAM83H | 1699 | 0.0538426587423627 |  |  |  |  |  |  |
| FAM83H | 1702 | 0.0637638173776552 |  |  |  |  |  |  |
| FAM83H | 1704 | 0.0628035802853541 |  |  |  |  |  |  |
| FAM83H | 1705 | 0.0730252689896435 |  |  |  |  |  |  |
| FAM83H | 1707 | 0.0640803954794515 |  |  |  |  |  |  |
| FAM83H | 1710 | 0.0702903228177572 |  |  |  |  |  |  |
| FAM83H | 1712 | 0.0767862157144596 |  |  |  |  |  |  |
| FAM83H | 1713 | 0.0846932126461126 |  |  |  |  |  |  |
| FAM83H | 1715 | 0.0661949617428595 |  |  |  |  |  |  |
| FAM83H | 1715 | 0.0768565737092314 |  |  |  |  |  |  |
| FAM83H | 1719 | 0.0780566355064636 |  |  |  |  |  |  |
| FAM83H | 1721 | 0.0935327673345353 |  |  |  |  |  |  |
| FAM83H | 1722 | 0.0820673128049803 |  |  |  |  |  |  |
| FAM83H | 1728 | 0.0848118015262922 |  |  |  |  |  |  |
| FAM83H | 1731 | 0.0744829207645885 |  |  |  |  |  |  |
| FAM83H | 1732 | 0.0654309839057089 |  |  |  |  |  |  |
| FAM83H | 1735 | 0.0756302972637757 |  |  |  |  |  |  |
| FAM83H | 1737 | 0.086684229996351 |  |  |  |  |  |  |
| FAM83H | 1741 | 0.103207743272081 |  |  |  |  |  |  |
| FAM83H | 1745 | 0.117901964003861 |  |  |  |  |  |  |
| FAM83H | 1746 | 0.133292827056237 |  |  |  |  |  |  |
| FAM83H | 1747 | 0.157313780005284 |  |  |  |  |  |  |
| FAM83H | 1754 | 0.181841954637575 |  |  |  |  |  |  |
| FAM83H | 1759 | 0.204881636851763 |  |  |  |  |  |  |
| FAM83H | 1763 | 0.182769913655067 |  |  |  |  |  |  |
| FAM83H | 1769 | 0.211725900148469 |  |  |  |  |  |  |
| FAM83H | 1770 | 0.184277523783627 |  |  |  |  |  |  |
| FAM83H | 1772 | 0.199281351225475 |  |  |  |  |  |  |
| FAM83H | 1772 | 0.223530864853136 |  |  |  |  |  |  |
| FAM83H | 1773 | 0.259647541017538 |  |  |  |  |  |  |
| FAM83H | 1775 | 0.282184020691902 |  |  |  |  |  |  |
| FAM83H | 1778 | 0.320035355871691 |  |  |  |  |  |  |
| FAM83H | 1784 | 0.36697244453265 |  |  |  |  |  |  |
| FAM83H | 1788 | 0.411615381742659 |  |  |  |  |  |  |
| FAM83H | 1790 | 0.461944518579442 |  |  |  |  |  |  |
| FAM83H | 1794 | 0.461057905921788 |  |  |  |  |  |  |
| FAM83H | 1795 | 0.45642518900222 |  |  |  |  |  |  |
| FAM83H | 1800 | 0.42327261512415 |  |  |  |  |  |  |
| FAM83H | 1806 | 0.403416068224769 |  |  |  |  |  |  |
| FAM83H | 1808 | 0.37251589028959 |  |  |  |  |  |  |
| FAM83H | 1808 | 0.315297435229404 |  |  |  |  |  |  |
| FAM83H | 1809 | 0.346733570405419 |  |  |  |  |  |  |
| FAM83H | 1811 | 0.316762003922005 |  |  |  |  |  |  |
| FAM83H | 1813 | 0.267446955094466 |  |  |  |  |  |  |
| FAM83H | 1814 | 0.309655403838041 |  |  |  |  |  |  |
| FAM83H | 1816 | 0.283317306915483 |  |  |  |  |  |  |
| FAM83H | 1818 | 0.279776563309923 |  |  |  |  |  |  |
| FAM83H | 1820 | 0.314936499217259 |  |  |  |  |  |  |
| FAM83H | 1821 | 0.2952453851757 |  |  |  |  |  |  |
| FAM83H | 1821 | 0.269658922190709 |  |  |  |  |  |  |
| FAM83H | 1825 | 0.230585632144681 |  |  |  |  |  |  |
| FAM83H | 1825 | 0.206948235908317 |  |  |  |  |  |  |
| FAM83H | 1836 | 0.160024899544348 |  |  |  |  |  |  |
| FAM83H | 1842 | 0.140247959972831 |  |  |  |  |  |  |
| FAM83H | 1846 | 0.117230579148407 |  |  |  |  |  |  |
| FAM83H | 1849 | 0.129862277645895 |  |  |  |  |  |  |
| FAM83H | 1851 | 0.13675722955482 |  |  |  |  |  |  |
| FAM83H | 1852 | 0.110690806877178 |  |  |  |  |  |  |
| FAM83H | 1857 | 0.129469423594333 |  |  |  |  |  |  |
| FAM83H | 1857 | 0.144153652310035 |  |  |  |  |  |  |
| FAM83H | 1859 | 0.119239834148938 |  |  |  |  |  |  |
| FAM83H | 1864 | 0.123991305806978 |  |  |  |  |  |  |
| FAM83H | 1865 | 0.108106267934581 |  |  |  |  |  |  |
| FAM83H | 1868 | 0.11147038417649 |  |  |  |  |  |  |
| FAM83H | 1872 | 0.128265661656448 |  |  |  |  |  |  |
| FAM83H | 1874 | 0.108899755376497 |  |  |  |  |  |  |
| FAM83H | 1883 | 0.132058045672482 |  |  |  |  |  |  |
| FAM83H | 1887 | 0.153821189437267 |  |  |  |  |  |  |
