# Supplementary material for: Systematic Analysis of Expression Profiles and Prognostic Significance for FAM83 Family in Non-small-Cell Lung Cancer
Source: Front Mol Biosci. 2020 Dec 10;7:572406. doi: 10.3389/fmolb.2020.572406 (PMC7758490; doi:10.3389/fmolb.2020.572406)
Supplement: Supplementary file 5 [file Table_3.DOCX]

Supplementary Table 3. The sensitivity and specificity of FAM83 family as biomarker in NSCLC patients.

|  | OS | | FP | | PPS | |
| --- | --- | --- | --- | --- | --- | --- |
| FAM83 family | sensitivity | specificity | sensitivity | specificity | sensitivity | specificity |
| FAM83A | 0.9475 | 1 | 1 | NA | 0.952381 | 0.7586207 |
| FAM83B | 1 | NA | 0.8235294 | 0.9910714 | NA | 1 |
| FAM83C | 0.971897 | 1 | 1 | 0.9810427 | NA | 1 |
| FAM83D | 1 | NA | NA | 1 | 0.8333333 | 0.9661017 |
| FAM83E | NA | 1 | 0.3424658 | 0.9504717 | NA | 1 |
| FAM83F | 1 | NA | 0.9148936 | 0.6139241 | NA | 1 |
| FAM83H | 0.9454545 | 0.8974359 | NA | 1 | 1 | NA |
